# Guilty by association: direct interaction with the tetraspanin CD63 suggests a role for organic cation transporter 3 in histamine release from granulocytes

**DOI:** 10.1186/s12929-025-01158-2

**Published:** 2025-07-12

**Authors:** Moritz Pernecker, Miriam Dibos, Sophie Götz, Rouvier Al-Monajjed, Vivien Barz, Christian Albiker, Rita Schröter, Ute Neugebauer, Lena Ludwig-Radtke, R. Verena Taudte, Thomas Vogl, Giuliano Ciarimboli

**Affiliations:** 1 Exp. Nephrology, Med. Clinic D, University Hospital Münster, Albert-Schweitzer-Campus 1/A14, 48149 Münster, Germany; 2 Core Facility for Metabolomics, Department of Medicine, Philipps University Marburg, Marburg, Germany; 3 Institute of Immunology, University of Münster, Münster, Germany

**Keywords:** Histamine, Organic cation transporter 3, CD63, Allergy

## Abstract

**Background:**

The organic cation transporter 3 (OCT3) is a ubiquitous transporter that carries both endogenous and exogenous substrates, such as histamine and cisplatin. Our investigations have shown that OCT3 directly interacts with the tetraspanin CD63. CD63 is a marker for activated basophils and mast cells, which are granulocytes capable of rapidly releasing large amounts of histamine. This makes them key players in the development of allergic reactions.

**Methods and results:**

In this work, we demonstrated that OCT3 is present in murine and human basophils and is strongly colocalized with CD63 in a specific region of the plasma membrane, particularly after cell activation leading to histamine release. Furthermore, we confirmed that part of the histamine release from basophils is mediated by OCT3. In a mouse model of contact dermatitis, the presence of OCT3 is crucial for determining the severity of the allergic reaction. The presence of CD63 also seems to be important for regulating the allergic response, although it does not directly affect histamine secretion. RNA-Seq and metabolome analyses revealed that wild-type mice and mice with genetic deletion of OCT3 (OCT3^−/−^) are phenotypically very similar, and that the observed effects in OCT3^−/−^ organisms can be attributed mainly to the genetic deletion of the OCT3 transporter.

**Conclusions:**

In conclusion, OCT3 is a transporter for histamine in granulocytes, which plays a crucial role in determining the intensity of allergic reactions and may be a target for interventions aimed at reducing their severity.

**Supplementary Information:**

The online version contains supplementary material available at 10.1186/s12929-025-01158-2.

## Introduction

Organic cation transporters (OCTs) are integral membrane proteins categorized within the Solute Carrier Family 22A (SLC22A). OCTs play pivotal roles in physiology, pharmacology and toxicology by facilitating the movement across the plasma membrane of various compounds, including important neurotransmitters like serotonin, histamine, and dopamine [1, 2], drugs like metformin and cisplatin [3], and toxins like cadmium [4]. OCTs exhibit broad substrate specificity [5] and demonstrate distinct localization within organs and cells. In humans, OCT1, encoded by the *SLC22A1* gene, shows prominent expression in the sinusoidal membrane of hepatocytes [6], while OCT2 (*SLC22A2*) is notably concentrated in the basolateral plasma membrane of renal proximal tubular cells [7, 8]. These specific distributions highlight their roles in facilitating the secretory clearance of substrates associated with both OCT1 and OCT2. In contrast to OCT1 and OCT2, OCT3, the third subtype of OCTs, exhibits near-ubiquitous expression [9]. Apart from its interaction with numerous drugs [10], OCT3 has been suggested to play a role as a supplementary corticosterone-sensitive reuptake pathway for serotonin within the murine central nervous system [11, 12]. Furthermore, there is a proposed significant role for OCT3 in the transport of histamine [13–15]. These data suggest that OCT3 can have important physiological roles, even though they are not yet fully disclosed. To glean further insights into OCT3's functional role, we previously employed a mating-based split ubiquitin system (mbSUS) to identify its interacting partners. As Goethe's adage echoes, “Tell me with whom you associate, and I will tell you who you are” [16] understanding interaction partners becomes pivotal in predicting a protein's role in biological processes—embracing the concept of 'guilty by association'. If a protein of unknown function associates with one of known function, it reveals clues about its potential physiological role.

Utilizing the mbSUS-based interaction partner screening with human OCT3 (hOCT3) as the bait, our prior investigations successfully pinpointed CD63, a tetraspanin protein, as a prey and therefore as a promising interaction candidate of hOCT3 [17]. Tetraspanins form a crucial network of proteins known for their propensity to interact with various partners, orchestrating the assembly of multimolecular membrane microdomains termed tetraspanin-enriched microdomains [18]. Within these microdomains, tetraspanins engage with integrins, signaling enzymes, and other transmembrane proteins, fostering a dynamic association [19]. The capacity of tetraspanins to facilitate interactions among diverse proteins, culminating in the formation of functional complexes, has led to the conceptualization of these proteins as "molecular facilitators" [20]. Tetraspanins frequently play a role in vesicle fusion processes. Specifically, CD63, a widely distributed tetraspanin, localizes within both the endosomal system and the cell surface, demonstrating a trafficking pattern between these compartments [21].

This project is particularly intrigued by the observation that in human basophils, CD63 manifests on the membrane of intracellular granules containing histamine. Upon basophil activation, these granules fuse with the plasma membrane, releasing their contents (including histamine and other mediators) into the extracellular environment. Consequently, the presence of CD63 on the plasma membrane stands as a reliable marker for basophil activation [22], notably during anaphylactic degranulation [23]. Similarly, in mast cells (MCs), CD63 exhibits expression both at the cell surface and within secretory granules containing allergic mediators like histamine. The degranulation process in MCs has been correlated with the relocation of CD63 to the plasma membrane, a phenomenon reliant on microtubule dynamics [24].

Histamine plays a pivotal role in allergic diseases and stands as the primary mediator of anaphylactic shock—an immediate and potentially life-threatening systemic reaction often triggered by IgE-mediated degranulation of mast cells and basophils. The manifestation of signs and symptoms during anaphylactic shock is largely attributed to histamine binding to its receptors [25]. Considering histamine's status as a low-affinity substrate of hOCT3, the interaction between hOCT3 and CD63 becomes potentially consequential in cells characterized by rapid and substantial histamine release.

This study aims to confirm the OCT3/CD63 interaction and to delve into its plausible role in histamine release and the onset of allergic reactions, both in controlled in vitro settings and in live animal models of allergic reaction.

## Materials and methods

In a prior investigation, the mbSUS was employed as an in vivo method to screen for proteins from a cDNA plasmid library that could potentially interact with hOCT [17]. Notably, the study identified CD63 as a potential interaction partner of hOCT3. To validate this interaction, we conducted further experiments using the mbSUS technique. In these experiments, bait-expressing yeast (specifically, THY.AP4) was used, which expressed hOCT3 cloned in-frame into the expression vector pMETYCgate. This vector contains the C-terminal half of ubiquitin (Cub) and the transcription factor protA-LexA-VP16 (PLV), forming the construct hOCT3-Cub-PLV. These bait yeast strains were then mated with prey-expressing yeast (THY.AP5) that carried the N-terminal half of ubiquitin (Nub) fused with a prey protein, specifically CD63, in the pNXgate33 vector, resulting in the construct CD63-Nub. As a negative control, we utilized the pNXgate33 vector alone. For positive control purposes, the pNubWTXgate vector, expressing the wild-type Nub without mutation, was employed as it reconstitutes ubiquitin without interaction. Successful mating led to the formation of diploid cells harboring two plasmids with their respective Cub and Nub fusions. The selection of diploid forms containing both plasmids was achieved through media that lacked leucine (Leu, for Cub vector selection) and tryptophan (Trp, for NubG vector selection). The assessment of interaction was subsequently conducted by observing the growth on media that also lacked adenine (Ade) and histidine (His).

### Cell culture, transfection, and pull-down experiments

HEK293 cells (CRL-1573; American Type Culture Collection, Manassas, VA, USA) were cultured under standard conditions. The wild-type (WT) HEK293 cells and HEK293 cells stably expressing hOCT3 were maintained at 37 °C in Dulbecco's Modified Eagle Medium (DMEM) supplemented with 3.7 g/L NaHCO_3_, 1.0 g/l D-glucose, and 2.0 mM L-glutamine (Biochrom, Berlin, Germany). The culture medium was equilibrated with 8% CO_2_ and further supplemented with 100 U/ml of penicillin, 100 mg/l of streptomycin (both from Biochrom), 10% fetal bovine serum, and, for hOCT3 cells, 0.8 mg/ml of geneticin (PAA Laboratories, Coelbe, Germany). HEK293 WT and hOCT3-expressing cells were transiently transfected with either control vector pcDNA3.1 or CD63 in the pcDNA6.F9 vector containing a FLAG and 6xHis-Tag [26] using the Turbofect method, as recommended by the manufacturer (Fermentas, St. Leon-Rot, Germany). Relative gene expression values were determined with the 2-^ΔΔCt^ method using GAPDH as housekeeping gene [27]. Transient transfection of CD63 increased CD63 mRNA expression (mean ± SEM Ct values of mRNA for CD63 and GAPDH before and after CD63 transient transfection, respectively: 23.2 ± 0.2 and 18.5 ± 0.1 for CD63 and 17.4 ± 0.1 and 19.1 ± 0.3 for GAPDH, N = 4–5, Suppl. Figure 1). Transient transfection of CD63 did not change the hOCT3 mRNA expression (Ct values of mRNA for hOCT3 before and after CD63 transient transfection, respectively: 21.8 ± 0.1 and 22.2 ± 0.2, N = 4–5, Suppl. Figure 1).

For pull-down experiments, HEK293 cells were transfected with pcDNA6.F9 CD63 or the control vector. Following transfection, the cells were washed with ice-cold phosphate-buffered saline (PBS) and subsequently collected in 0.5 ml of lysis buffer. This lysis buffer consisted of 1% Triton X-100 (v/v), 30% glycerin (v/v), a complete proteinase inhibitor cocktail (1 minipill per 10 ml; Roche, Basel, Switzerland), as well as the following mM concentrations: 150 NaCl, 50 Tris, 1 EDTA, 1 EGTA, and 10 NaVO_3_. The lysates were allowed to incubate overnight on ice at 4 °C. On the following day, the lysates were centrifuged for 1 min at 10000 g to remove insoluble cellular debris. The CD63 lysates were then immobilized on Talon Sepharose beads (Clontech Laboratories, Takara Bio, San Jose, CA, USA) and subsequently exposed to total lysates from HEK293 cells stably expressing hOCT3 for a duration of 2 h. Following this incubation, the beads were washed five times with 0.5 ml of washing buffer, which was composed of the following mM concentrations: 50 Na_3_PO_4_, 30 NaCl, and 4 imidazole. Proteins were eluted through centrifugation at 9000 g for 2 min using 50 μl of elution buffer, which contained the following mM concentrations: 50 Na_3_PO_4_, 30 NaCl, and 150 imidazole. Negative control samples were obtained by incubating lysates from hOCT3-expressing cells with beads lacking CD63. For Western blot analysis, cells were lysed as previously described and subsequently heated in sample buffer for 15 min at 60 °C. After this, the proteins were separated by SDS-PAGE and transferred onto PVDF membranes for Western blot analysis. Immunoblotting was carried out using anti-hOCT3 antibody (sc-18515, Santa Cruz Biotechnology, Santa Cruz, CA, USA) and anti-CD63 antibody (Developmental Studies Hybridoma Bank, Iowa City, IA, USA). Immunoreactive bands were visualized using horseradish peroxidase-conjugated secondary antibodies (Dako, Glostrup, Denmark) and enhanced chemiluminescence.

### Isolation of basophils

Peripheral blood mononuclear cells (PBMC) were isolated from Buffy coats (German Red Cross) of six healthy volunteers with a Ficoll density gradient assay and used for experiments within 6 h. The ethics committee of the University of Münster approved all experiments (2013-286-f-S). PBMCs were isolated using a Ficoll density gradient assay and basophil isolation was carried out using the Basophil Isolation Kit II according to the instructions of the manufacturer (Miltenyi Biotech, Bergisch Gladbach, Germany). The Basophil Isolation Kit II is based on an indirect, immunomagnetic method for the isolation of basophilic granulocytes from human PBMCs. Non-basophils, i.e. T cells, NK cells, B cells, monocytes, dendritic cells, erythroid cells, platelets, neutrophil granulocytes and eosinophil granulocytes are labelled with a cocktail of biotinylated antibodies against CD3, CD4, CD7, CD14, CD15, CD16, CD36, CD45RA, HLA-DR and CD235a. Iron-loaded antibodies are then used against the biotinylated antibodies, which subsequently ensure that the labelled cells remain in the magnetic field of the magnetic cell separation (MACS)-LS column and only unlabelled cells, the basophilic granulocytes, can pass through the LS column. The purity and vitality of the isolated basophils were measured by flow cytometry as described in detail in the supplementary materials (Suppl. Figure 2 and 3). Basophils were cultured in Gibco RPMI 1640 medium (Fisher Scientific, Schwerte, Germany) containing 100 U/ml penicillin/streptomycin (Biochrom), 2 mM L-glutamine, and 10% fetal calf serum (FCS, Biochrom). Expression of mRNA for hOCT1-3, CD63, and GAPDH was investigated by PCR analysis using the primer pairs listed in Suppl. Table 1. To induce release of histamine, human basophils (200,000 cells/ml) were incubated 24 h with 20 ng/ml human IL-3 (Miltenyi Biotech), similarly to what described in [28, 29]. Incubation with IL-3 was performed in the presence or absence of 1 mM 1-methyl-4-phenylpyridinium (MPP^+^). MPP^+^ is a substrate of OCT3 [30] and a 1 mM concentration is much higher than its IC_50_ (146 µM) for inhibition of transport of the well-known model substrate 4-(4-(dimethylamino)styryl)-N-methylpyridinium (ASP^+^) by OCT3 (Suppl. Figure 4). Histamine was measured using an ELISA test according to the instructions of the manufacturer (Histamin ELISA, IBL International, Hamburg, Germany).

Further experiments were performed in the KU812 cell line (ATCC CRL-2099). The KU812 cell line was established from the peripheral blood of a patient in blast crisis of chronic myelogenous leukemia and has some characteristics of basophilic leukocytes (high-affinity IgE receptors, the FcεRI receptors, basophilic granules, histamine production) [31, 32]. KU812 cells were cultured in RPMI 1640 medium (Sigma-Aldrich, Taufkirchen, Germany) containing 100 U/ml penicillin/streptomycin (Biochrom), and 10% FCS (Biochrom) at 37 °C and 5% CO_2_. Expression of mRNA for hOCT1-3, hOCTN1-2, MATE1 and 2 K, CD63, CD9, and GAPDH was investigated by PCR analysis using the primer pairs listed in Suppl. Table 1. For studies on histamine release by KU812 cells, cells were first counted using Scepter™ Automated Cell Counter (EMD Millipore Life Science, Merck Millipore, Darmstadt, Germany). 500.000 cells/ml were then seeded into 24-well plates and the FcεRI-receptors on KU812 cells were sensitized by incubation with 5 µg/ml IgE (Calbiochem, Merck Millipore) for 24 h at 37 °C. After this step, cells were stimulated for 20 min with 0.2 µg/ml anti-IgE (Beckmann Coulter, Brea, CA, USA) in the presence or absence of 1 mM MPP^+^ or of the non-transported inhibitor corticosterone. At the end of incubation, pellet and supernatant were separated by centrifugation at 151 g for 5 min. Histamine concentration in supernatants and in cell pellets was measured using the commercial ELISA kit (IBL International). For measurement of histamine concentration in the pellet, it was subjected to hypo-osmotic lysis with 300 µl distilled water.

Immunofluorescence analysis of KU812 cells. KU812 cells stimulated or not with IgE/anti-IgE as described above or 24 h after stimulation, were centrifuged at 151 g for 5 min and afterwards incubated for 15 min on ice to stop a possible activation of the cells. Supernatants were removed and the cell pellets were plated on microscope slides. The microscope slides were dried at room temperature and each sample was circled with a delimiting DAKO fat pen. The samples on the microscope slides were first fixed for 5 min with 4% paraformaldehyde (PFA) at room temperature, PFA was washed off 3 times for 5 min each using physiological buffered saline (PBS: 137 mM NaCl, 2.7 mM KCl, 2 mM KH_2_PO_4_, and 10 mM Na_2_HPO_4_). Then cells were permeabilized by 5 min incubation with 0.2% Triton in PBS at room temperature. After this step the slides were washed again 3 times for 5 min with PBS. Unspecific binding sites were blocked by 90 min incubation at room temperature with 10% bovine serum albumin (BSA). In the meanwhile, the primary antibodies against CD63 (H5C6, Developmental Studies Hybridoma Bank) and against hOCT3 (ab124826, Abcam, Cambridge, UK) were diluted 1:25 and 1:50, respectively, in 1% BSA. The microscope slides were incubated at 4 °C overnight with the primary antibodies. The following day the slides were first washed 3 times in 1 × PBS buffer for 5 min each and then incubated in the appropriate secondary antibodies (Goat anti-Mouse IgG (H + L) Secondary Antibody, Alexa Fluor^®^ 647 conjugate and Donkey anti-Rabbit IgG (H + L) Secondary Antibody, Alexa Fluor^®^ 488 conjugate (both from Thermo Fisher, Waltham, Massachusetts, USA)) and 4′,6-diamidino-2-phenylindole (DAPI) for 45 min. All the secondary antibodies were diluted 1:1000 in 1% BSA. Fluorescence photographs were taken with Observer Z1 with Apotome (Carl Zeiss, Göttingen, Germany) using ZEN software. Some pictures were taken as a Z-stack with 10 levels and pictures obtained by maximum intensity Z-projection are shown.

ASP^+^ uptake in hOCT3-HEK293 cells. The fluorescent organic cation ASP^+^ was employed at a concentration of 1 μM as a substrate for OCTs, in line with established protocols in our laboratory [10]. Microfluometric detection of ASP^+^ uptake was achieved using a fluorescence plate reader (Infinity M200; Tecan, Crailsheim, Germany) equipped with a monochromator system (excitation at 450 nm and emission at 590 nm), following the procedures described for cells stably transfected with OCT [10]. The emission shift between ASP^+^ in the solution and after it traversed the cell membrane enabled the monitoring of cellular ASP^+^ accumulation [10]. The dynamics of organic cation transport were measured at 37 °C, focusing on the initial rate of fluorescence increase during the first approximately 100 s [10]. The slopes of fluorescence changes were subjected to linear fitting and used as the parameter for assessing ASP^+^ uptake. Using this technique, the inhibitory potential of histamine, MPP^+^, tetrapentylammonium (TPA^+^), and corticosterone on the initial uptake of ASP^+^ was assessed (Suppl. Figure 4). A Ringer-like solution (145 mM NaCl, 1.6 mM K_2_HPO_4_, 0.4 mM KH_2_PO_4_, 5 mM glucose, 1 mM MgCl_2_, 1.3 mM Calcium D-gluconate) with pH 7.4 was used as uptake buffer. Changes of ASP^+^ uptake rate were measured in hOCT3-HEK293 cells and after transient transfection of an empty vector (EV, pcDNA6.F9) or of CD63 cloned into pcDNA6.F9 [26] via *MluI* and *NotI* sites.

Mice. C57BL/6 mice (obtained from the Animal facilities of the Medical Faculty of Münster University) designated as wild-type (WT), OCT3^−/−^, and CD63^−/−^ mice, all bred on a C57BL/6 background, aged up to 1 year and weighing between 25 and 30 g, were utilized in this study. The OCT3^−/−^ mice were generously provided by Dr. Gautron [33], while the CD63^−/−^ mice were acquired through Dr. Saftig [17]. The mouse colonies underwent rejuvenation every 10 generations to ensure the preservation of their genetic fidelity. All experiments conducted in this study were ethically approved by the North Rhine Westphalia State Environment Agency (LANUV, approval 81-02.04.2018.A387) and adhered strictly to national guidelines concerning animal welfare and protection. Focussing on OCT3^−/−^ mice, since OCT3 is involved in the transport of important neurotransmitters and their metabolites, a metabolomic analysis was performed to compare the serum metabolome of male and female WT and OCT3^−/−^ animals. Moreover, the phenotypic consequences of OCT3 genetic deletion were investigated by comparing the complete blood count and the differential of white blood cell in male WT and OCT3^−/−^mice.

Isolation of bone marrow cells. The mice were anesthetized using isoflurane and euthanized by cervical dislocation. Subsequently, tubular bones were collected in a 50 ml centrifuge tube and kept on ice during the process. A one-ml syringe filled with RPMI medium was introduced into the initial region of the bone marrow canal, and gentle pressure was applied to collect bone marrow cells (BMCs) at the lower end of the tubular bone. Subsequently, the collected solution was resuspended using a glass pipette until any clumps dissolved, resulting in a uniformly cloudy solution. The suspension was then passed through a filter two times and then subjected to centrifugation at 138 × g for 10 min. Following centrifugation, the supernatant was carefully removed, and the cellular residue was resuspended in 5–8 ml of medium. This centrifugation and resuspension process was repeated a total of three times. For the final resuspension, only 3 ml of RPMI medium was added. The entire mixture was then transferred through a filter into a new centrifuge tube. Following this, the cells were counted and subsequently diluted with RPMI-1640 medium to achieve a cell count of 250,000 cells/ml. To initiate cell culture, 1 ml of the cell suspension was pipetted into each well of a 12-well plate.

For the experiment, the BMCs were divided into two groups: one to be stimulated and the other to serve as a control. The BMCs were stimulated by 24 h incubation with 1 ng/ml recombinant murine IL-3 (Peprotech, Thermo Fisher Scientific, Waltham, MA, USA) and further addition after 24 h incubation of 5 µg/ml monoclonal anti-dinitrophenyl mouse IgE isotype (Sigma). After further 24 h incubation, stimulation was completed by 20 min incubation with 2 µg/ml dinitrophenylated BSA (DNP-BSA) (Molecular Probes, Thermo Fisher Scientific). The control group received the same volumes of sterile PBS at the same time intervals. Subsequently, the contents of each well were transferred to reaction tubes, which were centrifuged at 63 × g for 5 min at 4 °C. The resulting supernatants were carefully pipetted into new reaction tubes, while the cell pellets were resuspended in 250 µl of distilled water to induce a hypoosmotic cell lysis. After a 15-min incubation, the reaction vessels containing the cell pellets were placed in an ultrasonic bath for an additional 15 min to ensure complete cell lysis. Finally, the supernatants and cell lysates were frozen at −20 °C for further use. Supernatants and pellets of BMCs from WT and OCT3^−/−^ mice ± IL-3/IgE/anti-IgE incubation were analyzed by an untargeted metabolomic assay (s. below).

Surface biotinylation. To quantitatively assess OCT3 surface expression, surface biotinylation was performed on BMCs derived from WT and CD63⁻/⁻ mice. Cells were stimulated with IL-3, IgE, and anti-IgE antibodies to induce basophil maturation and histamine release. In selected experiments, stimulated cells were further cultured for an additional 24 h in standard medium (Minimum essential medium α, with 10% fetal bovine serum, 2% Na-Pyruvate, and 1% penicillin/streptomycin, Thermo Fisher, Rockford, IL, USA). Non-stimulated BMCs maintained in standard medium served as controls. After incubation, surface proteins were labeled with biotin, quenched, sonicated, lysed, and clarified by centrifugation, following the protocol provided with the Pierce Cell Surface Protein Isolation Kit (Thermo Scientific, Rockford, IL, USA). Biotinylated proteins were isolated by incubating lysates with immobilized NeutrAvidin gel, ensuring equal protein loading across samples. The gel was washed and incubated for 1 h with SDS-PAGE sample buffer containing 50 mM dithiothreitol. Eluates, along with whole-cell lysates, were analyzed for OCT3 expression via immunoblotting. Equal amounts of protein were loaded onto the gels, and signal intensities were quantified as percentages relative to the corresponding control samples (whole lysate or biotinylated fractions), which were set at 100%.

Sequencing/RNAseq Analysis Methods. To elucidate which pathways are regulated by stimulation of degranulation in BMCs from WT and OCT3^−/−^ mice, a RNAseq analysis was performed. BMC-RNA was isolated by GenElute^™^ Mammalian Total RNA Miniprep Kit (Merck) with additional DNase I (Merck) digestion. For measurements, only RNA with integrity number equal or higher than 7 was used. The library preparation of the total RNA was performed with NEBNext Ultra^™^ II Directional RNA Library Prep Kit for Illumina and NEBNext Poly(A) mRNA Magnetic Isolation Module. Single-end read sequencing was performed using a NextSeq 2000 System with a read length of 72 bp. The samples were demultiplexed with the Illumina DRAGEN Bio-IT Platform. Quality control was done using FastQC version 0.12.1 [34], Trimmomatic version 0.39, parameter: trimmomatic-0.39.jar SE -phred33 $input $output1 ILLUMINACLIP: NEB-SE.fa:2:30:10 LEADING:3 TRAILING:3 MINLEN:15 [35] was used for adapter and low quality end trimming as well as for general quality trimming utilizing a sliding window of 4 bp with a minimal average base quality of 15. Reads below a minimum read length of 15 bp were discarded. The resulting reads were aligned to the Ensembl GRCm39 reference genome, using HISAT2 version 2.2.1. release 6/8/2017 [36] (parameter: hisat2-q-rna-strandness RF -S ${samfile} -x ${brefindex} -U ${infilepluspath} -p ${cores}) and sorted using SAMtools version 1.16.1. parameter: samtools view -bS ${samfile} > ${bamfile} [37]. Gene based read counting was done using HTSeq version 2.0.3 [38] with the Ensembl annotation version 111. Differential expression analysis was performed using the R package DESeq2 version 1.40.1 [39]. The R package org.Mm.eg.db version 3.17.0 [40] was used to convert Ensembl IDs to mgi symbols. Plots were created using the R packages gplots version 3.1.3 [41], ggplot2 version 3.4.2 [42] and pcaExplorer version 2.26.1 [43].

**Fig. 1:**
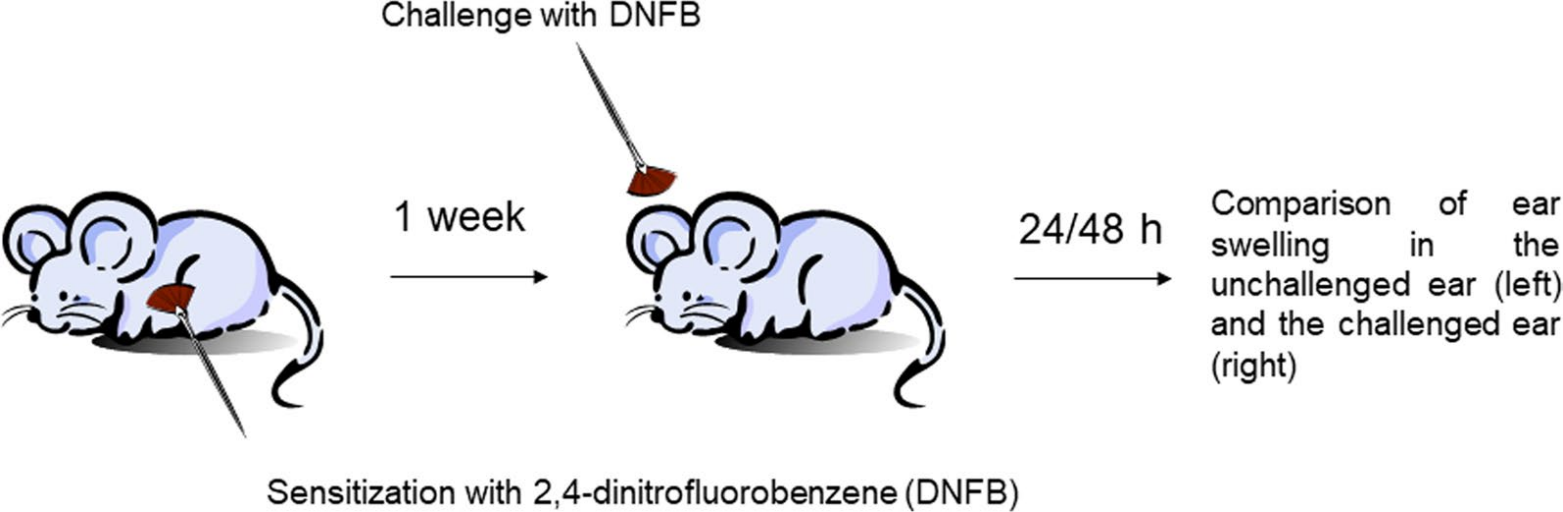
Schematic representation of contact allergy experiments with DNFB as performed in WT, CD63^−/−^, and OCT3^−/−^ mice

Contact-allergy model. Histamine is considered to participate in the formation of ear edema in the contact-allergy model with 2,4-dinitrofluorobenzene (DNFB), which is IgE-dependent and involves activation of both basophils and mast cells [44, 45]. To investigate whether OCT3 plays a role in this process, a DNFB contact allergy has been induced in WT, CD63^−/−^, and OCT3^−/−^ mice. Mice were first sensitized on the shaved belly with 100 μl 0.4% (w/v) DNFB in acetone/olive oil (4:1) and challenged 6 days later on the ear with 20 μl 0.4% DNFB (10 μl each side of the ear). Ear swelling has been compared to what observed 24 and 48 h after challenge with DNFB in the contralateral ear treated only with solvent (Fig. 1). At the end of the experiments, ears and blood were collected for histochemical analysis and determination of serum histamine, respectively.

Toluidine blue staining. Toluidine blue is a very common tissue staining for granulocytes, which especially visualizes their granules due to the presence of heparin and histamine [46, 47]. The staining was performed in mouse ear tissue cut under cryostatic conditions. Frozen (−20 °C) microscope slides were dried at room temperature for 10 min. Afterwards, they were placed in the glass basin with 1 × Toluidine Blue for 1 min. The microscope slides were then dipped three times into distilled water, four times in 70% ethanol and three times in 96% ethanol. To dehydrate the slides, they were placed for 1 min in 96% ethanol, 1 min in 100% ethanol and incubated for 10 min in Xylol before being prepared for microscopy. Microscopy analysis was performed using a Leica DM2500 microscope (Leica, Wetzlar, Germany) and the Pannoramic Viewer software (3D Histech, Budapest, Hungary).

PCR-analysis. For PCR analysis, total RNAs were isolated using the Qiagen RNeasy Minikit (Qiagen, Gilden, Germany) and reverse transcription was performed using the Superscript II system (Invitrogen, Carlsbad, CA, United States), both according to the manufacturer’s recommendations. Standard PCR was performed using specific primer pairs as listed in Suppl. Table 1. The PCR products were separated using agarose gel electrophoresis. The bands were sequenced for confirmation of amplification product identity.

### Untargeted metabolome analysis

Analysis of mouse serum: serum samples from male and female WT and OCT3^−/−^ mice in age of 1 year were isolated and immediately snap-frozen using liquid nitrogen. 100 µl of each serum sample were transferred into a 1.5 ml reaction tube, and an additional 50 µl of each sample were aliquoted in a screw cap tube to prepare pooled quality control samples. From this pooled quality control samples, three 100 µl aliquots were treated equivalent to the samples as follows. To every sample, 400 µl 80% methanol were added and mixed thoroughly. After a centrifugation step of 11000 × g for 10 min at 4 °C, supernatants of each sample were divided in two parts, each 200 µl, whereby one aliquot was used for hydrophilic interaction liquid chromatography (HILIC) for the detection of polar compounds and the other one for the reverse phased chromatography (RP) for the detection of non-polar compounds. All samples were dried using a vacuum concentrator (Labconco Corporation, Kansas City, MO, USA) at -4 °C and kept at −80 °C until analysis. Before analysis, the dried sample were reconstituted in 50 µl eluent for analysis.

Analysis of BMCs. Preparation of supernatants and pellets of BMCs: isolated BMCs from male (male were used to avoid variations due to oestrus cyclus phase) WT and OCT3^−/−^ mice in age of 1 year were used. BMCS were treated as described above and were technical replicates of pooled BMCs from at least 3 different mice/genotype and treatment. All samples were centrifuged by 150 × g for 5 min and the supernatants were collected. Cell pellets were resuspended in Dulbecco’s PBS and centrifuged again. Supernatants and pellets were snap-frozen using liquid nitrogen.

For metabolomic measurement of supernatants, 200 µl of each supernatant were pooled and split into three 500 µl aliquots for quality control. 500 µl of all samples and of the quality controls were centrifuged by 11000 × g for 10 min at 4 °C. Afterwards, 600 µl of solvent mixture (methanol/chloroform 1:1) was added. All mixtures were vortexed for 1 min and centrifuged again for 15 min using the same conditions. After this, 200 µl H_2_O was added and the mixture was vortexed for 1 min. After centrifugation, organic and aqueous phases were transferred individually into HPLC vials, whereby the aqueous phase was used for HILIC, and the organic phase for RP analysis. All samples were dried with a vacuum concentrator at −4 °C and kept at −80 °C until analysis. For analysis, all samples were reconstituted in 200 µl of the respective eluent (HILIC or RP).

For metabolomic measurement of cell lysates, 1 ml of 80% methanol was added to each pellet. After thorough vortexing, every sample was centrifuged at 11000 × g for 5 min at 4 °C. From each cell lysate 350 µl supernatant were transferred into two separate vials (one for HILIC and one for RP). For quality control, 230 µl of each sample were pooled and three aliquots of 350 µl taken from the mixture. All samples were dried with a vacuum concentrator at −4 °C and kept at −80 °C until analysis. Prior to the analysis, every sample was reconstituted in 200 µl eluent for HILIC and RP measurements.

HPLC and mass spectrometry: Chromatographic separation was achieved using an Agilent 1260 HPLC system equipped with an Acquity UPLC BEH C18, 1.7 µm, 2.1 × 100 mm column for RP mode and an Acquity UPLC BEH Amide, 1.7 µm, 2.1 × 100 mm column for HILIC mode. For both columns, associated guard columns were used to prolong column lifetime. During analysis, the column temperature was held at 40 °C and the flow rate was 250 µl/min. For both modi, a gradient elution program was used. For RP, it consisted of mobile phase A: (0.1% (v/v) formic acid in H_2_O) and mobile phase B: (0.1% (v/v) formic acid in methanol). The gradient was as follows: 0–20 min, 0.5–98% B; 20–22.5 min, 98% B, followed by a column re-calibration step using 0.5% B until 30 min. For HILIC, mobile phase A (10 mM ammonium formate in H_2_O containing 0.1% (v/v) formic acid) and mobile phase B (10 mM ammonium formate in acetonitrile/H_2_O (95:5) containing 0.1% (v/v) formic acid) was used. The gradient was applied with the setting: 0–2 min 98% B, 2–14 min from 98 to 30% B, 14–17.5 min 30% B, followed by a column re-calibration step using 98% B until 30 min.

The Q Exactive Plus mass spectrometer was operated using Xcalibur^™^ 2.13 software (Thermo Fisher Scientific) and equipped with a HESI source (Thermo Fisher Scientific). The source temperature was 320 °C. Each sample was analyzed in HILIC and RP mode and both in negative and positive polarization. For positive polarization, capillary voltage was set to 3500 V, whereas for negative polarization it was set to −3000 V. For both polarizations, sheath and aux gas were set to 30 and 20 respectively. The full scan range was 80–800 atomic mass units with a resolution of 70000 with the automatic gain control (AGC) target set at 1*10^6^ and the maximum injection time set to auto mode. The MS2 acquisition was data dependent with a resolution of 35000 with an AGC target of 5*10^4^ and the maximum injection time also set to auto, whereby the ten most abundant ions were fragmented.

Data processing: The acquired MS data sets were processed with Compound Discoverer 3.3 (Thermo Fisher Scientific). Metabolites were annotated using mzcloud and ChemSpider databases. All annotated metabolites were categorized in annotation levels (AL) 1–4. AL 1 defines metabolites identified using reference standards. AL 2 was used for annotated metabolites with MS2 and mzcloud best match > 80. AL 3 was used for annotated metabolites with MS2 and mzcloud best match 60–80. The lowest level, AL 4, was attributed to metabolites with either MS2 and mzcloud best match below 60 or only MS1. After preprocessing, metabolite lists were further filtered by excluding metabolites without or with divergent annotation and retention within the void volume. In case of supernatants and cell lysates, peak areas were normalized using cell count. Peak lists of the four modes were finally merged, whereby for metabolites detected in multiple modi, the entry with the highest abundancy was used. Pathway analyses were performed with metabolites with AL 2 and 3 by MetaboAnalyst 6.0 [48].

## Results

**Fig. 2:**
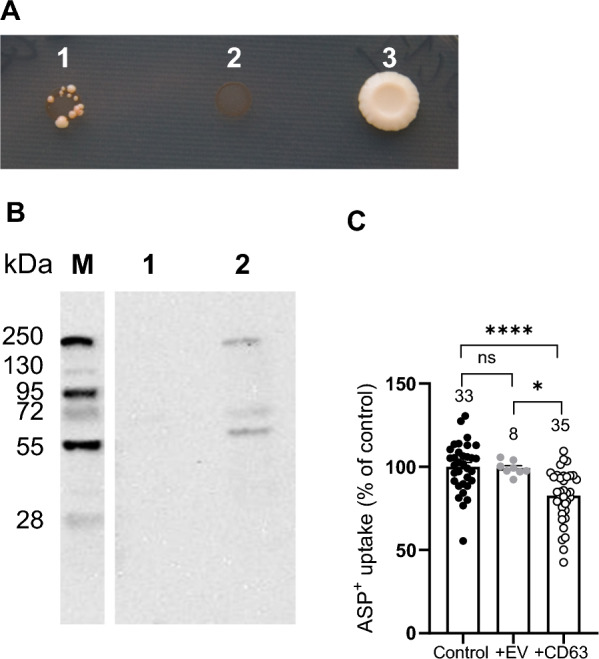
Interaction of hOCT3 and CD63 in the mbSUS (panel **A**), in pulldown assays (panel **B**), and in ASP^+^ uptake experiments (panel C). **A** Example of interaction of CD63 with hOCT3 in the yeast mating assay, which demonstrates interaction between NubG-CD63 (prey) and hOCT3-Cub (bait, colony formation in 1). Lack of interaction between hOCT3-Cub and the empty Nub vector provides a negative control (no colony formation in 2), whereas Nub-WT and hOCT3-Cub mating functions as positive control (colony formation in 3). **B** Example of pulldown assay confirming interaction between CD63 and hOCT3. Pulldown assay of CD63 linked to talon sepharose beads via 6xHIS tag incubated with hOCT3 demonstrates physical interaction (lane 2), whereas hOCT3 alone as a negative control is not detectable in Western blot analysis (lane 1). M is the molecular weight of markers (kDa). **C** Effect of transfection of empty vector (EV) or of CD63 in HEK293 cells stably expressing hOCT3 on ASP^+^ uptake. The ASP^+^ uptake was expressed as % of what measured in hOCT3-HEK293 cells (Control = 100%). Transfection with EV did not change ASP^+^ uptake compared to what measured in hOCT3-HEK293 cells (ns). Conversely, CD63 transfection significantly decreased ASP^+^ uptake compared to what measured in hOCT3-HEK293 (****p < 0.0001) and in hOCT3-HEK293-EV cell (*p = 0.0141, ANOVA test). The numbers on the top of the columns indicate the replicates that were measured in at least 3 independent experiments

The mbSUS enables the investigation of protein interactions occurring in vivo, specifically at the plasma membrane. A previous mbSUS screening revealed CD63 as a potential interaction partner for hOCT3 [17]. Here, we confirmed this result, as shown in Fig. 2A, which illustrates yeast cell growth on minimal medium after the mating of hOCT3-Cub and Nub-CD63 (colony 1), along with hOCT3-Cub with Nub-WT serving as a positive control (colony 3). Conversely, in the negative control experiments where hOCT3-Cub was paired with the empty Nub vector, no yeast cell growth was observed (colony 2). The confirmation of the hOCT3/CD63 interaction was established through a pull-down assay. This involved the binding of His-tagged CD63 to Talon beads followed by incubation with cell lysates obtained from hOCT3-expressing HEK293 cells. Subsequently, hOCT3 was detected in the eluates from the CD63-bound beads (see Fig. 2B). To validate these findings, as a negative control, beads were subjected to incubation with hOCT3 lysates lacking preincubation with His-tagged CD63. Collectively, these data confirm a physical interaction between CD63 and hOCT3. The hOCT3/CD63 interaction appears to carry functional implications for hOCT3. The introduction of CD63 expression into hOCT3-HEK293 cells resulted in a notable reduction in ASP^+^ uptake facilitated by hOCT3 (Fig. 2C). Conversely, transfection with an empty vector did not exert any discernible influence on ASP^+^ transport by hOCT3-HEK293 cells (Fig. 2C). This indicates that the presence of CD63 specifically impacts the transport function mediated by hOCT3.

**Fig. 3:**
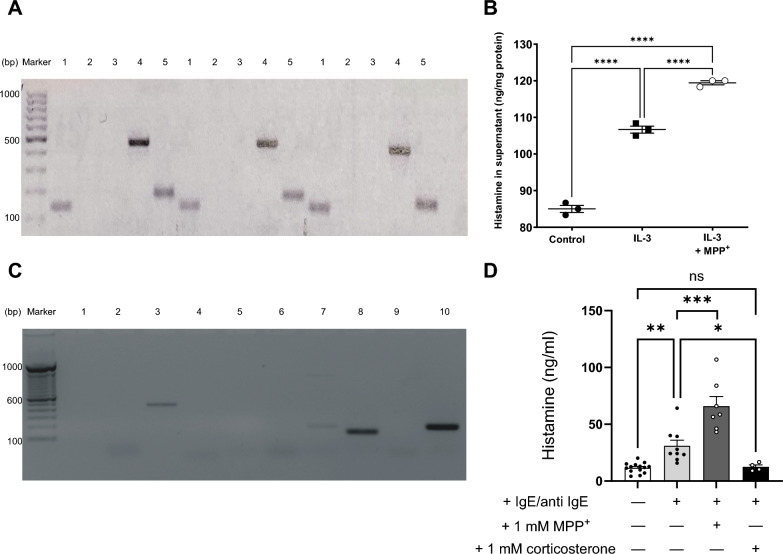
Panel **A** shows PCR analysis of mRNA expression of CD63 (lane 1), hOCT1 (lane 2), hOCT2 (lane 3), hOCT3 (lane 4), and GAPDH (lane 5) in three different preparations of fresh isolated human basophils. Panel **B** shows the effect of 24 h incubation with 20 ng/ml IL-3 in the presence or not of 1 mM MPP^+^, a known substrate of hOCT3, on the histamine release (ng/mg protein) into cell supernatant from fresh isolated human basophils. Panel **C** shows a PCR analysis of mRNA expression of hOCT1 (lane 1), hOCT2 (lane 2), hOCT3 (lane 3), MATE1 (lane 4), MATE2K (lane 5), hOCTN1 (lane 6), hOCTN2 (lane 7), CD63 (lane 8), CD9 (lane 9), and GAPDH (lane 10) in the human basophilic cell line KU812. Panel **D** shows histamine concentration in supernatants from KU812 cells with (grey columns) or without (open columns) stimulation with 5 µg/ml IgE and 0.2 µg/ml anti-IgE. The effects of the addition of 1 mM MPP.^+^ or 1 mM corticosterone to experiments with stimulation (black columns) are also shown. Histamine concentration (ng/ml) is expressed as mean ± SEM. Every single point in the figures represents the result of an independent experiment. Asterisks indicate a statistically significant difference (****p < 0.0001, ***p = 0.0001, **p = 0.0011, *p = 0.0207. ANOVA test with Dunnett’s multiple comparison test)

The investigation extended to examine the expression of CD63 and hOCT3, along with histamine release, in freshly isolated human basophils and the basophilic cell line KU812. Freshly isolated human basophils, obtained through negative selection to minimize potential stimulation of mediator release [the mean purity of basophils was 86.5 ± 4.3% (N = 6)], were found to express mRNA for CD63 and hOCT3, while lacking expression for hOCT1 and −2 (see Fig. 3A). Upon incubation with IL-3, these cells exhibited an increase in histamine release (Fig. 3B). An intriguing observation emerged during these experiments: the addition of the organic cation MPP^+^ (a known substrate of hOCT3) to the incubation medium led to a significant augmentation in histamine release (Fig. 3B). This suggests a potential trans-stimulation effect on histamine transport mediated by hOCT3. Trans-stimulation is a typical characteristic of OCT-mediated transport [49], underscoring the functional influence of hOCT3 on histamine release.

The basophilic cell line KU812 exhibited expression of hOCT3 and CD63, while lacking expression of hOCT1-2 and CD9, another tetraspanin known to interact with OCTs [50]. Among other organic cation transporters investigated (such as Multidrug and Toxin Extrusion Protein (MATE)1, MATE2K, hOCTN1, and −2), only OCTN2 showed minimal expression in KU812 (see Fig. 3C). Similar to observations with freshly isolated basophils, stimulation of histamine release in KU812 cells via IgE/anti-IgE incubation was effective. Intriguingly, the addition of MPP^+^ further amplified histamine release, while that of corticosterone, an OCT3 inhibitor, suppressed it (Fig. 3D). Similar results to those obtained with corticosterone were observed following treatment with dasatinib [51] and tetrapentylammonium [10], two non-transported inhibitors of hOCT3 (see Suppl. Figure 5).

These findings suggest that KU812 cells serve as a dependable model to explore the relationship between hOCT3, CD63, and histamine release, effectively recapitulating observations made using freshly isolated basophils.

**Fig. 4:**
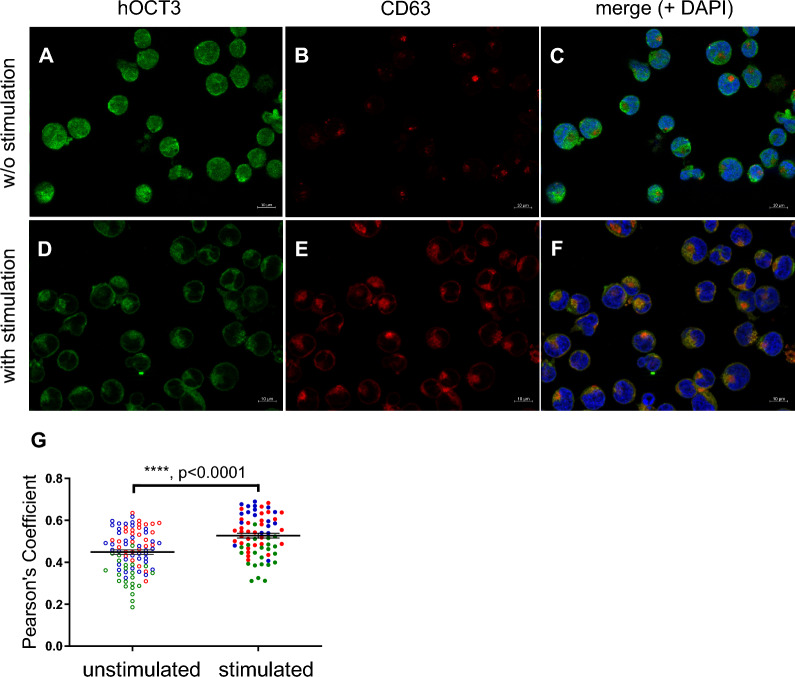
Immunofluorescence analysis of hOCT3 (green) and CD63 (red) distribution in KU812 cells without (w/o stimulation) or with stimulation (with stimulation) using 5 µg/ml IgE and 0.2 µg/ml anti-IgE (magnification 630x). Panels **A** and **B** and panels **D** and **E** show the labelling of hOCT3 and CD63 without and with stimulation, respectively. Panels **C** and **F** show the overlay of hOCT3, CD63, and DAPI labelling without and with stimulation, respectively. After stimulation, there is a clear translocation of hOCT3 and CD63 to the plasma membrane, where they co-localize. A 10 µm scale bar is also shown. Panel **G** shows Pearson’s coefficient for co-localization of hOCT3 and CD63 in the presence (stimulated) or absence (unstimulated) of histamine release stimulation with IgE/anti-IgE. Stimulation of histamine release significantly increased the hOCT3/CD63 co-localization coefficient measured using a Jacop plug-in in 88 and 70 unstimulated (open circles) and stimulated (closed circles) cells, respectively, from at least 3 independent experiments, each marked with different colors (****p < 0.0001, unpaired t-test)

**Fig. 5:**
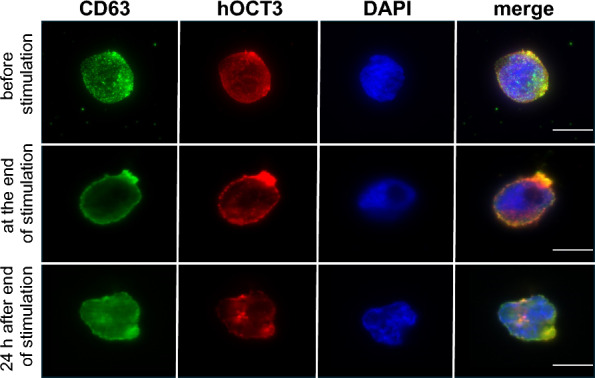
Cellular distribution of CD63 (green) and hOCT3 (red) before, at the end, and 24 h after end of incubation of KU812 cells to stimulate histamine release. The immunofluorescence analysis was conducted on stack images; histamine release was stimulated by 24 h incubation with 5 µg/ml IgE followed by 20 min incubation with 0.2 µg/ml anti-IgE (magnification 630x). Upper row represents the CD63 and hOCT3 labeling before the IgE/anti-IgE incubation, middle row immediately after IgE/anti-IgE incubation and the lower row 24 h after the end of incubation. DAPI labeling of cell nuclei (blue) and merge images are also presented. A 10 µm scale bar is included for reference in the merge picture

We conducted immunofluorescence analysis in KU812 cells to examine the expression of hOCT3 and CD63 (Fig. 4). Both proteins were identified within these cells. In resting KU812 cells, hOCT3 was observed in both the plasma membrane and intracellular compartments (Fig. 4A), while CD63 was localized mainly within intracellular vesicles (Fig. 4B). Interestingly, no overlap between hOCT3 and CD63 was detected (Fig. 4C). Upon stimulating histamine release using IgE/anti-IgE incubation, there was a distinct change: hOCT3 predominantly localized to the plasma membrane (Fig. 4D), where also an increased presence of CD63 was observed (Fig. 4E). This change resulted in the co-localization of hOCT3 and CD63 following histamine release stimulation (Fig. 4F). Utilizing the JaCoP plugin [52] to quantify the degree of co-localization between hOCT3 and CD63, a significantly stronger degree of co-localization was observed following IgE/anti-IgE incubation. This is evidenced by the higher Pearson’s coefficient noted in stimulated KU812 cells (panel 4G). Figure 5 presents additional immunofluorescence data from KU812 cells, which were analyzed before and after IgE/anti-IgE incubation to release histamine, and 24 h after end of IgE/anti-IgE incubation. These pictures were generated as a total projection from a z-stack. These images notably display the pronounced co-localization of hOCT3 and CD63 within a specific region of the plasma membrane under stimulation of histamine release, forming a distinct conical structure (Fig. 5, middle panels). The same labeling was examined 24 h after concluding the IgE/anti-IgE incubation (lower panels). Although the conical structure remains observable, a discernible trend could be observed: both hOCT3 and CD63 are gradually reverting to intracellular compartments. Interestingly, within these intracellular spaces, there is a continued co-localization of hOCT3 and CD63.

**Fig. 6:**
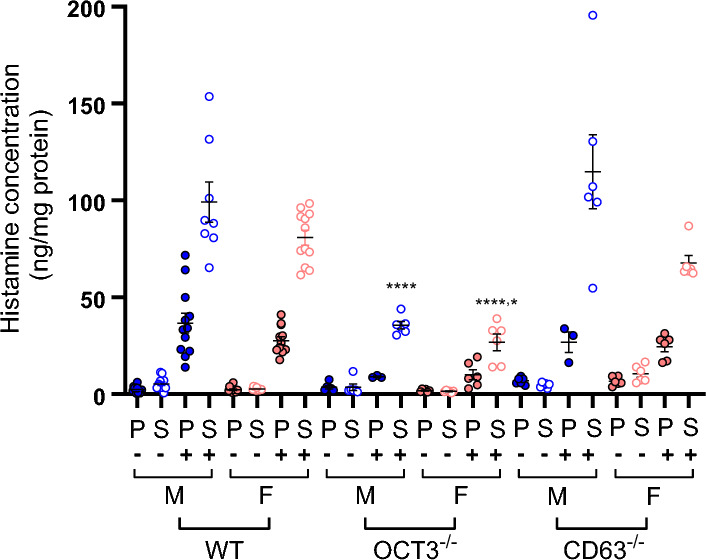
Histamine concentrations in pellets (P) and supernatants (S) of bone marrow cells (BMCs) from WT, OCT3^−/−^, and CD63^−/−^ male (M, blue closed and open symbols) and female (F, pink closed and open symbols) mice. Histamine concentration was measured by an Elisa method and reported to the protein concentration in the pellets. In some experiments, BMCs were incubated with 1 ng/ml recombinant murine IL-3 for 48 h, and for 24 h with 5 µg/ml monoclonal anti-dinitrophenyl (Maus IgE Isotyp) IgE, before adding 2 µg/ml (2,4-dinitrophenilated BSA) anti-IgE for 30 min, followed by separation of supernatants and pellets (stimulation experiments, indicated by +). Every single point indicates the results of experiments performed with BMCs isolated from one animal and the mean values with the SEM are also reported. Stimulation of BMCs significantly increased histamine content in supernatants and, only for WT mice, also in pellets (ANOVA)

The involvement of OCT3 and CD63 in the histamine release process prompted an investigation using freshly isolated bone marrow cells (BMCs) obtained from male and female WT, OCT3^−/−^, and CD63^−/−^mice. Post-isolation, BMCs were incubated with IL-3 for 48 h to initiate basophil differentiation [53]. Twenty-four hours after start of IL-3 incubation, IgE was introduced to the BMCs and after again 24 h anti-IgE was added to trigger histamine release. Comparison of histamine release between treated and untreated BMCs was conducted. In untreated BMCs no dependence from sex of histamine concentration in pellets and supernatants was observed. Treatment with IL-3 and IgE/anti-IgE notably induced a significant histamine release (p < 0.0001, ANOVA test) in BMCs obtained from both male and female WT mice (see Fig. 6). Evidently, during treatment, the BMCs exhibited increased histamine synthesis in WT mice, as indicated by the comparison of histamine content in cell pellets from male and female animals with and without stimulation (p < 0.0001, and p = 0.0228, respectively, ANOVA test). The increase of histamine concentration in pellets and supernatants from BMCs of male and female OCT3^−/−^ mice after stimulation did not reach a statistically significant difference. Compared with what was measured in OCT3^−/−^ mice, histamine release in supernatants of BMCs from male and female WT and CD63^−/−^ mice was significant higher (p < 0.0001 both for male and female supernatants from WT mice and p < 0.0001 and p = 0.0307 for supernatants from male and female CD63^−/−^ mice, respectively, Fig. 6).

The histamine release in the supernatant after stimulation of BMCs from OCT3^−/−^ mice was significantly lower (ANOVA test) than that measured in WT and CD63^−/−^ mice. **** (p < 0.0001, ANOVA) shows a statistically significant different histamine concentration in supernatants from OCT3^−/−^ compared with WT and CD63^−/−^ mice. Only for the comparison between supernatants of BMCs from female OCT3^−/−^ and female CD63^−/−^ mice the significance level was (*p = 0.0307).

**Fig. 7:**
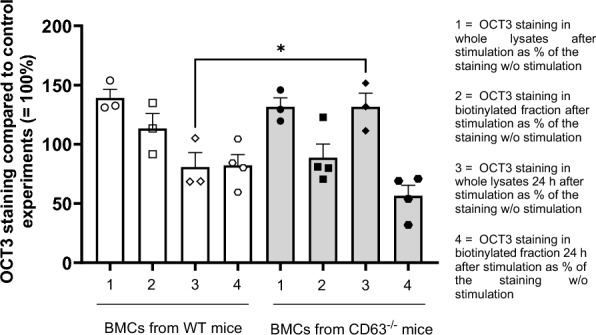
Results of the Western blot analysis of OCT3 expression in whole cell lysates and biotinylated membrane fractions from BMCs of WT and CD63⁻/⁻ mice. BMCs were stimulated with IL-3, IgE, and anti-IgE to promote basophil maturation and histamine release. In a subset of experiments, cells were further incubated for 24 h in standard culture medium following stimulation. OCT3 levels are presented as percentages relative to the corresponding control whole lysate and biotinylated fraction samples, which were set to 100%. Bars represent the mean ± SEM from 3 to 4 independent experiments, with individual data points indicated by unique symbols. Stimulation led to a marked increase in OCT3 expression in whole cell lysates from both WT and CD63⁻/⁻ BMCs. However, OCT3 levels in the biotinylated membrane fractions did not differ significantly from those of control cells in either genotype. Notably, in CD63⁻/⁻ BMCs, OCT3 expression in whole lysates remained elevated 24 h post-stimulation (p = 0.032, Welch’s t-test), in contrast to the decline observed in WT cells. In both genotypes, OCT3 levels in the biotinylated fractions were significantly reduced after 24 h

To investigate the physiological significance of the OCT3/CD63 interaction in BMCs, we compared the surface distribution of OCT3 in BMCs from WT and CD63⁻/⁻ mice under basal conditions, immediately after stimulation of basophil maturation and histamine release with IL-3, IgE, and anti-IgE, and again 24 h post-stimulation. Surface expression of OCT3 was assessed using a biotinylation assay and compared to total OCT3 levels in whole-cell lysates (Fig. 7). Stimulation led to a marked increase in OCT3 expression in whole cell lysates from both WT and CD63⁻/⁻ BMCs. However, OCT3 levels in the biotinylated membrane fractions did not differ significantly from those of control cells in either genotype. Notably, in CD63⁻/⁻ BMCs, OCT3 expression in whole lysates remained elevated 24 h post-stimulation, in contrast to the decline observed in WT cells. In both genotypes, OCT3 levels in the biotinylated fractions were significantly reduced after 24 h. The original Western blot membranes of these experiments are shown in Suppl. Figure 6.

**Fig. 8:**
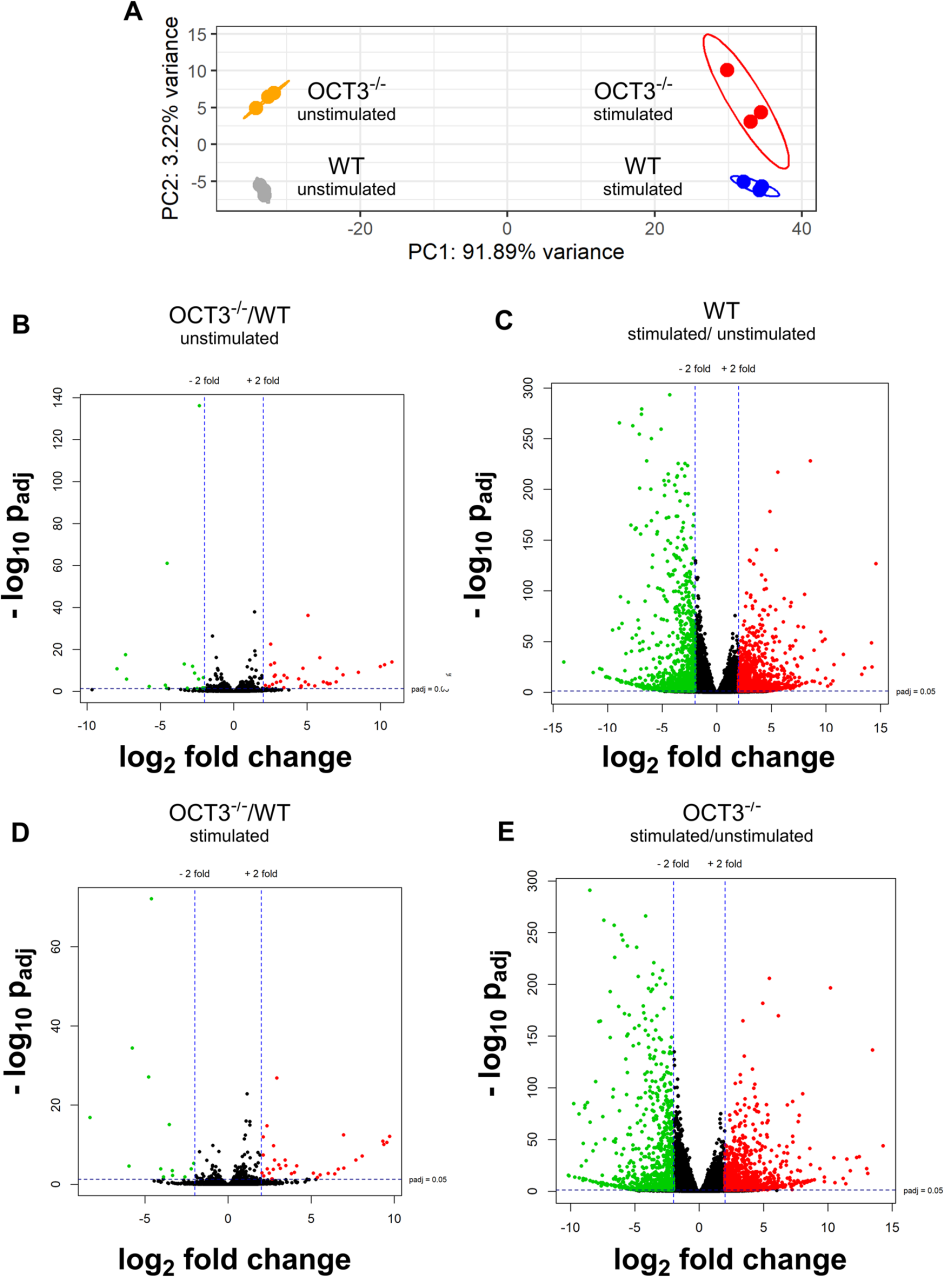
Results of RNA-Seq analysis of unstimulated and stimulated BMCs from WT, and OCT3^−/−^-mice. Panel **A** shows the principal component (PC) analysis of the mRNA-Seq data: the BMC samples cluster in 4 groups and are distinctly separated from each other. Stimulation is responsible for the biggest changes in mRNA expression. Panels **B**–**E** show Volcano plots relating the changes in expression of a mRNA (log_2_ fold change) depending on animal genotype (panels **B** and **D**) or treatment (panels **C** and **E**) to their statistical significance (−log_10_ p_adj_). Dashed lines on the x-axis represent log2 fold change thresholds of ± 2, while the dashed line on the y-axis marks the significance threshold (p = 0.05). RNAs with p < 0.05 and log2 fold change < −2 or > 2 are labeled in green for down-regulation and in red for stimulation. The genotype is responsible for a few significant changes, while treatment strongly changes mRNA-profile

Since BMCs seem to well model the process of histamine release and its dependence from OCT3, RNA-Seq analysis of stimulated and non-stimulated BMCs from male WT- and OCT3^−/−^ mice was performed. Principal component (PC) analysis of the mRNA-Seq data shows that samples from the four BMC groups cluster within each group and sample groups are separated from each other (Fig. 8). Pathways regulated by genetic deletion of OCT3 and by stimulation of basophil maturation and histamine release by incubation with IL-3, IgE, and anti-IgE (treated) in BMCs from WT-, and OCT3^−/−^-mice were analyzed using the Gene Ontology Annotations [54, 55].

**Fig. 9:**
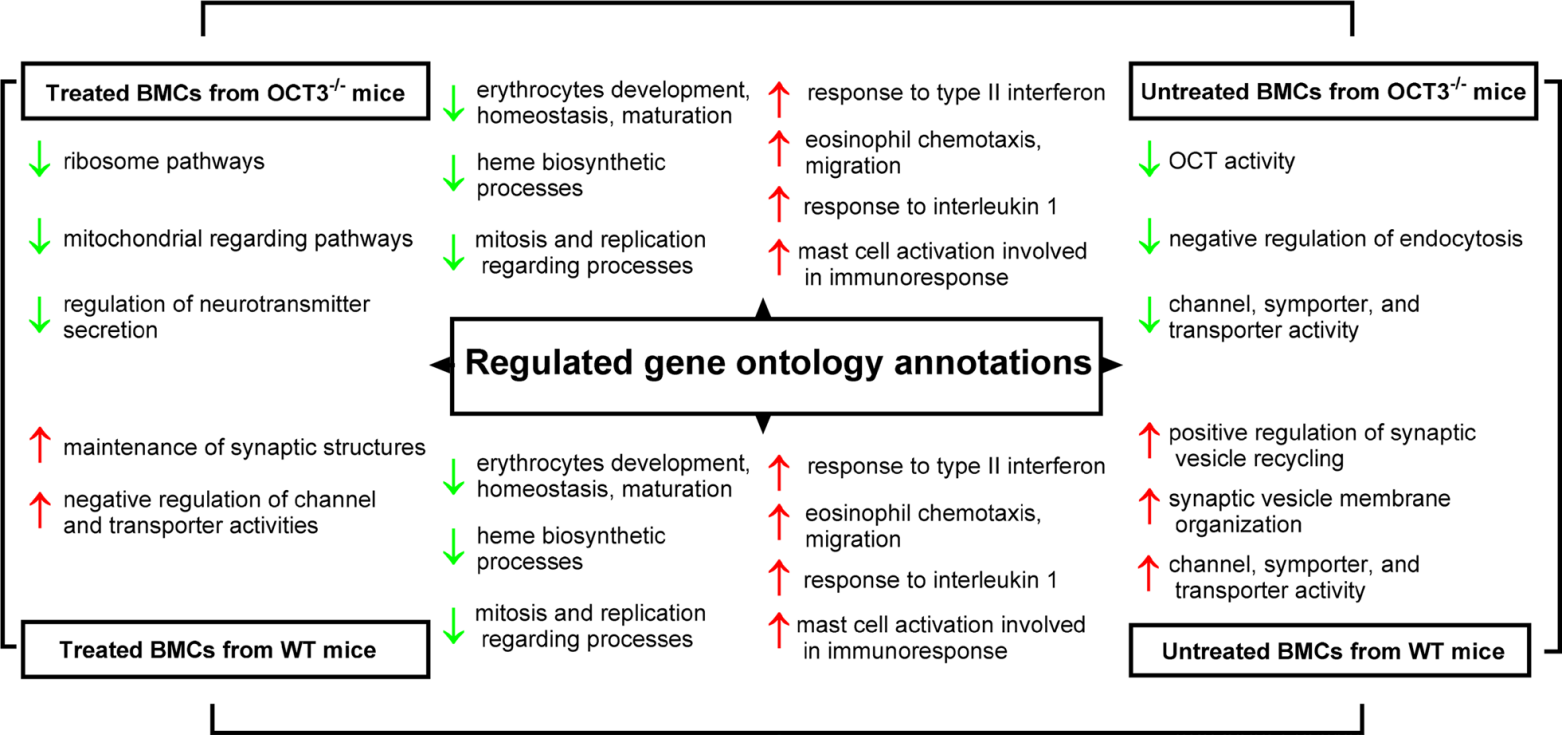
Analysis of pathways regulated by genetic deletion of OCT3 (columns) and by stimulation of basophil maturation and histamine release by incubation with IL-3, IgE, and anti-IgE (treated, rows) in BMCs from WT, and OCT3^−/−^ mice. Pathways were analyzed using the Gene Ontology Annotations [54, 55]. Green arrows with arrowheads pointing down indicate downregulation, red arrows with arrowheads pointing up indicate upregulation of a specific pathway

Treatment with IL-3, IgE, and anti-IgE produced consistent changes in mRNA expression in BMCs from both wild-type (WT) and OCT3^−/−^ mice (Fig. 9). Specifically, these treatments downregulated mRNAs associated with erythrocyte development, homeostasis, maturation, heme biosynthesis, mitosis, and DNA replication. Conversely, mRNAs linked to immune processes—including response to type II interferon, eosinophil chemotaxis and migration, interleukin-1 response, and mast cell activation—were upregulated. These changes were observed irrespective of genotype, suggesting that the treatments influenced these pathways independently of OCT3 function.

Effect of OCT3 deletion on mRNA expression in untreated BMCs: When comparing untreated BMCs from WT and OCT3^−/−^ mice, genetic deletion of OCT3 caused notable changes in baseline mRNA expression. As expected, the deletion reduced the expression of mRNAs associated with organic cation transporter (OCT) activity (Fig. 9). Additionally, mRNAs encoding proteins involved in the negative regulation of endocytosis were downregulated. Interestingly, mRNAs involved in channel, symporter, and transporter activity were also reduced. On the other hand, genetic deletion of OCT3 upregulated mRNAs associated with the positive regulation of synaptic vesicle recycling and synaptic membrane organization. Similarly, some mRNAs encoding proteins involved in channel, symporter, and transporter activity (distinct from those downregulated) were also upregulated (Fig. 9). This suggests that OCT3 deletion impairs transporter-related homeostasis, potentially compensating by enhancing alternative pathways linked to synaptic activity and membrane organization.

Effect of OCT3 deletion on mRNA expression in treated BMCs: In treated BMCs, the absence of OCT3 further modified mRNA expression patterns compared to WT mice. Specifically, the deletion of OCT3 decreased mRNA levels associated with ribosomal and mitochondrial pathways, as well as those regulating neurotransmitter secretion. Conversely, mRNAs linked to the maintenance of synaptic structures and the negative regulation of channel and transporter activities were upregulated. These findings suggest that OCT3 deletion impairs some cellular processes, such as protein synthesis and energy metabolism, while promoting compensatory changes in synaptic organization and transporter regulation. These results highlight the critical role of OCT3 in maintaining cellular homeostasis in BMCs. The downregulation of mRNAs involved in transporter activity, ribosomal function, and mitochondrial pathways in OCT3^−/−^ cells underscores the importance of OCT3 in sustaining metabolic and transport-related processes. Moreover, the upregulation of mRNAs associated with synaptic vesicle recycling and synaptic structure maintenance suggests a compensatory response to OCT3 deletion, potentially to mitigate disruptions in neurotransmitter handling. These findings also align with previous evidence linking OCT3 to neurotransmitter transport and its broader involvement in cellular signaling and metabolic pathways. The observed responses to IL-3, IgE, and anti-IgE treatment, which were consistent across WT and OCT3^−/−^ BMCs, suggest that these treatments act through mechanisms independent of OCT3. However, the amplified changes in mitochondrial, ribosomal, and synaptic pathways in treated OCT3^−/−^ cells suggest that the absence of OCT3 exacerbates cellular stress or compensatory demands in response to external stimuli. When specifically analyzing the expression of some genes as determined by mRNA-Seq analysis, it is evident that BMCs from OCT3^−/−^-mice do not express OCT3 (Suppl. Figure 7A). Since the vesicular monoamine transporters (VMAT1 and 2) and the plasma membrane monoamine transporter (PMAT) are other potential histamine transporters, we analyzed whether genetic deletion of OCT3 or treatment with IL-3, IgE, and anti-IgE altered their expression levels in BMCs. Interestingly, treatment increased VMAT1 expression (Suppl. Figure 7B) in BMCs from WT, but not in those from OCT3^−/−^ mice, suggesting that OCT3 and VMAT1 expressions are linked. Stimulation of BMCs increased VMAT2 expression independent of genotype (Suppl. Figure 7C), while PMAT expression did not change (Suppl. Figure 7D). Independently from the genotype, expression of the granulocyte marker CPA3 (Suppl. Figure 7E), the basophil marker CD41 (Suppl. Figure 7F), and the IgE receptor FCεR1a (Suppl. Figure 7H) is increased by treatment with IL-3, IgE, and anti-IgE, while the expression of the mast cell marker CD117 is decreased (Suppl. Figure 7G). These data suggest that incubation with IL-3, IgE, and anti-IgE shifts the BMCs towards a basophilic cell line, confirming the suitability of this approach in this project. Moreover, genetic deletion of OCT3 causes only small changes in specific mRNA expression. Comparing the RNA-Seq analysis in BMCs from WT and OCT3^−/−^mice, it is evident, that mainly degranulation processes are influenced by genetic deletion of OCT3. IL-4, IL-13, and Leukotriene C4s (Lct4s) secretion are not changed in BMCs from OCT3^−/−^ compared to what observed in WT mice (Suppl. Figure 8). The results of RNA-Seq analysis confirm genetic deletion of OCT3. Moreover, OCT3 deletion changed the expression of only few genes (Suppl. Figure 9), whereby the major changes derived from stimulation of basophil maturation, histamine synthesis and release by incubation with IL-3, IgE, and anti-IgE. Furthermore, OCT3 deletion seems to influence mainly the degranulation pathway of BMCs (Suppl. Figure 10). The RNA-Seq results were validated for selected key mRNAs using quantitative PCR, as shown in Suppl. Figure 11. The results of the RNA-Seq analysis are reported in Suppl. Table 3.

This hypothesis was supported by pathway analysis, which showed enrichment of eosinophil chemotaxis and granulocyte activation in response to IL-3, IgE, and anti-IgE treatment in BMCs from both WT and OCT3⁻/⁻ mice. As detailed in Suppl. Figure 12, the changes in these pathways were highly similar between the two genotypes. Therefore, we next investigated the histidine metabolism and transport pathway in stimulated BMCs. Notably, we observed a marked divergence in the expression of Amine Oxidase, Copper Containing 1 (Aoc1): transcript levels of this enzyme, which catalyzes the oxidative deamination of histamine to imidazole acetaldehyde, were significantly upregulated only in BMCs from OCT3⁻/⁻ mice (see Suppl. Figure 12). This selective upregulation suggests a potential compensatory mechanism to limit histamine signaling in the absence of OCT3. Consistent with this, increased Aoc1 activity is expected to elevate aspartate levels—a finding that was confirmed by our metabolomic analysis.

**Fig. 10:**
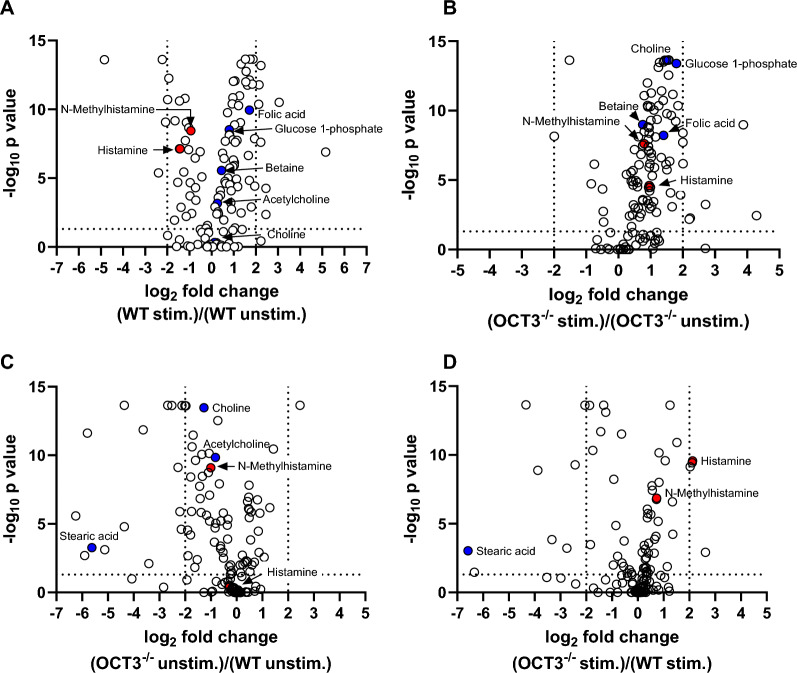
Comparison of metabolomic analysis results of BMC pellets from male WT and OCT3^−/−^ mice after stimulation with IL-3, IgE, and anti-IgE to promote basophil maturation and histamine release. Volcano plots comparing stimulated (stim.) and unstimulated (unstim.) BMCs are shown for WT mice (panel **A**) and OCT3^−/−^ mice (panel **B**). Volcano plots comparing metabolomes from unstimulated and stimulated BMCs from OCT3^−/−^ and WT mice are also shown in panel **C** and **D**, respectively. Histamine and N-methylhistamine are highlighted with red circles. Blue circles show members of the one carbon pool by folate metabolism (betaine, choline, folic acid), of glycerophospholipid metabolism (acetylcholine, choline), sucrose metabolism (glucose-1-phosphate), and biosynthesis of unsaturated fatty acids metabolism (stearic acid) pathways. Dashed lines on the x-axis represent log2 fold change thresholds of ± 2, while the dashed line on the y-axis marks the significance threshold (p = 0.05). Stimulation of WT-BMCs decreases histamine and N-methylhistamine amounts in pellets compared to unstimulated WT-BMCs (panel **A**). Conversely, after stimulation of OCT3^−/−^-BMCs histamine and N-methylhistamine amounts in pellets stayed higher than compared to unstimulated BMCs (panel **B**), suggesting that these substances do not exit the cells efficiently because of OCT3 missing. Without stimulation, OCT3^−/−^-BMCs showed few changes compared to unstimulated WT-BMCs, with histamine amount not significantly different and N-methylhistamine presents at lower concentration in pellets from unstimulated OCT3^−/−^-BMCs compared to pellets from unstimulated WT-BMCs (panel **C**). The comparison of histamine and N-methylhistamine amounts in pellets from stimulated BMCs shows that OCT3^−/−^-BMCs retain higher quantities of these substances, probably because of the missing transport out of the cells through OCT3 (panel **D**)

**Fig. 11:**
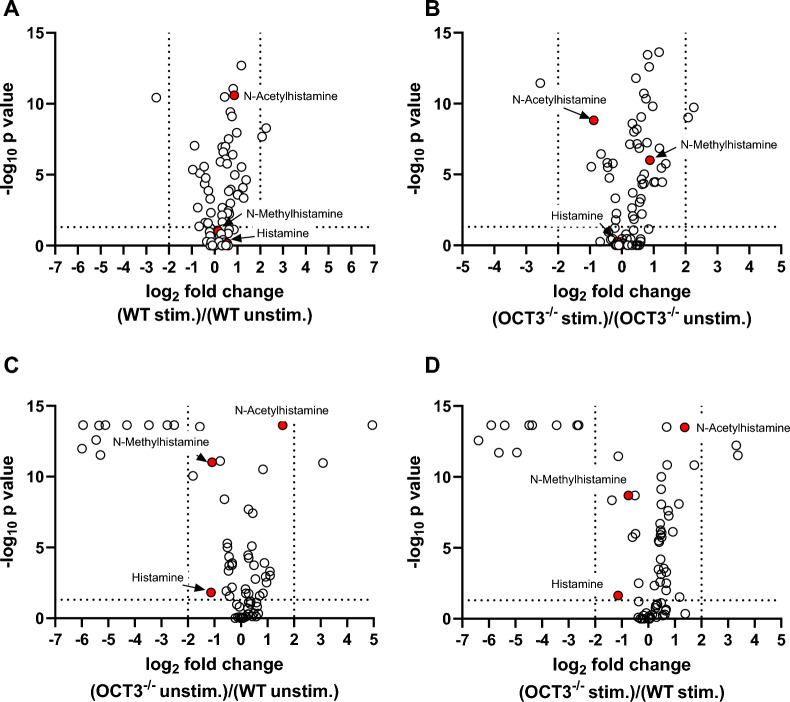
Comparison of metabolomic analysis results of BMC supernatants from male WT and OCT3^−/−^ mice after stimulation with IL-3, IgE, and anti-IgE to promote basophil maturation and histamine release. Volcano plots comparing stimulated (stim.) and unstimulated (unstim.) BMCs are shown for WT mice (panel **A**) and OCT3^−/−^ mice (panel **B**). Volcano plots comparing metabolomes from unstimulated and stimulated BMCs from OCT3^−/−^ and WT mice are also shown in panel **C** and **D**, respectively. Histamine, N-acetylhistamine, and N-methylhistamine are highlighted with red circles. Dashed lines on the x-axis represent log2 fold change thresholds of ± 2, while the dashed line on the y-axis marks the significance threshold (p = 0.05). Stimulation of WT-BMCs slightly increases only N-acetylhistamine amounts in supernatants compared to unstimulated WT-BMCs (panel **A**). Conversely, after stimulation of OCT3^−/−^-BMCs histamine abundance did not change in supernatants, while the amount of N-acetylhistamine decreased, and that of N-methylhistamine increased, compared to what measured in unstimulated BMCs (panel **B**), suggesting that N- acetylhistamine does not exit the cells efficiently because of OCT3 missing. The scarce permeability of plasma membrane to histamine and N-methylhistamine in OCT3^−/−^-BMCs is evidentiated in panels **C** and **D**, where their amount in supernatant was lower than what measured in WT-BMCs

Comparing the metabolomic profile of BMC pellets from WT and OCT3^−/−^ mice, stimulation of basophil maturation and histamine release significantly changed expression of members of histidine (histamine, 1-methylhistamine, glutamic acid), and starch and sucrose metabolism (D-maltose, glucose-1-phosphate) pathways, independently from genotype (Fig. 10). These pathways work together to ensure bone marrow cells' proper function and metabolic homeostasis: Histidine metabolism pathway is important because it is involved in histamine production and degradation. Interestingly, in BMC pellets from OCT3^−/−^ mice, a higher histamine content was found compared with those from WT animals (Fig. 10A, B), suggesting that in this case histamine cannot leave the cells efficiently. The biosynthesis of unsaturated fatty acids pathway (arachidonic acid, docohexanoic acid) is activated in stimulated BMC pellets from OCT3^−/−^ mice (Fig. 10D). This may be necessary, because the concentration of stearic acid is strongly down-regulated in BMCs from OCT3^−/−^-compared with WT-mice (Fig. 10C, D). This may be due to reduce cellular uptake of acetylcholine and choline in OCT3^−/−^ cells (Fig. 10C), which, probably as a compensation mechanism, upon stimulation of basophil maturation and mediators release, increase the activity of the one carbon pool by folate metabolic pathway (betaine, choline, folic acid). Lipid mediators are known to influence granulocyte functions and allergic responses [56]. The metabolomic changes described here are very similar to those previously shown for IgE-mediated fatal anaphylactic shock in rats [57]. Analyzing the metabolomic changes in supernatants from stimulated and unstimulated BMCs from WT and OCT3^−/−^ mice, it is evident that histidine metabolism is significantly down-regulated both in unstimulated and stimulated BMCs from OCT3^−/−^ mice in comparison to BMCs from the WT mice (Fig. 11A–D), confirming the importance of OCT3 for histamine signaling. Interestingly, in supernatants of BMCs from both genotypes, N-acetylhistamine appears as the major histamine derivative. This can be explained admitting that N-acetylhistamine formed in the cells from histamine is completely secreted into the supernatants or that histamine is metabolized in the extracellular space to N-acetylhistamine.

The findings obtained from metabolomic analysis of BMCs from OCT3-deficient mice provide valuable insights into the physiological function of OCT3 in these cells. A summary of these findings is shown in Table 1, where metabolites, whose amount is significantly changed (p < 0.05) uniquely in supernatants and pellets from WT- and OCT3^−/−^ BMCs are shown. After stimulation, WT BMCs cells exhibit lower levels of oxidized lipid species. Specifically, oxidized or epoxy derivatives such as 9-HpODE, tri-hydroxy-octadecenoic acid, and EpETE are present at reduced levels in WT-BMC pellets. In the supernatants from WT BMCs, the reduced levels of dipalmitoylphosphatidylcholine and isoleucine suggest that these cells may either release fewer phospholipid fragments or process amino acids differently compared to OCT3-deficient cells. In contrast, the increased taurochenodeoxycholic acid in supernatants from WT samples might indicate unique interactions with bile acid pathways or a specific influence from the microbiota. The lower concentrations of uracil and isocytosine in WT cell pellets imply that there may be less RNA turnover or fewer catabolic by-products of nucleotide degradation in these cells. This contrasts with OCT3^−/−^ BMCs, which appear to undergo higher nucleic acid turnover or stress-related breakdown. These findings indicate that WT BMCs produce fewer oxidative lipid by-products and exhibit distinct patterns of metabolite release compared to OCT3-deficient cells. Specifically, the increased levels of metabolites such as taurine, acetyl-L-carnitine, choline, and leucylproline in the pellets of OCT3^−/−^ BMCs imply that these compounds might serve as substrates for OCT3. In the absence of OCT3, the cells seem to retain higher amounts of certain lipids, amino acids, and choline derivatives, suggesting an intracellular metabolic reprogramming that could influence granulocyte maturation and functionality. Furthermore, the metabolite profile in the supernatants, including elevated levels of N-methylhistamine, methylated nucleosides, and acylcarnitines, indicates that OCT3^−/−^ BMCs secrete a distinct set of metabolites. These alterations are likely associated with release of epigenetic by-products, and modifications in histamine metabolism, reflecting an augmented mediator release. Additionally, shifts in the endocannabinoid (2-arachidonoyl glycerol) and eicosanoid (15(S)-HpETE) pathways, together with changes in histamine metabolism, underscore the possibility of modified inflammatory responses in OCT3-deficient granulocytes. This supports the idea that the absence of OCT3 alters lipid oxidation and nucleotide turnover.

**Table 1:** Supernatant and pellet endogenous metabolites, which are uniquely significantly (p < 0.05) regulated by IL-3, IgE, and anti IgE incubation of blood marrow cells from WT- and OCT3^−/−^ mice

Unique metabolites	Log_2_ fold changes
BMC from WT mice	
Supernatants	
DL-Dipalmitoylphosphatidylcholine	−0.46
L-Isoleucine	−0.44
Taurochenodeoxycholic acid	1.06
Pellets	
(15Z)-9,12,13-Trihydroxy-15-octadecenoic acid	−2.40
Isocytosine	−1.93
9-Hydroperoxyoctadeca-10,12-dienoate (9-HpODE)	−1.53
Uracil	−1.38
17,18 EpETE	−1.15
BMC from OCT3^−/−^ mice	
Supernatants	
Isocytosine	0.45
N-Methylhistamine	0.50
5-Methylcytosine	0.57
Nicotinamide	0.67
N-Methyladenosine	0.69
Hexanoylcarnitine	0.89
Pellets	
Taurine	0.44
Acetyl-L-Carnitine	0.62
Sphingosine	0.64
Palmitic acid	0.76
L-Tyrosine	0.90
DL-Lysine	1.18
Choline	1.50
3’-AMP	1.56
Leucylproline	1.62
15(S)-HpETE	2.20
2-Arachidonoyl glycerol	2.71

**Fig. 12:**
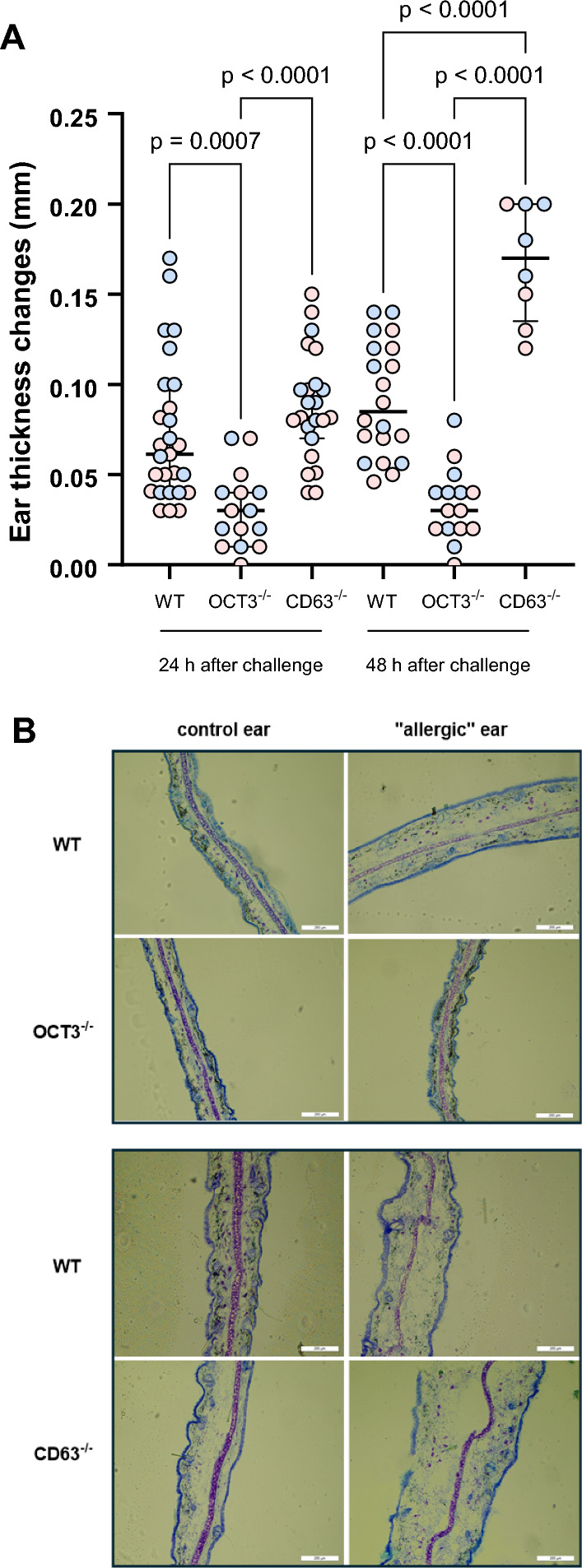
Panel **A** Ear swelling measured as ear thickness changes in WT, OCT3^−/−^, and CD63^−/−^ mice 24 and 48 h after challenge with DNFB compared with the contralateral unchallenged ear. In WT and in CD63^−/−^ mice a significantly higher ear swelling than in OCT3^−/−^ mice was measured already 24 h after challenge. Every point represents an animal. Indicated are the median values together with the interquartile ranges as well as the significance levels of statistically significant differences (ANOVA). Pink and blue points indicated results obtained with female and male animals, respectively. Panel **B** Toluidine blue staining of mouse ear pinnae from WT, OCT3^−/−^, and CD63^−/−^ mice 48 h after challenge with DFNB (“allergic” ear) compared with the contralateral unchallenged ear (“control” ear). In WT and CD63^−/−^ mice a robust ear swelling is visible, while it is not evident in OCT3^−/−^ animals. Scale bar = 200 µm

To explore the potential pathophysiological impact of the reduced histamine release seen in BMCs from OCT3^−/−^-mice, the extent of ear swelling was compared between WT, CD63^−/−^, and OCT3^−/−^ mice using a contact dermatitis model. In this particular model of irritant dermatitis, granulocytes, encompassing both basophils and mast cells, hold significant importance [58, 59]. Additionally, it appears that histamine release by mast cells mediates the initial phases of ear swelling in DNFB-induced contact dermatitis [60]. In this work, following challenges with DNFB at both 24 and 48 h in male and female animals, a statistically significant reduction in ear swelling was observed in OCT3^−/−^ mice compared to the swelling observed in WT and CD63^−/−^ mice (Fig. 12). Figure 12B shows a histological analysis of the DNFB-induced inflammation in mouse ear tissues 48 h after challenge (“allergic” ear) compared with the contralateral unchallenged ear (control ear). In WT and CD63^−/−^ mice a robust ear swelling is visible, while it is not evident in OCT3^−/−^ animals.

However, when analyzing the results according to the sex of the animals, it is evident that one day after challenge with DNFB, male WT animals show a significant higher ear swelling than female mice (Table 2). Only at this time-point, ear swelling in female WT mice is not different from what measured in OCT3^−/−^mice. Since OCT3 seems to have a pivotal importance in determining the grade of allergic reaction in the DNFB contact dermatitis model, the complete blood count and the white blood cell differential of male WT and OCT3^−/−^ mice were compared. Male mice were investigated since they showed a robust reaction to challenge with DNFB. No significant difference in dependence from genotype was found (s. Suppl. Materials Table 2).

**Table 2:** Ear thickness changes in male (M) and female (F) animals (in mm, mean ± SEM, with the number of animals N in every group) measured 1 and 2 days after challenge in the contact dermatitis assay

Days after challenge	Ear thickness changes in male (M) and female (F) animals (in mm, mean ± SEM, with the number of animals N in every group)					
	WT M	WT F	OCT3^−/−^ M	OCT3^−/−^ F	CD63^−/−^ M	CD63^−/−^ F
+ 1	0.10 ± 0.01 N = 12	0.05 ± 0.01** N = 11	0.03 ± 0.01 N = 7	0.03 ± 0.01 N = 8	0.09 ± 0.01 N = 9	0.09 ± 0.01 N = 14
+ 2	0.10 ± 0.01 N = 8	0.08 ± 0.01 N = 12	0.04 ± 0.01 N = 7	0.03 ± 0.01 N = 8	0.19 ± 0.01 N = 4	0.15 ± 0.02 N = 4

Only 1 day after challenge a significant difference in ear swelling was measured between male and female mice

Significantly different from WT M + 1 day after challenge, ** p = 0.004, t-test

**Fig. 13:**
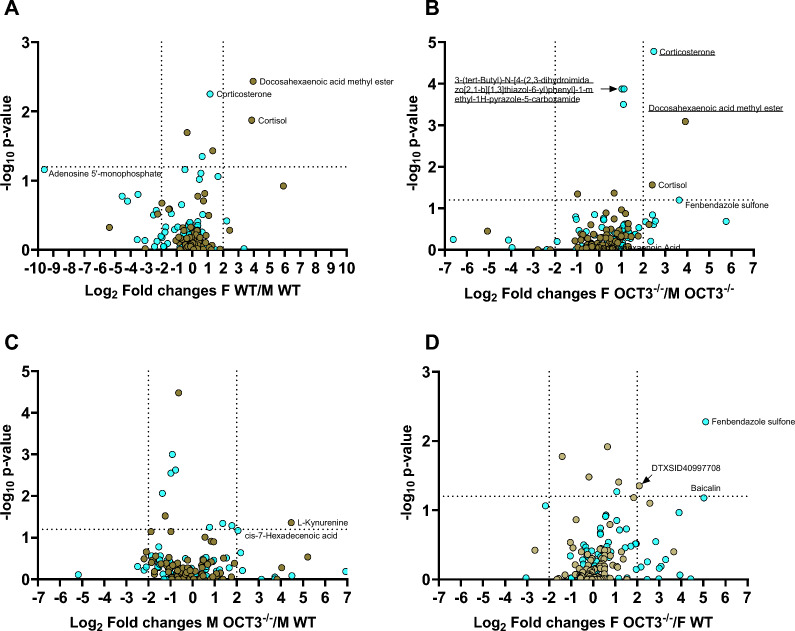
Volcano plots from serum metabolomic analysis of male (M) and female (F) WT and OCT3^−/−^ mice. The metabolomic profiles revealed minimal differences between groups. Metabolites annotated at level 2 are highlighted in light blue, and those at level 3 in brown. Dashed lines on the x-axis represent log2 fold change thresholds of ± 2, while the dashed line on the y-axis marks the significance threshold (p = 0.05). Metabolites with p < 0.05 and log2 fold change < −2 or > 2 are labeled, alongside a few additional metabolites of interest that do not meet these criteria. Panels **A** and **B** show that female mice of both WT and OCT3^−/−^ genotypes exhibit higher serum concentrations of corticosterone and cortisol compared to males. The genotype had no significant impact on the serum metabolome of male mice (panel **C**), whereas small differences were observed in females (panel **D**). Underlined names show substances with significantly different p_adj_ values (p < 0.05) in the statistical comparison

Moreover, we analyzed whether the genetic deletion of OCT3 alters the concentration of serum metabolites that may play a role in allergic reactions using an untargeted metabolomic analysis of blood serum from WT and OCT3^−/−^ mice. Genetic deletion of OCT3 resulted in few differences in the serum metabolome, and the PCA analysis did not show a clear difference between the groups (Suppl. Figure 13). The most striking differences were observed when comparing female and male mice regardless of genotype. Interestingly, female mice had higher serum corticosterone and cortisol concentrations than males (Fig. 13A, B). Corticosterone and cortisol are key hormones within the hypothalamic–pituitary–adrenal (HPA) axis, a critical neuroendocrine system that regulates hormonal responses to both internal and external stressors in an organism's environment. Interestingly, adult female rodents exhibit a significantly stronger acute HPA axis response to stressors compared to their male counterparts [61]. Corticosterone is the major corticosteroid hormone involved in the regulation of stress responses in rodents. Recently, Nguyen et al. [62] reported that female mice display higher serum corticosterone concentration than male animals. In female mice, no significant changes in corticosterone concentrations were observed throughout the estrous cycle [63]. Corticosterone is known to directly and acutely inhibit OCT3 activity (for a review s. [64]). Moreover, female mice showed higher serum level of docosahexaenoic acid methyl ester, a fatty acid ester that is highly abundant in neuronal membranes and, only in OCT3^−/−^ animals of fenbendazole sulfone, a broad spectrum benzimidazole anthelmintic (Fig. 13A, B). Only female WT mice showed a lower serum concentration of AMP compared to male counterpart (Fig. 13A). The significance of this is not clear. Comparing the mouse serum metabolome in dependence from the genotype, male OCT3^−/−^ mice show higher serum concentration of L-kynurenine, and of 7-hexadecenoic acid than male WT mice (Fig. 13C). Interestingly, the kynurenine (KYN) pathway of tryptophan (TRP) metabolism acts as a tolerogenic, immunosuppressive enzyme to attenuate allergic responses by the induction of the KYN- indoleamine 2,3 dioxygenase (IDO) pathway, resultant depletion of TRP, and elevation in KYN metabolites [65]. 7-hexadecenoic acid is a candidate marker for foamy monocyte formation [66]. Serum metabolites from female OCT3^−/−^ and WT mice displayed differences in the concentration of some exogenous substance, which are taken in via food, such as the flavonoids 4H-1-Benzopyran-4-one, 6-β-D-glucopyranosyl-2,3-dihydro-5,7-dihydroxy-2-(4-hydroxyphenyl) and baicalin, and anthelmintic fenbendazole sulfone (Fig. 13D), suggesting a possible role of OCT3 for excretion of these substances.

## Discussion

The OCT3 is a plasma membrane transporter, which is widely distributed across different tissues, exhibiting a nearly ubiquitous presence. It is distinguished by its low-affinity interaction with numerous crucial neurotransmitters, such as serotonin, dopamine, and histamine [10, 30]. For example, the OCT3 appears to serve a significant role as an additional and supportive transporter for serotonin reuptake within serotoninergic synapses [11, 12, 67]. Due to its specific functional attributes, OCT3 represents one possible molecular counterpart of the uptake2 system [30, 68]. This system orchestrates the Na^+^- and Cl^−^-independent uptake of several steroid-sensitive monoamines across multiple extraneuronal tissues [30, 68]. In order to gain new insights into the function of hOCT3, the interaction of OCT3 with the tetraspanin CD63 was investigated in the present study. The importance of protein–protein interactions stems from their role in revealing the operational mechanisms within biological systems. Grasping these interactions is essential for understanding the functionalities and dynamics of proteins. It facilitates the anticipation of a protein's participation in diverse biological processes through the 'guilty by association' principle. This principle suggests that when proteins with unknown functions associate with those of known functions, valuable insights emerge. Moreover, understanding protein–protein interactions enables the construction of intricate interaction networks, enriching our understanding of biological pathways with detailed information. Previously, a direct interaction between hOCT1-3 with CD63 had been demonstrated [17]. In this study, we successfully validated the hOCT3/CD63 interaction using the mbSUS method and confirmed it through pull-down analysis. This interaction appears to hold potential functional implications, at least in vitro, since the introduction of CD63 via transfection into HEK cells that stably express hOCT3 led to a notable reduction in hOCT3 functionality. CD63 has previously exhibited associations with membrane proteins. Its direct interaction has been documented in the endocytosis of the gastric H^+^/K^+^-ATPase [69] and in the recycling process of hOCT2 from endosomes back to the basolateral membrane [17].

After the activation triggered by IgE cross-linking of FcɛRI receptors, the heightened presence of CD63 on the plasma membrane serves as a recognized marker indicating activation in both basophils and mast cells (MCs) [22–24]. Activated basophils and MCs secrete histamine, cytokines, chemokines, heparin, and proteases, which cause smooth muscle constriction, increased vascular permeability and inflammatory cell recruitment [70]. Histamine is a prominent contributor to allergic disease and is the primary mediator of anaphylactic shock, an immediate and potentially fatal systemic reaction most commonly caused by IgE-mediated degranulation of MCs and basophils, whose signs and symptoms are caused by histamine binding to its receptors [25]. Histamine release from MCs and basophils is a regulated process resulting in a rash and high increase of histamine concentration in the extracellular space. Mechanisms underlying the clearance of histamine are still unknown, as outlined above. Since OCT3 is expressed in MCs [71] and basophils [15] and activated basophils and MCs secrete high concentrations of histamine, the discovery of a direct interaction between hOCT3 and CD63 prompted us to examine whether there can be a link between CD63 and hOCT3 function. Histamine is a low-affinity substrate of OCT3, and for this reason represents the possible link for understanding the significance of OCT3/CD63 interaction, suggesting a not yet identified role of OCT3 for controlling histamine extracellular concentration and clearance. The OCT3/CD63 interaction may provide an autocrine control loop for histamine secretion in basophils and MCs. In this work, expression of hOCT3 and CD63 was confirmed in freshly isolated human basophils, where hOCT1 and hOCT2 were not detected. Freshly obtained human basophils exhibited the capability to discharge histamine when prompted by IL-3. This cytokine, IL-3, stands as the most influential growth and activation agent for basophils in both humans and mice [72]. Fascinatingly, the inclusion of MPP^+^, a substrate of hOCT3, in the incubation solution resulted in an additional augmentation of histamine release facilitated by IL-3. This observation implies that hOCT3 within basophils contributes to a portion of histamine release and that MPP^+^ present in the extracellular fluid has the ability to trans-stimulate hOCT3 transport from basophils into the extracellular compartment. It is worth noting that trans-stimulation is a recognized property commonly associated with transport mediated by OCTs [49]. Interestingly, the administration of high doses of morphine through intravenous infusion consistently leads to elevated levels of plasma histamine, frequently culminating in shock [70]. Morphine is recognized as a substrate of OCT [73], and the trans-stimulation of histamine transport within granulocytes could potentially be the underlying cause of these effects associated with morphine.

Additional investigations were conducted utilizing the KU812 basophilic cell line. This cell line was chosen as it mirrors some for this study important characteristics of freshly isolated human basophils. These properties include the expression of hOCT3 and CD63, as well as the release of histamine upon stimulation with IgE/anti-IgE. Notably, this histamine release can be amplified through further incubation with MPP^+^. On the contrary, incubation with corticosterone or TPA^+^, poorly transported inhibitors of hOCT3 [74], did not elicit any further stimulation of histamine release. Immunofluorescence analysis reveals that in quiescent KU812 cells, CD63 predominantly localizes within intracellular granules, while hOCT3 exhibits a dispersed distribution throughout the cell. Upon incubation with IgE/anti-IgE, KU812 cells demonstrate a translocation of CD63 and hOCT3 to the plasma membrane, notably showcasing a distinct co-localization of both proteins. This co-localization is particularly evident within a distinct conical protrusion of the cells, distinctly visible in projected cell volume images. Following histamine release, a portion of the hOCT3 and CD63 proteins relocates to intracellular compartments, where they exhibit further co-localization. The findings indicate that the interaction between hOCT3 and CD63 primarily occurs during histamine release. Subsequent to this event, as CD63 relocates to intracellular compartments, it appears to carry along some hOCT3. In the absence of CD63, OCT3 will stay in the plasma membrane further releasing histamine and probably increasing severity of allergic reactions. This scenario seems to be confirmed by the experiments measuring histamine release of BMCs from CD63^−/−^ mice and from contact allergy experiments with CD63^−/−^ mice. Surface biotinylation experiments with BMCs from WT and CD63^−/−^ mice (Fig. 7) showed that stimulation led to a marked increase in OCT3 expression in whole cell lysates from both WT and CD63⁻/⁻ BMCs. However, OCT3 levels in the biotinylated membrane fractions did not differ significantly from those of control cells in either genotype. Notably, in CD63⁻/⁻ BMCs, OCT3 expression in whole lysates remained elevated 24 h post-stimulation, in contrast to the decline observed in WT cells. In both genotypes, OCT3 levels in the biotinylated fractions were significantly reduced after 24 h.

These findings strengthen the idea that the interaction between CD63 and OCT3 may play a role in directing OCT3 toward lysosomal degradation. In the absence of CD63, OCT3 appears to internalize from the plasma membrane but is not efficiently degraded, instead accumulating within the cell. Notably, CD63 is known to traffic between various intracellular compartments—most prominently the lysosomes—and the plasma membrane, supporting its potential involvement in this pathway [21].

The potential role of OCT3 in the release of histamine by murine basophils was suggested in a study by Schneider et al. [15]. However, due to the inherent challenges in studying basophils, which constitute only a small fraction (about 0.5–1%) of circulating leukocytes, the precise involvement of OCT3 in histamine action remains incompletely understood. Using BMCs isolated from WT, CD63^−/−^, or OCT3^−/−^ mice, this study demonstrated the critical role of OCT3 in facilitating efficient histamine release. RNA-Seq analysis confirmed that the BMC stimulation method employed here effectively differentiated the cells into basophils and highlighted the importance of OCT3 for their secretory function. Both RNA-Seq and untargeted metabolomic analyses revealed that the global deletion of OCT3 resulted in minimal phenotypic changes, with alterations primarily focused on histamine release. A pronounced divergent fold change in expression was observed for Amine oxidase copper containing 1 (Aoc1): the transcript levels of this enzyme, responsible for catalyzing the oxidative deamination of histamine to imidazole acetaldehyde, were significantly upregulated following stimulation of bone marrow cells (BMCs) derived from OCT3^−/−^ mice (Suppl. Figure 12). This upregulation suggests a potential compensatory mechanism that mitigates histamine signaling in the absence of OCT3.

Interestingly, contact allergy testing showed that female WT mice developed sign of contact dermatitis with a delay compared with male animals. This may be due to the higher serum concentration of corticosterone in female mice compared to male animals. Corticosterone may inhibit OCT3 and slow OCT3-mediated histamine release, resulting in a delayed development of allergic reaction. Indeed, it has been already shown that female mice exhibit higher serum corticosterone levels than males due to neuroendocrine, hormonal, and metabolic factors. Females display weaker negative feedback of the hypothalamic–pituitary–adrenal (HPA) axis, contributing to elevated basal corticosterone [75]. Elevated corticosteroid-binding globulin (CBG) further modulates its bioavailability. Although acute stress raises corticosterone similarly in both sexes (~ 350 to 450 ng/ml), females maintain higher baseline levels (41.6 vs 20.1 ng/ml) [76, 77]. Ovariectomy dampens stress-induced corticosterone in females, but sex hormone replacement does not restore it, implying a role for non-steroidal ovarian factors [76]. In males, castration heightens HPA reactivity, while androgens suppress it [75]. Finally, females exhibit distinct adipose profiles (e.g., higher adiponectin, hyperplastic fat), which may indirectly affect HPA regulation via insulin-leptin pathways [75]. Together, these mechanisms underlie the persistently higher corticosterone levels in females, despite similar anxiety-related behaviors. This sex-specific regulation appears evolutionarily conserved and may reflect divergent adaptive strategies in rodents [77–79]. In humans, allergy sensitivity is greater in males than females primarily during childhood and for most common allergens, with the exception of fungal allergens, where females may be more sensitive. This pattern reverses in adulthood, with females generally exhibiting higher allergy prevalence and sensitivity [80–82]. In humans the main stress hormone is cortisol. Free cortisol concentrations are higher in males than females during adulthood, particularly before age 50, due to sex-specific differences in basal secretion and ACTH responsiveness. This gap diminishes after age 50, as aging elevates cortisol uniformly. In children, patterns reverse, with girls showing higher stimulated cortisol. These dynamics underscore the interplay between sex hormones, aging, and hypothalamic–pituitary–adrenal axis regulation [83, 84].

The incubation conditions used with freshly isolated mouse BMCs were suitable to increase both histamine biosynthesis and release, similar to what has been observed previously [85]. While genetic deletion of CD63 did not alter histamine biosynthesis and release, the absence of OCT3 resulted in a significant decrease in these parameters. However, when histamine release as % of total cellular histamine content (histamine in pellet + supernatants) was compared, no difference between genotypes was found (histamine release as % of total cellular histamine 78 ± 4.9%, 77 ± 8.4%, and 78 ± 12.5% in stimulated BMCs from WT-, CD63^−/−^-, and OCT3^−/−^-mice, respectively), suggesting a role for OCT3 in histamine biosynthesis. The pathophysiological implications of this observation are evidentiated by the low allergic reaction measured in OCT3^−/−^ mice compared to what observed in WT mice in the contact dermatitis model. Histological pictures confirm a stronger ear swelling in WT-mice than in OCT3^−/−^-mice. These results reveal a previously unknown role of OCT3 in determining the extent of the allergic reaction and make it a potential candidate for the development of preventive measures against excessive histamine release.

Effective drug treatment is often compromised by hypersensitivity reactions. Major chemotherapeutic drugs such as cisplatin and oxaliplatin are known to cause early-onset IgE-mast cell and basophil mediated hypersensitivity reactions with an incidence of 5–20% [86]. Conversely, other drugs, such as the tyrosine kinase inhibitors dasatinib and masitinib are known to inhibit FCεR1a-mediated activation of basophils [87] and of MCs [88], respectively. OCT substrates stimulate histamine release, while OCT inhibitors can decrease it. It has been demonstrated that cisplatin, and oxaliplatin are substrates of OCT [51, 89–92], while dasatinib is a negative regulator of OCT activity [51]. Therefore, cisplatin and oxaliplatin may trans-stimulate histamine release from sensitized granulocytes via OCT, producing a hypersensitivity reaction, whereas dasatinib may decrease it by inhibiting OCT activity. Inhibition of OCT3 may be an option to reduce hypersensitivity reactions induced by drug treatment.

## Conclusions

In conclusion, OCT3 appears to play a significant role in facilitating histamine release from granulocytes. Following granulocyte activation, OCT3 interacts with CD63, triggering its intracellular recycling. Targeting OCT3 in granulocytes could represent a potential strategy for mitigating the severity of histamine-mediated allergic responses.

## Supplementary Information

Supplementary material 1.

Supplementary material 2.

Supplementary material 3.

Supplementary material 4.

Supplementary material 5.

## Data Availability

The results of the RNA-Seq analysis are made available as supplementary materials to this submission and consist of an Excel spreadsheet showing the Ensembl Gene ID, the Group (mean of normalized counts), the baseMean, (mean of normalized counts for all samples), the log2FoldChange (log2 fold change (MLE)), the lfc standard error (SE), the stat (Wald statistic), the pvalue (the Wald test p-value), Sig (the fdr adjusted p value padj, sig < 0.05; padj > 0.05 = not sig), the Symbol (Gene Symbol), the Entrez (entrez gene-id), the Name (gene name), the Start (gene start position) and the End (gene end position). Shown are comparisons between WT with stimulation/WT without stimulation (WT stim.vs WT unstim.), OCT3 KO stim. vs OCT3 KO unstim, OCT3 KO unstim. vs WT unstim, OCT3 KO stim. vs WT stim.). The data from the metabolomic analysis of mouse sera, BMCs pellets, and supernatants are also available as supplementary material to this submission in form of an Excel spreadsheet, where the name of identified metabolite, the mass spectrometry measurement modus (MS Modus: HILIC, RP), the sum formula of metabolite (Formula), the calculated molecular weight (Calc. MW), the mass/charge ratio for the metabolite (m/z), the retention time (RT [min]), the peak area (Area (Max.)), the Annotation Level, the mzCloud Best Match, the name of the investigated group,the Group Area (median), the area Ratio between the groups, the Log2 Fold Change between the groups, the p-value of the comparison between the groups, the adjusted p-value of the comparison between the groups according to Benjamini and Hochberg correction.
