# Supplementary material for: Guilty by association: direct interaction with the tetraspanin CD63 suggests a role for organic cation transporter 3 in histamine release from granulocytes
Source: J Biomed Sci. 2025 Jul 12;32:68. doi: 10.1186/s12929-025-01158-2 (PMC12255121; doi:10.1186/s12929-025-01158-2)
Supplement: Supplementary file 4 — Supplementary material 4. [file 12929_2025_1158_MOESM4_ESM.docx]

Supplementary Materials to:

Guilty by association: direct interaction with the tetraspanin CD63 suggests a role for organic cation transporter 3 in histamine release from granulocytes.

Moritz Pernecker^1^, Miriam Dibos^1^, Sophie Götz^1^, Rouvier Al-Monajjed^1^, Vivien Barz^1^, Christian Albiker^1^, Rita Schröter^1^, Ute Neugebauer^1^, Lena Ludwig-Radtke^2^, R. Verena Taudte^2^, Thomas Vogl^3^, Giuliano Ciarimboli^1^

^1^Exp. Nephrology, Med. Clinic D, University Hospital Münster, Münster, Germany; ^2^Core Facility for Metabolomics, Department of Medicine, Philipps University Marburg, Marburg, Germany; ^3^Institute of Immunology, University of Münster, Münster, Germany.

 Supplementary Figure 1: Effect of CD63 transfection on the CD63 and hOCT3 expression in HEK293 cells stably expressing hOCT3 expressed in % of GAPDH expression. The transfection with CD63 strongly increased CD63 mRNA quantity but had no effect on hOCT3 expression. Values are means ± SEM and the number of experiments is indicated above the columns. * indicates statistically significant difference to transfection with empty vector (unpaired t-test, P < 0.05).

Isolation of human basophils.

Human basophils were isolated from buffy coats by depletion of non-basophils using the MACS Basophil Isolation Kit II from Miltenyi (Miltenyi Biotec., Bergisch Gladbach, Germany). The success of basophil isolation was assessed as CD123+/CD203c+ cells using a FACSCalibur flow cytometer (BD Bioscience, San Jose, CA, USA). The anti-human CD203c antibody coupled to phycoerythrin (PE) (CD203c PE) and the anti-human CD123 antibody coupled to fluorescein isothiocyanate (FITC) (CD123 FITC) were purchased from Miltenyi and used at 1:50 dilution. CD203c is a surface marker specific for basophils and mast cells [1], whereas CD123 is the receptor for IL-3 and is also specific for basophils [2]. The acquired data were processed using FlowJo 10.6.2 software (BD Bioscience, San Jose, CA, USA). Cell debris was excluded from the analysis based on scatter signals. The specificity of the labelling was checked using isotype control antibodies. Supplementary Figure 1 shows a representative result of the cytometric analysis of isolated basophils. The mean purity of basophils was 86.5 ± 4.3% (N = 6). Many basophils appear to be activated (they are CD203c positive), probably due to the preparation procedure used for FACS analysis.

The assessment of apoptosis relied on the interaction between annexin V and exposed phosphatidylserine, employing fluorescence-activated cell sorting (FACS). The evaluation of cell necrosis was conducted through the detection of propidium iodide uptake in nonpermeabilized cells. Isolated basophils were cultured at 37°C with 5% CO_2_. At two time points, one and six days after isolation, the cells were washed with 500 µL of FACS-Buffer (comprising PBS with Ca^2+^ and Mg^2+^, 0.5% fetal calf serum, and 0.5% NaN_3_), and then incubated with 5 µL of annexin V-FITC and propidium iodide (5 μg/mL) from BD Biosciences in 100 µL of FACS-Buffer for 25 minutes at 4°C. Subsequently, the cells were washed once again, resuspended in 500 µL of FACS-Buffer, and immediately subjected to analysis using a FACSCalibur flow cytometer from BD Biosciences. A minimum of 20.000 events were triggered using forward-scatter and side-scatter light, and data were analyzed using FlowJo_v10.6.2 software from BD Biosciences. Cells that tested positive for annexin V-FITC and negative for propidium iodide were classified as early apoptotic, whereas double-positive cells were designated as late apoptotic. Cells that stained positive for propidium iodide alone were predominantly characterized as necrotic.


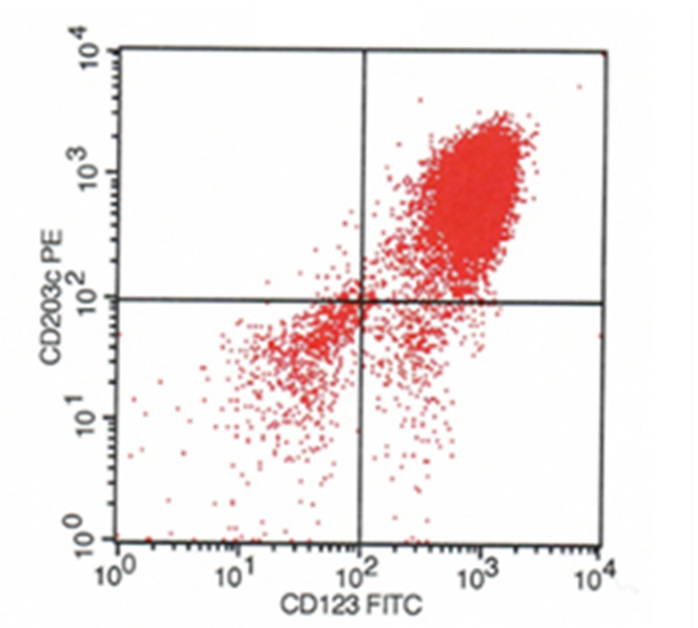


Supplementary Figure 2: Example of FACS analysis of freshly isolated basophils using CD123-FITC and CD203c-PE. The resulting data were categorised into four different quadrants: 1. The lower left quadrant represented non-basophilic cells (including cells that were both CD123-FITC and CD203c-PE negative). 2. The upper right quadrant contained cells with characteristics of activated basophils (consisting of cells that were both CD123-FITC and CD203c-PE positive). 3. The upper left quadrant was assigned to cells with characteristics of activated basophils (specifically CD203c-PE positive cells). 4. The lower right quadrant contained cells with basophilic properties (CD123-FITC positive cells).


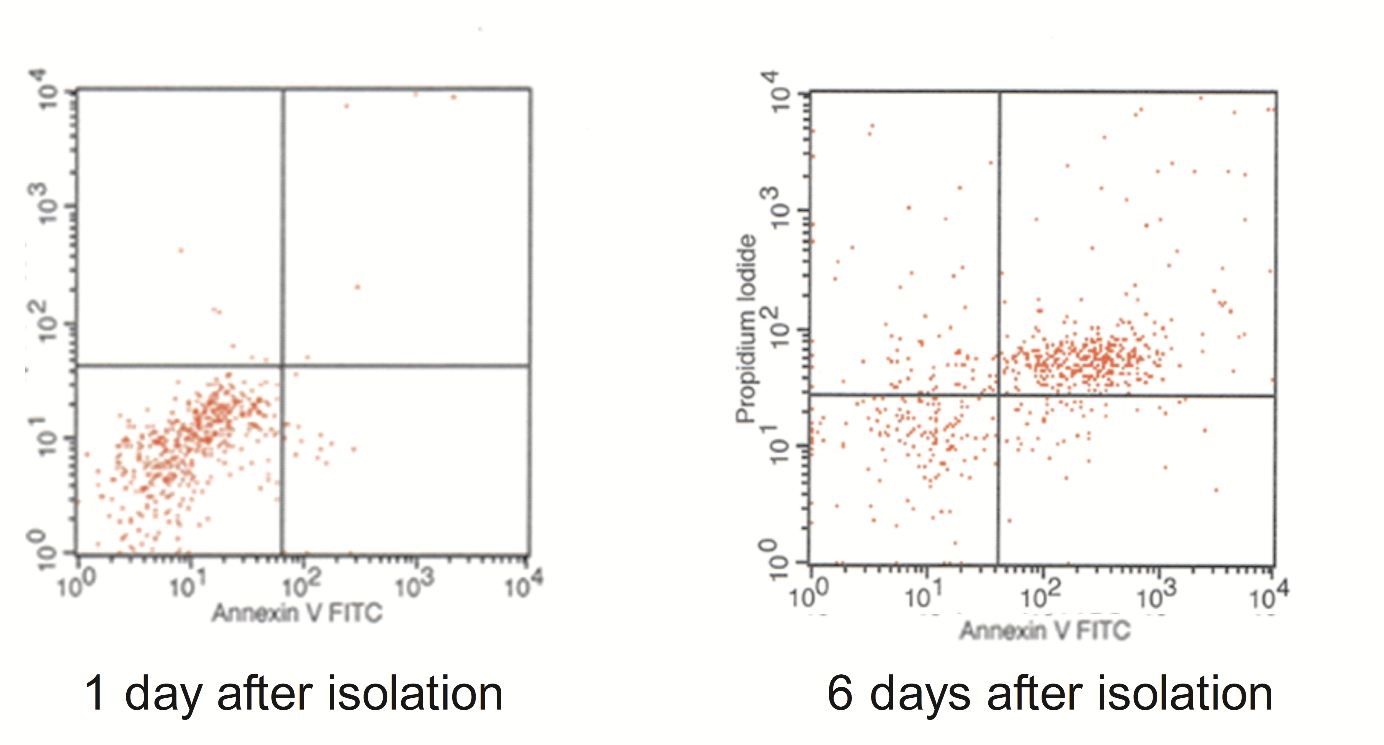


Supplementary Figure 3: Example of FACS analysis performed on isolated basophilic granulocytes using annexin-V-FITC and propidium iodide at two time points, one day and six days after basophils isolation. The resulting data was categorized into four distinct quadrants: 1. The lower left quadrant represented healthy cells (comprising cells that were both propidium iodide and Annexin V negative). 2. The upper right quadrant contained cells displaying necrotic or apoptotic characteristics (consisting of cells that were both propidium iodide and Annexin V positive). 3. The upper left quadrant was assigned to cells exhibiting necrotic properties (specifically propidium iodide positive cells). 4. The lower right quadrant contained cells displaying apoptotic properties (Annexin V positive cells). Notably, the population of healthy cells constituted nearly 95% of the total cells one day after their isolation, but this proportion decreased to 23% six days after the isolation.

Supplementary Figure 4: Concentration response curves for inhibition of hOCT3-mediated ASP^+^-uptake by histamine, MPP^+^, TPA^+^, and corticosterone. The IC_50_ values for inhibition of ASP^+^-uptake were 1100 (95% confidence interval -CI- of logIC_50_ = -3.0 to -2.9, with 150 degrees of freedom -DF-), 146 (95% CI of logIC_50_ = -3.9 to -3.7, with 77 DF), 24 (95% CI of logIC_50_ = -4.7 to -4.5, with 72 DF), and 4 µM (95% CI of logIC_50_ = -5.4 to -5.3, with 133 DF) for histamine, MPP^+^, TPA^+^, and corticosterone, respectively. These values were in close accordance with was calculated in [3].

Supplementary Figure 5. This figure shows histamine concentration in supernatants from KU812 cells with (dark grey columns) or without (open columns) stimulation with 5 µg/ml IgE and 0.2 µg/ml anti-IgE. The effects of the addition of 10 µM dasatinib (panel A) or 1 mM TPA^+^ (panel B) without (light grey columns) and with (black columns) stimulation are also shown. Histamine concentration (ng/ml) is expressed as mean ± SEM. Asterisks indicate statistically significant differences (**** p < 0.0001, *** p = 0.0005, * p = 0.0165, ANOVA test with Tuckey’s multiple comparison test).

Supplementary Figure 6: Western blot membranes showing OCT3 expression in whole cell lysates and biotinylated membrane fractions from BMCs of WT and CD63⁻/⁻ mice. Lane M indicates molecular weight markers, with molecular weights annotated in kDa. Lanes 1, 3, and 5 correspond to whole lysates from stimulated WT BMCs, control WT BMCs, and WT BMCs 24 hours post-stimulation, respectively. Lanes 2, 4, and 6 contain biotinylated membrane fractions from the same respective WT conditions. Lanes 7, 9, and 11 show whole lysates from stimulated, control, and 24-hour post-stimulation CD63⁻/⁻ BMCs, respectively, while lanes 8, 10, and 12 contain the corresponding biotinylated fractions. OCT3 was detected as a band at an apparent molecular weight of ~55 kDa. In some lanes, a secondary, fainter band was observed at ~70 kDa, likely corresponding to a glycosylated form of OCT3; where visible, the intensity of this band was also evaluated.

Supplementary Figure 7: Comparison of expression levels of A: OCT3; B: VMAT1; C: VMAT2; D: PMAT; E: CPA3; F: CD41; G: CD117; H: FCεR1a as determined by mRNA-Seq-analysis in untreated and treated BMCs from WT-, and OCT3^−/−^-mice. Stars indicate the level of statistical significance.

Supplementary Figure 8: Comparison of expression levels of A: IL-4; B: IL-13; C: IL-6; D: Ltc4s; E: Hdc; F: CD63; as determined by mRNA-Seq-analysis in untreated and treated BMCs from WT-, and OCT3^−/−^-mice. Stars indicate the level of statistical significance.

Supplementary Figure 9: Number of regulated and non-regulated mRNAs in BMCs from WT and OCT3⁻^/^⁻ mice following IL-3 + IgE/anti-IgE treatment. RNA-Seq analysis was performed on BMCs from WT and OCT3⁻^/^⁻ mice, either untreated or treated with IL-3 + IgE/anti-IgE to stimulate basophil maturation and histamine secretion. A total of 35,370 mRNAs were detected, with regulated and non-regulated mRNAs represented in 10x10 dot plots as part of the whole. Panel A shows the comparison of mRNA expression in treated versus untreated BMCs from WT mice. Pink dots: mRNAs upregulated by treatment in both WT and OCT3⁻^/^⁻ BMCs. Yellow dots: mRNAs specifically upregulated in WT BMCs. White dots: mRNAs unchanged in both WT and OCT3⁻^/^⁻ BMCs. Gray dots: mRNAs unchanged specifically in WT BMCs. Green dots: mRNAs downregulated by treatment in both WT and OCT3⁻^/^⁻ BMCs. Blue dots: mRNAs specifically downregulated in WT BMCs. The numbers of mRNAs in each category are indicated on the left. Panel B shows the comparison of mRNA expression in treated versus untreated BMCs from OCT3⁻^/^⁻ mice. Pink dots: mRNAs upregulated by treatment in both WT and OCT3⁻^/^⁻ BMCs. Yellow dots: mRNAs specifically upregulated in OCT3⁻^/^⁻ BMCs. White dots: mRNAs unchanged in both WT and OCT3⁻^/^⁻ BMCs. Gray dots: mRNAs unchanged specifically in OCT3⁻^/^⁻ BMCs. Green dots: mRNAs downregulated by treatment in both WT and OCT3⁻^/^⁻ BMCs. Blue dots: mRNAs specifically downregulated in OCT3⁻^/^⁻ BMCs. The numbers of mRNAs in each category are indicated on the left. Panel C shows the genotype effect on mRNA expression in untreated BMCs from WT and OCT3⁻^/^⁻ mice. Pink dots: mRNAs upregulated by both genotype and treatment in OCT3⁻^/^⁻ BMCs compared to WT BMCs. Yellow dots: mRNAs specifically upregulated by genotype in OCT3⁻^/^⁻ BMCs, independent of treatment. White dots: mRNAs unchanged either by genotype or under treatment. Gray dots: mRNAs unchanged by genotype only. Green dots: mRNAs downregulated by both genotype and treatment. Blue dots: mRNAs specifically downregulated by genotype.

The numbers of mRNAs in each category are indicated on the left. Panel D shows the treatment effect on mRNA expression in BMCs from WT and OCT3⁻^/^⁻ mice. Pink dots: mRNAs upregulated by both genotype and treatment in OCT3⁻^/^⁻ BMCs compared to WT BMCs. Yellow dots: mRNAs specifically upregulated by treatment in OCT3⁻^/^⁻ BMCs. White dots: mRNAs unchanged either by genotype or under treatment. Gray dots: mRNAs unchanged under treatment only. Green dots: mRNAs downregulated by both genotype and treatment. Blue dots: mRNAs specifically downregulated by treatment. The numbers of mRNAs in each category are indicated on the left.


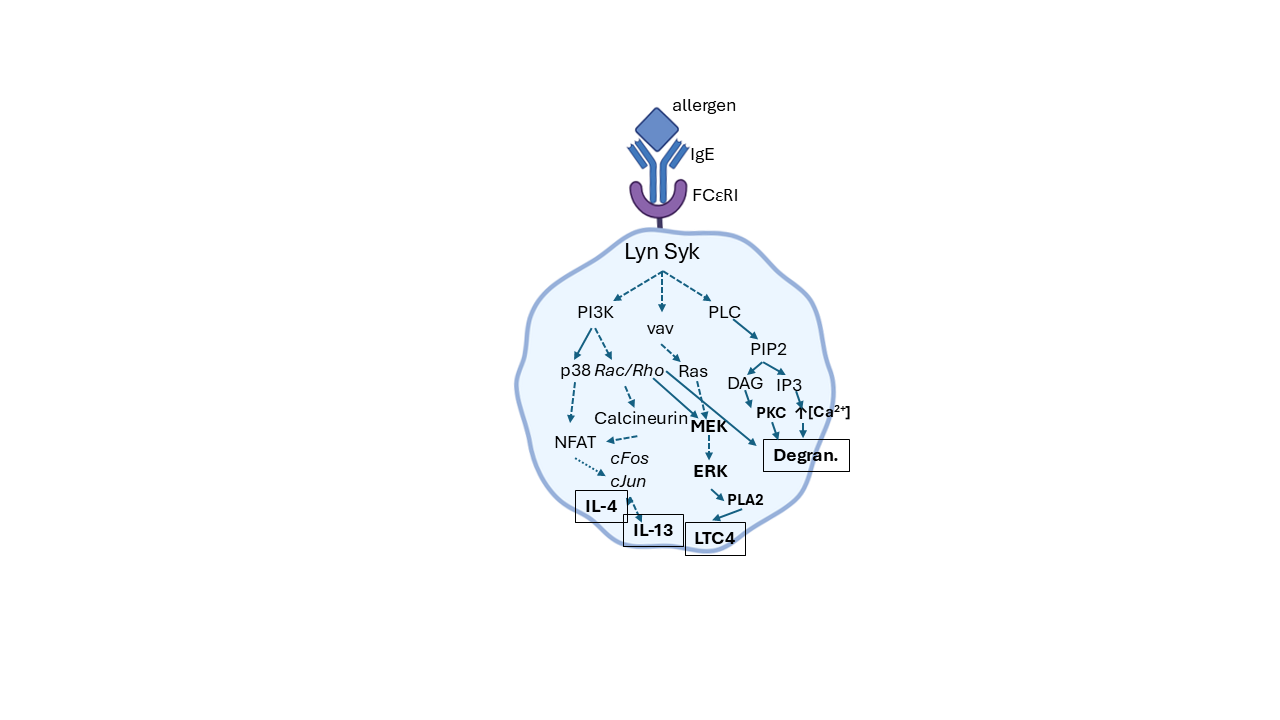


Supplementary Figure 10. Schematic diagram of possible IgE-mediated signaling pathways in basophils. Following high-affinity IgE-receptor cross-linking, various tyrosine kinases are activated, including Lyn and Syk, which consequently lead to the activation of PI3K, ERK-associated MAP kinases (Vav, Ras, Raf1, MEK), and PLC. PI3K targets include both p38MAP-kinase (p38) and Rac/Rho GTPases (Rac/Rho), which are involved in cytokine production. Further, Rac/Rho affect the cytoskeletal processes during degranulation as well as extracellular signal-related kinase-activating kinase (MEK). IgE-dependent ERK activation in basophils is largely limited to controlling Leukotriene C4 (LTC4) generation; PKC is involved in degranulation. The release of calcium from intracellular stores by IP3 together with the influx of the ion through calcium channels affects degranulation. Thicker print is used to highlight what is currently known in human basophils. The remaining has been extrapolated from studies with rodent mast cells or cell lines. Taken from [4]. Created with Biorender.

Supplementary Figure 11: Comparison of expression levels of A: OCT3 (primer sequences (5′→3′) Sense (S): CCTGCTGGAAGCCACTAATACC; Antisense (AS) GTGAGGTCCAACATCCAGGC); B: VMAT2 (S: GCGAAGCAAAGCTATGGCCC; AS: GAACTACGACGGTGAGCAGC; C: PMAT (S: GGTTGAACCTGCACACCAGG; AS: GCAACCAAAGGCCACAGTGC); D: Hdc (S: AACATCATGGACTGGCTGGC; AS: CTCATTAGCATCGGGCTCGC) as determined by Quantitative PCR-analysis in untreated and treated (IL-3, IgE, and anti-IgE) BMCs from WT-, and OCT3^−/−^-mice. Stars indicate the level of statistical significance. GOI = gene of interest. Total RNA was isolated using the Qiagen RNeasy Midikit (Qiagen, Gilden, Germany) and reverse transcription was performed using SuperScript III First-Strand Synthesis SuperMix (Invitrogen, Carlsbad, CA), both according to the manufacturer's recommendation. Real-time PCR was performed using the SYBR Select Master Mix for CFX (Thermo Fisher, Waltham, MA, USA) on a CFX Realtime Detection System (Biorad, Hercules, CA, USA). Relative gene expression values were evaluated with the 2^−ΔΔCt^ method using glyceraldehyde-3-phosphate dehydrogenase (Gapdh, S: TGG CCT TCC GTG TTC CTA CC; AS: GGT CCT CAG TGT AGC CCA) as housekeeping genes [5]. Values are means ± SEM and the number of experiments is indicated above the columns.

Supplementary Figure 12. Heatmap of gene expression changes in the eosinophil chemotaxis pathway (panel A), granulocyte activation pathway (panel B) and histidine transport and metabolism (panel C) in bone marrow cells (BMCs) from wild-type (WT) and OCT3 knockout (OCT3⁻/⁻) mice following IL-3, IgE, and anti-IgE stimulation. The heatmap presents log₂ fold change values derived from RNA-Seq analysis for genes involved in these pathways. Each row corresponds to a specific mRNA transcript, while columns represent BMCs from WT and OCT3⁻/⁻ mice. The color gradient reflects relative expression levels, with yellow indicating upregulation and blue indicating downregulation. No significant difference in the pattern of gene expression between stimulated BMCs from WT and OCT3⁻/⁻ mice was detected, except for Aoc1, which after stimulation is significantly higher expressed in BMCs from OCT3⁻/⁻ mice.

Supplementary Figure 13: Principal component (PC) analysis of detected metabolites of sera isolated from male/female WT and OCT3^−/−^ mice. Blotting PC1 (13.2 %) against PC2 (11 %) shows a low variance between male WT (violet dots), male OCT3^−/−^ (green dots), female WT (blue dots) and female OCT3^−/−^ (red dots) mice in sera. The PC analysis of sera shows no strong grouping of the animals investigated. This may be due to high variability, since samples can still exhibit high variability in other dimensions that PCA may not fully capture.

References

1. Bühring HJ, Simmons PJ, Pudney M, Müller R, Jarrossay D, van Agthoven A, et al. The monoclonal antibody 97A6 defines a novel surface antigen expressed on human basophils and their multipotent and unipotent progenitors. Blood. 1999;94:2343–56.

2. Sonder SU, Plassmeyer M, Loizou D, Alpan O. Towards standardizing basophil identification by flow cytometry. Frontiers in Allergy. 2023;4.

3. Massmann V, Edemir B, Schlatter E, Al-Monajjed R, Harrach S, Klassen P, et al. The organic cation transporter 3 (OCT3) as molecular target of psychotropic drugs: transport characteristics and acute regulation of cloned murine OCT3. Pflügers Arch. 2014;466:517–27. http://www.ncbi.nlm.nih.gov/pubmed/23982114

4. Falcone FH, Haas H, Gibbs BF. The human basophil: a new appreciation of its role in immune responses. Blood. 2000;96:4028–38.

5. Livak KJ, Schmittgen TD. Analysis of relative gene expression data using real-time quantitative PCR and the 2(-Delta Delta C(T)) Method. Methods. 2001;25:402–8. http://www.ncbi.nlm.nih.gov/pubmed/11846609

Supplementary Materials - Table 1. Sequences of primers used in this study.

| Abbreviation  (h = human) | Name | Product-length (basepairs) | Primer-Sequence (5‘-3‘) |
| --- | --- | --- | --- |
| hGAPDH | Glyceraldehyde-3-phosphate dehydrogenase | 180 | Sense (s): CAA GCT CAT TTC CTG GTA TGA C |
|  |  |  | Anti-Sense (As): GTG TGG TGG GGG ACT GAG TGT GG |
| hMATE 1 | Multidrug- und Toxin-Extrusionsprotein 1 | 180 | S: AAG CTG GAG CTG GAT GCA GTC |
|  |  |  | AS: CAG CAG AGG AGC AGG ACG AGC |
| hOCT1 | Organic Cation Transporter 1 | 206 | S: CAT CAT AAT CAT GTG TGT TGG CC |
|  |  |  | AS: CAA  ACA AAA TGA GGG GCA AGG CTT |
| hOCT2 | Organic Cation Transporter 2 | 176 | S: gtt ggg cgg aga tat cgg ag |
|  |  |  | AS: aag gcc cat gtg cat gat ga |
| hOCT3 | Organic Cation Transporter 3 | 431 | S: GAC AAG AGA AGC CCC CAA CCT GAT |
|  |  |  | AS: CAC TAA AGG AGA GCC AAA AAT GTC |
| HDC | Histidine decarboxylase | 187 | S: TGC CAT CAA CTG CTT GGG AT |
|  |  |  | AS: CAG CAG GGC AAT CAA AGT GG |
| hCD6 | CD63 | 132 | S: gcc tgt gtg gag aag att ggg |
|  |  |  | AS: cac ttc tga tac tct tca cga gg |
| NK1 (TACR1) | Neurokinin-1-receptor (Substance-P-Receptor) | 186 | S: CTG CTG GTG ATT GGC TAT GC |
|  |  |  | AS: AGG AGG AAG AAG ATG TGG AAG G |
| hHRH 1 | Histamine Receptor 1 | 145 | S: ATC TGC TGG GAA ACT GAC CAA |
|  |  |  | AS: GCG GAC AAC TTC TCT ACG GC |
| hHRH 2 | Histamine Receptor 2 | 153 | S: GGA GTG GGA CAG AAG TCA ACG |
|  |  |  | AS: CCT GGC ATG TGG TGG GAA T |
| hHRH 3 | Histamine Receptor 3 | 185 | S: GCC CCC AGA AGC TCA AAA TC |
|  |  |  | AS: TTA AGA GAG GGC CAC AGA CAC |
| hHRH 4 | Histamine Receptor 4 | 196 | S: TCA CAC GCT GTT CGA ATG GG |
|  |  |  | AS: AAC GGC CAC CAT CAG AGT AA |

The primers were from Eurofins (Eurofins, Ebersberg, Deutschland)

Supplementary Materials - Table 2

Complete blood count and the white blood cell differential of male WT- and OCT3^−/−^-mice

| Parameter | WT mice | OCT3^−/−^ mice |
| --- | --- | --- |
| Leucocytes (x10^3^/µl) | 4.3 ± 1.2, N = 6 | 6.6 ± 0.8, N = 7 |
| Erythrocytes (x10^6^/µl) | 9.1 ± 0.1, N = 6 | 8.7 ± 0.2, N = 6 |
| Hemoglobin (gd/dl) | 13.3 ± 0.2, N = 6 | 12.7 ± 0.2, N = 6 |
| Hematocrit (%) | 45.7 ± 1.0, N = 6 | 43.4 ± 0.9, N = 6 |
| Mean Corpuscolar Volume (fl) | 50.0 ± 0.6, N = 6 | 50.0 ± 0.4, N = 7 |
| Mean Cellular Hemoglobin (pg) | 14.5 ± 0.2, N = 6 | 14.7 ± 0.1, N = 7 |
| Mean Corpuscolar/Cellular Hemoglobin (g/dl) | 29.1 ± 0.4, N = 6 | 30.0 ± 0.3, N = 7 |
| Thrombocytes (x10^3^/µl) | 1182 ± 75, N = 6 | 1216 ± 106, N = 7 |
| Mean Thrombocytes Volume (fl) | 6.9 ± 0.1, N = 6 | 6.9 ± 0.1, N = 4 |
| Immature Thrombocytes (%) | 0.2 ± 0.1, N = 6 | 0.2 ± 0.04, N = 7 |
| Lymphocytes (x10^3^/µl) | 3.7 ± 1.2, N = 6 | 4.6 ± 0.8, N = 4 |
| Neutrophils (x10^3^/µl) | 0.6 ± 0.1, N = 6 | 0.7 ± 0.04, N = 4 |
| Monocytes (x10^3^/µl) | 0.02 ± 0.01, N = 6 | 0.06 ± 0.02, N = 7 |
| Eosinophils (x10^3^/µl) | 0.004 ± 0.003, N = 6 | 0.05 ± 0.02, N = 7 |
| Basophils (x10^3^/µl) | 0.008 ± 0.002, N = 6 | 0.005 ± 0.002, N = 6 |
| Immature Granulocytes (x10^3^/µl) | 0.0 ± 0.0, N = 6 | 0.001 ± 0.007, N = 4 |
| Reticulocytes (x10^3^/µl) | 232.8 ± 30.4, N = 6 | 199.4 ± 25.6, N = 7 |
| Reticulocytes (%) | 2.5 ± 0.3, N = 6 | 2.3 ± 0.3, N = 7 |
| Reticulocytes-Hemoglobin (pg) | 17.7 ± 0.3, N = 6 | 18.0 ± 0.1, N = 7 |

| Supplementary Materials - Table 3. Results of RNA-Seq analysis of unstimulated and stimulated BMCs isolated from OCT3^−/−^ and WT-mice. Significant regulation (adjusted p-value < 0.05) of mRNA expression with log2 fold change <-2 and >2 is shown. Green color indicates down-regulated and red color upregulated mRNA expression. The following comparisons are listed:   1. Comparison of mRNA expression in unstimulated BMCs from OCT3^−/−^- and WT-mice 2. Comparison of mRNA expression in stimulated BMCs from OCT3^−/−^- and WT-mice 3. Comparison of mRNA expression in stimulated and unstimulated BMCs from WT-mice 4. Comparison of mRNA expression in stimulated and unstimulated BMCs from OCT3^−/−^- mice | | |  |  |
| --- | --- | --- | --- | --- |
| 1. **Comparison of mRNA expression in unstimulated BMCs from OCT3^−/−^- and WT-mice** | | |  |  |
|  | log2  Fold Change | Name |  |  |
|  | -8.0 | solute carrier family 22 (organic cation transporter), member 3 |  |  |
|  | -7.4 | BAI1-associated protein 3 |  |  |
|  | -7.3 | fibroblast growth factor 3 |  |  |
|  | -5.8 | RIKEN cDNA E030030I06 gene |  |  |
|  | -4.7 | RIKEN cDNA 5830444B04 gene |  |  |
|  | -4.6 | cDNA sequence AF067061 |  |  |
|  | -3.4 | RAB6B, member RAS oncogene family |  |  |
|  | -3.2 | thymosin beta 15b1 |  |  |
|  | -2.7 | RIKEN cDNA 4930481A15 gene |  |  |
|  | -2.4 | vanin 3 |  |  |
|  | -2.3 | intraflagellar transport 140 |  |  |
|  | -2.1 | immunoglobulin kappa chain variable 2-137 |  |  |
|  | -2.1 | matrix Gla protein |  |  |
|  | -2.0 | immunoglobulin kappa variable 16-104 |  |  |
|  | 2.2 | dynein light chain Tctex-type 2A2 |  |  |
|  | 2.4 | cystatin domain containing 6 |  |  |
|  | 2.5 | NHERF family PDZ scaffold protein 2 |  |  |
|  | 2.6 | zinc finger protein 985 |  |  |
|  | 2.8 | malic enzyme 1, NADP(+)-dependent, cytosolic |  |  |
|  | 2.8 | predicted gene, 41341 |  |  |
|  | 2.8 | epithelial membrane protein 1 |  |  |
|  | 3.2 | zinc finger protein 534 |  |  |
|  | 3.4 | desmocollin 2 |  |  |
|  | 3.4 | cyclin B1 interacting protein 1 |  |  |
|  | 4.6 | transmembrane protein 200B |  |  |
|  | 4.7 | noncompact myelin associated protein |  |  |
|  | 4.7 | histocompatibility 60b |  |  |
|  | 5.1 | malic enzyme 1, NADP(+)-dependent, cytosolic pseudogene |  |  |
|  | 5.5 | Protease, serine 41 |  |  |
|  | 5.9 | Ribonuclease, RNase A family, 2A (liver, eosinophil-derived neurotoxin) |  |  |
|  | 7.0 | predicted gene 27216 |  |  |
|  | 7.0 | zinc finger protein 990 |  |  |
|  | 10.0 | chloride channel, voltage-sensitive Ka |  |  |
| Total: 33 |  |  |  |  |
| **B. Comparison of mRNA expression in stimulated BMCs from OCT3^−/−^- and WT-mice** | | |  |  |
|  | -8.3 | BAI1-associated protein 3 |  |  |
|  | -6.0 | fibroblast growth factor 3 |  |  |
|  | -5.7 | solute carrier family 22 (organic cation transporter), member 3 |  |  |
|  | -4.0 | RIKEN cDNA 5830444B04 gene |  |  |
|  | -3.9 | thymosin beta 15b2 |  |  |
|  | -3.5 | RAB6B, member RAS oncogene family |  |  |
|  | -3.3 | androglobin |  |  |
|  | -3.3 | thymosin beta 15b1 |  |  |
|  | -2.6 | thymosin beta 15b like |  |  |
|  | -2.2 | calcium channel, voltage-dependent, T type, alpha 1H subunit |  |  |
|  | -2.1 | RIKEN cDNA 4930481A15 gene |  |  |
|  | 2.0 | mucin 6, gastric |  |  |
|  | 2.1 | glyoxalase 1, pseudogene |  |  |
|  | 2.2 | ribonuclease, RNase A family, 2A (liver, eosinophil-derived neurotoxin) |  |  |
|  | 2.3 | zinc finger protein 949 |  |  |
|  | 2.6 | histocompatibility 2, M region locus 2 |  |  |
|  | 2.7 | zinc finger protein 985 |  |  |
|  | 2.7 | predicted gene, 41341 |  |  |
|  | 2.8 | histocompatibility 60b |  |  |
|  | 2.9 | NHERF family PDZ scaffold protein 2 |  |  |
|  | 3.1 | zinc finger protein 534 |  |  |
|  | 3.8 | RIKEN cDNA C030013G03 gene |  |  |
|  | 4.0 | cell adhesion molecule 1 |  |  |
|  | 4.1 | noncompact myelin associated protein |  |  |
|  | 5.1 | protease, serine 41 |  |  |
|  | 5.4 | meiosis specific with OB domains |  |  |
|  | 6.4 | glutamate receptor, metabotropic 4 |  |  |
|  | 6.6 | predicted gene 27216 |  |  |
|  | 7.0 | zinc finger protein 990 |  |  |
|  | 9.4 | chloride channel, voltage-sensitive Ka |  |  |
| Total: 30 |  |  |  |  |
| **C. Comparison of mRNA expression in stimulated and unstimulated BMCs from WT-mice** | | |  |  |
|  | -14.0 | polycystic kidney and hepatic disease 1-like 1 |  |  |
|  | -11.3 | angiotensin II receptor, type 1a |  |  |
|  | -10.7 | calcium channel, voltage-dependent, T type, alpha 1G subunit |  |  |
|  | -10.5 | phytanoyl-CoA hydroxylase interacting protein |  |  |
|  | -10.2 | tachykinin 2 |  |  |
|  | -10.1 | protein disulfide isomerase associated 2 |  |  |
|  | -10.1 | RIKEN cDNA A730036I17 gene |  |  |
|  | -9.5 | SH3 domain and tetratricopeptide repeats 2 |  |  |
|  | -9.1 | solute carrier family 30, member 10 |  |  |
|  | -9.0 | polycystic kidney disease 1 like 1 |  |  |
|  | -9.0 | TLC domain containing 4 |  |  |
|  | -8.9 | Rhesus blood group-associated A glycoprotein |  |  |
|  | -8.8 | butyrophilin-like 10 |  |  |
|  | -8.8 | ATP binding cassette subfamily G member 4 |  |  |
|  | -8.7 | acyl-CoA synthetase long-chain family member 6 |  |  |
|  | -8.7 | fructosamine 3 kinase |  |  |
|  | -8.5 | Rh blood group, D antigen |  |  |
|  | -8.4 | SH2 domain containing 4A |  |  |
|  | -8.3 | ring finger protein 212 |  |  |
|  | -8.3 | apolipoprotein L 10C, pseudogene |  |  |
|  | -8.3 | glial fibrillary acidic protein |  |  |
|  | -8.1 | AKNA domain containing 1 |  |  |
|  | -8.1 | pyruvate kinase liver and red blood cell |  |  |
|  | -8.1 | predicted gene, 37915 |  |  |
|  | -7.8 | Redrum, erythroid developmental long intergenic non-protein coding transcript |  |  |
|  | -7.8 | microRNA 144 |  |  |
|  | -7.8 | erythroblast membrane-associated protein |  |  |
|  | -7.8 | ankyrin repeat and SOCS box-containing 17 |  |  |
|  | -7.8 | RIKEN cDNA 9830132P13 gene |  |  |
|  | -7.7 | apolipoprotein L 8 |  |  |
|  | -7.7 | adducin 2 (beta) |  |  |
|  | -7.5 | glycerophosphodiester phosphodiesterase domain containing 2 |  |  |
|  | -7.5 | solute carrier family 38, member 5 |  |  |
|  | -7.5 | ankyrin 1, erythroid |  |  |
|  | -7.4 | sphingosine-1-phosphate receptor 5 |  |  |
|  | -7.4 | sosondowah ankyrin repeat domain family member A |  |  |
|  | -7.4 | solute carrier family 6 (neurotransmitter transporter), member 20A |  |  |
|  | -7.3 | RIKEN cDNA 1810053B23 gene |  |  |
|  | -7.2 | SRY (sex determining region Y)-box 6 |  |  |
|  | -7.2 | solute carrier family 2 (facilitated glucose transporter), member 4 |  |  |
|  | -7.1 | collagen, type XIV, alpha 1 |  |  |
|  | -7.1 | microRNA 451a |  |  |
|  | -7.1 | solute carrier family 25 (mitochondrial oxodicarboxylate carrier), member 21 |  |  |
|  | -7.1 | aquaporin 1 |  |  |
|  | -7.1 | angiotensin I converting enzyme (peptidyl-dipeptidase A) 1 |  |  |
|  | -7.1 | leucine rich repeat containing 39 |  |  |
|  | -7.1 | Kruppel-like factor 1 (erythroid) |  |  |
|  | -7.0 | predicted gene 11837 |  |  |
|  | -7.0 | amino carboxymuconate semialdehyde decarboxylase |  |  |
|  | -7.0 | atypical chemokine receptor 4 |  |  |
|  | -7.0 | solute carrier family 26 (sulfate transporter), member 1 |  |  |
|  | -7.0 | inhibitor of carbonic anhydrase |  |  |
|  | -7.0 | hemogen |  |  |
|  | -6.9 | transient receptor potential cation channel, subfamily V, member 5 |  |  |
|  | -6.9 | dematin actin binding protein |  |  |
|  | -6.9 | neurexophilin and PC-esterase domain family, member 2 |  |  |
|  | -6.8 | ADAM-like, decysin 1 |  |  |
|  | -6.8 | Wnt inhibitory factor 1 |  |  |
|  | -6.8 | fer-1-like 6 (C. elegans) |  |  |
|  | -6.8 | carbonic anhydrase 1 |  |  |
|  | -6.7 | butyrophilin, subfamily 1, member A1 |  |  |
|  | -6.7 | CD209f antigen |  |  |
|  | -6.7 | phospholamban |  |  |
|  | -6.7 | spectrin alpha, erythrocytic 1 |  |  |
|  | -6.6 | LON peptidase N-terminal domain and ring finger 2 |  |  |
|  | -6.6 | predicted gene 15290 |  |  |
|  | -6.6 | predicted gene 6249 |  |  |
|  | -6.6 | flavin containing monooxygenase 2 |  |  |
|  | -6.6 | golgi associated RAB2 interactor family member 4 |  |  |
|  | -6.6 | predicted gene 867 |  |  |
|  | -6.5 | integrin binding sialoprotein |  |  |
|  | -6.5 | spectrin beta, erythrocytic |  |  |
|  | -6.5 | Fas apoptotic inhibitory molecule 2 |  |  |
|  | -6.4 | RIKEN cDNA 5430401H09 gene |  |  |
|  | -6.4 | carbonic anhydrase 1 pseudogene |  |  |
|  | -6.4 | agmatine ureohydrolase (agmatinase) |  |  |
|  | -6.4 | sterile alpha motif domain containing 11 |  |  |
|  | -6.4 | bone morphogenic protein/retinoic acid inducible neural-specific 2 |  |  |
|  | -6.4 | DEPP1 autophagy regulator |  |  |
|  | -6.4 | olfactory receptor family 52 subfamily Z member 1 |  |  |
|  | -6.3 | hemoglobin X, alpha-like embryonic chain in Hba complex |  |  |
|  | -6.3 | carboxylesterase 2G |  |  |
|  | -6.2 | G-protein coupled receptor 88 |  |  |
|  | -6.2 | ankyrin repeat and SOCS box-containing 17, opposite strand |  |  |
|  | -6.2 | beta-1,4-N-acetyl-galactosaminyl transferase 3 |  |  |
|  | -6.0 | tetraspanin 33 |  |  |
|  | -6.0 | erythrocyte membrane protein band 4.2 |  |  |
|  | -6.0 | ribosomal protein S3 pseudogene |  |  |
|  | -6.0 | solute carrier family 4 (anion exchanger), member 1 |  |  |
|  | -5.9 | sorting nexin 22 |  |  |
|  | -5.9 | apolipoprotein L 11a |  |  |
|  | -5.9 | aldolase B, fructose-bisphosphate |  |  |
|  | -5.9 | claudin 13 |  |  |
|  | -5.9 | calmegin |  |  |
|  | -5.9 | bone morphogenetic protein 8b |  |  |
|  | -5.9 | Kell blood group |  |  |
|  | -5.9 | aldehyde dehydrogenase family 1, subfamily A7 |  |  |
|  | -5.9 | RIKEN cDNA 9430069I07 gene |  |  |
|  | -5.8 | CD40 ligand |  |  |
|  | -5.8 | pleckstrin 2 |  |  |
|  | -5.8 | tripartite motif-containing 15 |  |  |
|  | -5.8 | cyclin M1 |  |  |
|  | -5.7 | membrane metallo endopeptidase |  |  |
|  | -5.7 | membrane metallo-endopeptidase-like 1 |  |  |
|  | -5.6 | predicted gene 14862 |  |  |
|  | -5.6 | tetraspanin 8 |  |  |
|  | -5.6 | folate receptor 1 (adult) |  |  |
|  | -5.6 | predicted gene, 33934 |  |  |
|  | -5.6 | predicted gene 15816 |  |  |
|  | -5.6 | chemokine (C-X-C motif) ligand 9 |  |  |
|  | -5.6 | apolipoprotein L 10A |  |  |
|  | -5.5 | contactin 3 |  |  |
|  | -5.5 | protease, serine 50 |  |  |
|  | -5.5 | butyrophilin-like 6 |  |  |
|  | -5.5 | predicted gene, 20161 |  |  |
|  | -5.5 | tripartite motif-containing 10 |  |  |
|  | -5.5 | thrombospondin, type I, domain containing 7B |  |  |
|  | -5.5 | WAP four-disulfide core domain 3 |  |  |
|  | -5.4 | yippee like 4 |  |  |
|  | -5.4 | progestin and adipoQ receptor family member IX |  |  |
|  | -5.4 | prostaglandin D receptor |  |  |
|  | -5.4 | microRNA 486 |  |  |
|  | -5.4 | predicted gene, 33148 |  |  |
|  | -5.4 | coiled-coil domain containing 92B |  |  |
|  | -5.4 | calcineurin-like EF hand protein 2 |  |  |
|  | -5.4 | RIKEN cDNA gene D630044L22 gene |  |  |
|  | -5.3 | ATPase, Na+/K+ transporting, beta 2 polypeptide |  |  |
|  | -5.3 | G protein-coupled receptor 182 |  |  |
|  | -5.3 | leukocyte immunoglobulin-like receptor, subfamily A (with TM domain), member 5 |  |  |
|  | -5.3 | artemin |  |  |
|  | -5.3 | solute carrier family 26, member 7 |  |  |
|  | -5.2 | nuclear RNA export factor 3 |  |  |
|  | -5.2 | popeye domain containing 2 |  |  |
|  | -5.2 | RIKEN cDNA 5430431A17 gene |  |  |
|  | -5.2 | potassium inwardly-rectifying channel, subfamily J, member 9 |  |  |
|  | -5.2 | a disintegrin and metallopeptidase domain 33 |  |  |
|  | -5.2 | TLR4 interactor with leucine-rich repeats |  |  |
|  | -5.2 | RIKEN cDNA F930017D23 gene |  |  |
|  | -5.2 | growth hormone releasing hormone receptor |  |  |
|  | -5.2 | predicted gene 2061 |  |  |
|  | -5.2 | potassium inwardly-rectifying channel, subfamily J, member 10 |  |  |
|  | -5.2 | angiopoietin-like 1 |  |  |
|  | -5.1 | predicted gene, 24265 |  |  |
|  | -5.1 | ATPase, Cu++ transporting, beta polypeptide |  |  |
|  | -5.1 | ring finger protein 165 |  |  |
|  | -5.1 | carbonic anhydrase 2 |  |  |
|  | -5.1 | carboxyesterase 2B |  |  |
|  | -5.1 | UDP galactosyltransferase 8A |  |  |
|  | -5.1 | predicted gene, 16793 |  |  |
|  | -5.1 | phosphate regulating endopeptidase homolog, X-linked |  |  |
|  | -5.0 | predicted gene 13010 |  |  |
|  | -5.0 | RIKEN cDNA 2610027K06 gene |  |  |
|  | -5.0 | PPARGC1 and ESRR induced regulator, muscle 1 |  |  |
|  | -5.0 | dipeptidylpeptidase 6 |  |  |
|  | -4.9 | fibronectin type III domain containing 11 |  |  |
|  | -4.9 | sosondowah ankyrin repeat domain family member D |  |  |
|  | -4.9 | VANGL planar cell polarity 1 |  |  |
|  | -4.9 | acyl-coenzyme A amino acid N-acyltransferase 1 |  |  |
|  | -4.9 | calsequestrin 2 |  |  |
|  | -4.9 | leptin receptor |  |  |
|  | -4.9 | RUNX1 translocation partner 1 |  |  |
|  | -4.9 | chemokine (C-X3-C motif) receptor 1 |  |  |
|  | -4.9 | predicted gene, 47996 |  |  |
|  | -4.9 | SEC14-like lipid binding 2 |  |  |
|  | -4.9 | family with sequence similarity 205, member A2 |  |  |
|  | -4.9 | angiotensinogen (serpin peptidase inhibitor, clade A, member 8) |  |  |
|  | -4.9 | IQ motif containing D |  |  |
|  | -4.8 | solute carrier family 6 (neurotransmitter transporter), member 20B |  |  |
|  | -4.8 | ATP-binding cassette, sub-family B (MDR/TAP), member 4 |  |  |
|  | -4.8 | membrane protein, palmitoylated 2 (MAGUK p55 subfamily member 2) |  |  |
|  | -4.8 | predicted gene 27179 |  |  |
|  | -4.8 | major intrinsic protein of lens fiber |  |  |
|  | -4.8 | thyroid hormone responsive |  |  |
|  | -4.8 | FH2 domain containing 1 |  |  |
|  | -4.8 | G-protein-coupled receptor 50 |  |  |
|  | -4.8 | fin bud initiation factor homolog (zebrafish) |  |  |
|  | -4.8 | dentin matrix protein 1 |  |  |
|  | -4.8 | erythroferrone |  |  |
|  | -4.7 | solute carrier family 43, member 1 |  |  |
|  | -4.7 | secretogranin V |  |  |
|  | -4.7 | solute carrier family 25, member 37 |  |  |
|  | -4.7 | DnaJ heat shock protein family (Hsp40) member A4 |  |  |
|  | -4.7 | killer cell lectin-like receptor subfamily A, member 14, pseudogene |  |  |
|  | -4.7 | predicted gene, 30292 |  |  |
|  | -4.7 | FBJ osteosarcoma oncogene B |  |  |
|  | -4.7 | V-set and immunoglobulin domain containing 4 |  |  |
|  | -4.7 | small integral membrane protein 1 |  |  |
|  | -4.7 | SET domain containing 9, pseudogene |  |  |
|  | -4.7 | unc-79 homolog |  |  |
|  | -4.7 | syncytin b |  |  |
|  | -4.7 | microRNA 6903 |  |  |
|  | -4.7 | coiled-coil domain containing 74A |  |  |
|  | -4.6 | MAGE family member B16 |  |  |
|  | -4.6 | synaptotagmin XIV |  |  |
|  | -4.6 | tripartite motif-containing 55 |  |  |
|  | -4.6 | erythropoietin receptor |  |  |
|  | -4.6 | sphingosine-1-phosphate phosphatase 2 |  |  |
|  | -4.6 | cytochrome P450, family 2, subfamily b, polypeptide 10 |  |  |
|  | -4.6 | telomere repeat binding bouquet formation protein 1 |  |  |
|  | -4.6 | RIKEN cDNA 9530026F06 gene |  |  |
|  | -4.6 | serine rich and transmembrane domain containing 2 |  |  |
|  | -4.6 | carboxylesterase 2E |  |  |
|  | -4.6 | plasma membrane proteolipid |  |  |
|  | -4.6 | divergent protein kinase domain 1C |  |  |
|  | -4.6 | DnaJ heat shock protein family (Hsp40) member B3 |  |  |
|  | -4.5 | intercellular adhesion molecule 4, Landsteiner-Wiener blood group |  |  |
|  | -4.5 | PDZ domain containing 1 |  |  |
|  | -4.5 | DENN domain containing 2B |  |  |
|  | -4.5 | glycophorin A |  |  |
|  | -4.5 | plasminogen |  |  |
|  | -4.5 | complement receptor 2 |  |  |
|  | -4.5 | odd-skipped related 2 |  |  |
|  | -4.5 | solute carrier family 22 (organic anion transporter), member 8 |  |  |
|  | -4.5 | predicted gene, 17344 |  |  |
|  | -4.5 | RasGEF domain family, member 1C |  |  |
|  | -4.5 | microsomal glutathione S-transferase 3 |  |  |
|  | -4.5 | transmembrane serine protease 6 |  |  |
|  | -4.5 | H4 histone 16 |  |  |
|  | -4.5 | cache domain containing 1 |  |  |
|  | -4.4 | osteoglycin |  |  |
|  | -4.4 | predicted gene, 34567 |  |  |
|  | -4.4 | family with sequence similarity 229, member A |  |  |
|  | -4.4 | ATPase, H+ transporting, lysosomal V0 subunit A4 |  |  |
|  | -4.4 | ADP-ribosyltransferase 4 |  |  |
|  | -4.4 | predicted gene, 16867 |  |  |
|  | -4.4 | gamma-aminobutyric acid (GABA) B receptor, 2 |  |  |
|  | -4.4 | chondromodulin |  |  |
|  | -4.4 | myosin, heavy polypeptide 10, non-muscle |  |  |
|  | -4.4 | proline rich 15-like |  |  |
|  | -4.4 | phospholipid phosphatase 1 |  |  |
|  | -4.4 | killer cell lectin-like receptor subfamily A, member 10 |  |  |
|  | -4.4 | apolipoprotein L 11b |  |  |
|  | -4.4 | chloride channel, voltage-sensitive 2 |  |  |
|  | -4.4 | matrix metallopeptidase 21 |  |  |
|  | -4.3 | cyclin-dependent kinase-like 1 (CDC2-related kinase) |  |  |
|  | -4.3 | scavenger receptor class A, member 5 |  |  |
|  | -4.3 | RIKEN cDNA 0610043K17 gene |  |  |
|  | -4.3 | ninjurin 2 |  |  |
|  | -4.3 | 3-oxoacid CoA transferase 2B |  |  |
|  | -4.3 | kininogen 1 |  |  |
|  | -4.3 | Ttc39a opposite strand RNA 1 |  |  |
|  | -4.3 | butyrophilin-like 4 |  |  |
|  | -4.3 | ribonuclease, RNase A family, 2B (liver, eosinophil-derived neurotoxin) |  |  |
|  | -4.3 | spondin 2, extracellular matrix protein |  |  |
|  | -4.3 | transmembrane channel-like gene family 3 |  |  |
|  | -4.3 | family with sequence similarity 205, member A1 |  |  |
|  | -4.3 | aminolevulinate, delta-, dehydratase |  |  |
|  | -4.3 | schlafen 14 |  |  |
|  | -4.3 | neuroligin 3 |  |  |
|  | -4.3 | killer cell lectin-like receptor, subfamily A, member 8 |  |  |
|  | -4.3 | R-spondin 2 |  |  |
|  | -4.3 | transmembrane channel-like gene family 5 |  |  |
|  | -4.2 | ATPase, Ca^++^ transporting, plasma membrane 4 |  |  |
|  | -4.2 | microfibrillar-associated protein 2 |  |  |
|  | -4.2 | predicted gene, 29778 |  |  |
|  | -4.2 | elastin |  |  |
|  | -4.2 | natural cytotoxicity triggering receptor 1 |  |  |
|  | -4.2 | sorting nexin 31 |  |  |
|  | -4.2 | cytochrome P450, family 2, subfamily ab, polypeptide 1 |  |  |
|  | -4.2 | H2B clustered histone 27 |  |  |
|  | -4.2 | ectonucleotide pyrophosphatase/phosphodiesterase 3 |  |  |
|  | -4.2 | NHL repeat containing 4 |  |  |
|  | -4.2 | FERM and PDZ domain containing 4 |  |  |
|  | -4.2 | carboxylesterase 2F |  |  |
|  | -4.2 | serine/threonine/tyrosine kinase 1 |  |  |
|  | -4.2 | coagulation factor II |  |  |
|  | -4.2 | family with sequence similarity 205, member A2 |  |  |
|  | -4.2 | adiponectin, C1Q and collagen domain containing |  |  |
|  | -4.1 | hairy/enhancer-of-split related with YRPW motif-like |  |  |
|  | -4.1 | RIKEN cDNA E330011O21 gene |  |  |
|  | -4.1 | phospholipase A2, group IVC (cytosolic, calcium-independent) |  |  |
|  | -4.1 | ficolin B |  |  |
|  | -4.1 | regulator of G-protein signaling 6 |  |  |
|  | -4.1 | serine and arginine-rich splicing factor 12 |  |  |
|  | -4.1 | patched 2 |  |  |
|  | -4.1 | predicted gene 8947 |  |  |
|  | -4.1 | peptidase M20 domain containing 2 |  |  |
|  | -4.1 | microRNA 3058 |  |  |
|  | -4.0 | kelch repeat and BTB (POZ) domain containing 12 |  |  |
|  | -4.0 | sushi-repeat-containing protein |  |  |
|  | -4.0 | FBJ osteosarcoma oncogene |  |  |
|  | -4.0 | cDNA sequence BC065403 |  |  |
|  | -4.0 | ubiquitin associated domain containing 1 pseudogene |  |  |
|  | -4.0 | nuclear receptor subfamily 4, group A, member 1 |  |  |
|  | -4.0 | bone morphogenetic protein 3 |  |  |
|  | -4.0 | atypical chemokine receptor 1 (Duffy blood group) |  |  |
|  | -4.0 | inositol hexaphosphate kinase 3 |  |  |
|  | -4.0 | GRB2 associated regulator of MAPK1 subtype 1 |  |  |
|  | -4.0 | T cell receptor beta joining 2-1 |  |  |
|  | -4.0 | myosin XVA |  |  |
|  | -4.0 | kelch-like 13 |  |  |
|  | -3.9 | tripartite motif-containing 58 |  |  |
|  | -3.9 | chemokine (C motif) ligand 1 |  |  |
|  | -3.9 | regulatory factor X, 2 (influences HLA class II expression) |  |  |
|  | -3.9 | papilin, proteoglycan-like sulfated glycoprotein |  |  |
|  | -3.9 | predicted gene, 24451 |  |  |
|  | -3.9 | immunoglobulin superfamily containing leucine-rich repeat |  |  |
|  | -3.9 | polycystic kidney disease 2-like 2 |  |  |
|  | -3.9 | hydroxymethylbilane synthase |  |  |
|  | -3.9 | immunoglobulin kappa chain variable 9-128 |  |  |
|  | -3.9 | astrotactin 2 |  |  |
|  | -3.9 | killer cell lectin-like receptor, subfamily A, member 4 |  |  |
|  | -3.9 | IQ motif containing N |  |  |
|  | -3.9 | immunoglobulin heavy variable 7-4 |  |  |
|  | -3.9 | predicted gene, 41043 |  |  |
|  | -3.9 | 2'-5' oligoadenylate synthetase 1E |  |  |
|  | -3.9 | solute carrier organic anion transporter family, member 1a5 |  |  |
|  | -3.9 | cysteine rich protein 2 |  |  |
|  | -3.8 | interleukin 22 receptor, alpha 1 |  |  |
|  | -3.8 | collagen, type XI, alpha 1 |  |  |
|  | -3.8 | uroporphyrinogen III synthase |  |  |
|  | -3.8 | orosomucoid 2 |  |  |
|  | -3.8 | fructosamine 3 kinase related protein |  |  |
|  | -3.8 | collagen, type XXII, alpha 1 |  |  |
|  | -3.8 | ninein-like |  |  |
|  | -3.8 | cerebellin 1 precursor protein |  |  |
|  | -3.8 | solute carrier family 16 (monocarboxylic acid transporters), member 10 |  |  |
|  | -3.8 | olfactory marker protein |  |  |
|  | -3.8 | tyrosine kinase, non-receptor, 1 |  |  |
|  | -3.8 | predicted gene, 31828 |  |  |
|  | -3.8 | aldehyde dehydrogenase family 1, subfamily A1 |  |  |
|  | -3.8 | cytochrome P450, family 4, subfamily b, polypeptide 1 |  |  |
|  | -3.8 | solute carrier family 5 (sodium/glucose cotransporter), member 2 |  |  |
|  | -3.8 | tropomodulin 1 |  |  |
|  | -3.8 | fibroblast growth factor receptor-like 1 |  |  |
|  | -3.8 | platelet-derived growth factor, D polypeptide |  |  |
|  | -3.8 | myopalladin |  |  |
|  | -3.7 | family with sequence similarity 131, member B |  |  |
|  | -3.7 | H2B.U histone 1, pseudogene |  |  |
|  | -3.7 | predicted gene, 22748 |  |  |
|  | -3.7 | immunoglobulin kappa chain variable 1-122 |  |  |
|  | -3.7 | a disintegrin and metallopeptidase domain 30 |  |  |
|  | -3.7 | translocator protein 2 |  |  |
|  | -3.7 | complement component 4B (Chido blood group) |  |  |
|  | -3.7 | ferrochelatase |  |  |
|  | -3.7 | pirin |  |  |
|  | -3.7 | double PHD fingers 3 |  |  |
|  | -3.7 | serine (or cysteine) peptidase inhibitor, clade A, member 3C |  |  |
|  | -3.7 | S100 protein, beta polypeptide, neural |  |  |
|  | -3.7 | RIKEN cDNA C730002L08 gene |  |  |
|  | -3.7 | solute carrier family 22, member 23 |  |  |
|  | -3.7 | transcription factor Dp 2 |  |  |
|  | -3.7 | complement component 6 |  |  |
|  | -3.7 | paraneoplastic antigen MA1 |  |  |
|  | -3.7 | tripartite motif-containing 59 pseudogene |  |  |
|  | -3.7 | glutaminase 2 (liver, mitochondrial) |  |  |
|  | -3.6 | T cell acute lymphocytic leukemia 1 |  |  |
|  | -3.6 | exportin 7 |  |  |
|  | -3.6 | predicted gene, 40372 |  |  |
|  | -3.6 | coiled-coil domain containing 103 |  |  |
|  | -3.6 | solute carrier family 6 (neurotransmitter transporter, glycine), member 9 |  |  |
|  | -3.6 | RIKEN cDNA 4933428G20 gene |  |  |
|  | -3.6 | coproporphyrinogen oxidase |  |  |
|  | -3.6 | C-type lectin domain family 14, member a |  |  |
|  | -3.6 | phospholipase A2, group IID |  |  |
|  | -3.6 | microRNA 7653 |  |  |
|  | -3.6 | doublesex and mab-3 related transcription factor like family A1 |  |  |
|  | -3.6 | translin-associated factor X pseudogene |  |  |
|  | -3.6 | desmocollin 2 |  |  |
|  | -3.6 | coiled-coil domain containing 141 |  |  |
|  | -3.6 | histidine ammonia lyase |  |  |
|  | -3.6 | ADP-ribosylation factor-like 4A |  |  |
|  | -3.6 | CD300E molecule |  |  |
|  | -3.6 | myosin light chain kinase 3 |  |  |
|  | -3.6 | predicted gene, 23690 |  |  |
|  | -3.6 | predicted gene, 47985 |  |  |
|  | -3.6 | expressed sequence AW011738 |  |  |
|  | -3.6 | dehydrogenase/reductase (SDR family) member 11 |  |  |
|  | -3.6 | Rho guanine nucleotide exchange factor (GEF) 37 |  |  |
|  | -3.6 | ATP binding cassette subfamily G member 2 (Junior blood group) |  |  |
|  | -3.6 | membrane bound O-acyltransferase domain containing 2 |  |  |
|  | -3.6 | complement component 1, s subcomponent 1 |  |  |
|  | -3.6 | TLC domain containing 1 |  |  |
|  | -3.6 | hydroxyprostaglandin dehydrogenase 15 (NAD) |  |  |
|  | -3.5 | ubiquitin associated domain containing 1 |  |  |
|  | -3.5 | H2B clustered histone 18 |  |  |
|  | -3.5 | bone morphogenetic protein 4 |  |  |
|  | -3.5 | bone morphogenetic protein 6 |  |  |
|  | -3.5 | T cell receptor beta, variable 3 |  |  |
|  | -3.5 | ATP-binding cassette, sub-family B (MDR/TAP), member 10 |  |  |
|  | -3.5 | T cell receptor beta joining 1-1 |  |  |
|  | -3.5 | a disintegrin and metallopeptidase domain 2 |  |  |
|  | -3.5 | killer cell lectin-like receptor subfamily B member 1F |  |  |
|  | -3.5 | poly(A) binding protein, cytoplasmic 2 pseudogene |  |  |
|  | -3.5 | DENN domain containing 2C |  |  |
|  | -3.5 | vomeronasal 1 receptor 4 |  |  |
|  | -3.5 | inhibitor of DNA binding 4 |  |  |
|  | -3.5 | Kruppel-like factor 2 (lung) |  |  |
|  | -3.5 | pyruvate carboxylase |  |  |
|  | -3.5 | H1.5 linker histone, cluster member |  |  |
|  | -3.5 | immunoglobulin heavy constant alpha |  |  |
|  | -3.4 | WNK lysine deficient protein kinase 4 |  |  |
|  | -3.4 | predicted gene, 47995 |  |  |
|  | -3.4 | zinc finger, C2HC-type containing 1C |  |  |
|  | -3.4 | selectin, endothelial cell |  |  |
|  | -3.4 | ankyrin repeat domain 65 |  |  |
|  | -3.4 | 4-aminobutyrate aminotransferase |  |  |
|  | -3.4 | membrane associated ring-CH-type finger 3 |  |  |
|  | -3.4 | leucine rich repeat containing 36 |  |  |
|  | -3.4 | methionine aminopeptidase 2 pseudogene |  |  |
|  | -3.4 | tetratricopeptide repeat domain 39A |  |  |
|  | -3.4 | transglutaminase 3, E polypeptide |  |  |
|  | -3.4 | neural retina leucine zipper gene |  |  |
|  | -3.4 | predicted gene, 23451 |  |  |
|  | -3.4 | neurexophilin and PC-esterase domain family, member 4 |  |  |
|  | -3.4 | CD160 antigen |  |  |
|  | -3.4 | glycophorin C |  |  |
|  | -3.4 | sodium channel, type IV, beta |  |  |
|  | -3.4 | otoferlin |  |  |
|  | -3.4 | family with sequence similarity 210, member B |  |  |
|  | -3.4 | macrophage stimulating 1 (hepatocyte growth factor-like) |  |  |
|  | -3.4 | chordin-like 1 |  |  |
|  | -3.4 | X-linked Kx blood group |  |  |
|  | -3.3 | dual-specificity tyrosine-(Y)-phosphorylation regulated kinase 3 |  |  |
|  | -3.3 | cardiomyopathy associated 5 |  |  |
|  | -3.3 | brain expressed X-linked 4 |  |  |
|  | -3.3 | EF hand domain containing 1 |  |  |
|  | -3.3 | transmembrane and coiled-coil domains 2 |  |  |
|  | -3.3 | eosinophil-associated, ribonuclease A family, member 1 |  |  |
|  | -3.3 | fibulin 5 |  |  |
|  | -3.3 | uroporphyrinogen decarboxylase |  |  |
|  | -3.3 | cerebellar degeneration-related 2 |  |  |
|  | -3.3 | ST3 beta-galactoside alpha-2,3-sialyltransferase 5 |  |  |
|  | -3.3 | signal peptide, CUB domain, EGF-like 3 |  |  |
|  | -3.3 | family with sequence similarity 169, member A |  |  |
|  | -3.3 | T cell receptor beta, variable 13-1 |  |  |
|  | -3.3 | V-set and transmembrane domain containing 4 |  |  |
|  | -3.3 | transferrin receptor 2 |  |  |
|  | -3.3 | T cell receptor beta joining 2-7 |  |  |
|  | -3.3 | orosomucoid 1 |  |  |
|  | -3.2 | cytochrome P450, family 4, subfamily f, polypeptide 39 |  |  |
|  | -3.2 | cytidine monophosphate (UMP-CMP) kinase 2, mitochondrial |  |  |
|  | -3.2 | CD59a antigen |  |  |
|  | -3.2 | flavin containing monooxygenase 1 |  |  |
|  | -3.2 | phosphatidylserine synthase 2 |  |  |
|  | -3.2 | potassium voltage-gated channel, subfamily G, member 1 |  |  |
|  | -3.2 | solute carrier family 22 (organic cation transporter), member 4 |  |  |
|  | -3.2 | transmembrane protein 255A |  |  |
|  | -3.2 | predicted gene 10371 |  |  |
|  | -3.2 | myosin, light polypeptide 9, regulatory |  |  |
|  | -3.2 | spire type actin nucleation factor 1 |  |  |
|  | -3.2 | olfactory receptor family 52 subfamily A member 5 |  |  |
|  | -3.2 | fructose bisphosphatase 1 |  |  |
|  | -3.2 | killer cell lectin-like receptor subfamily A, member 13, pseudogene |  |  |
|  | -3.2 | G-protein signalling modulator 2 (AGS3-like, C. elegans) |  |  |
|  | -3.2 | cathepsin F |  |  |
|  | -3.2 | glutathione S-transferase, mu 5 |  |  |
|  | -3.2 | microRNA 7026 |  |  |
|  | -3.2 | Indian hedgehog |  |  |
|  | -3.2 | H1.3 linker histone, cluster member |  |  |
|  | -3.2 | killer cell lectin-like receptor subfamily B member 1C |  |  |
|  | -3.2 | sterile alpha motif domain containing 3 |  |  |
|  | -3.2 | adenylate cyclase activating polypeptide 1 receptor 1 |  |  |
|  | -3.2 | dual specificity phosphatase 1 |  |  |
|  | -3.2 | H1.1 linker histone, cluster member |  |  |
|  | -3.2 | dual specificity phosphatase 8 |  |  |
|  | -3.2 | dipeptidase 1 |  |  |
|  | -3.2 | calpain 5 |  |  |
|  | -3.1 | REC114 meiotic recombination protein |  |  |
|  | -3.1 | ficolin A |  |  |
|  | -3.1 | mitochondrial amidoxime reducing component 1 |  |  |
|  | -3.1 | target of myb1-like 1 (chicken) |  |  |
|  | -3.1 | radical S-adenosyl methionine domain containing 2 |  |  |
|  | -3.1 | C1q and tumor necrosis factor related 12 |  |  |
|  | -3.1 | protein phosphatase 1 (formerly 2C)-like |  |  |
|  | -3.1 | 3-hydroxy-3-methylglutaryl-Coenzyme A synthase 2 |  |  |
|  | -3.1 | potassium channel tetramerisation domain containing 14 |  |  |
|  | -3.1 | terminal nucleotidyltransferase 5C |  |  |
|  | -3.1 | thymocyte selection-associated high mobility group box |  |  |
|  | -3.1 | pregnancy-associated plasma protein A |  |  |
|  | -3.1 | C-type lectin domain family 4, member g |  |  |
|  | -3.1 | forkhead box H1 |  |  |
|  | -3.1 | zinc finger, FYVE domain containing 28 |  |  |
|  | -3.1 | ankyrin repeat and SOCS box-containing 1 |  |  |
|  | -3.1 | origin recognition complex, subunit 1 |  |  |
|  | -3.1 | RAN binding protein 10 |  |  |
|  | -3.1 | eosinophil-associated, ribonuclease A family, pseudogene 12 |  |  |
|  | -3.1 | ureidopropionase, beta |  |  |
|  | -3.1 | BCLl2-like 15 |  |  |
|  | -3.1 | predicted gene, 32803 |  |  |
|  | -3.1 | leucine-rich repeats and immunoglobulin-like domains 1 |  |  |
|  | -3.1 | ubiquitin specific protease 51 |  |  |
|  | -3.1 | trafficking protein, kinesin binding 2 |  |  |
|  | -3.1 | RIKEN cDNA 4933440M02 gene |  |  |
|  | -3.0 | solute carrier family 22 (organic cation transporter), member 4 pseudogene |  |  |
|  | -3.0 | zinc finger protein, multitype 1 |  |  |
|  | -3.0 | zonadhesin |  |  |
|  | -3.0 | ring finger protein 123 |  |  |
|  | -3.0 | prokineticin 2 |  |  |
|  | -3.0 | N-acetylglutamate synthase |  |  |
|  | -3.0 | peroxiredoxin 2 |  |  |
|  | -3.0 | meiosis-specific nuclear structural protein 1 |  |  |
|  | -3.0 | DnaJ heat shock protein family (Hsp40) member B2 |  |  |
|  | -3.0 | Rho guanine nucleotide exchange factor (GEF) 12 |  |  |
|  | -3.0 | matrix metallopeptidase 28 (epilysin) |  |  |
|  | -3.0 | RIKEN cDNA 6030468B19 gene |  |  |
|  | -3.0 | growth factor receptor bound protein 14 |  |  |
|  | -3.0 | non imprinted in Prader-Willi/Angelman syndrome 1 homolog (human) |  |  |
|  | -3.0 | polypeptide N-acetylgalactosaminyltransferase 9 |  |  |
|  | -3.0 | olfactomedin-like 2A |  |  |
|  | -3.0 | R-spondin 1 |  |  |
|  | -3.0 | small integral membrane protein 41 |  |  |
|  | -3.0 | heparan sulfate 6-O-sulfotransferase 1 |  |  |
|  | -3.0 | START domain containing 10 |  |  |
|  | -3.0 | microRNA 8116 |  |  |
|  | -3.0 | transmembrane protein 184a |  |  |
|  | -3.0 | predicted gene 6653 |  |  |
|  | -2.9 | glycoprotein m6a |  |  |
|  | -2.9 | cytoplasmic polyadenylation element binding protein 3 |  |  |
|  | -2.9 | HEPACAM family member 2 |  |  |
|  | -2.9 | phosphate cytidylyltransferase 1, choline, beta isoform |  |  |
|  | -2.9 | predicted gene, 33111 |  |  |
|  | -2.9 | eosinophil-associated, ribonuclease A family, member 10 |  |  |
|  | -2.9 | intraflagellar transport 140 |  |  |
|  | -2.9 | immunoglobulin kappa variable 9-123 |  |  |
|  | -2.9 | antigen identified by monoclonal antibody Ki 67 |  |  |
|  | -2.9 | dishevelled associated activator of morphogenesis 2 |  |  |
|  | -2.9 | capping protein inhibiting regulator of actin |  |  |
|  | -2.9 | intercellular adhesion molecule 5, telencephalin |  |  |
|  | -2.9 | tetraspanin 15 |  |  |
|  | -2.9 | HEAT repeat containing 9 |  |  |
|  | -2.9 | complement component 1, s subcomponent 2 |  |  |
|  | -2.9 | secernin 3 |  |  |
|  | -2.9 | Max dimerization protein 3 |  |  |
|  | -2.9 | phosphatidylinositol glycan anchor biosynthesis, class Q |  |  |
|  | -2.9 | nuclear factor of kappa light polypeptide gene enhancer in B cells inhibitor, zeta |  |  |
|  | -2.9 | HECT domain E3 ubiquitin protein ligase 4 |  |  |
|  | -2.9 | eosinophil peroxidase |  |  |
|  | -2.9 | cathelicidin antimicrobial peptide |  |  |
|  | -2.9 | insulin-like growth factor 2 |  |  |
|  | -2.9 | serine (or cysteine) peptidase inhibitor, clade G, member 1 |  |  |
|  | -2.9 | tripartite motif-containing 2 |  |  |
|  | -2.9 | PDZK1 interacting protein 1 |  |  |
|  | -2.9 | family with sequence similarity 117, member A |  |  |
|  | -2.9 | vitrin |  |  |
|  | -2.9 | alcohol dehydrogenase 1 (class I) |  |  |
|  | -2.9 | stabilin 2 |  |  |
|  | -2.9 | Kruppel-like factor 11 |  |  |
|  | -2.8 | eosinophil-associated, ribonuclease A family, member 2 |  |  |
|  | -2.8 | ephrin B3 |  |  |
|  | -2.8 | tripartite motif-containing 59 |  |  |
|  | -2.8 | mitogen-activated protein kinase kinase kinase kinase 5 |  |  |
|  | -2.8 | arylsulfatase i |  |  |
|  | -2.8 | membrane frizzled-related protein |  |  |
|  | -2.8 | sulfotransferase family 1A, phenol-preferring, member 1 |  |  |
|  | -2.8 | predicted gene 16283 |  |  |
|  | -2.8 | family with sequence similarity 78, member A |  |  |
|  | -2.8 | prolactin receptor |  |  |
|  | -2.8 | predicted gene, 25788 |  |  |
|  | -2.8 | RIKEN cDNA 3110040M04 gene |  |  |
|  | -2.8 | solute carrier family 16 (monocarboxylic acid transporters), member 1 |  |  |
|  | -2.8 | phospholipase A and acyltransferase 3 |  |  |
|  | -2.8 | RIKEN cDNA 4933431E20 gene |  |  |
|  | -2.8 | bromodomain and PHD finger containing, 3 |  |  |
|  | -2.8 | ATP-binding cassette, sub-family B (MDR/TAP), member 6 |  |  |
|  | -2.8 | cell division cycle 6 |  |  |
|  | -2.8 | death-associated protein kinase 2 |  |  |
|  | -2.8 | kininogen 2 |  |  |
|  | -2.8 | ubiquitin-conjugating enzyme E2O |  |  |
|  | -2.8 | ST3 beta-galactoside alpha-2,3-sialyltransferase 6 |  |  |
|  | -2.8 | SH2 domain containing 1A |  |  |
|  | -2.8 | Scm polycomb group protein like 2 |  |  |
|  | -2.8 | glutaredoxin 5 |  |  |
|  | -2.8 | atlastin GTPase 1 |  |  |
|  | -2.8 | bone gamma carboxyglutamate protein |  |  |
|  | -2.8 | immunoglobulin joining chain |  |  |
|  | -2.8 | prokineticin receptor 1 |  |  |
|  | -2.8 | heat shock protein 1A |  |  |
|  | -2.8 | tumor necrosis factor (ligand) superfamily, member 13b |  |  |
|  | -2.8 | centrosomal protein 76 |  |  |
|  | -2.8 | apolipoprotein L domain containing 1 |  |  |
|  | -2.8 | sterile alpha motif domain containing 14 |  |  |
|  | -2.8 | immunoglobulin heavy variable V1-42 |  |  |
|  | -2.7 | adrenergic receptor, beta 1 |  |  |
|  | -2.7 | heat shock factor binding protein 1-like 1 |  |  |
|  | -2.7 | cerebral cavernous malformation 2-like |  |  |
|  | -2.7 | polypeptide N-acetylgalactosaminyltransferase 15 |  |  |
|  | -2.7 | signal peptide peptidase like 2B |  |  |
|  | -2.7 | early B cell factor 3 |  |  |
|  | -2.7 | transmembrane protein 178 |  |  |
|  | -2.7 | ADP-ribosyltransferase 3 |  |  |
|  | -2.7 | carboxymethylenebutenolidase-like (Pseudomonas) |  |  |
|  | -2.7 | H3 clustered histone 4 |  |  |
|  | -2.7 | CD59b antigen |  |  |
|  | -2.7 | formin 2 |  |  |
|  | -2.7 | chemokine (C-C motif) ligand 5 |  |  |
|  | -2.7 | sperm antigen with calponin homology and coiled-coil domains 1 |  |  |
|  | -2.7 | glial cell line derived neurotrophic factor family receptor alpha 1 |  |  |
|  | -2.7 | neuronal PAS domain protein 4 |  |  |
|  | -2.7 | glutamate-ammonia ligase (glutamine synthetase) pseudogene |  |  |
|  | -2.7 | nuclear receptor interacting protein 3 |  |  |
|  | -2.7 | NAC alpha domain containing |  |  |
|  | -2.7 | T cell receptor beta joining 1-2 |  |  |
|  | -2.7 | meiosis 1 associated protein |  |  |
|  | -2.7 | OTU domain, ubiquitin aldehyde binding 2 |  |  |
|  | -2.7 | receptor accessory protein 6 |  |  |
|  | -2.7 | lymphocyte antigen 6 complex, locus G |  |  |
|  | -2.7 | Bloodlinc, erythroid developmental long intergenic non-protein coding transcript |  |  |
|  | -2.7 | centromere protein F |  |  |
|  | -2.7 | sorbin and SH3 domain containing 1 |  |  |
|  | -2.7 | DEP domain containing 1a |  |  |
|  | -2.7 | TSC22 domain family, member 3 |  |  |
|  | -2.7 | hepsin |  |  |
|  | -2.7 | STE20-related kinase adaptor beta |  |  |
|  | -2.7 | PMS1 homolog2, mismatch repair system component |  |  |
|  | -2.7 | pyridoxine 5'-phosphate oxidase |  |  |
|  | -2.7 | heat shock protein 1B |  |  |
|  | -2.7 | von Willebrand factor C and EGF domains |  |  |
|  | -2.7 | REC8 meiotic recombination protein |  |  |
|  | -2.6 | RIKEN cDNA 2500002B13 gene |  |  |
|  | -2.6 | killer cell lectin-like receptor family I member 2 |  |  |
|  | -2.6 | expressed sequence AA986860 |  |  |
|  | -2.6 | Sh3 domain YSC-like 1 |  |  |
|  | -2.6 | predicted gene 12579 |  |  |
|  | -2.6 | jun proto-oncogene |  |  |
|  | -2.6 | cytochrome c oxidase subunit 6B2 |  |  |
|  | -2.6 | endothelial cell-specific molecule 1 |  |  |
|  | -2.6 | crystallin, beta A4 |  |  |
|  | -2.6 | sphingosine kinase 1 |  |  |
|  | -2.6 | myosin light chain, phosphorylatable, fast skeletal muscle |  |  |
|  | -2.6 | solute carrier family 1 (glial high affinity glutamate transporter), member 3 |  |  |
|  | -2.6 | H4 clustered histone 14 |  |  |
|  | -2.6 | H2B clustered histone 8 |  |  |
|  | -2.6 | arrestin domain containing 3 |  |  |
|  | -2.6 | fatty acyl CoA reductase 2 |  |  |
|  | -2.6 | regulator of G-protein signaling 5 |  |  |
|  | -2.6 | CD5 antigen-like |  |  |
|  | -2.6 | endonuclease domain containing 1 |  |  |
|  | -2.6 | 5'-nucleotidase, cytosolic III |  |  |
|  | -2.6 | H2B clustered histone 3 |  |  |
|  | -2.6 | immunoglobulin-like domain containing receptor 2 |  |  |
|  | -2.6 | calmodulin regulated spectrin-associated protein family, member 3 |  |  |
|  | -2.6 | cell division cycle 25B |  |  |
|  | -2.6 | nuclear GTPase, germinal center associated |  |  |
|  | -2.6 | solute carrier family 43, member 3 |  |  |
|  | -2.6 | aldehyde dehydrogenase 3 family, member B2 |  |  |
|  | -2.6 | interferon-related developmental regulator 2 |  |  |
|  | -2.6 | MFSD2 lysolipid transporter B, sphingolipid |  |  |
|  | -2.6 | immunoglobulin kappa chain variable 2-137 |  |  |
|  | -2.6 | transmembrane epididymal family member 2 |  |  |
|  | -2.6 | E2F transcription factor 2 |  |  |
|  | -2.6 | Spi-C transcription factor (Spi-1/PU.1 related) |  |  |
|  | -2.6 | protoporphyrinogen oxidase |  |  |
|  | -2.6 | septin 4 |  |  |
|  | -2.6 | coiled-coil domain containing 96 |  |  |
|  | -2.6 | sperm associated antigen 5 |  |  |
|  | -2.6 | killer cell lectin-like receptor, subfamily A, member 17 |  |  |
|  | -2.6 | death associated protein-like 1 |  |  |
|  | -2.6 | interferon gamma |  |  |
|  | -2.6 | GTP cyclohydrolase 1 |  |  |
|  | -2.5 | predicted gene, 23650 |  |  |
|  | -2.5 | coiled-coil domain containing 68 |  |  |
|  | -2.5 | carbohydrate sulfotransferase 10 |  |  |
|  | -2.5 | regulator of G-protein signaling 12 |  |  |
|  | -2.5 | IBA57 homolog, iron-sulfur cluster assembly |  |  |
|  | -2.5 | growth factor independent 1B |  |  |
|  | -2.5 | H3 clustered histone 8 |  |  |
|  | -2.5 | DnaJ heat shock protein family (Hsp40) member B4 |  |  |
|  | -2.5 | ependymin related protein 1 (zebrafish) |  |  |
|  | -2.5 | vesicular, overexpressed in cancer, prosurvival protein 1 |  |  |
|  | -2.5 | PH domain containing endocytic trafficking adaptor 2 |  |  |
|  | -2.5 | ChaC, cation transport regulator 2 |  |  |
|  | -2.5 | coagulation factor VIII |  |  |
|  | -2.5 | transmembrane protein 45A2 |  |  |
|  | -2.5 | microRNA 7048 |  |  |
|  | -2.5 | rad and gem related GTP binding protein 1 |  |  |
|  | -2.5 | RIKEN cDNA 1700001K23 gene |  |  |
|  | -2.5 | ADP-ribosyltransferase 2b |  |  |
|  | -2.5 | myosin IA |  |  |
|  | -2.5 | coxsackie virus and adenovirus receptor |  |  |
|  | -2.5 | proteoglycan 2, bone marrow |  |  |
|  | -2.5 | acetylcholinesterase |  |  |
|  | -2.5 | kinesin family member 18A |  |  |
|  | -2.5 | ankyrin repeat and LEM domain containing 1 |  |  |
|  | -2.5 | EGF-like-domain, multiple 6 |  |  |
|  | -2.5 | Rho GTPase activating protein 23 |  |  |
|  | -2.5 | dystrophia myotonica-containing WD repeat motif |  |  |
|  | -2.5 | carbonic anhydrase 3 |  |  |
|  | -2.5 | SHC (Src homology 2 domain containing) family, member 4 |  |  |
|  | -2.5 | extracellular matrix protein 2, female organ and adipocyte specific |  |  |
|  | -2.5 | HECT domain and ankyrin repeat containing, E3 ubiquitin protein ligase 1 |  |  |
|  | -2.5 | nephronectin |  |  |
|  | -2.5 | frizzled class receptor 9 |  |  |
|  | -2.5 | small integral membrane protein 6 |  |  |
|  | -2.5 | transmembrane protein 44 |  |  |
|  | -2.5 | CD24a antigen |  |  |
|  | -2.5 | tumor necrosis factor receptor superfamily, member 19 |  |  |
|  | -2.5 | protein tyrosine phosphatase, receptor type, B |  |  |
|  | -2.4 | proteoglycan 3 |  |  |
|  | -2.4 | syntaxin binding protein 4 |  |  |
|  | -2.4 | predicted gene, 22806 |  |  |
|  | -2.4 | biliverdin reductase B (flavin reductase (NADPH)) |  |  |
|  | -2.4 | RIKEN cDNA 9830166K06 gene |  |  |
|  | -2.4 | kinesin family member 18B |  |  |
|  | -2.4 | lymphatic vessel endothelial hyaluronan receptor 1 |  |  |
|  | -2.4 | arylformamidase |  |  |
|  | -2.4 | ribonucleotide reductase M2 |  |  |
|  | -2.4 | SEC14-like lipid binding 5 |  |  |
|  | -2.4 | killer cell lectin-like receptor subfamily A, member 9 |  |  |
|  | -2.4 | glutamate receptor, metabotropic 8 |  |  |
|  | -2.4 | sterile alpha motif domain containing 4 |  |  |
|  | -2.4 | dynein regulatory complex subunit 7 |  |  |
|  | -2.4 | adrenergic receptor, beta 2 |  |  |
|  | -2.4 | predicted gene, 40617 |  |  |
|  | -2.4 | membrane associated ring-CH-type finger 8 |  |  |
|  | -2.4 | solute carrier family 25, member 38 |  |  |
|  | -2.4 | thioredoxin reductase 2 |  |  |
|  | -2.4 | centriolar coiled coil protein 110 |  |  |
|  | -2.4 | RIKEN cDNA G430095P16 gene |  |  |
|  | -2.4 | adenylate cyclase 10 |  |  |
|  | -2.4 | kelch-like 12 |  |  |
|  | -2.4 | uroplakin 1A |  |  |
|  | -2.4 | ubiquitin-conjugating enzyme E2C |  |  |
|  | -2.4 | tubulin polymerization promoting protein |  |  |
|  | -2.4 | zinc finger, FYVE domain containing 9 |  |  |
|  | -2.4 | RNA binding motif protein 24 |  |  |
|  | -2.4 | immunoglobulin heavy constant gamma 2B |  |  |
|  | -2.4 | amine oxidase, copper containing 3 |  |  |
|  | -2.4 | stomatin |  |  |
|  | -2.4 | centromere protein K pseudogene |  |  |
|  | -2.4 | neuronal tyrosine-phosphorylated phosphoinositide 3-kinase adaptor 1 |  |  |
|  | -2.4 | LIM homeobox protein 1 |  |  |
|  | -2.4 | regulator of G-protein signalling 9 binding protein |  |  |
|  | -2.4 | microRNA 6953 |  |  |
|  | -2.4 | cyclin E1 |  |  |
|  | -2.4 | CD69 antigen |  |  |
|  | -2.4 | netrin 4 |  |  |
|  | -2.4 | transmembrane protein 231 |  |  |
|  | -2.4 | WD repeat domain 31 |  |  |
|  | -2.4 | thyroid stimulating hormone receptor |  |  |
|  | -2.4 | shroom family member 3 |  |  |
|  | -2.4 | predicted gene 6377 |  |  |
|  | -2.3 | pregnancy upregulated non-ubiquitously expressed CaM kinase |  |  |
|  | -2.3 | mitochondrial fission regulator 1 |  |  |
|  | -2.3 | phosphatidylinositol-4-phosphate 5-kinase, type 1 beta |  |  |
|  | -2.3 | abnormal spindle microtubule assembly |  |  |
|  | -2.3 | nuclear factor I/A |  |  |
|  | -2.3 | grancalcin |  |  |
|  | -2.3 | neuraminidase 3 |  |  |
|  | -2.3 | transmembrane protein 120B |  |  |
|  | -2.3 | nocturnin |  |  |
|  | -2.3 | ATP-binding cassette, sub-family A (ABC1), member 6 |  |  |
|  | -2.3 | RAD23 homolog A, nucleotide excision repair protein |  |  |
|  | -2.3 | membrane associated ring-CH-type finger 2 |  |  |
|  | -2.3 | salt inducible kinase 1 |  |  |
|  | -2.3 | predicted gene 550 |  |  |
|  | -2.3 | tubulointerstitial nephritis antigen-like 1 |  |  |
|  | -2.3 | microtubule associated monooxygenase, calponin and LIM domain containing 3 |  |  |
|  | -2.3 | RNA binding motif protein 38 |  |  |
|  | -2.3 | microRNA 27a |  |  |
|  | -2.3 | polymerase (DNA directed), theta |  |  |
|  | -2.3 | CD4 antigen |  |  |
|  | -2.3 | regulator of G-protein signaling 2 |  |  |
|  | -2.3 | RIKEN cDNA 1700034H15 gene |  |  |
|  | -2.3 | fumarylacetoacetate hydrolase domain containing 1 |  |  |
|  | -2.3 | predicted gene 8251 |  |  |
|  | -2.3 | H3 clustered histone 11 |  |  |
|  | -2.3 | fibulin 1 |  |  |
|  | -2.3 | microRNA 5134 |  |  |
|  | -2.3 | erythrocyte membrane protein band 4.1 |  |  |
|  | -2.3 | glutathione S-transferase, alpha 4 |  |  |
|  | -2.3 | H3 clustered histone 2 |  |  |
|  | -2.3 | synovial sarcoma, X 2 interacting protein |  |  |
|  | -2.3 | aminolevulinic acid synthase 2, erythroid |  |  |
|  | -2.3 | synuclein, alpha |  |  |
|  | -2.3 | BRD8 domain containing |  |  |
|  | -2.3 | hydroxyacyl glutathione hydrolase |  |  |
|  | -2.3 | immunoglobulin heavy variable 1-31 |  |  |
|  | -2.3 | H2B clustered histone 9 |  |  |
|  | -2.3 | nucleolar and spindle associated protein 1 |  |  |
|  | -2.3 | fizzy and cell division cycle 20 related 1 |  |  |
|  | -2.3 | NAD kinase 2, mitochondrial |  |  |
|  | -2.3 | reelin |  |  |
|  | -2.3 | 2,3-bisphosphoglycerate mutase |  |  |
|  | -2.3 | multimerin 2 |  |  |
|  | -2.3 | F-box protein 30 |  |  |
|  | -2.3 | RIKEN cDNA 4930558J18 gene |  |  |
|  | -2.3 | tumor necrosis factor receptor superfamily, member 17 |  |  |
|  | -2.3 | beta-transducin repeat containing protein |  |  |
|  | -2.3 | Kv channel interacting protein 3, calsenilin |  |  |
|  | -2.3 | cadherin-related family member 4 |  |  |
|  | -2.3 | immunoglobulin kappa variable 4-78 |  |  |
|  | -2.3 | autophagy related 4A, cysteine peptidase |  |  |
|  | -2.3 | cytotoxic T-lymphocyte-associated protein 4 |  |  |
|  | -2.2 | DEP domain containing 1B |  |  |
|  | -2.2 | adhesion G protein-coupled receptor D1 |  |  |
|  | -2.2 | GATA binding protein 1 |  |  |
|  | -2.2 | predicted gene, 42067 |  |  |
|  | -2.2 | collagen, type II, alpha 1 |  |  |
|  | -2.2 | vascular cell adhesion molecule 1 |  |  |
|  | -2.2 | potassium intermediate/small conductance calcium-activated channel, subfamily N, member 1 |  |  |
|  | -2.2 | TLC domain containing 5 |  |  |
|  | -2.2 | desmoplakin |  |  |
|  | -2.2 | GIPC PDZ domain containing family, member 2 |  |  |
|  | -2.2 | dishevelled-binding antagonist of beta-catenin 1 |  |  |
|  | -2.2 | copine family member IX |  |  |
|  | -2.2 | doublecortin-like kinase 2 |  |  |
|  | -2.2 | glutathione S-transferase pi 3 |  |  |
|  | -2.2 | immunoglobulin kappa chain variable 12-89 |  |  |
|  | -2.2 | phospholipid scramblase 4 |  |  |
|  | -2.2 | RIKEN cDNA A530013C23 gene |  |  |
|  | -2.2 | kinesin family member 14 |  |  |
|  | -2.2 | establishment of sister chromatid cohesion N-acetyltransferase 2 |  |  |
|  | -2.2 | immunoglobulin heavy variable 7-3 |  |  |
|  | -2.2 | PH domain and leucine rich repeat protein phosphatase 2 |  |  |
|  | -2.2 | hemoglobin alpha, adult chain 1 |  |  |
|  | -2.2 | ISG15 ubiquitin-like modifier |  |  |
|  | -2.2 | H1.2 linker histone, cluster member |  |  |
|  | -2.2 | predicted gene 9895 |  |  |
|  | -2.2 | immunoglobulin kappa variable 4-80 |  |  |
|  | -2.2 | killer cell lectin-like receptor subfamily C, member 2 |  |  |
|  | -2.2 | H2B clustered histone 14 |  |  |
|  | -2.2 | H2A clustered histone 25 |  |  |
|  | -2.2 | killer cell lectin-like receptor subfamily C, member 1 |  |  |
|  | -2.2 | cyclin dependent kinase inhibitor 2C |  |  |
|  | -2.2 | ornithine decarboxylase, structural 1 |  |  |
|  | -2.2 | ubiquitin-conjugating enzyme E2L 6 |  |  |
|  | -2.2 | H3 clustered histone 10 |  |  |
|  | -2.2 | doublesex and mab-3 related transcription factor 3 |  |  |
|  | -2.2 | hemoglobin alpha, adult chain 2 |  |  |
|  | -2.2 | predicted gene, 32511 |  |  |
|  | -2.2 | pleckstrin and Sec7 domain containing |  |  |
|  | -2.2 | regulator of cell cycle |  |  |
|  | -2.2 | immunoglobulin heavy variable 3-5 |  |  |
|  | -2.2 | sushi domain containing 2 |  |  |
|  | -2.2 | fibroblast growth factor 1 |  |  |
|  | -2.2 | protein regulator of cytokinesis 1 |  |  |
|  | -2.2 | ankyrin repeat domain 6 |  |  |
|  | -2.2 | MICAL-like 2 |  |  |
|  | -2.2 | agrin |  |  |
|  | -2.2 | heat shock protein 1-like |  |  |
|  | -2.2 | nuclear receptor coactivator 7 |  |  |
|  | -2.2 | iron-sulfur cluster assembly 1 |  |  |
|  | -2.2 | #NV |  |  |
|  | -2.2 | predicted gene 13306 |  |  |
|  | -2.2 | radial spoke head 9 homolog (Chlamydomonas) |  |  |
|  | -2.2 | RIKEN cDNA 4930550C14 gene |  |  |
|  | -2.2 | immunoglobulin heavy constant gamma 2C |  |  |
|  | -2.2 | rabphilin 3A |  |  |
|  | -2.2 | ankyrin repeat domain 9 |  |  |
|  | -2.2 | polypeptide N-acetylgalactosaminyltransferase 10 |  |  |
|  | -2.2 | Rhesus blood group-associated C glycoprotein |  |  |
|  | -2.2 | hemoglobin, theta 1A |  |  |
|  | -2.2 | predicted gene, 34225 |  |  |
|  | -2.2 | cyclin A2 |  |  |
|  | -2.1 | immunoglobulin kappa variable 6-17 |  |  |
|  | -2.1 | triggering receptor expressed on myeloid cells-like 4 |  |  |
|  | -2.1 | glutathione peroxidase 1 |  |  |
|  | -2.1 | killer cell lectin-like receptor subfamily B member 1A |  |  |
|  | -2.1 | intraflagellar transport 80 |  |  |
|  | -2.1 | kinesin family member C1 |  |  |
|  | -2.1 | eukaryotic translation initiation factor 2 alpha kinase 1 |  |  |
|  | -2.1 | RAB30, member RAS oncogene family |  |  |
|  | -2.1 | E2F transcription factor 8 |  |  |
|  | -2.1 | recombination activating 1 |  |  |
|  | -2.1 | predicted gene, 19582 |  |  |
|  | -2.1 | Src homology 2 domain containing F |  |  |
|  | -2.1 | makorin, ring finger protein 1, pseudogene 1 |  |  |
|  | -2.1 | kelch domain containing 8B |  |  |
|  | -2.1 | DLG associated protein 5 |  |  |
|  | -2.1 | cilia and flagella associated protein 119 |  |  |
|  | -2.1 | H2B clustered histone 22 |  |  |
|  | -2.1 | cyclin E2 |  |  |
|  | -2.1 | H2B clustered histone 13 |  |  |
|  | -2.1 | nanos C2HC-type zinc finger 1 |  |  |
|  | -2.1 | nuclear receptor subfamily 4, group A, member 2 |  |  |
|  | -2.1 | H2A clustered histone 6 |  |  |
|  | -2.1 | kinesin family member C5B |  |  |
|  | -2.1 | myeloblastosis oncogene-like 2 |  |  |
|  | -2.1 | tau tubulin kinase 1 |  |  |
|  | -2.1 | predicted gene 16548 |  |  |
|  | -2.1 | forkhead box O3 |  |  |
|  | -2.1 | predicted gene, 24119 |  |  |
|  | -2.1 | MAS-related GPR, member A2A |  |  |
|  | -2.1 | threonyl-tRNA synthetase-like 2 |  |  |
|  | -2.1 | makorin, ring finger protein, 1 |  |  |
|  | -2.1 | tyrosine kinase, non-receptor, 2 |  |  |
|  | -2.1 | LIM domain only 1 |  |  |
|  | -2.1 | integrin alpha 4 |  |  |
|  | -2.1 | protein phosphatase 1, regulatory subunit 15A |  |  |
|  | -2.1 | diaphanous related formin 3 |  |  |
|  | -2.1 | protein phosphatase 1, regulatory subunit 42 |  |  |
|  | -2.1 | citron |  |  |
|  | -2.1 | baculoviral IAP repeat-containing 5 |  |  |
|  | -2.1 | H2A clustered histone 12 |  |  |
|  | -2.1 | toll-like receptor 11 |  |  |
|  | -2.1 | cDNA sequence AK157302 |  |  |
|  | -2.1 | kinetochore scaffold 1 |  |  |
|  | -2.1 | apelin receptor |  |  |
|  | -2.1 | extra spindle pole bodies 1, separase |  |  |
|  | -2.1 | fibronectin type III and SPRY domain containing 1-like |  |  |
|  | -2.1 | lactase |  |  |
|  | -2.1 | Rho guanine nucleotide exchange factor (GEF) 39 |  |  |
|  | -2.1 | acyl-CoA synthetase long-chain family member 1 |  |  |
|  | -2.1 | NK2 homeobox 3 |  |  |
|  | -2.1 | family with sequence similarity 83, member D |  |  |
|  | -2.1 | ADAMTS-like 3 |  |  |
|  | -2.1 | cytoplasmic polyadenylation element binding protein 4 |  |  |
|  | -2.1 | solute carrier family 25 (mitochondrial carrier, ornithine transporter) member 2 |  |  |
|  | -2.1 | cyclin B2 |  |  |
|  | -2.1 | mucin 6, gastric |  |  |
|  | -2.1 | N-acetylated alpha-linked acidic dipeptidase-like 1 |  |  |
|  | -2.1 | H3 clustered histone 1 |  |  |
|  | -2.1 | ras homolog family member B |  |  |
|  | -2.1 | cell division cycle 25C |  |  |
|  | -2.1 | #NV |  |  |
|  | -2.1 | H2B clustered histone 7 |  |  |
|  | -2.1 | AXL receptor tyrosine kinase |  |  |
|  | -2.1 | unc-5 family C-terminal like |  |  |
|  | -2.1 | H2A clustered histone 22 |  |  |
|  | -2.1 | cytoskeleton associated protein 2 |  |  |
|  | -2.1 | Rho guanine nucleotide exchange factor (GEF) 26 |  |  |
|  | -2.1 | dynein, axonemal, heavy chain 6 |  |  |
|  | -2.1 | golgi membrane protein 1 |  |  |
|  | -2.0 | YOD1 deubiquitinase |  |  |
|  | -2.0 | thymidine kinase 1 |  |  |
|  | -2.0 | kinesin family member 4 |  |  |
|  | -2.0 | F-box protein 7 |  |  |
|  | -2.0 | lactotransferrin |  |  |
|  | -2.0 | zymogen granule protein 16 |  |  |
|  | -2.0 | high mobility group box 3 |  |  |
|  | -2.0 | non-SMC condensin II complex, subunit D3 |  |  |
|  | -2.0 | microRNA 23a |  |  |
|  | -2.0 | autophagy related 4A, pseudogene |  |  |
|  | -2.0 | H3 clustered histone 6 |  |  |
|  | -2.0 | lipase, member H |  |  |
|  | -2.0 | NIMA (never in mitosis gene a)-related expressed kinase 3 |  |  |
|  | -2.0 | poly(A) binding protein, cytoplasmic 4 |  |  |
|  | -2.0 | macrophage stimulating 1 receptor (c-met-related tyrosine kinase) |  |  |
|  | -2.0 | BCL2-related ovarian killer |  |  |
|  | -2.0 | family with sequence similarity 220, member A |  |  |
|  | -2.0 | v-myc avian myelocytomatosis viral oncogene lung carcinoma derived |  |  |
|  | -2.0 | H4 clustered histone 8 |  |  |
|  | -2.0 | immunoglobulin heavy variable 9-4 |  |  |
|  | -2.0 | BCL2-interacting killer |  |  |
|  | -2.0 | kinetochore associated 1 |  |  |
|  | -2.0 | microtubule associated tyrosine carboxypeptidase 2 |  |  |
|  | -2.0 | prominin 1 |  |  |
|  | -2.0 | claudin domain containing 2 |  |  |
|  | -2.0 | amine oxidase, copper containing 2 (retina-specific) |  |  |
|  | -2.0 | BRCA1 associated RING domain 1 |  |  |
|  | -2.0 | cytochrome P450, family 27, subfamily a, polypeptide 1 |  |  |
|  | -2.0 | microRNA 7052 |  |  |
|  | -2.0 | interferon-induced protein 44 |  |  |
|  | -2.0 | cell division control protein 6 homolog |  |  |
|  | -2.0 | lipin 2 |  |  |
|  | -2.0 | potassium channel tetramerisation domain containing 7 |  |  |
|  | -2.0 | TGF-beta activated kinase 1/MAP3K7 binding protein 3 |  |  |
|  | -2.0 | multiple inositol polyphosphate histidine phosphatase 1 |  |  |
|  | -2.0 | USH1 protein network component harmonin binding protein 1 |  |  |
|  | -2.0 | glutamate-cysteine ligase, catalytic subunit |  |  |
|  | -2.0 | peroxiredoxin like 2A |  |  |
|  | -2.0 | BCL2-like 1 |  |  |
|  | -2.0 | growth differentiation factor 3 |  |  |
|  | -2.0 | CD300 molecule like family member G |  |  |
|  | -2.0 | breast cancer 1, early onset |  |  |
|  | -2.0 | zinc finger protein 354B |  |  |
|  | -2.0 | trophinin associated protein |  |  |
|  | -2.0 | PIF1 5'-to-3' DNA helicase |  |  |
|  | -2.0 | predicted gene 10030 |  |  |
|  | -2.0 | denticleless E3 ubiquitin protein ligase |  |  |
|  | -2.0 | lipopolysaccharide binding protein |  |  |
|  | -2.0 | poly(A)-binding protein, cytoplasmic pseudogene |  |  |
|  | 2.0 | heparan sulfate 6-O-sulfotransferase 2 |  |  |
|  | 2.0 | predicted gene, 19557 |  |  |
|  | 2.0 | RIKEN cDNA F730043M19 gene |  |  |
|  | 2.0 | RIKEN cDNA 2610528J11 gene |  |  |
|  | 2.0 | eva-1 homolog B (C. elegans) |  |  |
|  | 2.0 | interleukin 11 receptor, alpha chain 1 |  |  |
|  | 2.0 | triosephosphate isomerase 1 |  |  |
|  | 2.0 | RIKEN cDNA 0610009E02 gene |  |  |
|  | 2.0 | ring finger protein 150 |  |  |
|  | 2.0 | glyceraldehyde-3-phosphate dehydrogenase pseudogene |  |  |
|  | 2.0 | coagulation factor II (thrombin) receptor-like 2 |  |  |
|  | 2.0 | protocadherin 19 |  |  |
|  | 2.0 | neuralized E3 ubiquitin protein ligase 2 |  |  |
|  | 2.0 | phospholipase A2, group VII (platelet-activating factor acetylhydrolase, plasma) |  |  |
|  | 2.0 | kinesin family member 3A |  |  |
|  | 2.0 | dynein light chain Tctex-type 4 |  |  |
|  | 2.0 | cytochrome P450, family 4, subfamily v, polypeptide 3 |  |  |
|  | 2.0 | RIKEN cDNA 1700022N22 gene |  |  |
|  | 2.0 | transmembrane protein 236 |  |  |
|  | 2.0 | RIKEN cDNA B930018H19 gene |  |  |
|  | 2.0 | interleukin 6 signal transducer |  |  |
|  | 2.0 | disco interacting protein 2 homolog C |  |  |
|  | 2.0 | IKAROS family zinc finger 4 |  |  |
|  | 2.0 | lipoma HMGIC fusion partner-like 2 |  |  |
|  | 2.0 | phospholipid phosphatase 2 |  |  |
|  | 2.0 | sorting nexin 2 pseudogene |  |  |
|  | 2.0 | predicted gene, 31763 |  |  |
|  | 2.0 | Rho GTPase activating protein 6 |  |  |
|  | 2.0 | sushi-repeat-containing protein, X-linked 2 |  |  |
|  | 2.1 | solute carrier family 25, member 43 |  |  |
|  | 2.1 | sperm flagellar 1 like |  |  |
|  | 2.1 | predicted gene, 16861 |  |  |
|  | 2.1 | staufen double-stranded RNA binding protein 2 |  |  |
|  | 2.1 | calreticulin 4 |  |  |
|  | 2.1 | transmembrane protein 51 |  |  |
|  | 2.1 | solute carrier family 2 (facilitated glucose transporter), member 1 |  |  |
|  | 2.1 | RIKEN cDNA 0610038B21 gene |  |  |
|  | 2.1 | growth differentiation factor 15 |  |  |
|  | 2.1 | T cell receptor gamma, constant 1 |  |  |
|  | 2.1 | collagen, type XV, alpha 1 |  |  |
|  | 2.1 | cathepsin D |  |  |
|  | 2.1 | transmembrane inner ear |  |  |
|  | 2.1 | Fc fragment of IgG receptor and transporter |  |  |
|  | 2.1 | SLAM family member 8 |  |  |
|  | 2.1 | carboxypeptidase X 1 (M14 family) |  |  |
|  | 2.1 | huntingtin interacting protein 1 |  |  |
|  | 2.1 | procollagen-proline, 2-oxoglutarate 4-dioxygenase (proline 4-hydroxylase), alpha 1 polypeptide |  |  |
|  | 2.1 | stearoyl-coenzyme A desaturase 3 |  |  |
|  | 2.1 | solute carrier family 15, member 3 |  |  |
|  | 2.1 | interleukin 11 receptor, alpha chain 2 |  |  |
|  | 2.1 | solute carrier family 2 (facilitated glucose transporter), member 6 |  |  |
|  | 2.1 | transmembrane and tetratricopeptide repeat containing 2 |  |  |
|  | 2.1 | integrin alpha 7 |  |  |
|  | 2.1 | CDGSH iron sulfur domain 3 |  |  |
|  | 2.1 | coagulation factor II (thrombin) receptor |  |  |
|  | 2.1 | myocardin related transcription factor B |  |  |
|  | 2.1 | hypoxia inducible lipid droplet associated |  |  |
|  | 2.1 | galactokinase 2 |  |  |
|  | 2.1 | linker for activation of T cells family, member 2 |  |  |
|  | 2.1 | tissue inhibitor of metalloproteinase 3 |  |  |
|  | 2.1 | LIF receptor alpha |  |  |
|  | 2.1 | chitinase 3-like 3 pseudogene |  |  |
|  | 2.1 | wolframin ER transmembrane glycoprotein |  |  |
|  | 2.1 | predicted gene 5150 |  |  |
|  | 2.1 | procollagen-lysine, 2-oxoglutarate 5-dioxygenase 1 |  |  |
|  | 2.1 | potassium inwardly-rectifying channel, subfamily K, member 6 |  |  |
|  | 2.1 | class II transactivator |  |  |
|  | 2.1 | procollagen lysine, 2-oxoglutarate 5-dioxygenase 2 |  |  |
|  | 2.1 | vasohibin 2 |  |  |
|  | 2.1 | glycine C-acetyltransferase (2-amino-3-ketobutyrate-coenzyme A ligase) |  |  |
|  | 2.1 | adhesion G protein-coupled receptor A2 |  |  |
|  | 2.1 | interleukin 11 receptor, alpha chain 2 |  |  |
|  | 2.1 | prostaglandin E synthase 3 like |  |  |
|  | 2.1 | olfactory receptor family 8 subfamily B member 53 |  |  |
|  | 2.1 | pellino 3 |  |  |
|  | 2.2 | poliovirus receptor |  |  |
|  | 2.2 | unc-5 netrin receptor B |  |  |
|  | 2.2 | multiple EGF-like-domains 10 |  |  |
|  | 2.2 | kazrin, periplakin interacting protein |  |  |
|  | 2.2 | Rous sarcoma oncogene |  |  |
|  | 2.2 | a disintegrin-like and metallopeptidase (reprolysin type) with thrombospondin type 1 motif, 12 |  |  |
|  | 2.2 | integrin beta 3 |  |  |
|  | 2.2 | chemokine (C-X-C motif) ligand 3 |  |  |
|  | 2.2 | POU domain, class 4, transcription factor 1 |  |  |
|  | 2.2 | vasohibin 1 |  |  |
|  | 2.2 | tubulin tyrosine ligase-like family, member 11 |  |  |
|  | 2.2 | jun proto-oncogene, opposite strand |  |  |
|  | 2.2 | RIKEN cDNA 2810414N06 gene |  |  |
|  | 2.2 | roundabout guidance receptor 3 |  |  |
|  | 2.2 | MORN repeat containing 4 |  |  |
|  | 2.2 | ring finger protein 217 |  |  |
|  | 2.2 | UDP-GlcNAc:betaGal beta-1,3-N-acetylglucosaminyltransferase 9 |  |  |
|  | 2.2 | brain abundant, membrane attached signal protein 1 |  |  |
|  | 2.2 | serine/threonine kinase-like domain containing 1 |  |  |
|  | 2.2 | milk fat globule EGF and factor V/VIII domain containing |  |  |
|  | 2.2 | heme oxygenase 1 |  |  |
|  | 2.2 | transforming growth factor, beta induced |  |  |
|  | 2.2 | sorting nexin 2 |  |  |
|  | 2.2 | RAB19, member RAS oncogene family |  |  |
|  | 2.2 | aryl-hydrocarbon receptor |  |  |
|  | 2.2 | phytanoyl-CoA dioxygenase domain containing 1 |  |  |
|  | 2.2 | dipeptidase 2 |  |  |
|  | 2.2 | MyoD family inhibitor domain containing |  |  |
|  | 2.2 | adhesion G protein-coupled receptor B1 |  |  |
|  | 2.2 | phospholipid transfer protein |  |  |
|  | 2.2 | colony stimulating factor 1 receptor |  |  |
|  | 2.2 | phospholipase C, delta 1 |  |  |
|  | 2.2 | transmembrane protein 202 |  |  |
|  | 2.2 | Rho GTPase activating protein 22 |  |  |
|  | 2.2 | epidermal growth factor-containing fibulin-like extracellular matrix protein 2 |  |  |
|  | 2.2 | hepatocyte growth factor |  |  |
|  | 2.2 | glyceraldehyde-3-phosphate dehydrogenase pseudogene |  |  |
|  | 2.2 | thyroid hormone receptor beta |  |  |
|  | 2.2 | cyclin-dependent kinase-like 2 (CDC2-related kinase) |  |  |
|  | 2.2 | zinc finger protein 750 |  |  |
|  | 2.2 | N-acetylglucosamine kinase |  |  |
|  | 2.2 | insulin-like growth factor 2 mRNA binding protein 2 |  |  |
|  | 2.2 | tumor necrosis factor receptor superfamily, member 26 |  |  |
|  | 2.2 | WD repeat domain 54 |  |  |
|  | 2.2 | C-type lectin domain family 4, member b2 |  |  |
|  | 2.2 | collagen, type V, alpha 2 |  |  |
|  | 2.2 | vasorin |  |  |
|  | 2.2 | interleukin 1 receptor antagonist |  |  |
|  | 2.2 | solute carrier family 9 (sodium/hydrogen exchanger), member 9 |  |  |
|  | 2.2 | epithelial cell adhesion molecule |  |  |
|  | 2.2 | NLR family, pyrin domain containing 10 |  |  |
|  | 2.3 | macrophage migration inhibitory factor (glycosylation-inhibiting factor) |  |  |
|  | 2.3 | hypermethylated in cancer 1 |  |  |
|  | 2.3 | paired related homeobox 1 |  |  |
|  | 2.3 | major facilitator superfamily domain containing 4B5 |  |  |
|  | 2.3 | NPC intracellular cholesterol transporter 1 |  |  |
|  | 2.3 | glutathione S-transferase, theta 3 |  |  |
|  | 2.3 | mucin 1, transmembrane |  |  |
|  | 2.3 | proliferation and apoptosis adaptor protein 15A |  |  |
|  | 2.3 | ectonucleotide pyrophosphatase/phosphodiesterase 1 |  |  |
|  | 2.3 | DIX domain containing 1 |  |  |
|  | 2.3 | microRNA 8098 |  |  |
|  | 2.3 | fibroblast growth factor receptor 1 |  |  |
|  | 2.3 | a disintegrin and metallopeptidase domain 11 |  |  |
|  | 2.3 | archaelysin family metallopeptidase 1 |  |  |
|  | 2.3 | myosin IE |  |  |
|  | 2.3 | SH3 and multiple ankyrin repeat domains 1 |  |  |
|  | 2.3 | insulinoma-associated 1 |  |  |
|  | 2.3 | basic leucine zipper transcription factor, ATF-like 2 |  |  |
|  | 2.3 | RIKEN cDNA A430088P11 gene |  |  |
|  | 2.3 | solute carrier family 38, member 7 |  |  |
|  | 2.3 | TBC1 domain family, member 4 |  |  |
|  | 2.3 | WT1 interacting protein |  |  |
|  | 2.3 | cysteine-rich transmembrane module containing 1 |  |  |
|  | 2.3 | growth arrest specific 6 |  |  |
|  | 2.3 | protein kinase domain containing, cytoplasmic |  |  |
|  | 2.3 | interleukin 4 |  |  |
|  | 2.3 | protein kinase C and casein kinase substrate in neurons 3 |  |  |
|  | 2.3 | dipeptidase 2 |  |  |
|  | 2.3 | SH3 domain containing ring finger 3 |  |  |
|  | 2.3 | solute carrier family 4 (anion exchanger), member 3 |  |  |
|  | 2.3 | histocompatibility 2, M region locus 2 |  |  |
|  | 2.3 | shroom family member 4 |  |  |
|  | 2.3 | fibroblast growth factor 11 |  |  |
|  | 2.3 | microtubule-associated protein 6 |  |  |
|  | 2.3 | RIKEN cDNA 2810429I04 gene |  |  |
|  | 2.3 | cytochrome P450, family 4, subfamily x, polypeptide 1 |  |  |
|  | 2.3 | integrin beta 8 |  |  |
|  | 2.3 | microRNA 705 |  |  |
|  | 2.3 | nitric oxide synthase trafficker |  |  |
|  | 2.3 | ectodysplasin A2 receptor |  |  |
|  | 2.3 | G protein-coupled receptor, family C, group 5, member C |  |  |
|  | 2.4 | procollagen-proline, 2-oxoglutarate 4-dioxygenase (proline 4-hydroxylase), alpha II polypeptide |  |  |
|  | 2.4 | FMS-like tyrosine kinase 1 |  |  |
|  | 2.4 | adhesion G protein-coupled receptor E1 |  |  |
|  | 2.4 | mucin 20 |  |  |
|  | 2.4 | LIM domain containing preferred translocation partner in lipoma |  |  |
|  | 2.4 | BAI1-associated protein 2-like 1 |  |  |
|  | 2.4 | multiple coagulation factor deficiency 2 |  |  |
|  | 2.4 | ras homolog family member Q |  |  |
|  | 2.4 | RAB15, member RAS oncogene family |  |  |
|  | 2.4 | ATP binding cassette subfamily G member 1 |  |  |
|  | 2.4 | phospholipase D2 |  |  |
|  | 2.4 | Ras association (RalGDS/AF-6) and pleckstrin homology domains 1 |  |  |
|  | 2.4 | thromboxane A synthase 1, platelet |  |  |
|  | 2.4 | C-type lectin domain family 2, member L |  |  |
|  | 2.4 | thymic stromal lymphopoietin |  |  |
|  | 2.4 | arginine vasopressin-induced 1 |  |  |
|  | 2.4 | purinergic receptor P2X, ligand-gated ion channel 4 |  |  |
|  | 2.4 | enoyl Coenzyme A hydratase domain containing 3 |  |  |
|  | 2.4 | diacylglycerol lipase, alpha |  |  |
|  | 2.4 | predicted gene 6566 |  |  |
|  | 2.4 | apoptosis-inducing factor, mitochondrion-associated 2 |  |  |
|  | 2.4 | family with sequence similarity 113, member B pseudogene |  |  |
|  | 2.4 | tumor necrosis factor receptor superfamily, member 8 |  |  |
|  | 2.4 | testis expressed gene 14 |  |  |
|  | 2.4 | ankyrin repeat and SOCS box-containing 2 |  |  |
|  | 2.4 | ATP-binding cassette, sub-family A (ABC1), member 1 |  |  |
|  | 2.4 | inhibin beta-B |  |  |
|  | 2.4 | solute carrier family 35, member D3 |  |  |
|  | 2.4 | predicted gene 11767 |  |  |
|  | 2.4 | dedicator of cytokinesis 4 |  |  |
|  | 2.4 | tubulin, alpha 8 |  |  |
|  | 2.4 | collagen, type XII, alpha 1 |  |  |
|  | 2.4 | phosphatase and actin regulator 1 |  |  |
|  | 2.4 | forkhead-associated (FHA) phosphopeptide binding domain 1 |  |  |
|  | 2.4 | alpha-N-acetylglucosaminidase (Sanfilippo disease IIIB) |  |  |
|  | 2.5 | threonine aldolase 1 |  |  |
|  | 2.5 | SAM and SH3 domain containing 1 |  |  |
|  | 2.5 | brain-specific angiogenesis inhibitor 1-associated protein 2 |  |  |
|  | 2.5 | alcohol dehydrogenase, iron containing, 1 |  |  |
|  | 2.5 | ring finger protein 19B |  |  |
|  | 2.5 | plexin D1 |  |  |
|  | 2.5 | phosphodiesterase 10A |  |  |
|  | 2.5 | hes family bHLH transcription factor 7 |  |  |
|  | 2.5 | guanylate cyclase activator 1a (retina) |  |  |
|  | 2.5 | prostaglandin D2 synthase (brain) |  |  |
|  | 2.5 | chemokine (C-X-C motif) ligand 5 |  |  |
|  | 2.5 | proviral integration site 3 |  |  |
|  | 2.5 | androglobin |  |  |
|  | 2.5 | F-box and leucine-rich repeat protein 2 |  |  |
|  | 2.5 | PEAK1 related kinase activating pseudokinase 1 |  |  |
|  | 2.5 | RUN and FYVE domain containing 4 |  |  |
|  | 2.5 | cathepsin Z |  |  |
|  | 2.5 | sorting nexin 29 |  |  |
|  | 2.5 | tumor necrosis factor (ligand) superfamily, member 12 |  |  |
|  | 2.5 | predicted gene 4876 |  |  |
|  | 2.5 | platelet-derived growth factor, C polypeptide |  |  |
|  | 2.5 | adaptor protein, phosphotyrosine interaction, PH domain and leucine zipper containing 2 |  |  |
|  | 2.5 | macrophage scavenger receptor 1 |  |  |
|  | 2.5 | WD repeat and FYVE domain containing 2 |  |  |
|  | 2.5 | microRNA 7676-1 |  |  |
|  | 2.5 | mevalonate (diphospho) decarboxylase |  |  |
|  | 2.5 | kynureninase |  |  |
|  | 2.5 | peptidyl-tRNA hydrolase 1 homolog |  |  |
|  | 2.5 | toll-like receptor 4 |  |  |
|  | 2.5 | tubulin, beta 3 class III |  |  |
|  | 2.5 | neuregulin 1 |  |  |
|  | 2.5 | dual specificity phosphatase 4 |  |  |
|  | 2.5 | brain enriched myelin associated protein 1 |  |  |
|  | 2.5 | integrin alpha E, epithelial-associated |  |  |
|  | 2.5 | tumor protein p53 pathway corepressor 1 |  |  |
|  | 2.6 | corticotropin releasing hormone binding protein |  |  |
|  | 2.6 | adenosine A2b receptor |  |  |
|  | 2.6 | PRELI domain containing 2 |  |  |
|  | 2.6 | transmembrane protein 82 |  |  |
|  | 2.6 | cytochrome P450, family 11, subfamily a, polypeptide 1 |  |  |
|  | 2.6 | apelin |  |  |
|  | 2.6 | glyceraldehyde-3-phosphate dehydrogenase pseudogene |  |  |
|  | 2.6 | UDP-Gal:betaGlcNAc beta 1,4-galactosyltransferase, polypeptide 5 |  |  |
|  | 2.6 | lysyl oxidase-like 3 |  |  |
|  | 2.6 | KDEL (Lys-Asp-Glu-Leu) endoplasmic reticulum protein retention receptor 3 |  |  |
|  | 2.6 | pluripotency associated transcript 27 |  |  |
|  | 2.6 | dehydrogenase/reductase (SDR family) member 3 |  |  |
|  | 2.6 | solute carrier family 14 (urea transporter), member 2 |  |  |
|  | 2.6 | solute carrier family 16 (monocarboxylic acid transporters), member 14 |  |  |
|  | 2.6 | protein tyrosine phosphatase, receptor type, f polypeptide (PTPRF), interacting protein (liprin), alpha 3 |  |  |
|  | 2.6 | RIKEN cDNA E230016K23 gene |  |  |
|  | 2.6 | angiogenin, ribonuclease, RNase A family, 5 |  |  |
|  | 2.6 | BCL2-like 14 (apoptosis facilitator) |  |  |
|  | 2.6 | G protein-coupled receptor 176 |  |  |
|  | 2.6 | dipeptidylpeptidase 7 |  |  |
|  | 2.6 | aldehyde dehydrogenase family 1, subfamily A2 |  |  |
|  | 2.6 | SLP adaptor and CSK interacting membrane protein |  |  |
|  | 2.6 | stearoyl-coenzyme A desaturase 4 |  |  |
|  | 2.6 | ADP-ribosylation factor-like 4C |  |  |
|  | 2.6 | glycine C-acetyltransferase (2-amino-3-ketobutyrate-coenzyme A ligase) |  |  |
|  | 2.6 | solute carrier family 41, member 2 |  |  |
|  | 2.6 | cDNA sequence AF067061 |  |  |
|  | 2.6 | predicted gene, 19951 |  |  |
|  | 2.6 | ChaC, cation transport regulator 1 |  |  |
|  | 2.6 | SEC14 and spectrin domains 1 pseudogene |  |  |
|  | 2.6 | lectin, galactose binding, soluble 1 |  |  |
|  | 2.6 | sphingomyelin phosphodiesterase, acid-like 3B |  |  |
|  | 2.6 | caspase 12 |  |  |
|  | 2.6 | asialoglycoprotein receptor 2 |  |  |
|  | 2.6 | phosphatidylinositol-specific phospholipase C, X domain containing 1 |  |  |
|  | 2.6 | succinate receptor 1 |  |  |
|  | 2.6 | nucleolar protein 3 (apoptosis repressor with CARD domain) |  |  |
|  | 2.6 | serine (or cysteine) peptidase inhibitor, clade A, member 3G |  |  |
|  | 2.6 | solute carrier family 7 (cationic amino acid transporter, y+ system), member 11 |  |  |
|  | 2.6 | paternally expressed 10 |  |  |
|  | 2.6 | interferon gamma inducible protein 30 |  |  |
|  | 2.7 | RNA binding protein, fox-1 homolog (C. elegans) 2 |  |  |
|  | 2.7 | prostaglandin F2 receptor negative regulator |  |  |
|  | 2.7 | G protein-coupled receptor 68 |  |  |
|  | 2.7 | serine (or cysteine) peptidase inhibitor, clade B, member 2 |  |  |
|  | 2.7 | cellular repressor of E1A-stimulated genes 2 |  |  |
|  | 2.7 | tumor necrosis factor receptor superfamily, member 23 |  |  |
|  | 2.7 | transmembrane protein 215 |  |  |
|  | 2.7 | transmembrane protein 45a |  |  |
|  | 2.7 | phosphatidylinositol glycan anchor biosynthesis, class Z |  |  |
|  | 2.7 | secretory carrier membrane protein 5 |  |  |
|  | 2.7 | chemokine (C-C motif) ligand 6 |  |  |
|  | 2.7 | adenylate kinase 4 |  |  |
|  | 2.7 | transient receptor potential cation channel, subfamily V, member 4 |  |  |
|  | 2.7 | IQ motif containing G |  |  |
|  | 2.7 | killer cell lectin-like receptor family I member 1 |  |  |
|  | 2.7 | transmembrane protein 273 |  |  |
|  | 2.7 | a disintegrin and metallopeptidase domain 9 (meltrin gamma) |  |  |
|  | 2.7 | tetratricopeptide repeat domain 12 |  |  |
|  | 2.7 | N-acetylneuraminate pyruvate lyase |  |  |
|  | 2.7 | HemK methyltransferase family member 1 |  |  |
|  | 2.7 | hepatocyte growth factor activator |  |  |
|  | 2.7 | RIKEN cDNA A930009A15 gene |  |  |
|  | 2.7 | CD274 antigen |  |  |
|  | 2.7 | predicted gene 14400 |  |  |
|  | 2.7 | Cas scaffolding protein family member 4 |  |  |
|  | 2.7 | melanoregulin |  |  |
|  | 2.7 | tumor necrosis factor receptor superfamily, member 11a, NFKB activator |  |  |
|  | 2.7 | FERM domain containing 4B |  |  |
|  | 2.7 | spindlin family, member 4 |  |  |
|  | 2.7 | solute carrier family 39 (zinc transporter), member 14 |  |  |
|  | 2.7 | mcf.2 transforming sequence-like |  |  |
|  | 2.7 | CNDP dipeptidase 2 (metallopeptidase M20 family) |  |  |
|  | 2.7 | SET binding factor 2 |  |  |
|  | 2.7 | WAP four-disulfide core domain 17 |  |  |
|  | 2.7 | basic helix-loop-helix ARNT like 2 |  |  |
|  | 2.7 | solute carrier family 18 (vesicular monoamine), member 2 |  |  |
|  | 2.7 | polypeptide N-acetylgalactosaminyltransferase 6 |  |  |
|  | 2.7 | solute carrier family 2 (facilitated glucose transporter), member 9 |  |  |
|  | 2.7 | solute carrier family 13 (sodium-dependent dicarboxylate transporter), member 2 |  |  |
|  | 2.7 | growth hormone |  |  |
|  | 2.7 | megakaryocyte-associated tyrosine kinase |  |  |
|  | 2.8 | ST6 (alpha-N-acetyl-neuraminyl-2,3-beta-galactosyl-1,3)-N-acetylgalactosaminide alpha-2,6-sialyltransferase 5 |  |  |
|  | 2.8 | calcium channel, voltage-dependent, beta 2 subunit |  |  |
|  | 2.8 | cyclin J-like |  |  |
|  | 2.8 | collagen, type XVII, alpha 1 |  |  |
|  | 2.8 | aldehyde dehydrogenase 1 family, member L1 |  |  |
|  | 2.8 | plasminogen activator, tissue |  |  |
|  | 2.8 | purinergic receptor P2Y, G-protein coupled 12 |  |  |
|  | 2.8 | U1 small nuclear ribonucleoprotein 1C pseudogene |  |  |
|  | 2.8 | solute carrier family 18 (vesicular monoamine), member 1 |  |  |
|  | 2.8 | indoleamine 2,3-dioxygenase 1 |  |  |
|  | 2.8 | DS cell adhesion molecule like 1 |  |  |
|  | 2.8 | RAB17, member RAS oncogene family |  |  |
|  | 2.8 | mitogen-activated protein kinase kinase kinase 12 |  |  |
|  | 2.8 | enolase 2, gamma neuronal |  |  |
|  | 2.8 | solute carrier family 9 (sodium/hydrogen exchanger), member 2 |  |  |
|  | 2.8 | ankyrin repeat domain 66 |  |  |
|  | 2.8 | MER proto-oncogene tyrosine kinase |  |  |
|  | 2.8 | nuclear protein transcription regulator 1 |  |  |
|  | 2.8 | transforming growth factor, beta 3 |  |  |
|  | 2.8 | cathepsin S |  |  |
|  | 2.8 | calcium channel, voltage-dependent, N type, alpha 1B subunit |  |  |
|  | 2.8 | Mir99a and Mirlet7c-1 host gene (non-protein coding) |  |  |
|  | 2.8 | arrestin domain containing 4 |  |  |
|  | 2.8 | RIKEN cDNA C430002N11 gene |  |  |
|  | 2.8 | vitamin D (1,25-dihydroxyvitamin D3) receptor |  |  |
|  | 2.8 | interferon induced transmembrane protein 1 |  |  |
|  | 2.8 | ankyrin repeat and SOCs box-containing 5 |  |  |
|  | 2.8 | protein tyrosine phosphatase, receptor type, N |  |  |
|  | 2.8 | ankyrin repeat domain 37 |  |  |
|  | 2.8 | RIKEN cDNA 4933412O06 gene |  |  |
|  | 2.8 | Rho GTPase activating protein 28 |  |  |
|  | 2.8 | endothelial PAS domain protein 1 |  |  |
|  | 2.8 | protocadherin 7 |  |  |
|  | 2.8 | predicted gene 12758 |  |  |
|  | 2.8 | melanogenesis associated transcription factor |  |  |
|  | 2.8 | sorting nexin 8 |  |  |
|  | 2.8 | a disintegrin-like and metallopeptidase (reprolysin type) with thrombospondin type 1 motif, 9 |  |  |
|  | 2.8 | interleukin 4 receptor, alpha |  |  |
|  | 2.9 | uridine phosphorylase 2 |  |  |
|  | 2.9 | nectin cell adhesion molecule 2 |  |  |
|  | 2.9 | free fatty acid receptor 4 |  |  |
|  | 2.9 | EP300 interacting inhibitor of differentiation 2 |  |  |
|  | 2.9 | NIMA (never in mitosis gene a)-related expressed kinase 6 |  |  |
|  | 2.9 | insulin-like 6 |  |  |
|  | 2.9 | gametogenetin |  |  |
|  | 2.9 | granzyme B |  |  |
|  | 2.9 | killer cell lectin-like receptor subfamily G, member 2 |  |  |
|  | 2.9 | integrin alpha X |  |  |
|  | 2.9 | lectin, galactose binding, soluble 3 |  |  |
|  | 2.9 | wingless-type MMTV integration site family, member 5A |  |  |
|  | 2.9 | FLYWCH family member 2 |  |  |
|  | 2.9 | Ngfi-A binding protein 2 |  |  |
|  | 2.9 | G protein-coupled receptor 55 |  |  |
|  | 2.9 | interleukin 1 receptor-like 2 |  |  |
|  | 2.9 | guanylate binding protein 11 |  |  |
|  | 2.9 | wingless-type MMTV integration site family, member 9A |  |  |
|  | 2.9 | T cell immunoglobulin and mucin domain containing 2 |  |  |
|  | 2.9 | synaptotagmin VIII |  |  |
|  | 2.9 | receptor-associated protein of the synapse |  |  |
|  | 2.9 | vesicle amine transport 1 |  |  |
|  | 3.0 | CD200 receptor 1 |  |  |
|  | 3.0 | oncoprotein induced transcript 3 |  |  |
|  | 3.0 | nitric oxide synthase 2, inducible |  |  |
|  | 3.0 | angiopoietin-like 2 |  |  |
|  | 3.0 | WW, C2 and coiled-coil domain containing 1 |  |  |
|  | 3.0 | retinoic acid early transcript 1E |  |  |
|  | 3.0 | RAB11 family interacting protein 5 (class I) |  |  |
|  | 3.0 | cytochrome P450, family 4, subfamily f, polypeptide 37 |  |  |
|  | 3.0 | syndecan 4 |  |  |
|  | 3.0 | protein kinase inhibitor beta, cAMP dependent, testis specific |  |  |
|  | 3.0 | cAMP responsive element binding protein 3-like 4 |  |  |
|  | 3.0 | leupaxin |  |  |
|  | 3.0 | intraflagellar transport 43 |  |  |
|  | 3.0 | tetratricopeptide repeat domain 9 |  |  |
|  | 3.0 | histocompatibility 60b |  |  |
|  | 3.0 | ArfGAP with dual PH domains 2 |  |  |
|  | 3.0 | plakophilin 2 |  |  |
|  | 3.0 | colony stimulating factor 2 receptor, beta 2, low-affinity (granulocyte-macrophage) |  |  |
|  | 3.0 | hydroxyacid oxidase 1, liver |  |  |
|  | 3.0 | leucine zipper, putative tumor suppressor 1 |  |  |
|  | 3.0 | meteorin, glial cell differentiation regulator-like |  |  |
|  | 3.0 | t-complex 11 like 1 |  |  |
|  | 3.0 | solute carrier family 39 (zinc transporter), member 4 |  |  |
|  | 3.0 | malic enzyme 1, NADP(+)-dependent, cytosolic pseudogene |  |  |
|  | 3.0 | coiled-coil domain containing 162 |  |  |
|  | 3.0 | solute carrier family 7 (cationic amino acid transporter, y+ system), member 8 |  |  |
|  | 3.0 | BPI fold containing family C |  |  |
|  | 3.0 | SEC14 and spectrin domains 1 |  |  |
|  | 3.0 | RIKEN cDNA 2210408F21 gene |  |  |
|  | 3.0 | family with sequence similarity 170, member B |  |  |
|  | 3.0 | lysyl oxidase-like 4 |  |  |
|  | 3.1 | transmembrane protein 106A |  |  |
|  | 3.1 | glutamine fructose-6-phosphate transaminase 2 |  |  |
|  | 3.1 | CEA cell adhesion molecule 19 |  |  |
|  | 3.1 | ectonucleoside triphosphate diphosphohydrolase 3 |  |  |
|  | 3.1 | cyclin-dependent kinase 20 |  |  |
|  | 3.1 | angiopoietin 2 |  |  |
|  | 3.1 | SH3 and cysteine rich domain 2 |  |  |
|  | 3.1 | retinoic acid early transcript 1, alpha |  |  |
|  | 3.1 | plasminogen activator, urokinase |  |  |
|  | 3.1 | breast cancer anti-estrogen resistance 3 |  |  |
|  | 3.1 | serine (or cysteine) peptidase inhibitor, clade E, member 1 |  |  |
|  | 3.1 | carbohydrate sulfotransferase 14 |  |  |
|  | 3.1 | ephrin A5 |  |  |
|  | 3.1 | protein disulfide isomerase associated 4 |  |  |
|  | 3.1 | CD68 antigen |  |  |
|  | 3.1 | nyctalopin |  |  |
|  | 3.1 | adrenomedullin 2 |  |  |
|  | 3.1 | RIKEN cDNA 6430571L13 gene |  |  |
|  | 3.1 | olfactomedin 1 |  |  |
|  | 3.1 | epidermal growth factor receptor pathway substrate 8 |  |  |
|  | 3.1 | adrenomedullin |  |  |
|  | 3.1 | caspase 6 |  |  |
|  | 3.1 | ets variant 4 |  |  |
|  | 3.1 | piwi-like RNA-mediated gene silencing 2 |  |  |
|  | 3.1 | myocyte enhancer factor 2B |  |  |
|  | 3.2 | six transmembrane epithelial antigen of prostate 2 |  |  |
|  | 3.2 | predicted gene, 46069 |  |  |
|  | 3.2 | Ly6/Plaur domain containing 1 |  |  |
|  | 3.2 | transglutaminase 2, C polypeptide |  |  |
|  | 3.2 | adhesion G protein-coupled receptor G2 |  |  |
|  | 3.2 | cathepsin K |  |  |
|  | 3.2 | cytochrome P450, family 26, subfamily b, polypeptide 1 |  |  |
|  | 3.2 | ring finger protein 180 |  |  |
|  | 3.2 | apolipoprotein L 7d |  |  |
|  | 3.2 | spermatogenesis associated, serine-rich 2 |  |  |
|  | 3.2 | spectrin beta, non-erythrocytic 2 |  |  |
|  | 3.2 | CD200 receptor 4 |  |  |
|  | 3.2 | tubulin, beta 6 class V |  |  |
|  | 3.2 | alpha-kinase 2 |  |  |
|  | 3.2 | actin-related protein 2/3 complex inhibitor |  |  |
|  | 3.2 | myozenin 1 |  |  |
|  | 3.2 | microRNA 704 |  |  |
|  | 3.2 | fidgetin-like 2 |  |  |
|  | 3.2 | cadherin 17 |  |  |
|  | 3.2 | RIKEN cDNA D630039A03 gene |  |  |
|  | 3.2 | FSHD region gene 2 family member 1 |  |  |
|  | 3.3 | insulin receptor-related receptor |  |  |
|  | 3.3 | sodium channel, voltage-gated, type IV, alpha |  |  |
|  | 3.3 | sushi domain containing 4 |  |  |
|  | 3.3 | calpain 13 |  |  |
|  | 3.3 | protein phosphatase 2, regulatory subunit B'', alpha |  |  |
|  | 3.3 | leucine rich repeat containing 66 |  |  |
|  | 3.3 | TBC1 domain family, member 16 |  |  |
|  | 3.3 | lipase, endothelial |  |  |
|  | 3.3 | predicted gene, 17767 |  |  |
|  | 3.3 | glutamine repeat protein 1 |  |  |
|  | 3.3 | solute carrier family 7 (cationic amino acid transporter, y+ system), member 3 |  |  |
|  | 3.3 | solute carrier family 38, member 6 |  |  |
|  | 3.3 | chromodomain helicase DNA binding protein 5 |  |  |
|  | 3.3 | solute carrier family 30 (zinc transporter), member 4 |  |  |
|  | 3.3 | guanine nucleotide binding protein (G protein), gamma 4 |  |  |
|  | 3.3 | expressed sequence C77080 |  |  |
|  | 3.3 | proline rich 15 |  |  |
|  | 3.3 | transient receptor potential cation channel, subfamily M, member 3 |  |  |
|  | 3.3 | cytochrome P450, family 4, subfamily f, polypeptide 16 |  |  |
|  | 3.3 | G protein-coupled receptor 85 |  |  |
|  | 3.3 | cell growth regulator with EF hand domain 1 |  |  |
|  | 3.3 | platelet factor 4 |  |  |
|  | 3.3 | tweety family member 2 |  |  |
|  | 3.3 | basic helix-loop-helix family, member e40 |  |  |
|  | 3.3 | chymase 1, mast cell |  |  |
|  | 3.3 | laccase domain containing 1 |  |  |
|  | 3.4 | homeobox A1 |  |  |
|  | 3.4 | AE binding protein 1 |  |  |
|  | 3.4 | cytochrome P450, family 7, subfamily b, polypeptide 1 |  |  |
|  | 3.4 | regulator of G-protein signaling 11 |  |  |
|  | 3.4 | coagulation factor X |  |  |
|  | 3.4 | dermatan sulfate epimerase |  |  |
|  | 3.4 | chemokine (C-C motif) receptor 5 |  |  |
|  | 3.4 | ets variant gene 5 pseudogene |  |  |
|  | 3.4 | plexin domain containing 2 |  |  |
|  | 3.4 | complement component 1, q subcomponent, alpha polypeptide |  |  |
|  | 3.4 | serine (or cysteine) peptidase inhibitor, clade A, member 3I |  |  |
|  | 3.4 | homeostatic iron regulator |  |  |
|  | 3.4 | chemerin chemokine-like receptor 1 |  |  |
|  | 3.4 | matrix metallopeptidase 14 (membrane-inserted) |  |  |
|  | 3.4 | PTK6 protein tyrosine kinase 6 |  |  |
|  | 3.4 | delta like non-canonical Notch ligand 1 |  |  |
|  | 3.4 | interleukin 1 alpha |  |  |
|  | 3.4 | ring finger protein 128 |  |  |
|  | 3.4 | C-type lectin domain family 7, member a |  |  |
|  | 3.4 | predicted gene, 31223 |  |  |
|  | 3.4 | dedicator of cyto-kinesis 3 |  |  |
|  | 3.4 | cystatin B |  |  |
|  | 3.4 | myoferlin |  |  |
|  | 3.5 | renin binding protein |  |  |
|  | 3.5 | predicted gene 16201 |  |  |
|  | 3.5 | protein kinase, cAMP dependent regulatory, type I beta |  |  |
|  | 3.5 | unc-13 homolog A |  |  |
|  | 3.5 | matrix metallopeptidase 27 |  |  |
|  | 3.5 | insulin-like growth factor 1 |  |  |
|  | 3.5 | interleukin 13 |  |  |
|  | 3.5 | mastermind-like domain containing 1 |  |  |
|  | 3.5 | solute carrier family 2 (facilitated glucose transporter), member 13 |  |  |
|  | 3.5 | very low density lipoprotein receptor |  |  |
|  | 3.5 | transcription factor EC |  |  |
|  | 3.5 | colony stimulating factor 1 (macrophage) |  |  |
|  | 3.5 | RIKEN cDNA D930048N14 gene |  |  |
|  | 3.5 | Fc receptor, IgG, low affinity IIb |  |  |
|  | 3.5 | predicted gene, 20767 |  |  |
|  | 3.5 | Ndufa4, mitochondrial complex associated like 2 |  |  |
|  | 3.5 | uridine phosphorylase 1 |  |  |
|  | 3.5 | MAF bZIP transcription factor B |  |  |
|  | 3.5 | multiple EGF-like-domains 11 |  |  |
|  | 3.5 | T cell-interacting, activating receptor on myeloid cells 1 |  |  |
|  | 3.5 | RIKEN cDNA 4930579C12 gene |  |  |
|  | 3.5 | hedgehog acyltransferase |  |  |
|  | 3.5 | chemokine (C-X-C motif) receptor 1 |  |  |
|  | 3.5 | prostate transmembrane protein, androgen induced 1 |  |  |
|  | 3.6 | cyclin-dependent kinase 18 |  |  |
|  | 3.6 | pleckstrin homology domain containing, family F (with FYVE domain) member 1 |  |  |
|  | 3.6 | atonal bHLH transcription factor 8 |  |  |
|  | 3.6 | Cd200 receptor 2 |  |  |
|  | 3.6 | egl-9 family hypoxia-inducible factor 3 |  |  |
|  | 3.6 | docking protein 6 |  |  |
|  | 3.6 | BCL2/adenovirus E1B interacting protein 3 |  |  |
|  | 3.6 | leukotriene C4 synthase |  |  |
|  | 3.6 | ral guanine nucleotide dissociation stimulator,-like 1 |  |  |
|  | 3.6 | zinc finger, MYND-type containing 15 |  |  |
|  | 3.6 | tumor necrosis factor receptor superfamily, member 9 |  |  |
|  | 3.6 | ets variant 5 |  |  |
|  | 3.6 | stearoyl-Coenzyme A desaturase 2 |  |  |
|  | 3.7 | formin 1 |  |  |
|  | 3.7 | trans-golgi network vesicle protein 23A |  |  |
|  | 3.7 | GRB2 associated regulator of MAPK1 subtype 2 |  |  |
|  | 3.7 | 5'-nucleotidase, cytosolic IA |  |  |
|  | 3.7 | murinoglobulin 1 |  |  |
|  | 3.7 | noggin |  |  |
|  | 3.7 | niban apoptosis regulator 2 |  |  |
|  | 3.7 | retinoic acid induced 14 |  |  |
|  | 3.7 | ribonuclease, RNase A family 4 |  |  |
|  | 3.7 | A kinase (PRKA) anchor protein 6 |  |  |
|  | 3.7 | predicted gene 13546 |  |  |
|  | 3.7 | chemokine (C-C motif) ligand 9 |  |  |
|  | 3.7 | cysteinyl leukotriene receptor 1 |  |  |
|  | 3.7 | predicted gene 13470 |  |  |
|  | 3.7 | predicted gene 5122 |  |  |
|  | 3.7 | tropomodulin 2 |  |  |
|  | 3.7 | synapsin III |  |  |
|  | 3.7 | heme binding protein 2 |  |  |
|  | 3.7 | a disintegrin-like and metallopeptidase (reprolysin type) with thrombospondin type 1 motif, 4 |  |  |
|  | 3.7 | erythrocyte membrane protein band 4.1 like 1 |  |  |
|  | 3.7 | complement component 1, q subcomponent, C chain |  |  |
|  | 3.7 | dynamin 1 |  |  |
|  | 3.7 | N-deacetylase/N-sulfotransferase (heparan glucosaminyl) 3 |  |  |
|  | 3.8 | mucolipin 2 |  |  |
|  | 3.8 | ubiquitin specific peptidase 43 |  |  |
|  | 3.8 | transmembrane protein 144 |  |  |
|  | 3.8 | early growth response 2 |  |  |
|  | 3.8 | extracellular matrix protein 1 |  |  |
|  | 3.8 | calcium channel, voltage-dependent, gamma subunit 8 |  |  |
|  | 3.8 | potassium voltage-gated channel, subfamily H (eag-related), member 3 |  |  |
|  | 3.8 | leucine rich repeat containing 2 |  |  |
|  | 3.8 | C-type lectin domain family 10, member A |  |  |
|  | 3.8 | activating transcription factor 5 |  |  |
|  | 3.8 | ankyrin repeat and SOCS box-containing 4 |  |  |
|  | 3.8 | DNA-damage-inducible transcript 4-like |  |  |
|  | 3.8 | cytokine inducible SH2-containing protein |  |  |
|  | 3.8 | carbonic anhydrase 5b, mitochondrial |  |  |
|  | 3.9 | GATA binding protein 6 |  |  |
|  | 3.9 | RIKEN cDNA 2610300M13 gene |  |  |
|  | 3.9 | zinc finger protein 366 |  |  |
|  | 3.9 | uronyl-2-sulfotransferase |  |  |
|  | 3.9 | solute carrier family 6 (neurotransmitter transporter, L-proline), member 7 |  |  |
|  | 3.9 | PDZ and LIM domain 4 |  |  |
|  | 3.9 | solute carrier family 1 (glutamate/neutral amino acid transporter), member 4 |  |  |
|  | 3.9 | UDP-GlcNAc:betaGal beta-1,3-N-acetylglucosaminyltransferase 7 |  |  |
|  | 3.9 | dihydropyrimidinase-like 5 |  |  |
|  | 3.9 | lysyl oxidase |  |  |
|  | 3.9 | microRNA 5114 |  |  |
|  | 3.9 | sine oculis-related homeobox 4 |  |  |
|  | 3.9 | RIKEN cDNA 9130019P16 gene |  |  |
|  | 3.9 | RUN and SH3 domain containing 2 |  |  |
|  | 3.9 | neuronal PAS domain protein 2 |  |  |
|  | 3.9 | chemokine (C-X-C motif) ligand 16 |  |  |
|  | 3.9 | cadherin-related family member 1 |  |  |
|  | 3.9 | RIKEN cDNA G530011O06 gene |  |  |
|  | 3.9 | collagen, type XVIII, alpha 1 |  |  |
|  | 3.9 | CTTNBP2 N-terminal like |  |  |
|  | 4.0 | sel-1 suppressor of lin-12-like 3 (C. elegans) |  |  |
|  | 4.0 | transmembrane and immunoglobulin domain containing 1 |  |  |
|  | 4.0 | MAF bZIP transcription factor |  |  |
|  | 4.0 | aldolase C, fructose-bisphosphate |  |  |
|  | 4.0 | cannabinoid receptor interacting protein 1 |  |  |
|  | 4.0 | plexin A1 |  |  |
|  | 4.0 | carbonyl reductase 3 |  |  |
|  | 4.0 | RIKEN cDNA 0610040F04 gene |  |  |
|  | 4.0 | mab-21-like 3 |  |  |
|  | 4.0 | predicted gene 12589 |  |  |
|  | 4.0 | aldo-keto reductase family 1, member C18 |  |  |
|  | 4.0 | gap junction protein, alpha 1 |  |  |
|  | 4.0 | Hedgehog-interacting protein |  |  |
|  | 4.1 | dynein, axonemal, heavy chain 2 |  |  |
|  | 4.1 | ral guanine nucleotide dissociation stimulator |  |  |
|  | 4.1 | sorting nexing 24 |  |  |
|  | 4.1 | glutathione S-transferase, mu 2 |  |  |
|  | 4.1 | p21 (RAC1) activated kinase 3 |  |  |
|  | 4.1 | RIKEN cDNA F830045P16 gene |  |  |
|  | 4.1 | RIKEN cDNA A930007I19 gene |  |  |
|  | 4.1 | pyrimidinergic receptor P2Y, G-protein coupled, 6 |  |  |
|  | 4.1 | neuropilin 2 |  |  |
|  | 4.1 | cadherin 1 |  |  |
|  | 4.1 | transmembrane 4 superfamily member 5 |  |  |
|  | 4.1 | predicted gene 5127 |  |  |
|  | 4.1 | tetratricopeptide repeat, ankyrin repeat and coiled-coil containing 2 |  |  |
|  | 4.1 | splA/ryanodine receptor domain and SOCS box containing 1 |  |  |
|  | 4.2 | six transmembrane epithelial antigen of the prostate 1 |  |  |
|  | 4.2 | STRA6-like |  |  |
|  | 4.2 | CSA-conditional, T cell activation-dependent protein |  |  |
|  | 4.2 | predicted gene 8773 |  |  |
|  | 4.2 | potassium channel, subfamily K, member 13 |  |  |
|  | 4.2 | kinesin family member 1A |  |  |
|  | 4.2 | annexin A4 |  |  |
|  | 4.2 | hepatitis A virus cellular receptor 2 |  |  |
|  | 4.2 | B cell leukemia/lymphoma 2 related protein A1d |  |  |
|  | 4.2 | taxilin beta |  |  |
|  | 4.2 | filamin binding LIM protein 1 |  |  |
|  | 4.2 | matrix metallopeptidase 19 |  |  |
|  | 4.2 | ST8 alpha-N-acetyl-neuraminide alpha-2,8-sialyltransferase 1 |  |  |
|  | 4.2 | 4-hydroxy-2-oxoglutarate aldolase 1 |  |  |
|  | 4.3 | complement component 1, q subcomponent, beta polypeptide |  |  |
|  | 4.3 | predicted gene, 30794 |  |  |
|  | 4.3 | recoverin |  |  |
|  | 4.3 | taste receptor, type 2, member 143 |  |  |
|  | 4.3 | solute carrier family 36 (proton/amino acid symporter), member 2 |  |  |
|  | 4.3 | pyroglutamyl-peptidase I-like |  |  |
|  | 4.3 | tribbles pseudokinase 3 |  |  |
|  | 4.3 | RIKEN cDNA 4933416M06 gene |  |  |
|  | 4.3 | delta like non-canonical Notch ligand 2 |  |  |
|  | 4.3 | matrilin 3 |  |  |
|  | 4.3 | basic leucine zipper transcription factor, ATF-like 3 |  |  |
|  | 4.3 | RIKEN cDNA D330050G23 gene |  |  |
|  | 4.3 | serine (or cysteine) peptidase inhibitor, clade B, member 8 |  |  |
|  | 4.3 | predicted gene, 22935 |  |  |
|  | 4.3 | potassium intermediate/small conductance calcium-activated channel, subfamily N, member 3 |  |  |
|  | 4.3 | osteoclast stimulatory transmembrane protein |  |  |
|  | 4.3 | CD163 antigen |  |  |
|  | 4.3 | low density lipoprotein-related protein 12 |  |  |
|  | 4.4 | predicted gene 4610 |  |  |
|  | 4.4 | serine (or cysteine) peptidase inhibitor, clade A, member 3H |  |  |
|  | 4.4 | kallikrein 1-related peptidase b9 |  |  |
|  | 4.4 | triggering receptor expressed on myeloid cells 2 |  |  |
|  | 4.4 | lysosomal-associated membrane protein family, member 5 |  |  |
|  | 4.4 | predicted gene, 34643 |  |  |
|  | 4.4 | male germ cell-associated kinase |  |  |
|  | 4.4 | secreted phosphoprotein 1 |  |  |
|  | 4.4 | solute carrier family 4 (anion exchanger), member 4 |  |  |
|  | 4.4 | EGF-like repeats and discoidin I-like domains 3 |  |  |
|  | 4.4 | retinoic acid receptor responder (tazarotene induced) 1 |  |  |
|  | 4.4 | tumor protein D52-like 1 |  |  |
|  | 4.4 | RIKEN cDNA A430108G06 gene |  |  |
|  | 4.4 | predicted gene 2396 |  |  |
|  | 4.4 | B cell leukemia/lymphoma 2 related protein A1b |  |  |
|  | 4.4 | glutamic pyruvate transaminase (alanine aminotransferase) 2 |  |  |
|  | 4.4 | chloride channel, voltage-sensitive 5 |  |  |
|  | 4.4 | cysteine-rich secretory protein 1 |  |  |
|  | 4.4 | guanylate cyclase 2g |  |  |
|  | 4.4 | RIKEN cDNA 4932438H23 gene |  |  |
|  | 4.4 | serine (or cysteine) peptidase inhibitor, clade A, member 3B |  |  |
|  | 4.4 | tumor necrosis factor, alpha-induced protein 8-like 3 |  |  |
|  | 4.4 | copine VIII |  |  |
|  | 4.5 | suppressor of cytokine signaling 2 |  |  |
|  | 4.5 | suppressor of cytokine signaling 1 |  |  |
|  | 4.5 | neural cell adhesion molecule 2 |  |  |
|  | 4.5 | glyceraldehyde-3-phosphate dehydrogenase pseudogene |  |  |
|  | 4.5 | gastric inhibitory polypeptide receptor |  |  |
|  | 4.5 | CEA cell adhesion molecule 15 |  |  |
|  | 4.5 | X-ray radiation resistance associated 1 |  |  |
|  | 4.5 | G protein subunit alpha transducin 3 |  |  |
|  | 4.5 | cadherin 22 |  |  |
|  | 4.5 | phosphatase domain containing, paladin 1 |  |  |
|  | 4.6 | FAM20C, golgi associated secretory pathway kinase |  |  |
|  | 4.6 | fibroblast growth factor 23 |  |  |
|  | 4.6 | interleukin 13 receptor, alpha 2 |  |  |
|  | 4.6 | ATP-binding cassette, sub-family A (ABC1), member 4 |  |  |
|  | 4.7 | fascin actin-bundling protein 1 |  |  |
|  | 4.7 | chemokine (C-C motif) ligand 2 |  |  |
|  | 4.7 | G protein-coupled receptor 82 |  |  |
|  | 4.7 | serine (or cysteine) peptidase inhibitor, clade B (ovalbumin), member 12 |  |  |
|  | 4.7 | glutamic pyruvic transaminase, soluble pseudogene |  |  |
|  | 4.7 | fibroblast growth factor 2 |  |  |
|  | 4.7 | neuropeptide Y |  |  |
|  | 4.7 | BTB and CNC homology 2, opposite strand |  |  |
|  | 4.7 | C-type lectin domain family 4, member n |  |  |
|  | 4.7 | ERBB receptor feedback inhibitor 1 |  |  |
|  | 4.7 | ribosomal protein S2 pseudogene |  |  |
|  | 4.7 | dendrocyte expressed seven transmembrane protein |  |  |
|  | 4.7 | legumain |  |  |
|  | 4.7 | predicted gene, 34833 |  |  |
|  | 4.8 | TOG array regulator of axonemal microtubules 2 |  |  |
|  | 4.8 | B cell leukemia/lymphoma 2 related protein A1a |  |  |
|  | 4.8 | carbohydrate sulfotransferase 8 |  |  |
|  | 4.8 | eosinophil-associated, ribonuclease A family, pseudogene 10 |  |  |
|  | 4.8 | guanine nucleotide binding protein (G protein), beta 3 |  |  |
|  | 4.8 | placental growth factor |  |  |
|  | 4.8 | protein phosphatase 1, regulatory subunit 3G |  |  |
|  | 4.8 | transmembrane protein 150C |  |  |
|  | 4.8 | Na+/K+ transporting ATPase interacting 1 |  |  |
|  | 4.8 | membrane-spanning 4-domains, subfamily A, member 14 |  |  |
|  | 4.8 | RIKEN cDNA A230028O05 gene |  |  |
|  | 4.9 | carbonic anhydrase 6 |  |  |
|  | 4.9 | aquaporin 3 |  |  |
|  | 4.9 | SH3 and PX domains 2B |  |  |
|  | 4.9 | interleukin 23 receptor |  |  |
|  | 4.9 | hyaluronan and proteoglycan link protein 1 |  |  |
|  | 4.9 | antizyme inhibitor 2 |  |  |
|  | 4.9 | RIKEN cDNA 5730435O14 gene |  |  |
|  | 4.9 | stanniocalcin 2 |  |  |
|  | 4.9 | transmembrane 4 L six family member 19 |  |  |
|  | 4.9 | N-myc downstream regulated gene 4 |  |  |
|  | 5.0 | predicted gene 15413 |  |  |
|  | 5.0 | patatin-like phospholipase domain containing 3 |  |  |
|  | 5.0 | solute carrier family 39 (zinc transporter), member 12 |  |  |
|  | 5.0 | transmembrane protein 92 |  |  |
|  | 5.0 | aldo-keto reductase family 1, member B8 |  |  |
|  | 5.0 | solute carrier family 6 (neurotransmitter transporter, creatine), member 8 |  |  |
|  | 5.0 | membrane-spanning 4-domains, subfamily A, member 7 |  |  |
|  | 5.0 | butyrophilin-like 1 |  |  |
|  | 5.0 | predicted gene 5861 |  |  |
|  | 5.0 | predicted gene 12022 |  |  |
|  | 5.0 | septin 3 |  |  |
|  | 5.1 | predicted gene, 33100 |  |  |
|  | 5.1 | olfactory receptor family 2 subfamily AG member 15 |  |  |
|  | 5.1 | stabilin 1 |  |  |
|  | 5.1 | golgi associated kinase 1B |  |  |
|  | 5.1 | THO complex 4 pseudogene |  |  |
|  | 5.1 | small proline-rich protein 2A3 |  |  |
|  | 5.1 | monooxygenase, DBH-like 1 |  |  |
|  | 5.1 | apolipoprotein L 7a |  |  |
|  | 5.1 | myosin, light chain 10, regulatory |  |  |
|  | 5.1 | chemokine (C-C motif) ligand 22 |  |  |
|  | 5.1 | indoleamine 2,3-dioxygenase 2 |  |  |
|  | 5.1 | phospholipase A2, group V |  |  |
|  | 5.1 | ets variant 1 |  |  |
|  | 5.2 | family with sequence similarity 240 member A |  |  |
|  | 5.2 | epithelial membrane protein 1 |  |  |
|  | 5.2 | membrane-spanning 4-domains, subfamily A, member 6D |  |  |
|  | 5.2 | eukaryotic translation elongation factor 1 alpha 2 |  |  |
|  | 5.2 | potassium inwardly-rectifying channel, subfamily J, member 4 |  |  |
|  | 5.2 | G protein regulated inducer of neurite outgrowth 2 |  |  |
|  | 5.2 | membrane-spanning 4-domains, subfamily A, member 4A |  |  |
|  | 5.2 | aryl-hydrocarbon receptor repressor |  |  |
|  | 5.3 | histocompatibility 2, M region locus 5 |  |  |
|  | 5.3 | olfactory receptor family 2 subfamily AG member 18 |  |  |
|  | 5.3 | solute carrier family 1 (glial high affinity glutamate transporter), member 2 |  |  |
|  | 5.3 | cell migration inducing protein, hyaluronan binding |  |  |
|  | 5.3 | progesterone receptor |  |  |
|  | 5.3 | cathepsin L |  |  |
|  | 5.3 | H1.8 linker histone |  |  |
|  | 5.3 | zinc finger protein 469 |  |  |
|  | 5.3 | RIKEN cDNA F630040K05 gene |  |  |
|  | 5.3 | grainyhead like transcription factor 3 |  |  |
|  | 5.3 | sema domain, immunoglobulin domain (Ig), short basic domain, secreted, (semaphorin) 3C |  |  |
|  | 5.3 | apoptosis-inducing factor, mitochondrion-associated 3 |  |  |
|  | 5.3 | matrix metallopeptidase 10 |  |  |
|  | 5.4 | suppression of tumorigenicity 18 |  |  |
|  | 5.4 | sine oculis-related homeobox 1 |  |  |
|  | 5.4 | regulatory associated protein of MTOR, complex 1, opposite strand |  |  |
|  | 5.4 | endothelin 1 |  |  |
|  | 5.4 | matrix metallopeptidase 13 |  |  |
|  | 5.4 | prune homolog 2 |  |  |
|  | 5.4 | germ cell associated 1 |  |  |
|  | 5.4 | V-set and immunoglobulin domain containing 8 |  |  |
|  | 5.4 | complement component 3a receptor 1 |  |  |
|  | 5.4 | oxoglutarate dehydrogenase-like |  |  |
|  | 5.4 | RIKEN cDNA 4933417E11 gene |  |  |
|  | 5.5 | interleukin 4 induced 1 |  |  |
|  | 5.5 | superoxide dismutase 3, extracellular |  |  |
|  | 5.5 | ovo like zinc finger 2 |  |  |
|  | 5.5 | myotubularin related protein 7 |  |  |
|  | 5.5 | glutathione S-transferase, alpha 2 (Yc2) |  |  |
|  | 5.5 | transmembrane protein 37 |  |  |
|  | 5.5 | dimethylarginine dimethylaminohydrolase 1 |  |  |
|  | 5.5 | fatty acid binding protein 5, epidermal |  |  |
|  | 5.5 | transmembrane protein 59-like |  |  |
|  | 5.5 | receptor (calcitonin) activity modifying protein 3 |  |  |
|  | 5.5 | proprotein convertase subtilisin/kexin type 9 |  |  |
|  | 5.5 | stimulated by retinoic acid gene 6 |  |  |
|  | 5.5 | solute carrier family 9 (sodium/hydrogen exchanger), member 4 |  |  |
|  | 5.5 | phospholipase A2 inhibitor and LY6/PLAUR domain containing |  |  |
|  | 5.6 | mitochondria localized glutamic acid rich protein |  |  |
|  | 5.6 | macrophage galactose N-acetyl-galactosamine specific lectin 2 |  |  |
|  | 5.6 | synaptotagmin XIII |  |  |
|  | 5.6 | 5-hydroxytryptamine (serotonin) receptor 7 |  |  |
|  | 5.7 | small proline-rich protein 2A1 |  |  |
|  | 5.7 | TAFA chemokine like family member 3 |  |  |
|  | 5.7 | tumor necrosis factor receptor superfamily, member 11b (osteoprotegerin) |  |  |
|  | 5.7 | NADPH-dependent carbonyl reductase pseudogene |  |  |
|  | 5.7 | folate receptor 2 (fetal) |  |  |
|  | 5.7 | sodium channel, voltage-gated, type II, alpha |  |  |
|  | 5.8 | ARFGEF family member 3 |  |  |
|  | 5.8 | sodium channel, voltage-gated, type I, alpha |  |  |
|  | 5.8 | aldehyde dehydrogenase family 1, subfamily A3 |  |  |
|  | 5.8 | placenta expressed transcript 1 |  |  |
|  | 5.8 | ENTH domain containing 1 |  |  |
|  | 5.8 | chemokine (C-C motif) ligand 17 |  |  |
|  | 5.9 | transition protein 2 |  |  |
|  | 6.0 | lysozyme-like 4 |  |  |
|  | 6.0 | neurexophilin and PC-esterase domain family, member 5 |  |  |
|  | 6.0 | carbonyl reductase 2 |  |  |
|  | 6.0 | myelin regulatory factor |  |  |
|  | 6.0 | predicted gene 5833 |  |  |
|  | 6.0 | transmembrane protein 26 |  |  |
|  | 6.1 | death domain containing 1 |  |  |
|  | 6.1 | podoplanin |  |  |
|  | 6.1 | TNFAIP3 interacting protein 3 |  |  |
|  | 6.1 | ladinin |  |  |
|  | 6.2 | kelch-like 33 |  |  |
|  | 6.2 | platelet derived growth factor, alpha |  |  |
|  | 6.2 | calcium channel, voltage-dependent, beta 3 subunit |  |  |
|  | 6.2 | sterile alpha motif domain containing 5 |  |  |
|  | 6.2 | carbonic anhydrase 8 |  |  |
|  | 6.2 | BTB (POZ) domain containing 17 |  |  |
|  | 6.2 | spermatogenesis associated 18 |  |  |
|  | 6.3 | purinergic receptor P2X, ligand-gated ion channel, 5 |  |  |
|  | 6.3 | ribosomal modification protein rimK-like family member A |  |  |
|  | 6.3 | kallikrein 1-related peptidase b11 |  |  |
|  | 6.3 | kinase suppressor of ras 2 |  |  |
|  | 6.4 | apolipoprotein L 7c |  |  |
|  | 6.4 | aristaless related homeobox |  |  |
|  | 6.5 | CD209e antigen |  |  |
|  | 6.5 | a disintegrin and metallopeptidase domain 23 |  |  |
|  | 6.5 | AHNAK nucleoprotein 2 |  |  |
|  | 6.5 | TRPM8 channel-associated factor 2 |  |  |
|  | 6.7 | predicted gene 15056 |  |  |
|  | 6.7 | mannose receptor, C type 1 |  |  |
|  | 6.7 | programmed cell death 1 ligand 2 |  |  |
|  | 6.7 | phospholipase A2, group IIE |  |  |
|  | 6.8 | B cell leukemia/lymphoma 2 related protein A1c |  |  |
|  | 6.8 | DNA methyltransferase 3A, opposite strand |  |  |
|  | 6.8 | cAMP responsive element binding protein 5 |  |  |
|  | 6.8 | ATPase, H+ transporting, lysosomal V0 subunit D2 |  |  |
|  | 6.8 | heparin-binding EGF-like growth factor |  |  |
|  | 6.8 | small proline-rich protein 2A2 |  |  |
|  | 6.8 | endothelin receptor type B |  |  |
|  | 6.9 | RIKEN cDNA D830013O20 gene |  |  |
|  | 7.0 | alanyl (membrane) aminopeptidase |  |  |
|  | 7.0 | Rho GTPase activating protein 8 |  |  |
|  | 7.0 | glycoprotein (transmembrane) nmb |  |  |
|  | 7.0 | pre T cell antigen receptor alpha |  |  |
|  | 7.0 | predicted gene 6093 |  |  |
|  | 7.0 | aldehyde dehydrogenase 1 family, member L2 |  |  |
|  | 7.1 | tissue inhibitor of metalloproteinase 1 |  |  |
|  | 7.1 | solute carrier family 28 (sodium-coupled nucleoside transporter), member 3 |  |  |
|  | 7.1 | hippocalcin-like 4 |  |  |
|  | 7.2 | predicted gene, 19434 |  |  |
|  | 7.2 | Fc receptor-like B |  |  |
|  | 7.3 | sodium channel, voltage-gated, type IX, alpha |  |  |
|  | 7.4 | Fc receptor like 2 |  |  |
|  | 7.4 | fibronectin leucine rich transmembrane protein 3 |  |  |
|  | 7.4 | disabled 2, mitogen-responsive phosphoprotein |  |  |
|  | 7.5 | proline-rich transmembrane protein 4 |  |  |
|  | 7.6 | membrane associated ring-CH-type finger 10 |  |  |
|  | 7.6 | claudin 11 |  |  |
|  | 7.7 | ankyrin repeat domain 55 |  |  |
|  | 7.8 | RIKEN cDNA 4930512J16 gene |  |  |
|  | 7.8 | serum amyloid A 3 |  |  |
|  | 8.0 | fibronectin leucine rich transmembrane protein 2 |  |  |
|  | 8.1 | coagulation factor VII |  |  |
|  | 8.2 | predicted gene 6116 |  |  |
|  | 8.6 | solute carrier family 7 (cationic amino acid transporter, y+ system), member 2 |  |  |
|  | 8.8 | 5-hydroxytryptamine (serotonin) receptor 2B |  |  |
|  | 8.9 | ribonuclease, RNase A family, 2A (liver, eosinophil-derived neurotoxin) |  |  |
|  | 8.9 | transmembrane protein 171 |  |  |
|  | 9.2 | sodium channel, voltage-gated, type III, alpha |  |  |
|  | 9.5 | lipase, family member N |  |  |
|  | 9.5 | serine protease inhibitor, Kunitz type 1 |  |  |
|  | 9.7 | chemokine (C-C motif) ligand 7 |  |  |
|  | 9.9 | chemokine (C-C motif) ligand 12 |  |  |
|  | 10.0 | retinol binding protein 4, plasma |  |  |
|  | 10.2 | cellular communication network factor 3 |  |  |
|  | 10.5 | carbonic anhydrase 4 |  |  |
|  | 10.7 | potassium voltage-gated channel, Shal-related family, member 3 |  |  |
|  | 10.8 | matrix metallopeptidase 12 |  |  |
|  | 11.6 | chemokine (C-C motif) ligand 8 |  |  |
|  | 13.3 | msh homeobox 3 |  |  |
|  | 13.6 | cholesterol 25-hydroxylase |  |  |
|  | 14.2 | chemokine (C-C motif) ligand 24 |  |  |
|  | 14.2 | resistin like alpha |  |  |
|  | 14.6 | arginase, liver |  |  |
| Total 1863 |  |  |  |  |
| **D. Comparison of mRNA expression in stimulated and unstimulated BMCs from OCT3^−/−^-mice** | | |  |  |
|  | -10.2 | solute carrier family 30, member 10 |  |  |
|  | -10.1 | tachykinin 2 |  |  |
|  | -9.7 | polycystic kidney and hepatic disease 1-like 1 |  |  |
|  | -9.7 | protein disulfide isomerase associated 2 |  |  |
|  | -9.3 | ATP binding cassette subfamily G member 4 |  |  |
|  | -9.3 | RIKEN cDNA 9830132P13 gene |  |  |
|  | -9.0 | fructosamine 3 kinase |  |  |
|  | -8.9 | SH3 domain and tetratricopeptide repeats 2 |  |  |
|  | -8.9 | TLC domain containing 4 |  |  |
|  | -8.8 | angiotensin II receptor, type 1a |  |  |
|  | -8.7 | phytanoyl-CoA hydroxylase interacting protein |  |  |
|  | -8.6 | Rh blood group, D antigen |  |  |
|  | -8.5 | Rhesus blood group-associated A glycoprotein |  |  |
|  | -8.5 | ring finger protein 212 |  |  |
|  | -8.5 | calcium channel, voltage-dependent, T type, alpha 1G subunit |  |  |
|  | -8.4 | butyrophilin-like 10 |  |  |
|  | -8.2 | Redrum, erythroid developmental long intergenic non-protein coding transcript |  |  |
|  | -8.0 | pyruvate kinase liver and red blood cell |  |  |
|  | -8.0 | glial fibrillary acidic protein |  |  |
|  | -8.0 | butyrophilin, subfamily 1, member A1 |  |  |
|  | -7.9 | microRNA 144 |  |  |
|  | -7.7 | solute carrier family 38, member 5 |  |  |
|  | -7.6 | microRNA 451a |  |  |
|  | -7.6 | AKNA domain containing 1 |  |  |
|  | -7.6 | hemoglobin X, alpha-like embryonic chain in Hba complex |  |  |
|  | -7.6 | erythroblast membrane-associated protein |  |  |
|  | -7.5 | ankyrin repeat and SOCS box-containing 17, opposite strand |  |  |
|  | -7.5 | predicted gene, 37915 |  |  |
|  | -7.5 | solute carrier family 2 (facilitated glucose transporter), member 4 |  |  |
|  | -7.4 | collagen, type XIV, alpha 1 |  |  |
|  | -7.4 | adducin 2 (beta) |  |  |
|  | -7.3 | predicted gene, 40372 |  |  |
|  | -7.3 | apolipoprotein L 8 |  |  |
|  | -7.3 | ankyrin 1, erythroid |  |  |
|  | -7.2 | calcineurin-like EF hand protein 2 |  |  |
|  | -7.2 | phospholamban |  |  |
|  | -7.2 | predicted gene 11837 |  |  |
|  | -7.1 | desmocollin 2 |  |  |
|  | -6.9 | sosondowah ankyrin repeat domain family member A |  |  |
|  | -6.9 | inhibitor of carbonic anhydrase |  |  |
|  | -6.8 | membrane metallo endopeptidase |  |  |
|  | -6.8 | SRY (sex determining region Y)-box 6 |  |  |
|  | -6.8 | ankyrin repeat and SOCS box-containing 17 |  |  |
|  | -6.7 | solute carrier family 25 (mitochondrial oxodicarboxylate carrier), member 21 |  |  |
|  | -6.7 | predicted gene 867 |  |  |
|  | -6.6 | protease, serine 50 |  |  |
|  | -6.6 | SH2 domain containing 4A |  |  |
|  | -6.6 | dematin actin binding protein |  |  |
|  | -6.6 | spectrin alpha, erythrocytic 1 |  |  |
|  | -6.6 | aquaporin 1 |  |  |
|  | -6.6 | LON peptidase N-terminal domain and ring finger 2 |  |  |
|  | -6.5 | carbonic anhydrase 1 |  |  |
|  | -6.5 | golgi associated RAB2 interactor family member 4 |  |  |
|  | -6.5 | Wnt inhibitory factor 1 |  |  |
|  | -6.5 | apolipoprotein L 11a |  |  |
|  | -6.5 | polycystic kidney disease 1 like 1 |  |  |
|  | -6.5 | acyl-CoA synthetase long-chain family member 6 |  |  |
|  | -6.5 | amino carboxymuconate semialdehyde decarboxylase |  |  |
|  | -6.5 | hemogen |  |  |
|  | -6.4 | angiotensin I converting enzyme (peptidyl-dipeptidase A) 1 |  |  |
|  | -6.3 | fer-1-like 6 (C. elegans) |  |  |
|  | -6.3 | glycerophosphodiester phosphodiesterase domain containing 2 |  |  |
|  | -6.3 | Kruppel-like factor 1 (erythroid) |  |  |
|  | -6.2 | transient receptor potential cation channel, subfamily V, member 5 |  |  |
|  | -6.2 | solute carrier family 6 (neurotransmitter transporter), member 20A |  |  |
|  | -6.2 | RIKEN cDNA A730036I17 gene |  |  |
|  | -6.2 | apolipoprotein L 10C, pseudogene |  |  |
|  | -6.1 | leucine rich repeat containing 39 |  |  |
|  | -6.1 | coiled-coil domain containing 74A |  |  |
|  | -6.1 | predicted gene, 33934 |  |  |
|  | -6.1 | predicted gene 13010 |  |  |
|  | -6.0 | neurexophilin and PC-esterase domain family, member 2 |  |  |
|  | -6.0 | carbonic anhydrase 1 pseudogene |  |  |
|  | -6.0 | predicted gene 2061 |  |  |
|  | -5.9 | angiopoietin-like 1 |  |  |
|  | -5.9 | erythrocyte membrane protein band 4.2 |  |  |
|  | -5.9 | RIKEN cDNA 5430431A17 gene |  |  |
|  | -5.9 | WAP four-disulfide core domain 3 |  |  |
|  | -5.8 | RIKEN cDNA 1810053B23 gene |  |  |
|  | -5.8 | bone gamma-carboxyglutamate protein 2 |  |  |
|  | -5.8 | thrombospondin, type I, domain containing 7B |  |  |
|  | -5.8 | tripartite motif-containing 10 |  |  |
|  | -5.8 | RIKEN cDNA 9530026F06 gene |  |  |
|  | -5.8 | leukocyte immunoglobulin-like receptor, subfamily A (with TM domain), member 5 |  |  |
|  | -5.7 | SRY (sex determining region Y)-box 6, opposite strand |  |  |
|  | -5.7 | atypical chemokine receptor 4 |  |  |
|  | -5.7 | spectrin beta, erythrocytic |  |  |
|  | -5.7 | G-protein-coupled receptor 50 |  |  |
|  | -5.7 | G protein-coupled receptor 182 |  |  |
|  | -5.7 | Kell blood group |  |  |
|  | -5.6 | carboxylesterase 2G |  |  |
|  | -5.6 | predicted gene 15290 |  |  |
|  | -5.6 | solute carrier family 4 (anion exchanger), member 1 |  |  |
|  | -5.6 | predicted gene 14862 |  |  |
|  | -5.6 | apolipoprotein L 10A |  |  |
|  | -5.6 | synaptotagmin XIV |  |  |
|  | -5.6 | sorting nexin 22 |  |  |
|  | -5.6 | aldolase B, fructose-bisphosphate |  |  |
|  | -5.6 | claudin 13 |  |  |
|  | -5.6 | sterile alpha motif domain containing 11 |  |  |
|  | -5.6 | RIKEN cDNA gene D630044L22 gene |  |  |
|  | -5.5 | agmatine ureohydrolase (agmatinase) |  |  |
|  | -5.5 | RIKEN cDNA 5430401H09 gene |  |  |
|  | -5.5 | ATPase, Cu++ transporting, beta polypeptide |  |  |
|  | -5.5 | apolipoprotein D |  |  |
|  | -5.5 | apolipoprotein L 9b |  |  |
|  | -5.5 | predicted gene 27179 |  |  |
|  | -5.5 | serine rich and transmembrane domain containing 2 |  |  |
|  | -5.5 | membrane metallo-endopeptidase-like 1 |  |  |
|  | -5.5 | immunoglobulin kappa chain variable 7-33 |  |  |
|  | -5.4 | artemin |  |  |
|  | -5.4 | a disintegrin and metallopeptidase domain 33 |  |  |
|  | -5.4 | tetraspanin 33 |  |  |
|  | -5.4 | 3-oxoacid CoA transferase 2B |  |  |
|  | -5.4 | aldehyde dehydrogenase family 1, subfamily A7 |  |  |
|  | -5.4 | PDZ domain containing 1 |  |  |
|  | -5.3 | odd-skipped related 2 |  |  |
|  | -5.3 | ectodysplasin-A receptor |  |  |
|  | -5.3 | RIKEN cDNA 2810459M11 gene |  |  |
|  | -5.3 | predicted gene, 16793 |  |  |
|  | -5.3 | RasGEF domain family, member 1C |  |  |
|  | -5.3 | pleckstrin 2 |  |  |
|  | -5.2 | synaptotagmin II |  |  |
|  | -5.2 | protease, serine 2 |  |  |
|  | -5.2 | chondromodulin |  |  |
|  | -5.2 | RIKEN cDNA 2610027K06 gene |  |  |
|  | -5.1 | popeye domain containing 2 |  |  |
|  | -5.1 | major intrinsic protein of lens fiber |  |  |
|  | -5.1 | patched 2 |  |  |
|  | -5.1 | predicted gene, 20161 |  |  |
|  | -5.1 | T cell receptor delta variable 2-2 |  |  |
|  | -5.1 | RUNX1 translocation partner 1 |  |  |
|  | -5.1 | progestin and adipoQ receptor family member IX |  |  |
|  | -5.1 | solute carrier family 26 (sulfate transporter), member 1 |  |  |
|  | -5.1 | myosin XVA |  |  |
|  | -5.1 | microRNA 486 |  |  |
|  | -5.1 | zinc finger protein 385C |  |  |
|  | -5.1 | phospholipase A2, group IID |  |  |
|  | -5.0 | testis expressed gene 21 |  |  |
|  | -5.0 | theg spermatid protein like |  |  |
|  | -5.0 | elastin |  |  |
|  | -5.0 | ribonuclease, RNase A family, 2B (liver, eosinophil-derived neurotoxin) |  |  |
|  | -5.0 | calsequestrin 2 |  |  |
|  | -5.0 | predicted gene, 34567 |  |  |
|  | -5.0 | glycophorin A |  |  |
|  | -5.0 | calmegin |  |  |
|  | -5.0 | integrin binding sialoprotein |  |  |
|  | -5.0 | myopalladin |  |  |
|  | -5.0 | POU domain, class 3, transcription factor 1 |  |  |
|  | -4.9 | divergent protein kinase domain 1C |  |  |
|  | -4.9 | CD209f antigen |  |  |
|  | -4.9 | testis-specific protein, Y-encoded-like 5 |  |  |
|  | -4.9 | predicted gene, 24265 |  |  |
|  | -4.9 | ATPase, Na+/K+ transporting, beta 2 polypeptide |  |  |
|  | -4.9 | complement component 6 |  |  |
|  | -4.9 | adiponectin, C1Q and collagen domain containing |  |  |
|  | -4.9 | carbonic anhydrase 2 |  |  |
|  | -4.9 | kelch repeat and BTB (POZ) domain containing 12 |  |  |
|  | -4.8 | angiotensinogen (serpin peptidase inhibitor, clade A, member 8) |  |  |
|  | -4.8 | coiled-coil domain containing 92B |  |  |
|  | -4.8 | selectin, endothelial cell |  |  |
|  | -4.8 | predicted gene 6249 |  |  |
|  | -4.8 | RIKEN cDNA 1700063H04 gene |  |  |
|  | -4.8 | immunoglobulin kappa variable 1-108 |  |  |
|  | -4.8 | glycosylphosphatidylinositol anchored molecule like 2 |  |  |
|  | -4.8 | carboxylesterase 2F |  |  |
|  | -4.8 | tetraspanin 8 |  |  |
|  | -4.8 | apolipoprotein L 11b |  |  |
|  | -4.8 | PPARGC1 and ESRR induced regulator, muscle 1 |  |  |
|  | -4.7 | zinc finger, C2HC-type containing 1C |  |  |
|  | -4.7 | ATP-binding cassette, sub-family B (MDR/TAP), member 4 |  |  |
|  | -4.7 | syncytin b |  |  |
|  | -4.7 | RIKEN cDNA F930017D23 gene |  |  |
|  | -4.7 | tumor necrosis factor receptor superfamily, member 17 |  |  |
|  | -4.7 | yippee like 4 |  |  |
|  | -4.7 | cysteine rich protein 2 |  |  |
|  | -4.7 | dentin matrix protein 1 |  |  |
|  | -4.7 | microsomal glutathione S-transferase 3 |  |  |
|  | -4.7 | RIKEN cDNA 4933440M02 gene |  |  |
|  | -4.7 | dynein axonemal assembly factor 11 |  |  |
|  | -4.6 | solute carrier family 1 (glial high affinity glutamate transporter), member 3 |  |  |
|  | -4.6 | interleukin 22 receptor, alpha 1 |  |  |
|  | -4.6 | cytochrome P450, family 4, subfamily b, polypeptide 1 |  |  |
|  | -4.6 | folate receptor 1 (adult) |  |  |
|  | -4.6 | zinc finger protein 853 |  |  |
|  | -4.6 | somatostatin receptor 2 |  |  |
|  | -4.6 | predicted gene, 41043 |  |  |
|  | -4.6 | DENN domain containing 2B |  |  |
|  | -4.5 | R-spondin 2 |  |  |
|  | -4.5 | coiled-coil domain containing 68 |  |  |
|  | -4.5 | intercellular adhesion molecule 4, Landsteiner-Wiener blood group |  |  |
|  | -4.5 | solute carrier family 28 (sodium-coupled nucleoside transporter), member 1 |  |  |
|  | -4.5 | hairy/enhancer-of-split related with YRPW motif-like |  |  |
|  | -4.5 | ankyrin repeat domain 65 |  |  |
|  | -4.5 | aminolevulinate, delta-, dehydratase |  |  |
|  | -4.5 | sphingosine-1-phosphate receptor 5 |  |  |
|  | -4.5 | cache domain containing 1 |  |  |
|  | -4.5 | predicted gene 15816 |  |  |
|  | -4.5 | polypeptide N-acetylgalactosaminyltransferase 15 |  |  |
|  | -4.4 | leucine rich repeat containing 23 |  |  |
|  | -4.4 | C-type lectin domain family 14, member a |  |  |
|  | -4.4 | sorting nexin 31 |  |  |
|  | -4.4 | cyclin-dependent kinase-like 1 (CDC2-related kinase) |  |  |
|  | -4.4 | serine (or cysteine) peptidase inhibitor, clade A, member 3C |  |  |
|  | -4.4 | DnaJ heat shock protein family (Hsp40) member B3 |  |  |
|  | -4.4 | transient receptor potential cation channel, subfamily A, member 1 |  |  |
|  | -4.4 | solute carrier organic anion transporter family, member 1a5 |  |  |
|  | -4.4 | T cell receptor beta, variable 13-1 |  |  |
|  | -4.4 | transmembrane channel-like gene family 5 |  |  |
|  | -4.4 | CD248 antigen, endosialin |  |  |
|  | -4.4 | potassium inwardly-rectifying channel, subfamily J, member 10 |  |  |
|  | -4.4 | predicted gene, 26748 |  |  |
|  | -4.4 | killer cell lectin-like receptor, subfamily A, member 4 |  |  |
|  | -4.4 | collagen, type XXII, alpha 1 |  |  |
|  | -4.4 | a disintegrin and metallopeptidase domain 2 |  |  |
|  | -4.4 | snail family zinc finger 2 |  |  |
|  | -4.3 | myosin, heavy polypeptide 10, non-muscle |  |  |
|  | -4.3 | coiled-coil domain containing 103 |  |  |
|  | -4.3 | contactin 3 |  |  |
|  | -4.3 | solute carrier family 43, member 1 |  |  |
|  | -4.3 | Ttc39a opposite strand RNA 1 |  |  |
|  | -4.3 | dual specificity phosphatase 8 |  |  |
|  | -4.3 | phospholipid phosphatase 1 |  |  |
|  | -4.3 | carboxyesterase 2B |  |  |
|  | -4.3 | H2A histone family member L1K |  |  |
|  | -4.3 | tetraspanin 15 |  |  |
|  | -4.2 | predicted gene, 47996 |  |  |
|  | -4.2 | hemoglobin, theta 1B |  |  |
|  | -4.2 | solute carrier family 6 (neurotransmitter transporter), member 20B |  |  |
|  | -4.2 | RIKEN cDNA 0610043K17 gene |  |  |
|  | -4.2 | microRNA 7032 |  |  |
|  | -4.2 | tripartite motif-containing 15 |  |  |
|  | -4.2 | matrix metallopeptidase 21 |  |  |
|  | -4.2 | atypical chemokine receptor 1 (Duffy blood group) |  |  |
|  | -4.2 | killer cell lectin-like receptor subfamily A, member 14, pseudogene |  |  |
|  | -4.2 | DnaJ heat shock protein family (Hsp40) member A4 |  |  |
|  | -4.2 | transmembrane protein 178 |  |  |
|  | -4.2 | leptin receptor |  |  |
|  | -4.2 | predicted gene, 23971 |  |  |
|  | -4.2 | VANGL planar cell polarity 1 |  |  |
|  | -4.2 | FH2 domain containing 1 |  |  |
|  | -4.2 | translocator protein 2 |  |  |
|  | -4.2 | microRNA 3061 |  |  |
|  | -4.2 | butyrophilin-like 6 |  |  |
|  | -4.2 | solute carrier family 25, member 37 |  |  |
|  | -4.1 | schlafen 14 |  |  |
|  | -4.1 | RIKEN cDNA E330032C10 gene |  |  |
|  | -4.1 | membrane protein, palmitoylated 2 (MAGUK p55 subfamily member 2) |  |  |
|  | -4.1 | beta-1,4-N-acetyl-galactosaminyl transferase 3 |  |  |
|  | -4.1 | NHL repeat containing 4 |  |  |
|  | -4.1 | immunoglobulin-like domain containing receptor 2 |  |  |
|  | -4.1 | microRNA 877 |  |  |
|  | -4.1 | olfactory receptor family 2 subfamily W member 3 |  |  |
|  | -4.1 | predicted gene, 39213 |  |  |
|  | -4.1 | MAGE family member B16 |  |  |
|  | -4.1 | regulator of G-protein signaling 6 |  |  |
|  | -4.1 | ADAM-like, decysin 1 |  |  |
|  | -4.1 | predicted gene, 17344 |  |  |
|  | -4.1 | sterile alpha motif domain containing 4 |  |  |
|  | -4.1 | cyclin M1 |  |  |
|  | -4.1 | coiled-coil domain containing 141 |  |  |
|  | -4.0 | tropomodulin 1 |  |  |
|  | -4.0 | small nucleolar RNA SNORD50 |  |  |
|  | -4.0 | ectonucleotide pyrophosphatase/phosphodiesterase 3 |  |  |
|  | -4.0 | killer cell lectin-like receptor, subfamily A, member 8 |  |  |
|  | -4.0 | RIKEN cDNA 2700054A10 gene |  |  |
|  | -4.0 | inhibitor of DNA binding 4 |  |  |
|  | -4.0 | IQ motif containing D |  |  |
|  | -4.0 | spondin 2, extracellular matrix protein |  |  |
|  | -4.0 | predicted gene 8947 |  |  |
|  | -4.0 | predicted gene, 26316 |  |  |
|  | -3.9 | predicted gene, 16867 |  |  |
|  | -3.9 | C1q and tumor necrosis factor related protein 4 |  |  |
|  | -3.9 | ferrochelatase |  |  |
|  | -3.9 | predicted gene, 47995 |  |  |
|  | -3.9 | serine/threonine/tyrosine kinase 1 |  |  |
|  | -3.9 | SEC14-like lipid binding 2 |  |  |
|  | -3.9 | bone morphogenetic protein 6 |  |  |
|  | -3.9 | double PHD fingers 3 |  |  |
|  | -3.9 | leucine-rich repeats and immunoglobulin-like domains 1 |  |  |
|  | -3.9 | H2B clustered histone 27 |  |  |
|  | -3.9 | erythroferrone |  |  |
|  | -3.9 | killer cell lectin-like receptor subfamily B member 1 |  |  |
|  | -3.9 | predicted gene, 30292 |  |  |
|  | -3.9 | neuronal growth regulator 1 |  |  |
|  | -3.9 | CD40 ligand |  |  |
|  | -3.9 | cysteine-rich secretory protein 2 |  |  |
|  | -3.9 | ribosomal protein S3 pseudogene |  |  |
|  | -3.8 | DEPP1 autophagy regulator |  |  |
|  | -3.8 | paraneoplastic antigen MA1 |  |  |
|  | -3.8 | regulatory factor X, 2 (influences HLA class II expression) |  |  |
|  | -3.8 | erythropoietin receptor |  |  |
|  | -3.8 | Fas apoptotic inhibitory molecule 2 |  |  |
|  | -3.8 | prostaglandin D receptor |  |  |
|  | -3.8 | FBJ osteosarcoma oncogene B |  |  |
|  | -3.8 | ficolin B |  |  |
|  | -3.8 | hydroxymethylbilane synthase |  |  |
|  | -3.8 | collagen, type XI, alpha 1 |  |  |
|  | -3.8 | solute carrier family 22 (organic cation transporter), member 4 pseudogene |  |  |
|  | -3.8 | microRNA 3058 |  |  |
|  | -3.8 | ureidopropionase, beta |  |  |
|  | -3.7 | solute carrier family 5 (sodium/glucose cotransporter), member 2 |  |  |
|  | -3.7 | uroporphyrinogen III synthase |  |  |
|  | -3.7 | cytochrome P450, family 4, subfamily f, polypeptide 39 |  |  |
|  | -3.7 | ADP-ribosyltransferase 4 |  |  |
|  | -3.7 | fructose bisphosphatase 1 |  |  |
|  | -3.7 | transcription elongation factor A (SII)-like 3 |  |  |
|  | -3.7 | transmembrane channel-like gene family 3 |  |  |
|  | -3.7 | ATPase, Ca++ transporting, plasma membrane 4 |  |  |
|  | -3.7 | otoferlin |  |  |
|  | -3.7 | UDP galactosyltransferase 8A |  |  |
|  | -3.7 | kininogen 1 |  |  |
|  | -3.7 | osteoglycin |  |  |
|  | -3.7 | ubiquitin associated domain containing 1 |  |  |
|  | -3.7 | predicted gene, 23690 |  |  |
|  | -3.7 | poly(A) binding protein, cytoplasmic 2 pseudogene |  |  |
|  | -3.7 | family with sequence similarity 205, member A1 |  |  |
|  | -3.7 | chemokine (C motif) ligand 1 |  |  |
|  | -3.7 | nuclear factor of kappa light polypeptide gene enhancer in B cells inhibitor, zeta |  |  |
|  | -3.7 | Kruppel-like factor 11 |  |  |
|  | -3.7 | transglutaminase 3, E polypeptide |  |  |
|  | -3.7 | adrenergic receptor, beta 1 |  |  |
|  | -3.6 | tripartite motif-containing 58 |  |  |
|  | -3.6 | ubiquitin associated domain containing 1 pseudogene |  |  |
|  | -3.6 | ninein-like |  |  |
|  | -3.6 | neural retina leucine zipper gene |  |  |
|  | -3.6 | H2A histone family member L1M |  |  |
|  | -3.6 | transcription factor Dp 2 |  |  |
|  | -3.6 | potassium channel, subfamily K, member 2 |  |  |
|  | -3.6 | RIKEN cDNA 3110040M04 gene |  |  |
|  | -3.6 | phospholipase A2, group IVC (cytosolic, calcium-independent) |  |  |
|  | -3.6 | nuclear receptor subfamily 4, group A, member 1 |  |  |
|  | -3.6 | family with sequence similarity 210, member B |  |  |
|  | -3.6 | G-protein coupled receptor 88 |  |  |
|  | -3.6 | bone morphogenetic protein 8b |  |  |
|  | -3.6 | membrane associated ring-CH-type finger 3 |  |  |
|  | -3.6 | REC114 meiotic recombination protein |  |  |
|  | -3.6 | predicted gene, 47985 |  |  |
|  | -3.5 | aldehyde dehydrogenase family 1, subfamily A1 |  |  |
|  | -3.5 | immunoglobulin superfamily containing leucine-rich repeat |  |  |
|  | -3.5 | pyruvate carboxylase |  |  |
|  | -3.5 | solute carrier family 22, member 23 |  |  |
|  | -3.5 | ADP-ribosylation factor-like 4A |  |  |
|  | -3.5 | dual-specificity tyrosine-(Y)-phosphorylation regulated kinase 3 |  |  |
|  | -3.5 | membrane bound O-acyltransferase domain containing 2 |  |  |
|  | -3.5 | H2B clustered histone 18 |  |  |
|  | -3.5 | family with sequence similarity 229, member A |  |  |
|  | -3.5 | small integral membrane protein 1 |  |  |
|  | -3.5 | GRB2 associated regulator of MAPK1 subtype 1 |  |  |
|  | -3.5 | polycystic kidney disease 2-like 2 |  |  |
|  | -3.5 | chloride channel, voltage-sensitive 2 |  |  |
|  | -3.5 | orosomucoid 2 |  |  |
|  | -3.5 | H1.5 linker histone, cluster member |  |  |
|  | -3.5 | glycoprotein m6a |  |  |
|  | -3.5 | microRNA 7048 |  |  |
|  | -3.5 | coproporphyrinogen oxidase |  |  |
|  | -3.5 | bone gamma carboxyglutamate protein |  |  |
|  | -3.4 | glutaminase 2 (liver, mitochondrial) |  |  |
|  | -3.4 | TLC domain containing 5 |  |  |
|  | -3.4 | regulator of G protein signaling 7 |  |  |
|  | -3.4 | R-spondin 1 |  |  |
|  | -3.4 | IQ motif containing N |  |  |
|  | -3.4 | solute carrier family 26, member 7 |  |  |
|  | -3.4 | FBJ osteosarcoma oncogene |  |  |
|  | -3.4 | immunoglobulin heavy constant alpha |  |  |
|  | -3.4 | ST3 beta-galactoside alpha-2,3-sialyltransferase 5 |  |  |
|  | -3.4 | dehydrogenase/reductase (SDR family) member 11 |  |  |
|  | -3.4 | glutathione S-transferase, alpha 4 |  |  |
|  | -3.4 | uroporphyrinogen decarboxylase |  |  |
|  | -3.4 | chemokine (C-X3-C motif) receptor 1 |  |  |
|  | -3.4 | solute carrier family 16 (monocarboxylic acid transporters), member 10 |  |  |
|  | -3.4 | fibroblast growth factor receptor-like 1 |  |  |
|  | -3.3 | frizzled class receptor 9 |  |  |
|  | -3.3 | origin recognition complex, subunit 1 |  |  |
|  | -3.3 | RIKEN cDNA 4930507D05 gene |  |  |
|  | -3.3 | C1q and tumor necrosis factor related 12 |  |  |
|  | -3.3 | cingulin |  |  |
|  | -3.3 | chordin-like 1 |  |  |
|  | -3.3 | neurexophilin and PC-esterase domain family, member 4 |  |  |
|  | -3.3 | ATP-binding cassette, sub-family B (MDR/TAP), member 10 |  |  |
|  | -3.3 | H1.3 linker histone, cluster member |  |  |
|  | -3.3 | microRNA 7653 |  |  |
|  | -3.3 | Kruppel-like factor 2 (lung) |  |  |
|  | -3.3 | RIKEN cDNA 6030468B19 gene |  |  |
|  | -3.3 | exportin 7 |  |  |
|  | -3.3 | cathepsin F |  |  |
|  | -3.2 | myosin, light polypeptide 9, regulatory |  |  |
|  | -3.2 | peroxiredoxin 2 |  |  |
|  | -3.2 | solute carrier family 51, alpha subunit |  |  |
|  | -3.2 | potassium voltage-gated channel, subfamily G, member 1 |  |  |
|  | -3.2 | ATP binding cassette subfamily G member 2 (Junior blood group) |  |  |
|  | -3.2 | NK2 homeobox 3 |  |  |
|  | -3.2 | tyrosine kinase, non-receptor, 1 |  |  |
|  | -3.2 | fructosamine 3 kinase related protein |  |  |
|  | -3.2 | kininogen 2 |  |  |
|  | -3.2 | solute carrier family 6 (neurotransmitter transporter, glycine), member 9 |  |  |
|  | -3.2 | tyrosine kinase, non-receptor 2, opposite strand |  |  |
|  | -3.2 | predicted gene 6729 |  |  |
|  | -3.2 | glutathione S-transferase, mu 5 |  |  |
|  | -3.2 | START domain containing 10 |  |  |
|  | -3.2 | cerebellin 1 precursor protein |  |  |
|  | -3.2 | bone morphogenic protein/retinoic acid inducible neural-specific 2 |  |  |
|  | -3.2 | 2'-5' oligoadenylate synthetase 1E |  |  |
|  | -3.2 | H2B clustered histone 8 |  |  |
|  | -3.2 | glutathione S-transferase pi 3 |  |  |
|  | -3.1 | hemoglobin beta, pseudogene bh3 |  |  |
|  | -3.1 | multiple EGF-like-domains 6 |  |  |
|  | -3.1 | CD59a antigen |  |  |
|  | -3.1 | pirin |  |  |
|  | -3.1 | thrombospondin 4 |  |  |
|  | -3.1 | transmembrane and coiled-coil domains 2 |  |  |
|  | -3.1 | olfactory receptor family 52 subfamily Z member 1 |  |  |
|  | -3.1 | lipase, member H |  |  |
|  | -3.1 | histidine ammonia lyase |  |  |
|  | -3.1 | cerebellar degeneration-related 2 |  |  |
|  | -3.1 | radical S-adenosyl methionine domain containing 2 |  |  |
|  | -3.1 | chemokine (C-X-C motif) ligand 9 |  |  |
|  | -3.1 | predicted gene 10371 |  |  |
|  | -3.1 | TLC domain containing 1 |  |  |
|  | -3.1 | adhesion G protein-coupled receptor L3 |  |  |
|  | -3.1 | H2B.U histone 1, pseudogene |  |  |
|  | -3.1 | glutamate-ammonia ligase (glutamine synthetase) pseudogene |  |  |
|  | -3.1 | killer cell lectin-like receptor subfamily A, member 13, pseudogene |  |  |
|  | -3.1 | phosphatidylserine synthase 2 |  |  |
|  | -3.1 | enkurin, TRPC channel interacting protein |  |  |
|  | -3.1 | fibulin 5 |  |  |
|  | -3.1 | solute carrier family 22 (organic cation transporter), member 4 |  |  |
|  | -3.1 | myosin light chain, phosphorylatable, fast skeletal muscle |  |  |
|  | -3.1 | myosin light chain kinase 3 |  |  |
|  | -3.1 | spire type actin nucleation factor 1 |  |  |
|  | -3.0 | transmembrane and tetratricopeptide repeat containing 1 |  |  |
|  | -3.0 | predicted gene, 22154 |  |  |
|  | -3.0 | H2B clustered histone 13 |  |  |
|  | -3.0 | immunoglobulin kappa chain variable 12-38 |  |  |
|  | -3.0 | orosomucoid 1 |  |  |
|  | -3.0 | doublesex and mab-3 related transcription factor 3 |  |  |
|  | -3.0 | trafficking protein, kinesin binding 2 |  |  |
|  | -3.0 | ficolin A |  |  |
|  | -3.0 | Rho guanine nucleotide exchange factor (GEF) 37 |  |  |
|  | -3.0 | predicted gene 14137 |  |  |
|  | -3.0 | glutamate rich 5 |  |  |
|  | -3.0 | small integral membrane protein 6 |  |  |
|  | -3.0 | Kruppel-like factor 4 (gut) |  |  |
|  | -3.0 | NAC alpha domain containing |  |  |
|  | -3.0 | DENN domain containing 2C |  |  |
|  | -3.0 | protein phosphatase 1 (formerly 2C)-like |  |  |
|  | -3.0 | tripartite motif-containing 2 |  |  |
|  | -3.0 | olfactory marker protein |  |  |
|  | -3.0 | killer cell lectin-like receptor subfamily B member 1F |  |  |
|  | -3.0 | thymocyte selection-associated high mobility group box |  |  |
|  | -3.0 | H1.2 linker histone, cluster member |  |  |
|  | -3.0 | ATP-binding cassette, sub-family B (MDR/TAP), member 6 |  |  |
|  | -2.9 | heparan sulfate 6-O-sulfotransferase 1 |  |  |
|  | -2.9 | 4-aminobutyrate aminotransferase |  |  |
|  | -2.9 | terminal nucleotidyltransferase 5C |  |  |
|  | -2.9 | H2B clustered histone 7 |  |  |
|  | -2.9 | intercellular adhesion molecule 5, telencephalin |  |  |
|  | -2.9 | olfactory receptor family 52 subfamily A member 5 |  |  |
|  | -2.9 | DnaJ heat shock protein family (Hsp40) member B2 |  |  |
|  | -2.9 | CD59b antigen |  |  |
|  | -2.9 | cytidine monophosphate (UMP-CMP) kinase 2, mitochondrial |  |  |
|  | -2.9 | meiosis 1 associated protein |  |  |
|  | -2.9 | complement receptor 2 |  |  |
|  | -2.9 | transmembrane epididymal family member 2 |  |  |
|  | -2.9 | killer cell lectin-like receptor family I member 2 |  |  |
|  | -2.9 | H4 histone 16 |  |  |
|  | -2.9 | Eph receptor B1 |  |  |
|  | -2.9 | T cell acute lymphocytic leukemia 1 |  |  |
|  | -2.9 | H1.1 linker histone, cluster member |  |  |
|  | -2.9 | EGF-like-domain, multiple 6 |  |  |
|  | -2.9 | predicted gene, 17315 |  |  |
|  | -2.9 | dual specificity phosphatase 1 |  |  |
|  | -2.9 | brain expressed X-linked 4 |  |  |
|  | -2.9 | immunoglobulin joining chain |  |  |
|  | -2.9 | CD300E molecule |  |  |
|  | -2.9 | S100 protein, beta polypeptide, neural |  |  |
|  | -2.9 | X-linked Kx blood group |  |  |
|  | -2.9 | glutaredoxin 5 |  |  |
|  | -2.9 | C-type lectin domain family 4, member g |  |  |
|  | -2.9 | killer cell lectin-like receptor subfamily A, member 9 |  |  |
|  | -2.8 | phosphatidylinositol glycan anchor biosynthesis, class Q |  |  |
|  | -2.8 | phospholipase A and acyltransferase 3 |  |  |
|  | -2.8 | ependymin related protein 1 (zebrafish) |  |  |
|  | -2.8 | SPARC related modular calcium binding 2 |  |  |
|  | -2.8 | predicted gene, 22748 |  |  |
|  | -2.8 | natural cytotoxicity triggering receptor 1 |  |  |
|  | -2.8 | ubiquitin-conjugating enzyme E2O |  |  |
|  | -2.8 | G-protein signalling modulator 2 (AGS3-like, C. elegans) |  |  |
|  | -2.8 | transferrin receptor 2 |  |  |
|  | -2.8 | synuclein, alpha |  |  |
|  | -2.8 | RAN binding protein 10 |  |  |
|  | -2.8 | von Willebrand factor C and EGF domains |  |  |
|  | -2.8 | copine family member IX |  |  |
|  | -2.8 | Sh3 domain YSC-like 1 |  |  |
|  | -2.8 | transmembrane serine protease 6 |  |  |
|  | -2.8 | E2F transcription factor 2 |  |  |
|  | -2.8 | pregnancy-associated plasma protein A |  |  |
|  | -2.8 | macrophage stimulating 1 (hepatocyte growth factor-like) |  |  |
|  | -2.8 | potassium channel tetramerisation domain containing 14 |  |  |
|  | -2.8 | H2B clustered histone 3 |  |  |
|  | -2.8 | tetratricopeptide repeat domain 39A |  |  |
|  | -2.7 | dishevelled associated activator of morphogenesis 2 |  |  |
|  | -2.7 | cytotoxic T-lymphocyte-associated protein 4 |  |  |
|  | -2.7 | SH2 domain containing 1A |  |  |
|  | -2.7 | transmembrane protein 235 |  |  |
|  | -2.7 | pyridoxine 5'-phosphate oxidase |  |  |
|  | -2.7 | endothelial cell-specific molecule 1 |  |  |
|  | -2.7 | amine oxidase, copper containing 3 |  |  |
|  | -2.7 | family with sequence similarity 117, member A |  |  |
|  | -2.7 | OTU domain, ubiquitin aldehyde binding 2 |  |  |
|  | -2.7 | carboxypeptidase E |  |  |
|  | -2.7 | predicted gene, 33619 |  |  |
|  | -2.7 | growth factor receptor bound protein 14 |  |  |
|  | -2.7 | apelin receptor |  |  |
|  | -2.7 | rad and gem related GTP binding protein 1 |  |  |
|  | -2.7 | death-associated protein kinase 2 |  |  |
|  | -2.7 | ring finger protein 123 |  |  |
|  | -2.7 | small nucleolar RNA, C/D box 57 |  |  |
|  | -2.7 | prokineticin receptor 1 |  |  |
|  | -2.7 | sorbin and SH3 domain containing 1 |  |  |
|  | -2.7 | cell division cycle 6 |  |  |
|  | -2.7 | solute carrier family 16 (monocarboxylic acid transporters), member 1 |  |  |
|  | -2.7 | sperm antigen with calponin homology and coiled-coil domains 1 |  |  |
|  | -2.7 | non imprinted in Prader-Willi/Angelman syndrome 1 homolog (human) |  |  |
|  | -2.7 | aminolevulinic acid synthase 2, erythroid |  |  |
|  | -2.7 | dynein, axonemal, heavy chain 6 |  |  |
|  | -2.7 | 2,3-bisphosphoglycerate mutase |  |  |
|  | -2.7 | hydroxyprostaglandin dehydrogenase 15 (NAD) |  |  |
|  | -2.7 | predicted gene, 33111 |  |  |
|  | -2.7 | glycophorin C |  |  |
|  | -2.7 | prokineticin 2 |  |  |
|  | -2.7 | predicted gene 12579 |  |  |
|  | -2.7 | calcium channel, voltage-dependent, T type, alpha 1H subunit |  |  |
|  | -2.7 | cytochrome c oxidase subunit 6B2 |  |  |
|  | -2.7 | H2A clustered histone 25 |  |  |
|  | -2.7 | platelet-derived growth factor, D polypeptide |  |  |
|  | -2.7 | chemokine (C-C motif) ligand 5 |  |  |
|  | -2.7 | dipeptidase 1 |  |  |
|  | -2.7 | ankyrin repeat and SOCS box-containing 1 |  |  |
|  | -2.6 | golgi associated RAB2 interactor 3 |  |  |
|  | -2.6 | cathelicidin antimicrobial peptide |  |  |
|  | -2.6 | collagen, type I, alpha 1 |  |  |
|  | -2.6 | H2B clustered histone 9 |  |  |
|  | -2.6 | heat shock protein 1A |  |  |
|  | -2.6 | BCLl2-like 15 |  |  |
|  | -2.6 | interferon-related developmental regulator 2 |  |  |
|  | -2.6 | GTP cyclohydrolase 1 |  |  |
|  | -2.6 | flavin containing monooxygenase 2 |  |  |
|  | -2.6 | TSC22 domain family, member 3 |  |  |
|  | -2.6 | microRNA 6903 |  |  |
|  | -2.6 | nuclear receptor interacting protein 3 |  |  |
|  | -2.6 | H2B clustered histone 4 |  |  |
|  | -2.6 | tumor necrosis factor (ligand) superfamily, member 13b |  |  |
|  | -2.6 | interferon gamma |  |  |
|  | -2.6 | Rho guanine nucleotide exchange factor (GEF) 12 |  |  |
|  | -2.6 | V-set and transmembrane domain containing 4 |  |  |
|  | -2.6 | H3 clustered histone 7 |  |  |
|  | -2.6 | thyroid stimulating hormone receptor |  |  |
|  | -2.6 | bromodomain and PHD finger containing, 3 |  |  |
|  | -2.6 | phosphate regulating endopeptidase homolog, X-linked |  |  |
|  | -2.6 | small nucleolar RNA, C/D box 53 |  |  |
|  | -2.6 | zinc finger, FYVE domain containing 28 |  |  |
|  | -2.6 | RIKEN cDNA 2810029C07 gene |  |  |
|  | -2.6 | vesicular, overexpressed in cancer, prosurvival protein 1 |  |  |
|  | -2.6 | LIM and calponin homology domains 1 |  |  |
|  | -2.6 | meiosis-specific nuclear structural protein 1 |  |  |
|  | -2.6 | sphingosine kinase 1 |  |  |
|  | -2.6 | eosinophil-associated, ribonuclease A family, pseudogene 12 |  |  |
|  | -2.6 | sushi-repeat-containing protein |  |  |
|  | -2.5 | lymphocyte antigen 6 complex, locus G |  |  |
|  | -2.5 | transmembrane protein 120B |  |  |
|  | -2.5 | interferon-stimulated protein |  |  |
|  | -2.5 | expressed sequence AW011738 |  |  |
|  | -2.5 | coiled-coil domain containing 96 |  |  |
|  | -2.5 | Max dimerization protein 3 |  |  |
|  | -2.5 | killer cell lectin-like receptor, subfamily A, member 17 |  |  |
|  | -2.5 | N-acetylglutamate synthase |  |  |
|  | -2.5 | H1.4 linker histone, cluster member |  |  |
|  | -2.5 | fumarylacetoacetate hydrolase domain containing 1 |  |  |
|  | -2.5 | RIKEN cDNA G430095P16 gene |  |  |
|  | -2.5 | solute carrier family 25, member 38 |  |  |
|  | -2.5 | forkhead box H1 |  |  |
|  | -2.5 | calpain 5 |  |  |
|  | -2.5 | mitogen-activated protein kinase kinase kinase kinase 5 |  |  |
|  | -2.5 | predicted gene, 30716 |  |  |
|  | -2.5 | solute carrier family 25 (mitochondrial carrier), member 18 |  |  |
|  | -2.5 | secernin 3 |  |  |
|  | -2.5 | thioredoxin reductase 2 |  |  |
|  | -2.5 | complement component 1, s subcomponent 1 |  |  |
|  | -2.5 | chemokine (C-C motif) receptor-like 2 |  |  |
|  | -2.5 | chitinase 1 (chitotriosidase) |  |  |
|  | -2.5 | RIKEN cDNA 4933431E20 gene |  |  |
|  | -2.5 | leucine rich repeat containing 36 |  |  |
|  | -2.5 | hemoglobin Z, beta-like embryonic chain |  |  |
|  | -2.5 | interferon induced transmembrane protein 7 |  |  |
|  | -2.5 | cysteine-rich secretory protein LCCL domain containing 1 |  |  |
|  | -2.5 | arylsulfatase i |  |  |
|  | -2.5 | myosin, light polypeptide 2, regulatory, cardiac, slow |  |  |
|  | -2.5 | tripartite motif-containing 17 |  |  |
|  | -2.5 | hemoglobin, theta 1A |  |  |
|  | -2.5 | expressed sequence AA986860 |  |  |
|  | -2.5 | ST3 beta-galactoside alpha-2,3-sialyltransferase 6 |  |  |
|  | -2.5 | tubulointerstitial nephritis antigen-like 1 |  |  |
|  | -2.5 | centrosomal protein 76 |  |  |
|  | -2.5 | peroxiredoxin like 2A |  |  |
|  | -2.5 | membrane associated ring-CH-type finger 2 |  |  |
|  | -2.5 | CD24a antigen |  |  |
|  | -2.5 | coxsackie virus and adenovirus receptor |  |  |
|  | -2.5 | early B cell factor 3 |  |  |
|  | -2.5 | immunoglobulin kappa variable 5-48 |  |  |
|  | -2.5 | transmembrane protein 132B |  |  |
|  | -2.5 | shroom family member 3 |  |  |
|  | -2.5 | apolipoprotein L domain containing 1 |  |  |
|  | -2.5 | REC8 meiotic recombination protein |  |  |
|  | -2.5 | ubiquitin specific protease 51 |  |  |
|  | -2.5 | heat shock protein 1-like |  |  |
|  | -2.5 | jun proto-oncogene |  |  |
|  | -2.5 | target of myb1-like 1 (chicken) |  |  |
|  | -2.5 | nuclear factor I/A |  |  |
|  | -2.5 | protoporphyrinogen oxidase |  |  |
|  | -2.5 | cell division cycle 25B |  |  |
|  | -2.5 | H4 clustered histone 6 |  |  |
|  | -2.5 | septin 4 |  |  |
|  | -2.4 | ubiquitin-conjugating enzyme E2L 6 |  |  |
|  | -2.4 | thioesterase superfamily member 7 |  |  |
|  | -2.4 | immunoglobulin heavy variable 13-2 |  |  |
|  | -2.4 | cytoplasmic polyadenylation element binding protein 3 |  |  |
|  | -2.4 | leucine rich repeat containing 10B |  |  |
|  | -2.4 | fibulin 1 |  |  |
|  | -2.4 | 3-hydroxy-3-methylglutaryl-Coenzyme A synthase 2 |  |  |
|  | -2.4 | regulator of cell cycle |  |  |
|  | -2.4 | zinc finger and BTB domain containing 10 |  |  |
|  | -2.4 | H3 clustered histone 4 |  |  |
|  | -2.4 | hydroxyacyl glutathione hydrolase |  |  |
|  | -2.4 | atlastin GTPase 1 |  |  |
|  | -2.4 | WNK lysine deficient protein kinase 4 |  |  |
|  | -2.4 | hemoglobin, beta adult t chain |  |  |
|  | -2.4 | predicted gene 13306 |  |  |
|  | -2.4 | immunoglobulin heavy variable 7-1 |  |  |
|  | -2.4 | hemoglobin alpha, adult chain 1 |  |  |
|  | -2.4 | IBA57 homolog, iron-sulfur cluster assembly |  |  |
|  | -2.4 | immunoglobulin kappa variable 3-12 |  |  |
|  | -2.4 | hemoglobin alpha, adult chain 2 |  |  |
|  | -2.4 | membrane associated ring-CH-type finger 8 |  |  |
|  | -2.4 | glial cell line derived neurotrophic factor family receptor alpha 1 |  |  |
|  | -2.4 | tripartite motif-containing 59 pseudogene |  |  |
|  | -2.4 | immunoglobulin kappa variable 1-110 |  |  |
|  | -2.4 | small integral membrane protein 41 |  |  |
|  | -2.4 | arylformamidase |  |  |
|  | -2.4 | arrestin domain containing 3 |  |  |
|  | -2.4 | stabilin 2 |  |  |
|  | -2.4 | predicted gene, 33148 |  |  |
|  | -2.4 | complement component 4B (Chido blood group) |  |  |
|  | -2.4 | antigen identified by monoclonal antibody Ki 67 |  |  |
|  | -2.4 | immunoglobulin kappa chain variable 4-90 |  |  |
|  | -2.4 | ribonucleotide reductase M2 |  |  |
|  | -2.4 | phospholipase D family, member 6 |  |  |
|  | -2.4 | thymidine kinase 1 |  |  |
|  | -2.4 | WD40 repeat domain 95 |  |  |
|  | -2.4 | H2B clustered histone 15 |  |  |
|  | -2.4 | hepsin |  |  |
|  | -2.4 | biliverdin reductase B (flavin reductase (NADPH)) |  |  |
|  | -2.4 | RAD23 homolog A, nucleotide excision repair protein |  |  |
|  | -2.4 | nuclear receptor subfamily 4, group A, member 2 |  |  |
|  | -2.4 | stomatin |  |  |
|  | -2.4 | PH domain and leucine rich repeat protein phosphatase 2 |  |  |
|  | -2.4 | cyclin E1 |  |  |
|  | -2.3 | SPARC-like 1 |  |  |
|  | -2.3 | solute carrier family 43, member 3 |  |  |
|  | -2.3 | regulator of G-protein signaling 12 |  |  |
|  | -2.3 | RIKEN cDNA C130074G19 gene |  |  |
|  | -2.3 | H2B clustered histone 1 |  |  |
|  | -2.3 | 5'-nucleotidase, cytosolic III |  |  |
|  | -2.3 | signal peptide peptidase like 2B |  |  |
|  | -2.3 | sodium channel, type IV, beta |  |  |
|  | -2.3 | ADP-ribosyltransferase 3 |  |  |
|  | -2.3 | cerebral cavernous malformation 2-like |  |  |
|  | -2.3 | immunoglobulin heavy variable 1-77 |  |  |
|  | -2.3 | killer cell lectin-like receptor subfamily B member 1C |  |  |
|  | -2.3 | multiple inositol polyphosphate histidine phosphatase 1 pseudogene |  |  |
|  | -2.3 | H4 clustered histone 12 |  |  |
|  | -2.3 | tubulin polymerization promoting protein |  |  |
|  | -2.3 | tripartite motif-containing 59 |  |  |
|  | -2.3 | zinc finger protein, multitype 1 |  |  |
|  | -2.3 | bone morphogenetic protein 4 |  |  |
|  | -2.3 | immunoglobulin kappa variable 8-28 |  |  |
|  | -2.3 | membrane frizzled-related protein |  |  |
|  | -2.3 | DEP domain containing 1a |  |  |
|  | -2.3 | H2A clustered histone 6 |  |  |
|  | -2.3 | high mobility group box 3 |  |  |
|  | -2.3 | cytoglobin |  |  |
|  | -2.3 | inter-alpha-trypsin inhibitor, heavy chain 5 |  |  |
|  | -2.3 | sperm associated antigen 6-like |  |  |
|  | -2.3 | PH domain containing endocytic trafficking adaptor 2 |  |  |
|  | -2.3 | zinc finger protein 367 |  |  |
|  | -2.3 | pleckstrin and Sec7 domain containing |  |  |
|  | -2.3 | poly(A) binding protein, cytoplasmic 4 |  |  |
|  | -2.3 | CD300 molecule like family member G |  |  |
|  | -2.3 | receptor accessory protein 6 |  |  |
|  | -2.3 | erythrocyte membrane protein band 4.1 |  |  |
|  | -2.3 | family with sequence similarity 220, member A |  |  |
|  | -2.3 | olfactomedin-like 2A |  |  |
|  | -2.3 | autophagy related 4A, cysteine peptidase |  |  |
|  | -2.3 | H2B clustered histone 22 |  |  |
|  | -2.3 | high mobility group box 2 pseudogene |  |  |
|  | -2.3 | immunoglobulin kappa variable 2-112 |  |  |
|  | -2.3 | iron-sulfur cluster assembly 1 |  |  |
|  | -2.3 | family with sequence similarity 220, member A |  |  |
|  | -2.3 | DnaJ heat shock protein family (Hsp40) member B4 |  |  |
|  | -2.3 | cDNA sequence AK157302 |  |  |
|  | -2.3 | natural killer cell group 7 sequence |  |  |
|  | -2.3 | poly A binding protein, cytoplasmic 4 pseudogene |  |  |
|  | -2.3 | ABI family member 3 binding protein |  |  |
|  | -2.3 | immunoglobulin kappa chain variable 4-61 |  |  |
|  | -2.3 | kelch-like 12 |  |  |
|  | -2.3 | sterile alpha motif domain containing 14 |  |  |
|  | -2.3 | endonuclease domain containing 1 |  |  |
|  | -2.3 | coagulation factor VIII |  |  |
|  | -2.3 | STE20-related kinase adaptor beta |  |  |
|  | -2.3 | heat shock protein 1B |  |  |
|  | -2.3 | Rho guanine nucleotide exchange factor (GEF) 25 |  |  |
|  | -2.3 | vitrin |  |  |
|  | -2.2 | retinoblastoma binding protein 4 pseudogene |  |  |
|  | -2.2 | netrin 4 |  |  |
|  | -2.2 | RIKEN cDNA 1810006J02 gene |  |  |
|  | -2.2 | mitochondrial fission regulator 1 |  |  |
|  | -2.2 | killer cell lectin-like receptor, subfamily A, member 1 |  |  |
|  | -2.2 | H3 clustered histone 11 |  |  |
|  | -2.2 | H2B clustered histone 12 |  |  |
|  | -2.2 | ubiquitin-conjugating enzyme E2C |  |  |
|  | -2.2 | immunoglobulin heavy constant gamma 2B |  |  |
|  | -2.2 | H4 clustered histone 8 |  |  |
|  | -2.2 | matrix metallopeptidase 28 (epilysin) |  |  |
|  | -2.2 | scavenger receptor class A, member 5 |  |  |
|  | -2.2 | zymogen granule protein 16 |  |  |
|  | -2.2 | hemoglobin, beta adult major chain |  |  |
|  | -2.2 | multiple inositol polyphosphate histidine phosphatase 1 |  |  |
|  | -2.2 | capping protein inhibiting regulator of actin |  |  |
|  | -2.2 | aldehyde dehydrogenase 3 family, member B2 |  |  |
|  | -2.2 | Kruppel-like factor 3 (basic) |  |  |
|  | -2.2 | glutathione peroxidase 1 |  |  |
|  | -2.2 | HECT domain and ankyrin repeat containing, E3 ubiquitin protein ligase 1 |  |  |
|  | -2.2 | salt inducible kinase 1 |  |  |
|  | -2.2 | transmembrane protein 44 |  |  |
|  | -2.2 | poly(A)-binding protein, cytoplasmic pseudogene |  |  |
|  | -2.2 | Fas ligand (TNF superfamily, member 6) |  |  |
|  | -2.2 | BCL2-like 1 |  |  |
|  | -2.2 | kinesin family member 18A |  |  |
|  | -2.2 | cell division control protein 6 homolog |  |  |
|  | -2.2 | USH1 protein network component harmonin binding protein 1 |  |  |
|  | -2.2 | immunoglobulin kappa variable 12-46 |  |  |
|  | -2.2 | immunoglobulin heavy constant gamma 2C |  |  |
|  | -2.2 | makorin, ring finger protein 1, pseudogene 1 |  |  |
|  | -2.2 | tumor necrosis factor receptor superfamily, member 19 |  |  |
|  | -2.2 | immunoglobulin kappa variable 4-69 |  |  |
|  | -2.2 | carbohydrate sulfotransferase 10 |  |  |
|  | -2.2 | RNA binding motif protein 38 |  |  |
|  | -2.2 | makorin, ring finger protein, 1 |  |  |
|  | -2.2 | predicted gene 15972 |  |  |
|  | -2.2 | chemokine (C-C motif) receptor 8 |  |  |
|  | -2.2 | family with sequence similarity 205, member A2 |  |  |
|  | -2.2 | elastase, neutrophil expressed |  |  |
|  | -2.2 | keratin 83 |  |  |
|  | -2.2 | regulator of G-protein signaling 2 |  |  |
|  | -2.2 | phosphate cytidylyltransferase 1, choline, beta isoform |  |  |
|  | -2.2 | proteinase 3 |  |  |
|  | -2.2 | centromere protein F |  |  |
|  | -2.1 | sperm associated antigen 5 |  |  |
|  | -2.1 | growth factor independent 1B |  |  |
|  | -2.1 | Rho GTPase activating protein 23 |  |  |
|  | -2.1 | calcium regulated heat stable protein 1 |  |  |
|  | -2.1 | eukaryotic translation initiation factor 2 alpha kinase 1 |  |  |
|  | -2.1 | H3 clustered histone 8 |  |  |
|  | -2.1 | acyl-CoA synthetase long-chain family member 1 |  |  |
|  | -2.1 | HECT domain E3 ubiquitin protein ligase 4 |  |  |
|  | -2.1 | adenylate cyclase 10 |  |  |
|  | -2.1 | immunoglobulin kappa variable 4-80 |  |  |
|  | -2.1 | SH3 and multiple ankyrin repeat domains 3 |  |  |
|  | -2.1 | family with sequence similarity 169, member A |  |  |
|  | -2.1 | ChaC, cation transport regulator 2 |  |  |
|  | -2.1 | F-box protein 30 |  |  |
|  | -2.1 | immunoglobulin kappa variable 4-58 |  |  |
|  | -2.1 | killer cell lectin-like receptor subfamily B member 1A |  |  |
|  | -2.1 | immunoglobulin kappa variable 14-130 |  |  |
|  | -2.1 | H3 clustered histone 10 |  |  |
|  | -2.1 | immunoglobulin kappa variable 4-63 |  |  |
|  | -2.1 | SHC (Src homology 2 domain containing) family, member 4 |  |  |
|  | -2.1 | small nucleolar RNA, H/ACA box 28 |  |  |
|  | -2.1 | kinesin family member 18B |  |  |
|  | -2.1 | abnormal spindle microtubule assembly |  |  |
|  | -2.1 | H2A clustered histone 4 |  |  |
|  | -2.1 | predicted gene, 31828 |  |  |
|  | -2.1 | ankyrin repeat domain 6 |  |  |
|  | -2.1 | immunoglobulin heavy variable 7-3 |  |  |
|  | -2.1 | ornithine decarboxylase, structural 1 |  |  |
|  | -2.1 | autophagy related 4A, pseudogene |  |  |
|  | -2.1 | frequently rearranged in advanced T cell lymphomas 2 |  |  |
|  | -2.1 | phosphatidylinositol-4-phosphate 5-kinase, type 1 beta |  |  |
|  | -2.1 | eosinophil-associated, ribonuclease A family, member 1 |  |  |
|  | -2.1 | baculoviral IAP repeat-containing 5 |  |  |
|  | -2.1 | predicted gene 10030 |  |  |
|  | -2.1 | dishevelled-binding antagonist of beta-catenin 1 |  |  |
|  | -2.1 | cyclin B2 |  |  |
|  | -2.1 | Bloodlinc, erythroid developmental long intergenic non-protein coding transcript |  |  |
|  | -2.1 | nucleolar and spindle associated protein 1 |  |  |
|  | -2.1 | amine oxidase, copper containing 2 (retina-specific) |  |  |
|  | -2.1 | zinc finger and SCAN domain containing 18 |  |  |
|  | -2.1 | ethanolamine kinase 2 |  |  |
|  | -2.1 | immunoglobulin kappa variable 4-79 |  |  |
|  | -2.1 | family with sequence similarity 241, member A |  |  |
|  | -2.1 | cyclin dependent kinase inhibitor 2C |  |  |
|  | -2.1 | H2A clustered histone 11 |  |  |
|  | -2.1 | H2B clustered histone 11 |  |  |
|  | -2.1 | Rhesus blood group-associated C glycoprotein |  |  |
|  | -2.1 | RIKEN cDNA 2500002B13 gene |  |  |
|  | -2.1 | ATP-binding cassette, sub-family B (MDR/TAP), member 9 |  |  |
|  | -2.1 | unc-5 family C-terminal like |  |  |
|  | -2.1 | immunoglobulin heavy variable 1-78 |  |  |
|  | -2.1 | protein tyrosine phosphatase 4a3 |  |  |
|  | -2.1 | Spi-C transcription factor (Spi-1/PU.1 related) |  |  |
|  | -2.1 | RIKEN cDNA 9830166K06 gene |  |  |
|  | -2.1 | RAB30, member RAS oncogene family |  |  |
|  | -2.0 | fizzy and cell division cycle 20 related 1 |  |  |
|  | -2.0 | H2A.X variant histone |  |  |
|  | -2.0 | H3 clustered histone 3 |  |  |
|  | -2.0 | carbonic anhydrase 3 |  |  |
|  | -2.0 | H2A clustered histone 13 |  |  |
|  | -2.0 | lumican |  |  |
|  | -2.0 | adrenergic receptor, beta 2 |  |  |
|  | -2.0 | keratin 81 |  |  |
|  | -2.0 | predicted gene 10790 |  |  |
|  | -2.0 | immunoglobulin kappa joining 1 |  |  |
|  | -2.0 | CD160 antigen |  |  |
|  | -2.0 | PDZK1 interacting protein 1 |  |  |
|  | -2.0 | immunoglobulin kappa chain variable 9-124 |  |  |
|  | -2.0 | interleukin 23, alpha subunit p19 |  |  |
|  | -2.0 | threonyl-tRNA synthetase-like 2 |  |  |
|  | -2.0 | immunoglobulin kappa variable 1-133 |  |  |
|  | -2.0 | RIKEN cDNA 1700086O06 gene |  |  |
|  | -2.0 | H4 clustered histone 14 |  |  |
|  | -2.0 | nocturnin |  |  |
|  | -2.0 | hemoglobin alpha, pseudogene 4 |  |  |
|  | -2.0 | phosphofructokinase, muscle |  |  |
|  | -2.0 | transmembrane protein 231 |  |  |
|  | -2.0 | H2B clustered histone 14 |  |  |
|  | -2.0 | serine (or cysteine) peptidase inhibitor, clade F, member 2 |  |  |
|  | -2.0 | cyclin-dependent kinase inhibitor 3 |  |  |
|  | -2.0 | RIKEN cDNA 4930438A08 gene |  |  |
|  | 2.0 | repulsive guidance molecule family member B |  |  |
|  | 2.0 | protocadherin gamma subfamily B, 7 |  |  |
|  | 2.0 | leucine-rich repeat LGI family, member 4 |  |  |
|  | 2.0 | stearoyl-coenzyme A desaturase 3 |  |  |
|  | 2.0 | collagen, type VI, alpha 3 |  |  |
|  | 2.0 | staufen double-stranded RNA binding protein 2 |  |  |
|  | 2.0 | solute carrier protein family 52, member 3 |  |  |
|  | 2.0 | disco interacting protein 2 homolog C |  |  |
|  | 2.0 | purinergic receptor P2X, ligand-gated ion channel, 7 |  |  |
|  | 2.0 | multiple EGF-like-domains 8 |  |  |
|  | 2.0 | CDC42 effector protein (Rho GTPase binding) 5 |  |  |
|  | 2.0 | solute carrier family 15, member 3 |  |  |
|  | 2.0 | multiple coagulation factor deficiency 2 |  |  |
|  | 2.0 | solute carrier family 14 (urea transporter), member 2 |  |  |
|  | 2.0 | phosphofructokinase, liver, B-type |  |  |
|  | 2.0 | G protein-coupled receptor 137B |  |  |
|  | 2.0 | RIKEN cDNA 4632428C04 gene |  |  |
|  | 2.0 | CKLF-like MARVEL transmembrane domain containing 3 |  |  |
|  | 2.0 | ubiquitin specific peptidase 40 |  |  |
|  | 2.0 | Rho GTPase activating protein 22 |  |  |
|  | 2.0 | neuralized E3 ubiquitin protein ligase 1A |  |  |
|  | 2.0 | hypoxia inducible lipid droplet associated |  |  |
|  | 2.0 | vitamin D (1,25-dihydroxyvitamin D3) receptor |  |  |
|  | 2.0 | sphingosine-1-phosphate receptor 3 |  |  |
|  | 2.0 | dorsal inhibitory axon guidance protein |  |  |
|  | 2.0 | Fc fragment of IgG receptor and transporter |  |  |
|  | 2.0 | glutamine fructose-6-phosphate transaminase 2 |  |  |
|  | 2.0 | small G protein signaling modulator 1 |  |  |
|  | 2.0 | hepatocyte growth factor |  |  |
|  | 2.0 | versican | |  |
|  | 2.0 | calcium channel, voltage-dependent, beta 2 subunit |  |  |
|  | 2.1 | RIKEN cDNA 3300002I08 gene |  |  |
|  | 2.1 | sushi, von Willebrand factor type A, EGF and pentraxin domain containing 1 |  |  |
|  | 2.1 | solute carrier family 13 (sodium-dependent dicarboxylate transporter), member 2 |  |  |
|  | 2.1 | predicted gene 6566 |  |  |
|  | 2.1 | shisa family member 8 |  |  |
|  | 2.1 | NIPA-like domain containing 1 |  |  |
|  | 2.1 | peroxisomal biogenesis factor 26 |  |  |
|  | 2.1 | toll-like receptor 1 |  |  |
|  | 2.1 | cytochrome P450, family 1, subfamily a, polypeptide 1 |  |  |
|  | 2.1 | pleckstrin homology domain containing, family G (with RhoGef domain) member 1 |  |  |
|  | 2.1 | selectin, platelet |  |  |
|  | 2.1 | transmembrane protein 236 |  |  |
|  | 2.1 | secreted frizzled-related protein 1 |  |  |
|  | 2.1 | phospholipid transfer protein |  |  |
|  | 2.1 | ret proto-oncogene |  |  |
|  | 2.1 | mucin 1, transmembrane |  |  |
|  | 2.1 | IgA inducing protein |  |  |
|  | 2.1 | C-type lectin domain family 4, member a3 |  |  |
|  | 2.1 | hyaluronoglucosaminidase 1 |  |  |
|  | 2.1 | aldo-keto reductase family 1, member C12 |  |  |
|  | 2.1 | myeloid/lymphoid or mixed-lineage leukemia; translocated to, 11 |  |  |
|  | 2.1 | transmembrane protein 158 |  |  |
|  | 2.1 | macrophage migration inhibitory factor (glycosylation-inhibiting factor) |  |  |
|  | 2.1 | 5-hydroxytryptamine (serotonin) receptor 1B |  |  |
|  | 2.1 | frizzled class receptor 7 |  |  |
|  | 2.1 | hypoxia inducible factor 1, alpha subunit |  |  |
|  | 2.1 | C-type lectin domain family 2, member L |  |  |
|  | 2.1 | Rho GTPase activating protein 6 |  |  |
|  | 2.1 | basic helix-loop-helix family, member e40 |  |  |
|  | 2.1 | spermatogenesis and centriole associated 1 |  |  |
|  | 2.1 | SECIS binding protein 2-like |  |  |
|  | 2.1 | proline and serine rich 2 |  |  |
|  | 2.1 | polycystic kidney disease 1 like 2 |  |  |
|  | 2.1 | C-type lectin domain family 4, member a1 |  |  |
|  | 2.1 | ankyrin repeat domain 24 |  |  |
|  | 2.1 | WD repeat domain 19 |  |  |
|  | 2.1 | coiled-coil domain containing 80 |  |  |
|  | 2.1 | myocardin related transcription factor B |  |  |
|  | 2.1 | testis expressed gene 14 |  |  |
|  | 2.1 | melanophilin |  |  |
|  | 2.1 | coiled-coil and C2 domain containing 2B |  |  |
|  | 2.1 | clathrin binding box of aftiphilin containing 1 |  |  |
|  | 2.1 | ADAMTS-like 5 |  |  |
|  | 2.1 | growth arrest and DNA-damage-inducible 45 gamma |  |  |
|  | 2.1 | G protein-coupled receptor 82 |  |  |
|  | 2.1 | paired related homeobox 1 |  |  |
|  | 2.1 | transmembrane protein 273 |  |  |
|  | 2.1 | potassium channel tetramerisation domain containing 21 |  |  |
|  | 2.1 | dehydrogenase/reductase (SDR family) member 3 |  |  |
|  | 2.1 | sema domain, transmembrane domain (TM), and cytoplasmic domain, (semaphorin) 6B |  |  |
|  | 2.1 | arachidonate 8-lipoxygenase |  |  |
|  | 2.1 | Rho-related BTB domain containing 1 |  |  |
|  | 2.1 | cytokine-dependent hematopoietic cell linker |  |  |
|  | 2.1 | thromboxane A synthase 1, platelet |  |  |
|  | 2.1 | polycystin 2, transient receptor potential cation channel |  |  |
|  | 2.1 | syndecan 3 | |  |
|  | 2.1 | tubulin, alpha 8 |  |  |
|  | 2.1 | potassium inwardly-rectifying channel, subfamily K, member 6 |  |  |
|  | 2.1 | regulator of sex-limitation candidate 18 |  |  |
|  | 2.1 | sushi-repeat-containing protein, X-linked 2 |  |  |
|  | 2.1 | uncharacterized LOC102637806 |  |  |
|  | 2.1 | N-acetylglucosamine kinase |  |  |
|  | 2.1 | transforming growth factor, beta induced |  |  |
|  | 2.1 | solute carrier family 66 member 1 |  |  |
|  | 2.1 | potassium voltage-gated channel, subfamily F, member 1 |  |  |
|  | 2.1 | ring finger protein, transmembrane 2 |  |  |
|  | 2.1 | TRPM8 channel-associated factor 1 |  |  |
|  | 2.2 | transmembrane protein 202 |  |  |
|  | 2.2 | FYN binding protein 2 |  |  |
|  | 2.2 | latent transforming growth factor beta binding protein 1 |  |  |
|  | 2.2 | proviral integration site 3 |  |  |
|  | 2.2 | germinal center associated, signaling and motility |  |  |
|  | 2.2 | NLR family, pyrin domain containing 1C, pseudogene |  |  |
|  | 2.2 | TBC1 domain family, member 9 |  |  |
|  | 2.2 | epidermal growth factor receptor pathway substrate 8 |  |  |
|  | 2.2 | guanine nucleotide binding protein, alpha z subunit |  |  |
|  | 2.2 | tetraspanin 3 |  |  |
|  | 2.2 | jun proto-oncogene, opposite strand |  |  |
|  | 2.2 | U1 small nuclear ribonucleoprotein 1C pseudogene |  |  |
|  | 2.2 | phosphatidylinositol-specific phospholipase C, X domain containing 1 |  |  |
|  | 2.2 | epithelial membrane protein 2 |  |  |
|  | 2.2 | collagen, type V, alpha 2 |  |  |
|  | 2.2 | coatomer protein complex, subunit zeta 2 |  |  |
|  | 2.2 | transient receptor potential cation channel, subfamily C, member 6 |  |  |
|  | 2.2 | transmembrane protein 51 |  |  |
|  | 2.2 | Fc receptor, IgG, high affinity I |  |  |
|  | 2.2 | tumor necrosis factor (ligand) superfamily, member 4 |  |  |
|  | 2.2 | FGGY carbohydrate kinase domain containing |  |  |
|  | 2.2 | mitogen-activated protein kinase kinase kinase 12 |  |  |
|  | 2.2 | caveolin 1, caveolae protein |  |  |
|  | 2.2 | MyoD family inhibitor domain containing |  |  |
|  | 2.2 | predicted gene, 21814 |  |  |
|  | 2.2 | interleukin 6 signal transducer |  |  |
|  | 2.2 | protocadherin 19 |  |  |
|  | 2.2 | solute carrier family 27 (fatty acid transporter), member 1 |  |  |
|  | 2.2 | paternally expressed 10 |  |  |
|  | 2.2 | ectonucleoside triphosphate diphosphohydrolase 3 |  |  |
|  | 2.2 | CDK5 and Abl enzyme substrate 1 |  |  |
|  | 2.2 | predicted gene, 21451 |  |  |
|  | 2.2 | exocyst complex component 3-like 4 |  |  |
|  | 2.2 | RIKEN cDNA 4930556M19 gene |  |  |
|  | 2.2 | atypical chemokine receptor 2 |  |  |
|  | 2.2 | phytanoyl-CoA dioxygenase domain containing 1 |  |  |
|  | 2.2 | SH3 and multiple ankyrin repeat domains 1 |  |  |
|  | 2.2 | dishevelled-binding antagonist of beta-catenin 3 |  |  |
|  | 2.2 | tumor necrosis factor receptor superfamily, member 23 |  |  |
|  | 2.2 | interleukin 1 receptor antagonist |  |  |
|  | 2.2 | transient receptor potential cation channel, subfamily V, member 4 |  |  |
|  | 2.2 | collagen, type XV, alpha 1 |  |  |
|  | 2.2 | Rho GTPase activating protein 31 |  |  |
|  | 2.2 | mast cell protease 8 |  |  |
|  | 2.2 | proliferation and apoptosis adaptor protein 15A |  |  |
|  | 2.2 | Fc receptor-like 5 |  |  |
|  | 2.2 | SLAM family member 8 |  |  |
|  | 2.2 | ankyrin 3, epithelial |  |  |
|  | 2.2 | G protein-coupled receptor, family C, group 5, member C |  |  |
|  | 2.2 | SAM and SH3 domain containing 1 |  |  |
|  | 2.2 | ST6 (alpha-N-acetyl-neuraminyl-2,3-beta-galactosyl-1,3)-N-acetylgalactosaminide alpha-2,6-sialyltransferase 5 |  |  |
|  | 2.2 | epithelial membrane protein 1 |  |  |
|  | 2.2 | lectin, galactose binding, soluble 1 |  |  |
|  | 2.3 | G protein-coupled receptor 85 |  |  |
|  | 2.3 | PRELI domain containing 2 |  |  |
|  | 2.3 | UDP-Gal:betaGlcNAc beta 1,4- galactosyltransferase, polypeptide 2 |  |  |
|  | 2.3 | shroom family member 4 |  |  |
|  | 2.3 | RIKEN cDNA 2810429I04 gene |  |  |
|  | 2.3 | lysyl oxidase-like 3 |  |  |
|  | 2.3 | peptidyl-tRNA hydrolase 1 homolog |  |  |
|  | 2.3 | potassium inwardly-rectifying channel, subfamily J, member 5 |  |  |
|  | 2.3 | milk fat globule EGF and factor V/VIII domain containing |  |  |
|  | 2.3 | coagulation factor II (thrombin) receptor |  |  |
|  | 2.3 | melanoregulin |  |  |
|  | 2.3 | laminin, alpha 5 |  |  |
|  | 2.3 | dipeptidylpeptidase 7 |  |  |
|  | 2.3 | vascular endothelial growth factor A |  |  |
|  | 2.3 | lipoprotein lipase |  |  |
|  | 2.3 | glycosyltransferase 28 domain containing 2 |  |  |
|  | 2.3 | LIM domain only 7 |  |  |
|  | 2.3 | G protein-coupled receptor 35 |  |  |
|  | 2.3 | thyroid hormone receptor beta |  |  |
|  | 2.3 | predicted gene, 34643 |  |  |
|  | 2.3 | protein arginine N-methyltransferase 2 |  |  |
|  | 2.3 | brain-specific angiogenesis inhibitor 1-associated protein 2 |  |  |
|  | 2.3 | PR domain containing 1, with ZNF domain |  |  |
|  | 2.3 | cathepsin Z | |  |
|  | 2.3 | calcium channel, voltage-dependent, L type, alpha 1D subunit |  |  |
|  | 2.3 | LanC lantibiotic synthetase component C-like 3 (bacterial) |  |  |
|  | 2.3 | RIKEN cDNA A530088E08 gene |  |  |
|  | 2.3 | meningioma 1 |  |  |
|  | 2.3 | peripheral myelin protein 22 |  |  |
|  | 2.3 | sphingomyelin phosphodiesterase 5 |  |  |
|  | 2.3 | huntingtin interacting protein 1 |  |  |
|  | 2.3 | germ cell associated 1 |  |  |
|  | 2.3 | MAS-related GPR, member E |  |  |
|  | 2.3 | C-type lectin domain family 7, member a |  |  |
|  | 2.3 | predicted gene 11767 |  |  |
|  | 2.3 | angiopoietin 2 |  |  |
|  | 2.3 | transglutaminase 1, K polypeptide |  |  |
|  | 2.3 | CD209c antigen |  |  |
|  | 2.3 | predicted gene 1968 |  |  |
|  | 2.3 | pellino 3 | |  |
|  | 2.3 | C1q and tumor necrosis factor related protein 6 |  |  |
|  | 2.3 | Sec24 related gene family, member D (S. cerevisiae) |  |  |
|  | 2.3 | chemokine (C-X-C motif) ligand 5 |  |  |
|  | 2.3 | solute carrier family 25 (mitochondrial thiamine pyrophosphate carrier), member 19 |  |  |
|  | 2.3 | LIF receptor alpha |  |  |
|  | 2.3 | SEC14 and spectrin domains 1 pseudogene |  |  |
|  | 2.3 | receptor-associated protein of the synapse |  |  |
|  | 2.3 | integrin beta 3 |  |  |
|  | 2.3 | chondroitin sulfate synthase 3 |  |  |
|  | 2.3 | NLR family, pyrin domain containing 1B |  |  |
|  | 2.3 | insulin-like growth factor 2 mRNA binding protein 2 |  |  |
|  | 2.3 | low density lipoprotein receptor-related protein 1 |  |  |
|  | 2.3 | proline rich 36 |  |  |
|  | 2.3 | Rab interacting lysosomal protein |  |  |
|  | 2.3 | BTB and CNC homology 2, opposite strand |  |  |
|  | 2.3 | microRNA 678 |  |  |
|  | 2.3 | adenosine A2b receptor |  |  |
|  | 2.3 | hedgehog acyltransferase |  |  |
|  | 2.4 | UDP-Gal:betaGlcNAc beta 1,4-galactosyltransferase, polypeptide 5 |  |  |
|  | 2.4 | predicted gene, 17767 |  |  |
|  | 2.4 | integrin beta 5 |  |  |
|  | 2.4 | BEN domain containing 6 |  |  |
|  | 2.4 | purinergic receptor P2X, ligand-gated ion channel 4 |  |  |
|  | 2.4 | MAS-related GPR, member A4 pseudogene |  |  |
|  | 2.4 | wolframin ER transmembrane glycoprotein |  |  |
|  | 2.4 | poliovirus receptor |  |  |
|  | 2.4 | coagulation factor II (thrombin) receptor-like 2 |  |  |
|  | 2.4 | RIKEN cDNA 4931406B18 gene |  |  |
|  | 2.4 | toll-like receptor 8 |  |  |
|  | 2.4 | nectin cell adhesion molecule 3 |  |  |
|  | 2.4 | RAB27B, member RAS oncogene family |  |  |
|  | 2.4 | leucine zipper, putative tumor suppressor 1 |  |  |
|  | 2.4 | megakaryocyte-associated tyrosine kinase |  |  |
|  | 2.4 | v-myc avian myelocytomatosis viral related oncogene, neuroblastoma derived |  |  |
|  | 2.4 | G protein coupled receptor 15 ligand |  |  |
|  | 2.4 | adrenomedullin 2 |  |  |
|  | 2.4 | tsukushi, small leucine rich proteoglycan |  |  |
|  | 2.4 | solute carrier family 25, member 43 |  |  |
|  | 2.4 | NIMA (never in mitosis gene a)- related kinase 10 |  |  |
|  | 2.4 | EP300 interacting inhibitor of differentiation 2 |  |  |
|  | 2.4 | ADP-ribosylation factor-like 4C |  |  |
|  | 2.4 | interferon gamma inducible protein 30 |  |  |
|  | 2.4 | predicted gene 11110 |  |  |
|  | 2.4 | NPC intracellular cholesterol transporter 1 |  |  |
|  | 2.4 | colony stimulating factor 2 receptor, beta, low-affinity (granulocyte-macrophage) |  |  |
|  | 2.4 | CNDP dipeptidase 2 (metallopeptidase M20 family) |  |  |
|  | 2.4 | sodium channel, voltage-gated, type I, beta |  |  |
|  | 2.4 | stearoyl-coenzyme A desaturase 4 |  |  |
|  | 2.4 | centrosomal protein 126 |  |  |
|  | 2.4 | microtubule associated monooxygenase, calponin and LIM domain containing 2 |  |  |
|  | 2.4 | shootin 1 | |  |
|  | 2.4 | lymphocyte antigen 6 complex, locus I |  |  |
|  | 2.4 | protein kinase inhibitor beta, cAMP dependent, testis specific |  |  |
|  | 2.4 | kynureninase |  |  |
|  | 2.4 | PEAK1 related kinase activating pseudokinase 1 |  |  |
|  | 2.4 | ankyrin repeat domain 66 |  |  |
|  | 2.4 | myocyte enhancer factor 2B |  |  |
|  | 2.4 | chemokine (C-C motif) ligand 6 |  |  |
|  | 2.4 | solute carrier family 12, member 7 |  |  |
|  | 2.4 | prostaglandin E receptor 3 (subtype EP3) |  |  |
|  | 2.4 | procollagen lysine, 2-oxoglutarate 5-dioxygenase 2 |  |  |
|  | 2.4 | membrane associated ring-CH-type finger 9 |  |  |
|  | 2.4 | RIKEN cDNA E230016K23 gene |  |  |
|  | 2.4 | heparan sulfate 6-O-sulfotransferase 2 |  |  |
|  | 2.4 | S100 calcium binding protein A7A |  |  |
|  | 2.4 | cytochrome P450, family 1, subfamily b, polypeptide 1 |  |  |
|  | 2.4 | fibronectin 1 |  |  |
|  | 2.4 | phosphatidylinositol glycan anchor biosynthesis, class Z |  |  |
|  | 2.5 | ATP-binding cassette, sub-family C (CFTR/MRP), member 3 |  |  |
|  | 2.5 | G protein-coupled receptor 55 |  |  |
|  | 2.5 | alpha-N-acetylglucosaminidase (Sanfilippo disease IIIB) |  |  |
|  | 2.5 | bone morphogenetic protein 8a |  |  |
|  | 2.5 | RIKEN cDNA E230029C05 gene |  |  |
|  | 2.5 | RIPOR family member 3 |  |  |
|  | 2.5 | solute carrier family 37 (glycerol-3-phosphate transporter), member 2 |  |  |
|  | 2.5 | Rho GTPase activating protein 27, opposite strand 3 |  |  |
|  | 2.5 | a disintegrin-like and metallopeptidase (reprolysin type) with thrombospondin type 1 motif, 9 |  |  |
|  | 2.5 | protocadherin beta 22 |  |  |
|  | 2.5 | mevalonate (diphospho) decarboxylase |  |  |
|  | 2.5 | zinc finger protein 457 |  |  |
|  | 2.5 | collagen, type III, alpha 1 |  |  |
|  | 2.5 | A kinase (PRKA) anchor protein 6 |  |  |
|  | 2.5 | coiled-coil domain containing 162 |  |  |
|  | 2.5 | HemK methyltransferase family member 1 |  |  |
|  | 2.5 | predicted gene, 33318 |  |  |
|  | 2.5 | inositol 1,4,5-triphosphate receptor associated 1 |  |  |
|  | 2.5 | angiogenin, ribonuclease, RNase A family, 5 |  |  |
|  | 2.5 | colony stimulating factor 1 receptor |  |  |
|  | 2.5 | zinc finger protein 57 |  |  |
|  | 2.5 | class II transactivator |  |  |
|  | 2.5 | oncoprotein induced transcript 3 |  |  |
|  | 2.5 | transmembrane protein 215 |  |  |
|  | 2.5 | CD274 antigen |  |  |
|  | 2.5 | tumor necrosis factor receptor superfamily, member 11a, NFKB activator |  |  |
|  | 2.5 | protein kinase, AMP-activated, alpha 2 catalytic subunit |  |  |
|  | 2.5 | solute carrier family 38, member 7 |  |  |
|  | 2.5 | microRNA 705 |  |  |
|  | 2.5 | solute carrier family 9 (sodium/hydrogen exchanger), member 9 |  |  |
|  | 2.5 | WD repeat and FYVE domain containing 2 |  |  |
|  | 2.5 | noggin | |  |
|  | 2.5 | unc-5 netrin receptor B |  |  |
|  | 2.5 | SLP adaptor and CSK interacting membrane protein |  |  |
|  | 2.5 | sorting nexin 29 |  |  |
|  | 2.5 | solute carrier family 4, sodium bicarbonate transporter-like, member 11 |  |  |
|  | 2.5 | RIKEN cDNA D630039A03 gene |  |  |
|  | 2.5 | tubulin, beta 6 class V |  |  |
|  | 2.5 | solute carrier family 24, member 5 |  |  |
|  | 2.5 | adenosine A3 receptor |  |  |
|  | 2.5 | zinc finger protein 532 |  |  |
|  | 2.5 | adrenergic receptor, alpha 2a |  |  |
|  | 2.5 | serine (or cysteine) peptidase inhibitor, clade B, member 2 |  |  |
|  | 2.5 | RIKEN cDNA A930009A15 gene |  |  |
|  | 2.5 | purinergic receptor P2Y, G-protein coupled 12 |  |  |
|  | 2.5 | KDEL (Lys-Asp-Glu-Leu) endoplasmic reticulum protein retention receptor 3 |  |  |
|  | 2.5 | Rho GTPase activating protein 28 |  |  |
|  | 2.5 | N-ethylmaleimide sensitive fusion protein attachment protein beta |  |  |
|  | 2.6 | fibroblast growth factor receptor 1 |  |  |
|  | 2.6 | RAB19, member RAS oncogene family |  |  |
|  | 2.6 | solute carrier family 7 (cationic amino acid transporter, y+ system), member 11 |  |  |
|  | 2.6 | predicted gene, 21761 |  |  |
|  | 2.6 | growth arrest specific 6 |  |  |
|  | 2.6 | alcohol dehydrogenase, iron containing, 1 |  |  |
|  | 2.6 | adhesion G protein-coupled receptor E1 |  |  |
|  | 2.6 | protocadherin 1 |  |  |
|  | 2.6 | solute carrier family 41, member 2 |  |  |
|  | 2.6 | prostaglandin D2 synthase (brain) |  |  |
|  | 2.6 | stabilizer of axonemal microtubules 2 |  |  |
|  | 2.6 | glutamine repeat protein 1 |  |  |
|  | 2.6 | solute carrier family 35, member D3 |  |  |
|  | 2.6 | vesicle amine transport 1 |  |  |
|  | 2.6 | mucin 20 | |  |
|  | 2.6 | plasminogen activator, tissue |  |  |
|  | 2.6 | cysteine-rich with EGF-like domains 1 |  |  |
|  | 2.6 | interleukin 1 receptor, type I |  |  |
|  | 2.6 | plexin D1 | |  |
|  | 2.6 | sprouty-related EVH1 domain containing 3 |  |  |
|  | 2.6 | LIM domain containing preferred translocation partner in lipoma |  |  |
|  | 2.6 | guanylate cyclase 2g |  |  |
|  | 2.6 | alpha-kinase 2 |  |  |
|  | 2.6 | dystrobrevin alpha |  |  |
|  | 2.6 | UDP-GlcNAc:betaGal beta-1,3-N-acetylglucosaminyltransferase 9 |  |  |
|  | 2.6 | colony stimulating factor 1 (macrophage) |  |  |
|  | 2.6 | hes family bHLH transcription factor 5 |  |  |
|  | 2.6 | microRNA 7676-2 |  |  |
|  | 2.6 | hydroxyacid oxidase 1, liver |  |  |
|  | 2.6 | serine peptidase inhibitor, Kazal type 2 |  |  |
|  | 2.6 | mcf.2 transforming sequence-like |  |  |
|  | 2.6 | RAB11 family interacting protein 5 (class I) |  |  |
|  | 2.6 | transmembrane protein with EGF-like and two follistatin-like domains 1 |  |  |
|  | 2.6 | solute carrier family 1 (glial high affinity glutamate transporter), member 2 |  |  |
|  | 2.6 | RPA1 related single stranded DNA binding protein, X-linked |  |  |
|  | 2.6 | F-box and leucine-rich repeat protein 2 |  |  |
|  | 2.6 | polypeptide N-acetylgalactosaminyltransferase 6 |  |  |
|  | 2.6 | nuclear protein transcription regulator 1 |  |  |
|  | 2.6 | solute carrier family 39 (zinc transporter), member 14 |  |  |
|  | 2.7 | guanylate cyclase 1, soluble, beta 1 |  |  |
|  | 2.7 | protein C receptor, endothelial |  |  |
|  | 2.7 | polymeric immunoglobulin receptor |  |  |
|  | 2.7 | RIKEN cDNA 2010016I18 gene |  |  |
|  | 2.7 | plasminogen activator, urokinase |  |  |
|  | 2.7 | RIKEN cDNA A930033H14 gene |  |  |
|  | 2.7 | nestin | |  |
|  | 2.7 | eva-1 homolog B (C. elegans) |  |  |
|  | 2.7 | tumor necrosis factor (ligand) superfamily, member 12 |  |  |
|  | 2.7 | atypical chemokine receptor 3 |  |  |
|  | 2.7 | gametogenetin |  |  |
|  | 2.7 | RIKEN cDNA 9530082P21 gene |  |  |
|  | 2.7 | tissue inhibitor of metalloproteinase 3 |  |  |
|  | 2.7 | basic leucine zipper transcription factor, ATF-like 3 |  |  |
|  | 2.7 | a disintegrin and metallopeptidase domain 11 |  |  |
|  | 2.7 | IQ motif containing G |  |  |
|  | 2.7 | protein kinase C and casein kinase substrate in neurons 3 |  |  |
|  | 2.7 | heat shock transcription factor 4 |  |  |
|  | 2.7 | RNA binding protein with multiple splicing 2 |  |  |
|  | 2.7 | macrophage scavenger receptor 1 |  |  |
|  | 2.7 | FSHD region gene 2 family member 1 |  |  |
|  | 2.7 | ankyrin repeat and SOCS box-containing 2 |  |  |
|  | 2.7 | matrix metallopeptidase 2 |  |  |
|  | 2.7 | endothelial PAS domain protein 1 |  |  |
|  | 2.7 | brain enriched myelin associated protein 1 |  |  |
|  | 2.7 | lectin, galactose binding, soluble 3 |  |  |
|  | 2.7 | microRNA 7676-1 |  |  |
|  | 2.7 | solute carrier family 4 (anion exchanger), member 4 |  |  |
|  | 2.7 | cyclin-dependent kinase-like 2 (CDC2-related kinase) |  |  |
|  | 2.7 | archaelysin family metallopeptidase 1 |  |  |
|  | 2.7 | calmodulin-like 4 |  |  |
|  | 2.7 | growth hormone |  |  |
|  | 2.7 | serine (or cysteine) peptidase inhibitor, clade A, member 3G |  |  |
|  | 2.7 | Ras association (RalGDS/AF-6) and pleckstrin homology domains 1 |  |  |
|  | 2.7 | ras homolog family member Q |  |  |
|  | 2.7 | RUN and FYVE domain containing 4 |  |  |
|  | 2.7 | predicted gene 16201 |  |  |
|  | 2.7 | collectrin, amino acid transport regulator |  |  |
|  | 2.7 | a disintegrin-like and metallopeptidase (reprolysin type) with thrombospondin type 1 motif, 12 |  |  |
|  | 2.7 | phospholipase D2 |  |  |
|  | 2.7 | ALS2 C-terminal like |  |  |
|  | 2.7 | docking protein 7 |  |  |
|  | 2.7 | NIMA (never in mitosis gene a)-related expressed kinase 6 |  |  |
|  | 2.7 | cyclin-dependent kinase 20 |  |  |
|  | 2.7 | ral guanine nucleotide dissociation stimulator |  |  |
|  | 2.7 | solute carrier family 2 (facilitated glucose transporter), member 1 |  |  |
|  | 2.8 | uridine phosphorylase 1 |  |  |
|  | 2.8 | FERM and PDZ domain containing 1 |  |  |
|  | 2.8 | melanogenesis associated transcription factor |  |  |
|  | 2.8 | heme binding protein 2 |  |  |
|  | 2.8 | leucine rich repeat containing 2 |  |  |
|  | 2.8 | G protein regulated inducer of neurite outgrowth 2 |  |  |
|  | 2.8 | FERM domain containing 4B |  |  |
|  | 2.8 | TBC1 domain family, member 4 |  |  |
|  | 2.8 | epidermal growth factor-containing fibulin-like extracellular matrix protein 2 |  |  |
|  | 2.8 | solute carrier family 37 (glycerol-3-phosphate transporter), member 3 pseudogene |  |  |
|  | 2.8 | AE binding protein 1 |  |  |
|  | 2.8 | olfactomedin 1 |  |  |
|  | 2.8 | serine (or cysteine) peptidase inhibitor, clade E, member 1 |  |  |
|  | 2.8 | breast cancer anti-estrogen resistance 3 |  |  |
|  | 2.8 | sema domain, transmembrane domain (TM), and cytoplasmic domain, (semaphorin) 6D |  |  |
|  | 2.8 | RIKEN cDNA F730043M19 gene |  |  |
|  | 2.8 | insulin-like 6 |  |  |
|  | 2.8 | adaptor protein, phosphotyrosine interaction, PH domain and leucine zipper containing 2 |  |  |
|  | 2.8 | cation channel sperm associated auxiliary subunit gamma 2 |  |  |
|  | 2.8 | 4-hydroxy-2-oxoglutarate aldolase 1 |  |  |
|  | 2.8 | RIKEN cDNA 4921525O09 gene |  |  |
|  | 2.8 | procollagen-proline, 2-oxoglutarate 4-dioxygenase (proline 4-hydroxylase), alpha 1 polypeptide |  |  |
|  | 2.8 | Cd200 receptor 2 |  |  |
|  | 2.8 | solute carrier family 2 (facilitated glucose transporter), member 13 |  |  |
|  | 2.8 | heme oxygenase 1 |  |  |
|  | 2.8 | sorting nexin 8 |  |  |
|  | 2.9 | transmembrane and tetratricopeptide repeat containing 2 |  |  |
|  | 2.9 | tumor protein p53 pathway corepressor 1 |  |  |
|  | 2.9 | phospholipase A2, group IVF |  |  |
|  | 2.9 | predicted gene 12758 |  |  |
|  | 2.9 | cytochrome P450, family 4, subfamily f, polypeptide 37 |  |  |
|  | 2.9 | RNA binding protein, fox-1 homolog (C. elegans) 2 |  |  |
|  | 2.9 | FMS-like tyrosine kinase 1 |  |  |
|  | 2.9 | piwi-like RNA-mediated gene silencing 2 |  |  |
|  | 2.9 | adenylate kinase 4 |  |  |
|  | 2.9 | retinoic acid early transcript 1E |  |  |
|  | 2.9 | N-acetylneuraminate pyruvate lyase |  |  |
|  | 2.9 | cystatin B | |  |
|  | 2.9 | aldehyde dehydrogenase 1 family, member L1 |  |  |
|  | 2.9 | ephrin A5 | |  |
|  | 2.9 | aldehyde dehydrogenase family 1, subfamily A2 |  |  |
|  | 2.9 | endothelin 1 |  |  |
|  | 2.9 | roundabout guidance receptor 3 |  |  |
|  | 2.9 | spindlin family, member 4 |  |  |
|  | 2.9 | cytochrome P450, family 4, subfamily f, polypeptide 16 |  |  |
|  | 2.9 | scinderin | |  |
|  | 2.9 | transmembrane protein 106A |  |  |
|  | 2.9 | beta-1,4-N-acetyl-galactosaminyl transferase 2 |  |  |
|  | 2.9 | renin binding protein |  |  |
|  | 2.9 | calpain 13 | |  |
|  | 2.9 | cathepsin K | |  |
|  | 2.9 | guanylate cyclase 1, soluble, alpha 1 |  |  |
|  | 2.9 | vasohibin 1 | |  |
|  | 2.9 | guanine nucleotide binding protein (G protein), gamma 4 |  |  |
|  | 2.9 | ring finger protein 150 |  |  |
|  | 2.9 | phospholipase A2, group V |  |  |
|  | 2.9 | basic leucine zipper transcription factor, ATF-like 2 |  |  |
|  | 2.9 | transcription factor EC |  |  |
|  | 2.9 | RAS-like, family 2, locus 9 |  |  |
|  | 2.9 | multiple EGF-like-domains 10 |  |  |
|  | 2.9 | glutamate receptor, metabotropic 6 |  |  |
|  | 3.0 | transmembrane inner ear |  |  |
|  | 3.0 | prostaglandin E synthase 3 like |  |  |
|  | 3.0 | granzyme B | |  |
|  | 3.0 | protein phosphatase 2, regulatory subunit B'', alpha |  |  |
|  | 3.0 | solute carrier family 39 (zinc transporter), member 4 |  |  |
|  | 3.0 | exostosin-like glycosyltransferase 1 |  |  |
|  | 3.0 | forkhead-associated (FHA) phosphopeptide binding domain 1 |  |  |
|  | 3.0 | a disintegrin and metallopeptidase domain 9 (meltrin gamma) |  |  |
|  | 3.0 | predicted gene, 19951 |  |  |
|  | 3.0 | sodium channel, voltage-gated, type III, beta |  |  |
|  | 3.0 | insulin receptor-related receptor |  |  |
|  | 3.0 | RIKEN cDNA 2900079G21 gene |  |  |
|  | 3.0 | cAMP responsive element binding protein 3-like 4 |  |  |
|  | 3.0 | caspase 6 | |  |
|  | 3.0 | RIKEN cDNA 4933411E08 gene |  |  |
|  | 3.0 | threonine aldolase 1 |  |  |
|  | 3.0 | Ngfi-A binding protein 2 |  |  |
|  | 3.0 | collagen, type XII, alpha 1 |  |  |
|  | 3.0 | RIKEN cDNA 2210408F21 gene |  |  |
|  | 3.0 | cathepsin S | |  |
|  | 3.0 | dipeptidase 2 |  |  |
|  | 3.0 | phosphatase and actin regulator 1 |  |  |
|  | 3.0 | Rous sarcoma oncogene |  |  |
|  | 3.0 | transforming growth factor, beta 3 |  |  |
|  | 3.0 | laccase domain containing 1 |  |  |
|  | 3.0 | syndecan 4 | |  |
|  | 3.0 | uridine phosphorylase 2 |  |  |
|  | 3.0 | cytochrome P450, family 26, subfamily b, polypeptide 1 |  |  |
|  | 3.0 | leucine rich repeat containing 32 |  |  |
|  | 3.0 | spermatogenesis associated, serine-rich 2 |  |  |
|  | 3.1 | microRNA 21a |  |  |
|  | 3.1 | transmembrane protein 144 |  |  |
|  | 3.1 | SEC14 and spectrin domains 1 |  |  |
|  | 3.1 | prostaglandin F2 receptor negative regulator |  |  |
|  | 3.1 | nectin cell adhesion molecule 2 |  |  |
|  | 3.1 | receptor (calcitonin) activity modifying protein 3 |  |  |
|  | 3.1 | actin-related protein 2/3 complex inhibitor |  |  |
|  | 3.1 | RIKEN cDNA 6430571L13 gene |  |  |
|  | 3.1 | DIX domain containing 1 |  |  |
|  | 3.1 | interferon induced transmembrane protein 1 |  |  |
|  | 3.1 | CD68 antigen |  |  |
|  | 3.1 | interleukin 13 |  |  |
|  | 3.1 | RAB33A, member RAS oncogene family |  |  |
|  | 3.1 | secretory carrier membrane protein 5 |  |  |
|  | 3.1 | nebulin | |  |
|  | 3.1 | zinc finger protein 750 |  |  |
|  | 3.1 | myogenesis regulating glycosidase (putative) |  |  |
|  | 3.1 | transgelin 3 | |  |
|  | 3.1 | tropomodulin 2 |  |  |
|  | 3.1 | myoferlin | |  |
|  | 3.1 | caspase 12 | |  |
|  | 3.1 | toll-like receptor 4 |  |  |
|  | 3.1 | solute carrier family 39 (zinc transporter), member 2 |  |  |
|  | 3.1 | dedicator of cytokinesis 4 |  |  |
|  | 3.1 | ring finger protein 180 |  |  |
|  | 3.1 | carbohydrate sulfotransferase 14 |  |  |
|  | 3.1 | integrin alpha X |  |  |
|  | 3.1 | tribbles pseudokinase 3 |  |  |
|  | 3.1 | Eph receptor B3 |  |  |
|  | 3.1 | activating transcription factor 5 |  |  |
|  | 3.1 | BPI fold containing family C |  |  |
|  | 3.1 | WT1 interacting protein |  |  |
|  | 3.1 | ring finger protein 128 |  |  |
|  | 3.1 | procollagen-proline, 2-oxoglutarate 4-dioxygenase (proline 4-hydroxylase), alpha II polypeptide |  |  |
|  | 3.2 | phosphodiesterase 10A |  |  |
|  | 3.2 | leupaxin | |  |
|  | 3.2 | bromodomain containing 3, opposite strand |  |  |
|  | 3.2 | B cell leukemia/lymphoma 2 related protein A1b |  |  |
|  | 3.2 | leucine rich repeat containing 66 |  |  |
|  | 3.2 | predicted gene 12589 |  |  |
|  | 3.2 | t-complex 11 like 1 |  |  |
|  | 3.2 | solute carrier family 30 (zinc transporter), member 4 |  |  |
|  | 3.2 | arrestin domain containing 4 |  |  |
|  | 3.2 | protocadherin 7 |  |  |
|  | 3.2 | TBC1 domain family, member 16 |  |  |
|  | 3.2 | cyclin J-like | |  |
|  | 3.2 | coagulation factor X |  |  |
|  | 3.2 | fibroblast growth factor 11 |  |  |
|  | 3.2 | intraflagellar transport 43 |  |  |
|  | 3.2 | PTK6 protein tyrosine kinase 6 |  |  |
|  | 3.2 | cytochrome P450, family 11, subfamily a, polypeptide 1 |  |  |
|  | 3.2 | alpha 1,4-galactosyltransferase |  |  |
|  | 3.2 | uronyl-2-sulfotransferase |  |  |
|  | 3.2 | interleukin 4 receptor, alpha |  |  |
|  | 3.2 | inhibin beta-B |  |  |
|  | 3.2 | CD200 receptor 1 |  |  |
|  | 3.2 | solute carrier family 18 (vesicular monoamine), member 2 |  |  |
|  | 3.3 | ArfGAP with dual PH domains 2 |  |  |
|  | 3.3 | epithelial cell adhesion molecule |  |  |
|  | 3.3 | RIKEN cDNA 2610528J11 gene |  |  |
|  | 3.3 | MER proto-oncogene tyrosine kinase |  |  |
|  | 3.3 | interleukin 1 alpha |  |  |
|  | 3.3 | adhesion G protein-coupled receptor G2 |  |  |
|  | 3.3 | solute carrier family 17 (sodium-dependent inorganic phosphate cotransporter), member 8 |  |  |
|  | 3.3 | retinoic acid early transcript 1, alpha |  |  |
|  | 3.3 | matrix metallopeptidase 27 |  |  |
|  | 3.3 | T cell receptor gamma, constant 1 |  |  |
|  | 3.3 | dermatan sulfate epimerase |  |  |
|  | 3.3 | glutamate receptor, metabotropic 4 |  |  |
|  | 3.3 | predicted gene, 46069 |  |  |
|  | 3.3 | transmembrane protein 45a |  |  |
|  | 3.3 | SET binding factor 2 |  |  |
|  | 3.3 | sterile alpha motif domain containing 15 |  |  |
|  | 3.4 | histocompatibility 2, M region locus 2 |  |  |
|  | 3.4 | interleukin 4 |  |  |
|  | 3.4 | src homology 2 domain-containing transforming protein C3 |  |  |
|  | 3.4 | B cell leukemia/lymphoma 2 related protein A1d |  |  |
|  | 3.4 | RIKEN cDNA A430088P11 gene |  |  |
|  | 3.4 | Fc receptor, IgG, low affinity IIb |  |  |
|  | 3.4 | aldo-keto reductase family 1, member C18 |  |  |
|  | 3.4 | dipeptidase 2 |  |  |
|  | 3.4 | free fatty acid receptor 4 |  |  |
|  | 3.4 | taxilin beta | |  |
|  | 3.4 | angiopoietin-like 2 |  |  |
|  | 3.4 | ATPase, Na+/K+ transporting, alpha 2 polypeptide |  |  |
|  | 3.4 | colony stimulating factor 2 receptor, beta 2, low-affinity (granulocyte-macrophage) |  |  |
|  | 3.4 | niban apoptosis regulator 2 |  |  |
|  | 3.4 | docking protein 6 |  |  |
|  | 3.4 | predicted gene 4610 |  |  |
|  | 3.4 | advillin | |  |
|  | 3.4 | ets variant gene 5 pseudogene |  |  |
|  | 3.4 | neuronal tyrosine-phophorylated phosphoinositide 3-kinase adaptor 2 |  |  |
|  | 3.4 | serine (or cysteine) peptidase inhibitor, clade B, member 8 |  |  |
|  | 3.4 | protein disulfide isomerase associated 4 |  |  |
|  | 3.4 | ribonuclease, RNase A family 4 |  |  |
|  | 3.4 | calcium channel, voltage-dependent, N type, alpha 1B subunit |  |  |
|  | 3.4 | butyrophilin-like 1 |  |  |
|  | 3.5 | platelet factor 4 |  |  |
|  | 3.5 | cytochrome P450, family 4, subfamily x, polypeptide 1, opposite strand |  |  |
|  | 3.5 | retinoic acid induced 14 |  |  |
|  | 3.5 | six transmembrane epithelial antigen of prostate 2 |  |  |
|  | 3.5 | protein kinase, cAMP dependent regulatory, type I beta |  |  |
|  | 3.5 | a disintegrin-like and metallopeptidase (reprolysin type) with thrombospondin type 1 motif, 4 |  |  |
|  | 3.5 | adrenomedullin |  |  |
|  | 3.5 | hepatocyte growth factor activator |  |  |
|  | 3.5 | predicted gene, 31223 |  |  |
|  | 3.5 | RIKEN cDNA 2510009E07 gene |  |  |
|  | 3.5 | hippocalcin-like 4 |  |  |
|  | 3.5 | C-type lectin domain family 10, member A |  |  |
|  | 3.5 | stearoyl-Coenzyme A desaturase 2 |  |  |
|  | 3.5 | apolipoprotein L 7a |  |  |
|  | 3.5 | solute carrier family 7 (cationic amino acid transporter, y+ system), member 8 |  |  |
|  | 3.5 | dedicator of cyto-kinesis 3 |  |  |
|  | 3.5 | solute carrier family 38, member 6 |  |  |
|  | 3.5 | ral guanine nucleotide dissociation stimulator,-like 1 |  |  |
|  | 3.6 | mucolipin 2 | |  |
|  | 3.6 | fidgetin-like 2 |  |  |
|  | 3.6 | microRNA 5114 |  |  |
|  | 3.6 | homeostatic iron regulator |  |  |
|  | 3.6 | plakophilin 2 |  |  |
|  | 3.6 | sodium channel, voltage-gated, type IV, alpha |  |  |
|  | 3.6 | T cell-interacting, activating receptor on myeloid cells 1 |  |  |
|  | 3.6 | cellular repressor of E1A-stimulated genes 2 |  |  |
|  | 3.6 | G protein-coupled receptor 68 |  |  |
|  | 3.6 | transglutaminase 2, C polypeptide |  |  |
|  | 3.6 | RIKEN cDNA 4932441J04 gene |  |  |
|  | 3.6 | matrix metallopeptidase 14 (membrane-inserted) |  |  |
|  | 3.6 | ets variant 5 | |  |
|  | 3.6 | Na+/K+ transporting ATPase interacting 1 |  |  |
|  | 3.6 | dynein, axonemal, heavy chain 2 |  |  |
|  | 3.6 | chemokine (C-X-C motif) receptor 1 |  |  |
|  | 3.6 | chemokine (C-C motif) ligand 9 |  |  |
|  | 3.6 | cytokine inducible SH2-containing protein |  |  |
|  | 3.6 | filamin binding LIM protein 1 |  |  |
|  | 3.6 | PDZ and LIM domain 4 |  |  |
|  | 3.6 | gastrulation brain homeobox 2 |  |  |
|  | 3.6 | leukotriene C4 synthase |  |  |
|  | 3.6 | cyclin-dependent kinase 18 |  |  |
|  | 3.6 | interleukin 13 receptor, alpha 2 |  |  |
|  | 3.6 | chemokine (C-C motif) receptor 5 |  |  |
|  | 3.6 | RIKEN cDNA A930007I19 gene |  |  |
|  | 3.6 | ATP-binding cassette, sub-family A (ABC1), member 1 |  |  |
|  | 3.7 | FAT atypical cadherin 3 |  |  |
|  | 3.7 | pyroglutamyl-peptidase I-like |  |  |
|  | 3.7 | ankyrin repeat domain 37 |  |  |
|  | 3.7 | tweety family member 2 |  |  |
|  | 3.7 | B cell leukemia/lymphoma 2 related protein A1a |  |  |
|  | 3.7 | neuregulin 1 |  |  |
|  | 3.7 | enoyl Coenzyme A hydratase domain containing 3 |  |  |
|  | 3.7 | pleckstrin homology domain containing, family F (with FYVE domain) member 1 |  |  |
|  | 3.7 | nucleolar protein 3 (apoptosis repressor with CARD domain) |  |  |
|  | 3.7 | UDP-GlcNAc:betaGal beta-1,3-N-acetylglucosaminyltransferase 7 |  |  |
|  | 3.7 | ets variant 4 | |  |
|  | 3.7 | globoside alpha-1,3-N-acetylgalactosaminyltransferase 1 |  |  |
|  | 3.7 | SH3 and cysteine rich domain 2 |  |  |
|  | 3.7 | sphingomyelin phosphodiesterase, acid-like 3B |  |  |
|  | 3.7 | predicted gene 12722 |  |  |
|  | 3.7 | lysyl oxidase-like 4 |  |  |
|  | 3.7 | nyctalopin | |  |
|  | 3.7 | DNA-damage-inducible transcript 4-like |  |  |
|  | 3.8 | complement component 1, q subcomponent, alpha polypeptide |  |  |
|  | 3.8 | unc-13 homolog A |  |  |
|  | 3.8 | heat shock protein 8 |  |  |
|  | 3.8 | RUN and SH3 domain containing 2 |  |  |
|  | 3.8 | placenta expressed transcript 1 |  |  |
|  | 3.8 | hes family bHLH transcription factor 7 |  |  |
|  | 3.8 | formin 1 | |  |
|  | 3.8 | proprotein convertase subtilisin/kexin type 9 |  |  |
|  | 3.8 | matrix metallopeptidase 19 |  |  |
|  | 3.8 | CEA cell adhesion molecule 19 |  |  |
|  | 3.8 | ARFGEF family member 3 |  |  |
|  | 3.8 | BCL2/adenovirus E1B interacting protein 3 |  |  |
|  | 3.8 | carbonic anhydrase 5b, mitochondrial |  |  |
|  | 3.8 | complement component 1, q subcomponent, C chain |  |  |
|  | 3.8 | chymase 1, mast cell |  |  |
|  | 3.8 | cadherin 17 | |  |
|  | 3.8 | chemokine (C-X-C motif) ligand 3 |  |  |
|  | 3.8 | asialoglycoprotein receptor 2 |  |  |
|  | 3.8 | cysteinyl leukotriene receptor 1 |  |  |
|  | 3.8 | lysyl oxidase |  |  |
|  | 3.9 | dynamin 1 | |  |
|  | 3.9 | F-box protein 2 |  |  |
|  | 3.9 | RIKEN cDNA 4933417E11 gene |  |  |
|  | 3.9 | low density lipoprotein-related protein 12 |  |  |
|  | 3.9 | carbonyl reductase 3 |  |  |
|  | 3.9 | predicted gene 13546 |  |  |
|  | 3.9 | leucine rich adaptor protein 1 |  |  |
|  | 3.9 | copine VIII | |  |
|  | 3.9 | retinoic acid receptor responder (tazarotene induced) 1 |  |  |
|  | 3.9 | RIKEN cDNA 9130019P16 gene |  |  |
|  | 3.9 | solute carrier family 1 (glutamate/neutral amino acid transporter), member 4 |  |  |
|  | 3.9 | potassium voltage-gated channel, subfamily H (eag-related), member 3 |  |  |
|  | 3.9 | RIKEN cDNA G530011O06 gene |  |  |
|  | 4.0 | hepatitis A virus cellular receptor 2 |  |  |
|  | 4.0 | recoverin | |  |
|  | 4.0 | leukemia inhibitory factor |  |  |
|  | 4.0 | kinesin family member 1A |  |  |
|  | 4.0 | sel-1 suppressor of lin-12-like 3 (C. elegans) |  |  |
|  | 4.0 | erythrocyte membrane protein band 4.1 like 1 |  |  |
|  | 4.0 | zinc finger, MYND-type containing 15 |  |  |
|  | 4.0 | CD200 receptor 4 |  |  |
|  | 4.0 | cannabinoid receptor interacting protein 1 |  |  |
|  | 4.0 | lipase, endothelial |  |  |
|  | 4.0 | MAF bZIP transcription factor B |  |  |
|  | 4.0 | family with sequence similarity 170, member B |  |  |
|  | 4.0 | platelet-derived growth factor, C polypeptide |  |  |
|  | 4.0 | coiled-coil domain containing 89 |  |  |
|  | 4.0 | aldolase C, fructose-bisphosphate |  |  |
|  | 4.0 | suppressor of cytokine signaling 2 |  |  |
|  | 4.0 | microRNA 704 |  |  |
|  | 4.1 | predicted gene, 16897 |  |  |
|  | 4.1 | tumor necrosis factor receptor superfamily, member 9 |  |  |
|  | 4.1 | transmembrane O-methyltransferase |  |  |
|  | 4.1 | cytochrome P450, family 7, subfamily b, polypeptide 1 |  |  |
|  | 4.1 | solute carrier family 7 (cationic amino acid transporter, y+ system), member 3 |  |  |
|  | 4.1 | ribonuclease P/MRP 25 subunit |  |  |
|  | 4.1 | chemokine (C-C motif) ligand 2 |  |  |
|  | 4.1 | triggering receptor expressed on myeloid cells 2 |  |  |
|  | 4.1 | RIKEN cDNA D030025E07 gene |  |  |
|  | 4.1 | MAF bZIP transcription factor |  |  |
|  | 4.1 | neural cell adhesion molecule 2 |  |  |
|  | 4.1 | cadherin 22 | |  |
|  | 4.2 | neuropilin 2 | |  |
|  | 4.2 | serine (or cysteine) peptidase inhibitor, clade A, member 3I |  |  |
|  | 4.2 | secreted phosphoprotein 1 |  |  |
|  | 4.2 | Tmem51 opposite strand 1 |  |  |
|  | 4.2 | annexin A4 | |  |
|  | 4.2 | suppression of tumorigenicity 18 |  |  |
|  | 4.2 | homeobox A1 |  |  |
|  | 4.2 | purinergic receptor P2X, ligand-gated ion channel, 6 |  |  |
|  | 4.2 | phospholipase A2 inhibitor and LY6/PLAUR domain containing |  |  |
|  | 4.2 | basic helix-loop-helix ARNT like 2 |  |  |
|  | 4.2 | egl-9 family hypoxia-inducible factor 3 |  |  |
|  | 4.2 | fascin actin-bundling protein 1 |  |  |
|  | 4.2 | STRA6-like | |  |
|  | 4.2 | potassium channel, subfamily K, member 13 |  |  |
|  | 4.2 | atonal bHLH transcription factor 8 |  |  |
|  | 4.2 | pyrimidinergic receptor P2Y, G-protein coupled, 6 |  |  |
|  | 4.3 | extracellular matrix protein 1 |  |  |
|  | 4.3 | chemokine (C-X-C motif) ligand 16 |  |  |
|  | 4.3 | fibroblast growth factor 23 |  |  |
|  | 4.3 | interleukin 12b |  |  |
|  | 4.3 | serine (or cysteine) peptidase inhibitor, clade A, member 3H |  |  |
|  | 4.3 | septin 3 | |  |
|  | 4.3 | ecto-NOX disulfide-thiol exchanger 1 |  |  |
|  | 4.3 | dihydropyrimidinase-like 5 |  |  |
|  | 4.3 | chemerin chemokine-like receptor 1 |  |  |
|  | 4.3 | fibroblast growth factor 2 |  |  |
|  | 4.3 | EGF-like repeats and discoidin I-like domains 3 |  |  |
|  | 4.3 | ets variant 1 | |  |
|  | 4.3 | neurotrophic tyrosine kinase, receptor, type 1 |  |  |
|  | 4.3 | suppressor of cytokine signaling 1 |  |  |
|  | 4.3 | FAM20C, golgi associated secretory pathway kinase |  |  |
|  | 4.3 | CTTNBP2 N-terminal like |  |  |
|  | 4.4 | collagen, type XVIII, alpha 1 |  |  |
|  | 4.4 | complement component 1, q subcomponent, beta polypeptide |  |  |
|  | 4.4 | MAS-related GPR, member A6 |  |  |
|  | 4.4 | prostate transmembrane protein, androgen induced 1 |  |  |
|  | 4.4 | cannabinoid receptor 1 (brain) |  |  |
|  | 4.4 | sorting nexing 24 |  |  |
|  | 4.4 | RIKEN cDNA 9130213A22 gene |  |  |
|  | 4.4 | transmembrane protein 92 |  |  |
|  | 4.4 | dipeptidase 3 |  |  |
|  | 4.4 | predicted gene, 34106 |  |  |
|  | 4.4 | tripartite motif-containing 29 |  |  |
|  | 4.4 | cytochrome P450, family 4, subfamily x, polypeptide 1 |  |  |
|  | 4.4 | very low density lipoprotein receptor |  |  |
|  | 4.4 | lysosomal-associated membrane protein family, member 5 |  |  |
|  | 4.4 | mastermind-like domain containing 1 |  |  |
|  | 4.4 | chloride channel, voltage-sensitive 5 |  |  |
|  | 4.4 | zinc finger protein 366 |  |  |
|  | 4.4 | cadherin 1 | |  |
|  | 4.4 | protein tyrosine phosphatase, receptor type, N |  |  |
|  | 4.5 | RIKEN cDNA D930048N14 gene |  |  |
|  | 4.5 | ST8 alpha-N-acetyl-neuraminide alpha-2,8-sialyltransferase 1 |  |  |
|  | 4.5 | phosphodiesterase 11A |  |  |
|  | 4.5 | solute carrier family 36 (proton/amino acid symporter), member 2 |  |  |
|  | 4.5 | cadherin 3 | |  |
|  | 4.5 | RIKEN cDNA 4932438H23 gene |  |  |
|  | 4.5 | insulin-like growth factor 1 |  |  |
|  | 4.5 | RIKEN cDNA F830016B08 gene |  |  |
|  | 4.5 | neuronal PAS domain protein 2 |  |  |
|  | 4.5 | plexin A1 | |  |
|  | 4.5 | glutathione S-transferase, mu 2 |  |  |
|  | 4.5 | plexin domain containing 2 |  |  |
|  | 4.6 | pregnancy specific beta-1-glycoprotein 17 |  |  |
|  | 4.6 | galactose-3-O-sulfotransferase 2 |  |  |
|  | 4.6 | predicted gene, 19510 |  |  |
|  | 4.6 | solute carrier family 6 (neurotransmitter transporter, creatine), member 8 |  |  |
|  | 4.6 | Ly6/Plaur domain containing 1 |  |  |
|  | 4.6 | predicted gene 12022 |  |  |
|  | 4.6 | myosin, heavy polypeptide 3, skeletal muscle, embryonic |  |  |
|  | 4.6 | integrin beta 8 |  |  |
|  | 4.6 | aldehyde dehydrogenase family 1, subfamily A3 |  |  |
|  | 4.6 | chromodomain helicase DNA binding protein 5 |  |  |
|  | 4.6 | eukaryotic translation elongation factor 1 alpha 2 |  |  |
|  | 4.6 | H1.8 linker histone |  |  |
|  | 4.6 | ERBB receptor feedback inhibitor 1 |  |  |
|  | 4.6 | predicted gene 15413 |  |  |
|  | 4.6 | delta like non-canonical Notch ligand 1 |  |  |
|  | 4.6 | ATP binding cassette subfamily G member 8 |  |  |
|  | 4.6 | tetratricopeptide repeat domain 9 |  |  |
|  | 4.7 | predicted gene 13470 |  |  |
|  | 4.7 | myozenin 1 | |  |
|  | 4.7 | indoleamine 2,3-dioxygenase 2 |  |  |
|  | 4.7 | RIKEN cDNA 2610300M13 gene |  |  |
|  | 4.7 | golgi associated kinase 1B |  |  |
|  | 4.7 | angiogenin, ribonuclease A family, member 2 |  |  |
|  | 4.7 | early growth response 2 |  |  |
|  | 4.7 | TOG array regulator of axonemal microtubules 2 |  |  |
|  | 4.7 | splA/ryanodine receptor domain and SOCS box containing 1 |  |  |
|  | 4.7 | wingless-type MMTV integration site family, member 5A |  |  |
|  | 4.7 | cathepsin L | |  |
|  | 4.7 | legumain | |  |
|  | 4.7 | sema domain, immunoglobulin domain (Ig), short basic domain, secreted, (semaphorin) 3C |  |  |
|  | 4.8 | ATP-binding cassette, sub-family A (ABC1), member 4 |  |  |
|  | 4.8 | osteoclast stimulatory transmembrane protein |  |  |
|  | 4.8 | myosin, light chain 10, regulatory |  |  |
|  | 4.8 | gap junction protein, alpha 1 |  |  |
|  | 4.8 | C-type lectin domain family 4, member n |  |  |
|  | 4.8 | olfactory receptor family 5 subfamily V member 1B |  |  |
|  | 4.8 | olfactory receptor family 2 subfamily AG member 18 |  |  |
|  | 4.8 | serine (or cysteine) peptidase inhibitor, clade B (ovalbumin), member 12 |  |  |
|  | 4.8 | oxoglutarate dehydrogenase-like |  |  |
|  | 4.8 | serine (or cysteine) peptidase inhibitor, clade B, member 9b |  |  |
|  | 4.8 | tetratricopeptide repeat, ankyrin repeat and coiled-coil containing 2 |  |  |
|  | 4.8 | enolase 2, gamma neuronal |  |  |
|  | 4.9 | glutathione S-transferase, alpha 2 (Yc2) |  |  |
|  | 4.9 | ribosomal protein S2 pseudogene |  |  |
|  | 4.9 | potassium inwardly-rectifying channel, subfamily J, member 4 |  |  |
|  | 4.9 | predicted gene, 33100 |  |  |
|  | 4.9 | ribosomal modification protein rimK-like family member A |  |  |
|  | 4.9 | phosphatase domain containing, paladin 1 |  |  |
|  | 4.9 | membrane integral NOTCH2 associated receptor 1 |  |  |
|  | 4.9 | glutamic pyruvate transaminase (alanine aminotransferase) 2 |  |  |
|  | 4.9 | protease, serine 46 |  |  |
|  | 5.0 | SH3 and PX domains 2B |  |  |
|  | 5.0 | transmembrane 4 superfamily member 5 |  |  |
|  | 5.0 | predicted gene, 20767 |  |  |
|  | 5.0 | RIKEN cDNA F830045P16 gene |  |  |
|  | 5.0 | diacylglycerol lipase, alpha |  |  |
|  | 5.0 | small proline-rich protein 2A3 |  |  |
|  | 5.0 | MISP family member 3 |  |  |
|  | 5.0 | fatty acid binding protein 5, epidermal |  |  |
|  | 5.1 | synaptotagmin XIII |  |  |
|  | 5.1 | DLG associated protein 3 |  |  |
|  | 5.1 | RIKEN cDNA 4930579C12 gene |  |  |
|  | 5.1 | killer cell lectin-like receptor subfamily G, member 2 |  |  |
|  | 5.1 | CEA cell adhesion molecule 15 |  |  |
|  | 5.1 | solute carrier family 6 (neurotransmitter transporter, L-proline), member 7 |  |  |
|  | 5.1 | kallikrein 1-related peptidase b9 |  |  |
|  | 5.1 | tumor necrosis factor, alpha-induced protein 8-like 3 |  |  |
|  | 5.1 | predicted gene, 39822 |  |  |
|  | 5.1 | transmembrane 4 L six family member 19 |  |  |
|  | 5.1 | aldo-keto reductase family 1, member B8 |  |  |
|  | 5.1 | serine (or cysteine) peptidase inhibitor, clade A, member 3B |  |  |
|  | 5.2 | B cell leukemia/lymphoma 2 related protein A1c |  |  |
|  | 5.2 | cell migration inducing protein, hyaluronan binding |  |  |
|  | 5.2 | aquaporin 3 | |  |
|  | 5.2 | Ras association and DIL domains |  |  |
|  | 5.2 | ribonuclease, RNase A family, 2A (liver, eosinophil-derived neurotoxin) |  |  |
|  | 5.2 | epithelial splicing regulatory protein 2 |  |  |
|  | 5.2 | antizyme inhibitor 2 |  |  |
|  | 5.2 | RIKEN cDNA A230028O05 gene |  |  |
|  | 5.3 | membrane-spanning 4-domains, subfamily A, member 6D |  |  |
|  | 5.3 | kelch-like 33 |  |  |
|  | 5.3 | platelet derived growth factor, alpha |  |  |
|  | 5.3 | chemokine (C-C motif) ligand 22 |  |  |
|  | 5.3 | complement component 3a receptor 1 |  |  |
|  | 5.3 | Rho GTPase activating protein 8 |  |  |
|  | 5.3 | microRNA 511 |  |  |
|  | 5.3 | regulator of G-protein signaling 11 |  |  |
|  | 5.3 | prune homolog 2 |  |  |
|  | 5.4 | TNFAIP3 interacting protein 3 |  |  |
|  | 5.4 | carbonic anhydrase 8 |  |  |
|  | 5.4 | sushi domain containing 4 |  |  |
|  | 5.4 | proline rich 15 |  |  |
|  | 5.4 | transmembrane protein 37 |  |  |
|  | 5.4 | gastric inhibitory polypeptide receptor |  |  |
|  | 5.5 | macrophage galactose N-acetyl-galactosamine specific lectin 2 |  |  |
|  | 5.5 | wingless-type MMTV integration site family, member 9A |  |  |
|  | 5.5 | ENTH domain containing 1 |  |  |
|  | 5.5 | membrane-spanning 4-domains, subfamily A, member 4A |  |  |
|  | 5.5 | coiled-coil domain containing 33 |  |  |
|  | 5.5 | ovo like zinc finger 2 |  |  |
|  | 5.5 | WD repeat domain 54 |  |  |
|  | 5.5 | stabilin 1 | |  |
|  | 5.5 | superoxide dismutase 3, extracellular |  |  |
|  | 5.5 | membrane-spanning 4-domains, subfamily A, member 7 |  |  |
|  | 5.6 | CD163 antigen |  |  |
|  | 5.6 | mab-21-like 3 |  |  |
|  | 5.6 | solute carrier family 9 (sodium/hydrogen exchanger), member 4 |  |  |
|  | 5.6 | DS cell adhesion molecule like 1 |  |  |
|  | 5.6 | calcium channel, voltage-dependent, beta 3 subunit |  |  |
|  | 5.6 | predicted gene 2396 |  |  |
|  | 5.6 | sine oculis-related homeobox 1 |  |  |
|  | 5.6 | kallikrein 1-related peptidase b11 |  |  |
|  | 5.7 | dendrocyte expressed seven transmembrane protein |  |  |
|  | 5.7 | pre T cell antigen receptor alpha |  |  |
|  | 5.7 | solute carrier family 39 (zinc transporter), member 12 |  |  |
|  | 5.7 | synapsin III | |  |
|  | 5.7 | DNA methyltransferase 3A, opposite strand |  |  |
|  | 5.7 | RIKEN cDNA F630040K05 gene |  |  |
|  | 5.7 | carbonyl reductase 2 |  |  |
|  | 5.7 | placental growth factor |  |  |
|  | 5.8 | SH3 domain containing ring finger 3 |  |  |
|  | 5.8 | aristaless related homeobox |  |  |
|  | 5.8 | calcium channel, voltage-dependent, gamma subunit 8 |  |  |
|  | 5.8 | WW, C2 and coiled-coil domain containing 1 |  |  |
|  | 5.8 | BTB (POZ) domain containing 17 |  |  |
|  | 5.8 | RIKEN cDNA 5730435O14 gene |  |  |
|  | 5.8 | glycoprotein (transmembrane) nmb |  |  |
|  | 5.9 | matrix metallopeptidase 13 |  |  |
|  | 5.9 | podoplanin | |  |
|  | 5.9 | myelin regulatory factor |  |  |
|  | 5.9 | chemokine (C-C motif) ligand 17 |  |  |
|  | 5.9 | neurexophilin and PC-esterase domain family, member 5 |  |  |
|  | 6.0 | transition protein 2 |  |  |
|  | 6.0 | spermatogenesis associated 18 |  |  |
|  | 6.0 | transmembrane protein 150C |  |  |
|  | 6.0 | sodium channel, voltage-gated, type I, alpha |  |  |
|  | 6.0 | RIKEN cDNA D830013O20 gene |  |  |
|  | 6.0 | ankyrin repeat domain 55 |  |  |
|  | 6.0 | predicted gene 5833 |  |  |
|  | 6.1 | CD209e antigen |  |  |
|  | 6.1 | phospholipase A2, group IIE |  |  |
|  | 6.1 | cadherin-related family member 1 |  |  |
|  | 6.1 | lysozyme-like 4 |  |  |
|  | 6.1 | ATPase, H+ transporting, lysosomal V0 subunit D2 |  |  |
|  | 6.2 | TRPM8 channel-associated factor 2 |  |  |
|  | 6.2 | interleukin 4 induced 1 |  |  |
|  | 6.2 | TAFA chemokine like family member 3 |  |  |
|  | 6.2 | sodium channel, voltage-gated, type IX, alpha |  |  |
|  | 6.2 | Fc receptor-like B |  |  |
|  | 6.3 | NADPH-dependent carbonyl reductase pseudogene |  |  |
|  | 6.3 | tumor necrosis factor receptor superfamily, member 11b (osteoprotegerin) |  |  |
|  | 6.3 | transmembrane protein 26 |  |  |
|  | 6.4 | membrane-spanning 4-domains, subfamily A, member 14 |  |  |
|  | 6.4 | predicted gene 15056 |  |  |
|  | 6.4 | a disintegrin and metallopeptidase domain 23 |  |  |
|  | 6.4 | N-myc downstream regulated gene 4 |  |  |
|  | 6.4 | small proline-rich protein 2A1 |  |  |
|  | 6.5 | predicted gene 6116 |  |  |
|  | 6.5 | folate receptor 2 (fetal) |  |  |
|  | 6.5 | aryl-hydrocarbon receptor repressor |  |  |
|  | 6.5 | matrix metallopeptidase 10 |  |  |
|  | 6.5 | V-set and immunoglobulin domain containing 8 |  |  |
|  | 6.6 | predicted gene, 19434 |  |  |
|  | 6.6 | kinase suppressor of ras 2 |  |  |
|  | 6.6 | sodium channel, voltage-gated, type II, alpha |  |  |
|  | 6.7 | histocompatibility 2, M region locus 5 |  |  |
|  | 6.7 | small proline-rich protein 2A2 |  |  |
|  | 6.8 | tissue inhibitor of metalloproteinase 1 |  |  |
|  | 6.8 | Fc receptor like 2 |  |  |
|  | 6.8 | alanyl (membrane) aminopeptidase |  |  |
|  | 6.9 | endothelin receptor type B |  |  |
|  | 6.9 | heparin-binding EGF-like growth factor |  |  |
|  | 6.9 | progesterone receptor |  |  |
|  | 7.0 | zinc finger protein 469 |  |  |
|  | 7.1 | solute carrier family 28 (sodium-coupled nucleoside transporter), member 3 |  |  |
|  | 7.1 | stanniocalcin 2 |  |  |
|  | 7.1 | aldehyde dehydrogenase 1 family, member L2 |  |  |
|  | 7.2 | AHNAK nucleoprotein 2 |  |  |
|  | 7.2 | nitric oxide synthase 2, inducible |  |  |
|  | 7.2 | potassium intermediate/small conductance calcium-activated channel, subfamily N, member 3 |  |  |
|  | 7.2 | inter-alpha trypsin inhibitor, heavy chain 1 |  |  |
|  | 7.3 | cAMP responsive element binding protein 5 |  |  |
|  | 7.3 | mannose receptor, C type 1 |  |  |
|  | 7.3 | programmed cell death 1 ligand 2 |  |  |
|  | 7.4 | Ndufa4, mitochondrial complex associated like 2 |  |  |
|  | 7.4 | claudin 11 | |  |
|  | 7.5 | myotubularin related protein 7 |  |  |
|  | 7.5 | cDNA sequence AF067061 |  |  |
|  | 7.7 | sterile alpha motif domain containing 5 |  |  |
|  | 7.7 | 5-hydroxytryptamine (serotonin) receptor 7 |  |  |
|  | 7.7 | transmembrane protein 171 |  |  |
|  | 7.7 | disabled 2, mitogen-responsive phosphoprotein |  |  |
|  | 7.8 | serum amyloid A 3 |  |  |
|  | 7.8 | fibronectin leucine rich transmembrane protein 2 |  |  |
|  | 7.9 | apolipoprotein L 7c |  |  |
|  | 7.9 | predicted gene 6093 |  |  |
|  | 7.9 | mitochondria localized glutamic acid rich protein |  |  |
|  | 8.1 | coagulation factor VII |  |  |
|  | 8.1 | 5-hydroxytryptamine (serotonin) receptor 2B |  |  |
|  | 8.2 | membrane associated ring-CH-type finger 10 |  |  |
|  | 8.5 | ladinin | |  |
|  | 8.6 | chemokine (C-C motif) ligand 7 |  |  |
|  | 8.6 | purinergic receptor P2X, ligand-gated ion channel, 5 |  |  |
|  | 8.7 | fibronectin leucine rich transmembrane protein 3 |  |  |
|  | 8.8 | RIKEN cDNA 4930512J16 gene |  |  |
|  | 8.9 | retinol binding protein 4, plasma |  |  |
|  | 9.4 | sodium channel, voltage-gated, type III, alpha |  |  |
|  | 9.7 | proline-rich transmembrane protein 4 |  |  |
|  | 9.7 | lipase, family member N |  |  |
|  | 10.2 | solute carrier family 7 (cationic amino acid transporter, y+ system), member 2 |  |  |
|  | 10.5 | serine protease inhibitor, Kunitz type 1 |  |  |
|  | 10.6 | carbonic anhydrase 4 |  |  |
|  | 11.2 | potassium voltage-gated channel, Shal-related family, member 3 |  |  |
|  | 11.4 | cellular communication network factor 3 |  |  |
|  | 11.5 | matrix metallopeptidase 12 |  |  |
|  | 11.8 | chemokine (C-C motif) ligand 12 |  |  |
|  | 12.2 | chemokine (C-C motif) ligand 8 |  |  |
|  | 12.5 | resistin like alpha |  |  |
|  | 13.0 | cholesterol 25-hydroxylase |  |  |
|  | 13.1 | msh homeobox 3 |  |  |
|  | 13.5 | arginase, liver |  |  |
|  | 14.3 | chemokine (C-C motif) ligand 24 |  |  |
| Total 1838 |  |  |  |  |
